# Mendelian randomization study of sleep traits and risk of colorectal cancer

**DOI:** 10.1038/s41598-024-83693-w

**Published:** 2025-04-18

**Authors:** Olympia Dimopoulou, Harriett Fuller, Rebecca C. Richmond, Emmanouil Bouras, Bryony Hayes, Niki Dimou, Neil Murphy, Hermann Brenner, Andrea Gsur, Loic Le Marchand, Victor Moreno, Rish K. Pai, Amanda I. Phipps, Caroline Y. Um, Franzel J. B. van Duijnhoven, Pavel Vodicka, Richard M. Martin, Elizabeth A. Platz, Marc J. Gunter, Ulrike Peters, Sarah J. Lewis, Yin Cao, Konstantinos K. Tsilidis

**Affiliations:** 1 Department of Population Health Sciences, Bristol Medical School, University of Bristol, Bristol, UK; 2 MRC Integrative Epidemiology Unit, Population Health Sciences, Bristol Medical School, University of Bristol, Bristol, UK; 3 Public Health Sciences Division, Fred Hutchinson Cancer Research Center, Seattle, WA USA; 4 Department of Hygiene and Epidemiology, University of Ioannina School of Medicine, Ioannina, Greece; 5 Nutrition and Metabolism Branch, International Agency for Research On Cancer-WHO, Lyon, France; 6 Division of Clinical Epidemiology and Aging Research, German Cancer Research Center (DKFZ), Heidelberg, Germany; 7 Division of Preventive Oncology, German Cancer Research Center (DKFZ), National Center for Tumor Diseases (NCT), Heidelberg, Germany; 8 German Cancer Consortium (DKTK), German Cancer Research Center (DKFZ), Heidelberg, Germany; 9 Center for Cancer Research, Medical University of Vienna, Vienna, Austria; 10 University of Hawaii Cancer Center, Honolulu, HI USA; 11 Oncology Data Analytics Program, Catalan Institute of Oncology-IDIBELL, L’Hospitalet de Llobregat, Barcelona, Spain; 12 CIBER Epidemiología y Salud Pública (CIBERESP), Madrid, Spain; 13 Department of Clinical Sciences, Faculty of Medicine, University of Barcelona, Barcelona, Spain; 14 ONCOBEL Program, Bellvitge Biomedical Research Institute (IDIBELL), L’Hospitalet de Llobregat, Barcelona, Spain; 15 Department of Laboratory Medicine and Pathology, Mayo Clinic Arizona, Scottsdale, AZ USA; 16 Department of Epidemiology, University of Washington, Seattle, WA USA; 17 Population Science Department, American Cancer Society, Atlanta, GA USA; 18 Division of Human Nutrition, Wageningen University and Research, Wageningen, The Netherlands; 19 Department of Molecular Biology of Cancer, Institute of Experimental Medicine of the Czech Academy of Sciences, Prague, Czech Republic; 20 Institute of Biology and Medical Genetics, First Faculty of Medicine, Charles University, Prague, Czech Republic; 21 Faculty of Medicine and Biomedical Center in Pilsen, Charles University, Pilsen, Czech Republic; 22 University Hospitals Bristol and Weston NHS Foundation Trust and the University of Bristol National Institute for Health Research Bristol Biomedical Research Centre, University of Bristol, Bristol, UK; 23 Department of Epidemiology, Johns Hopkins Bloomberg School of Public Health, Baltimore, MD USA; 24 Sidney Kimmel Comprehensive Cancer Center at Johns Hopkins, Baltimore, MD USA; 25 Division of Public Health Sciences, Department of Surgery, Washington University in St. Louis, St. Louis, MO USA; 26 Alvin J. Siteman Cancer Center, Washington University School of Medicine, St. Louis, MO USA; 27 Division of Gastroenterology, Department of Medicine, Washington University School of Medicine, St. Louis, MO USA; 28 Department of Epidemiology and Biostatistics, School of Public Health, Imperial College London, London, UK

**Keywords:** Cancer, Genetics, Gastroenterology, Oncology, Risk factors

## Abstract

A potential association of endogenous circadian rhythm disruption with risk of cancer development has been suggested, however, epidemiological evidence for the association of sleep traits with colorectal cancer (CRC) is limited and often contradictory. Here we investigated whether genetically predicted chronotype, insomnia and sleep duration are associated with CRC risk in males, females and overall and according to CRC anatomical subsites using Mendelian randomization (MR). The two-sample inverse variance weighted (IVW) method was applied using summary-level data in up to 58,221 CRC cases and 67,694 controls and genome-wide association data of genetic variants for self-reported sleep traits. Secondary analyses using alternative instruments and sensitivity analyses assessing potential violations of MR assumptions were conducted. Genetically predicted morning preference was associated with 13% lower risk of CRC in men (OR_IVW_ = 0.87, 95% CI = 0.78, 0.97, *P* = 0.01), but not in women or in both sexes combined. Τhis association remained consistent in some, but not all, sensitivity analyses and was very similar for colon and rectal cancer. There was no evidence of an association for any other sleep trait. Overall, this study provides little to no evidence of an association between genetically predicted sleep traits and CRC risk.

## Introduction

Colorectal cancer (CRC) is the third most commonly diagnosed cancer and the second leading cause of cancer death worldwide, accounting for up to 10% of new cancer cases and 9% of cancer deaths in 2020^1^. Night shift work was classified as a probable risk factor (Group 2A carcinogen) to humans according to the 2019 report by the International Agency for Research on Cancer^2^. Beyond previously studied associations between night shift work and CRC, epidemiological evidence linking other metrics of circadian disruption with risk of CRC in the general population is emerging. A dose–response meta-analysis of six studies in the United States showed that long but not short sleep duration relative to 7–8 h of sleep was associated with a 21% increased risk of developing CRC^3^. In a more recent nested case–control study from Taiwan, participants (166 cases) with sleep disorders (having three or more outpatient diagnoses according to ICD-9) were found to have a 29% increased risk of developing CRC compared to those without^4^.

Genome-wide association studies (GWASs) have identified a large number of genetic variants robustly associated with sleep traits. According to recently published GWASs, 13.7% of chronotype (morning or evening preference) variance was explained by additive genetic variants^5^. Likewise, the heritability of insomnia was estimated at 16.7%^6^ and independently at 2.6%^7^ and of sleep duration at 9.8%^8^. Although data linking chronotype and insomnia with CRC risk are limited, Mendelian randomization (MR), a common approach to address inherent biases in observational studies and investigate potential causal associations provided that certain assumptions are met, has also been widely adopted to study the association of sleep-related traits with several outcomes, including breast^9,10^ and prostate cancer^10^, as well as known risk factors of CRC, such as adiposity^11^ and type 2 diabetes mellitus (T2DM)^12^. MR studies have reported that morning preference was associated with increased T2DM risk^13^, alcohol consumption^14^, educational attainment^15^, and reduced risks of breast^9,10^ and prostate cancer^10^; insomnia was associated with an increased T2DM risk, higher body mass index (BMI) and a decrease in educational attainment^7^; increased sleep duration was associated with an increased risk of breast cancer^9^ and a higher BMI in children^8^ and, lastly, short sleep duration was associated with an increased CRC risk using data from UK Biobank (UKB) based on 5,486 CRC cases^16^. To our knowledge, there is no other MR study that has investigated the potential association between sleep-related traits and CRC risk.

The aim of the current study was to assess whether genetically predicted chronotype, insomnia and sleep duration are associated with CRC risk in males, females and overall and according to CRC anatomical subsites using two-sample MR, leveraging data from the Genetics and Epidemiology of Colorectal Cancer Consortium (GECCO), the Colorectal Cancer Transdisciplinary Study (CORECT) and the Colon Cancer Family Registry (CCFR). We also investigated whether genetic variants associated with short sleep, long sleep and accelerometer-derived measures of sleep traits support the primary associations of self-reported sleep traits on CRC risk. We accounted for potential pleiotropy due to BMI, alcohol use, educational attainment, smoking and T2DM, phenotypes with a high frequency of associations with genome-wide significant sleep trait variants according to PhenoScanner database. Furthermore, we used genetic variants identified in sex-specific sleep trait GWASs to assess the extent to which findings differed by sex, as it has been suggested by observational findings^17–21^ on the effect of sleep traits on CRC outcomes and also by evidence from molecular studies reporting a differential timing of clock gene expression among men and women^22^.

## Methods

### Sleep trait GWASs

A GWAS Catalog search (https://www.ebi.ac.uk/gwas) was conducted in December 2020 for published GWASs on sleep traits. Genetic variants reported to be independently associated with self-reported chronotype^5^, insomnia symptoms^6,7^ and sleep duration^8^ at genome wide significance (*P* < 5 × 10^–8^) in participants of European ancestry were selected from the largest GWASs with adequate summary association data.

Self-reported chronotype was assessed at recruitment in UKB^23^ and 23andMe^24^ via a single question: “Do you consider yourself to be?” and “Are you naturally a night person or a morning person?”, respectively, and several possible answers, as described in more detail elsewhere^5^. To maximise statistical power, Jones et al*.* dichotomized chronotype into morning or evening preference and used results from both UKB and 23andMe in the GWAS meta-analysis (*n* = 697,828). Summary statistics were adjusted for age, sex, genotyping platform (UKB, 23andMe), study centre (UKB) and principal components (23andMe). Overall, 351 independent genetic variants at *P* < 5 × 10^–8^ were identified^5^.

Self-reported insomnia symptoms were assessed in UKB^23^ in response to the question: “Do you have trouble falling asleep at night, or do you wake up in the middle of the night?”, and similar questions in the Nord-Trøndelag Health Study (HUNT)^25^ and the Partners HealthCare Biobank (Partners Biobank)^26^. Lane et al*.* performed two GWAS meta-analyses for frequent insomnia symptoms (*n* = 254,767) and for any insomnia symptoms (*n* = 532,378). The models were adjusted for age, sex, genetic ancestry, and genotyping array^6^, and a total of 57 independent variants at *P* < 5 × 10^–8^ were identified. Summary statistics for insomnia complaints measured with similar questionnaires were also obtained from a large-scale GWAS meta-analysis of up to 1,331,010 European-ancestry individuals from UKB (*n* = 386,533) and 23andMe (*n* = 944,477) and overall 250 lead variants at *P* < 5 × 10^–8^ were identified^7^.

Self-reported sleep duration was assessed at recruitment in UKB^23^ with the question: “About how many hours sleep do you get in every 24 h? (please include naps)” and was treated as a continuous variable. Dashti et al*.* performed a GWAS among UKB participants (*n* = 446,118) and identified 78 independent variants after adjustment for age, sex, 10 principal components, genotyping array, and genetic correlation matrix. In addition, a fixed-effects meta-analysis of the UKB and the Cohorts for Heart and Aging Research in Genomics Epidemiology (CHARGE) (*n* = 493,298)^27^ identified 13 additional new variants. Overall, 91 independent variants at *P* < 5 × 10^–8^ emerged from this study^8^.

### Genetic variant selection

Out of the 351^5^ genetic variants for chronotype, 57^6^ and 250^7^ for insomnia symptoms and 91^8^ for continuous sleep duration, we excluded those variants which were not available in the CRC datasets, were palindromic with minor allele frequency ≥ 0.3, were in linkage disequilibrium (LD) (R^2^ ≥ 0.01) and had an F-statistic < 10^28^ to account for weak instrument bias based on the strength of their association with the sleep trait. The final number of variants used in the primary analysis were 290 for chronotype, 38 and 190 for insomnia and 76 for sleep duration, with 1% genetic variant overlap observed between chronotype and insomnia by Jansen et al*.* (2019), 2% between insomnia by Lane et al*.* (2019) and insomnia by Jansen et al*.* (2019), and no overlap in genetic variants between the rest of the exposures. Genetic variant selection is shown in Supplementary Figure S1.

### Colorectal cancer GWASs

The association of the genetic instruments for chronotype, insomnia and sleep duration with CRC risk was obtained from the GECCO, CORECT and CCRF consortia^29–31^. Further information on the characteristics of the contributing studies is provided in Supplementary Table S1. All CRC cases were defined as colorectal neoplasia and confirmed by pathological reports, medical records or death certificate information. In analysis of both sexes combined data on 58,221 CRC cases and 67,694 controls were available for 16,900,397 autosomal variants. GWAS results were also available by sex, with data on 59,889 females and 66,026 males overall, and by tumour anatomical subsite, with summary association data on 31,083 colon, 13,857 proximal colon, 15,306 distal colon and 15,775 rectal cancer cases.

### Statistical power

An online tool available at http://cnsgenomics.com/shiny/mRnd/ was employed to perform statistical power calculations^32^. For an odds ratio (OR) of 1.10 (or its reciprocal 0.91) per 1 standard deviation (SD) change in the exposure and a type 1 error of 5%, the statistical power (Supplementary Table S2) and the minimum detectable OR at 80% power were calculated (Supplementary Table S3).

### Primary MR analyses

Prior to MR analysis, it is essential to harmonize the data so that all variants are standardized to associate with the exposure in the same direction and that the effect and other alleles are aligned between the instrument-exposure (GX) and the instrument-outcome (GY) datasets ensuring that they are both coded from the same strand. Pearson’s correlation coefficient of the effect allele frequencies (EAFs) among datasets prior and post harmonization was estimated as a quality control measure of the harmonization process^33^ (Supplementary Tables S6, S15, S22, S43). Two-sample MR was conducted using summary association data from published GWASs to assess whether genetically predicted sleep traits (chronotype, insomnia and sleep duration) are associated with CRC risk overall and according to sex and anatomical subsites. Meta-analysis of the Wald estimates for each variant was performed by implementing an inverse-variance weighted (IVW) method. The fixed-effects IVW model assumes absence of horizontal pleiotropy and will return an unbiased estimate provided that all variants are valid instrumental variables (IVs)^34^. Correspondingly, the IVW random-effects model will return an unbiased estimate in the presence of balanced horizontal pleiotropy^35^.

### Sensitivity analyses for the assessment of MR assumptions

To evaluate instrument strength and potential violation of the first MR assumption, i.e., the genetic variants must be associated with the sleep trait of interest^36,37^, the F-statistic for each variant-trait association was calculated^38^. According to the second and third MR assumptions, the genetic variants should not be associated with any confounder of the sleep trait-CRC relationship and should not influence CRC via some other pathway than that of the sleep trait^36,37^. To account for potential horizontal pleiotropy, another five MR methods were performed, each of them providing a valid MR estimate under different combinations of assumptions. MR-Egger provides an unbiased causal effect estimate even if the third MR assumption is violated and all the variants are invalid IVs, provided that the InSIDE assumption of independency between the horizontal pleiotropic effects and the variants-exposure effects is met^39^. Instrument strength and variability of the genetic instrument-exposure association due to potential violation of the NO Measurement Error (NOME) assumption were also inspected via the I^2^_GX_ statistic^40^. The weighted median returns a valid estimate when at least 50% of the weight of the genetic variants comes from valid IVs^41^. The weighted mode is valid under the Zero Modal Pleiotropy Assumption (ZEMPA) according to which out of all clusters of variants with similar effects, the largest is the group of valid IVs^42^. The contamination mixture is a likelihood-based method that identifies groups of variants with similar estimates, an indicator of the existence of distinct mechanisms in the exposure-outcome relationship, and provides robust estimates under the ZEMPA assumption^43^. To detect potential outlying genetic variants, we implemented the MR pleiotropy residual sum and outlier test (MR-PRESSO), a method that identifies and excludes outliers, applying a random-effects IVW model^44^. The Cochran’s Q statistic for heterogeneity was also computed^34^. To further graphically assess the presence of pleiotropy, we generated diagnostic scatter, funnel and forest plots. To explore potential associations between the genetic instruments with secondary phenotypic traits that could indicate possible violations in the second or third MR assumptions, we queried the PhenoScanner database (http://www.phenoscanner.medschl.cam.ac.uk/) using a *P* threshold of 1 × 10^–5^ and tabulated the resulting associations per variant. All analyses were conducted in the statistical software R version 4.2.0 using the packages MendelianRandomization^45^ and MRCIEU/TwoSampleMR^36^.

### Secondary analyses using alternative exposures/instruments

To account for potential non-linear associations of sleep duration with CRC risk, we performed two-sample MR using 27 genome-wide significant variants for short sleep (< 7 h vs. 7–8 h; *n* = 106,192 cases and 305,742 controls) and 8 variants for long sleep (> 8 h vs. 7–8 h; *n* = 34,184 cases and 305,742 controls) in participants of European ancestry from UKB^8^.

Given that all sleep traits were based on self-reported information, we assessed the robustness of our findings using accelerometer-derived equivalents of sleep measures in up to 85,670 participants from UKB. We used genetic variants for seven accelerometer-derived sleep traits representative of chronotype [L5 timing defined as the midpoint (number of hours since the previous midnight) of the least active five hours of each day, reflects whether a person goes to bed earlier or later in the day (6 variants); M10 timing defined as the midpoint of the most-active 10 h, reflects whether a person is most active earlier or later in the day (1 variant); sleep midpoint (1 variant)], insomnia [number of sleep episodes (21 variants)] and sleep duration [actigraphy nocturnal sleep duration (11 variants); sleep efficiency (5 variants); diurnal inactivity (2 variants)] as described elsewhere^46^. The genetic correlation of self-reported and accelerometer-derived measures has been investigated in a previous study, according to which the correlation coefficient is equal to 0.903 between chronotype and L5 timing, 0.095 between any insomnia and the number of sleep episodes, and 0.430 between sleep duration and actigraphy nocturnal sleep duration^9^. The observed overlap in genetic variants was approximately 1% between L5 timing and chronotype, and 1%, 0.5%, 1% and 0.5% between insomnia by Jansen et al*.* (2019) and L5 timing, number of sleep episodes, actigraphy nocturnal sleep duration and sleep efficiency, respectively. There was no overlap between the rest of accelerometer-derived measures and any self-reported sleep trait.

Traits with a high frequency of associations with genome-wide significant variants for sleep traits using the PhenoScanner database, i.e., BMI, alcohol use, educational attainment, lifetime smoking and T2DM were followed up in Multivariable Mendelian randomization (MVMR) analysis to assess direct effects of sleep traits independent of potential confounders and to evaluate potential pleiotropy arising from those factors^47^. In order for the MVMR estimates to be valid, the genetic variants should be robustly associated with at least one of the exposures in the model (relevance), be independent of all confounders of all the exposure-outcome associations (exchangeability) and be independent of the outcome conditional on the exposures and confounders (exclusion restriction). Details on the GWASs used in MVMR analysis are available in Supplementary Table S28 and summary association data for the corresponding genetic instruments are included in Supplementary Tables S29-S33. To test for instrument strength in the MVMR setting, the conditional F-statistic was calculated with a threshold of less than 10 suggesting probable weak instrument bias^48^. To further test instrument strength, a modified version of the Cochran’s Q statistic was calculated under the null hypothesis that IVs do not sufficiently account for any of the variation in the exposures. Also, to test for pleiotropy, an adjusted version of the Cochran’s Q statistic (Q_A_), under the null hypothesis of no heterogeneity in the genetic variants (Supplementary Table S34), as well as the MVMR MR-Egger regression^49^ (Supplementary Table S35) were used. MVMR analysis was conducted using the R packages MendelianRandomization^45^ and MRPracticals^49^, R version 4.2.0. MVMR findings were followed up in a bi-directional MR framework to decipher the direction of the association of sleep traits with factors of interest from the MVMR analyses (Supplementary Tables S36-S41).

The genetic instruments used in all previous primary and secondary analyses were identified in the corresponding GWAS meta-analyses for chronotype, insomnia and sleep duration, in which results for females and males were combined. We also carried out MR analyses using instruments identified in sex-specific GWASs from UKB. Following LD-clumping, 32 variants were associated at *P* < 5 × 10^–8^ with chronotype in males, 65 with chronotype in females, 10 with frequent insomnia symptoms in males, 15 with frequent insomnia symptoms in females, 7 with sleep duration in males, and 16 with sleep duration in females. After relaxing the threshold at *P* < 5 × 10^–6^ in supplementary analysis to increase the number of instruments, 199, 126, 90, 58, 80 and 72 variants were identified, respectively.

## Results

**Fig. 1:**
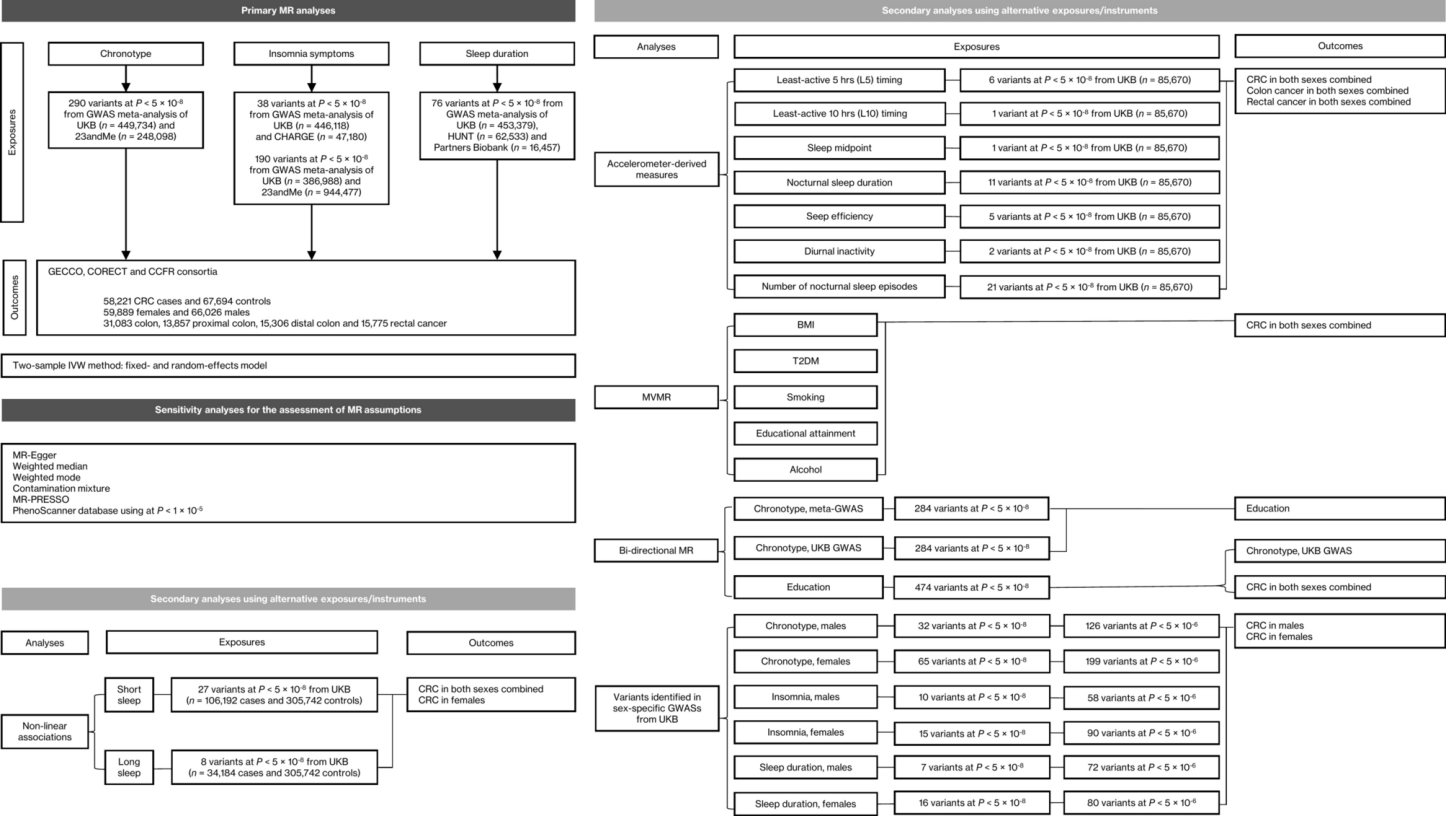
Outline of the study design and data sources for the primary and secondary analyses performed. BMI: body mass index; CCFR: Colon Cancer Family Registry; CHARGE: Cohorts for Heart and Aging Research in Genomics Epidemiology; CORECT: Colorectal Cancer Transdisciplinary Study; CRC: colorectal cancer; GECCO: Genetics and Epidemiology of Colorectal Cancer Consortium; GWAS: Genome-wide association study; HUNT: Nord-Trøndelag Health Study; L5: midpoint of the least active five hours of each day; M10: midpoint of the most-active 10 h; MR: Mendelian randomization; MR-PRESSO: MR pleiotropy residual sum and outlier test; MVMR: Multivariable Mendelian randomization; Partners Biobank: Partners HealthCare Biobank; T2DM: type 2 diabetes mellitus; UKB: UK Biobank.

**Fig. 2:**
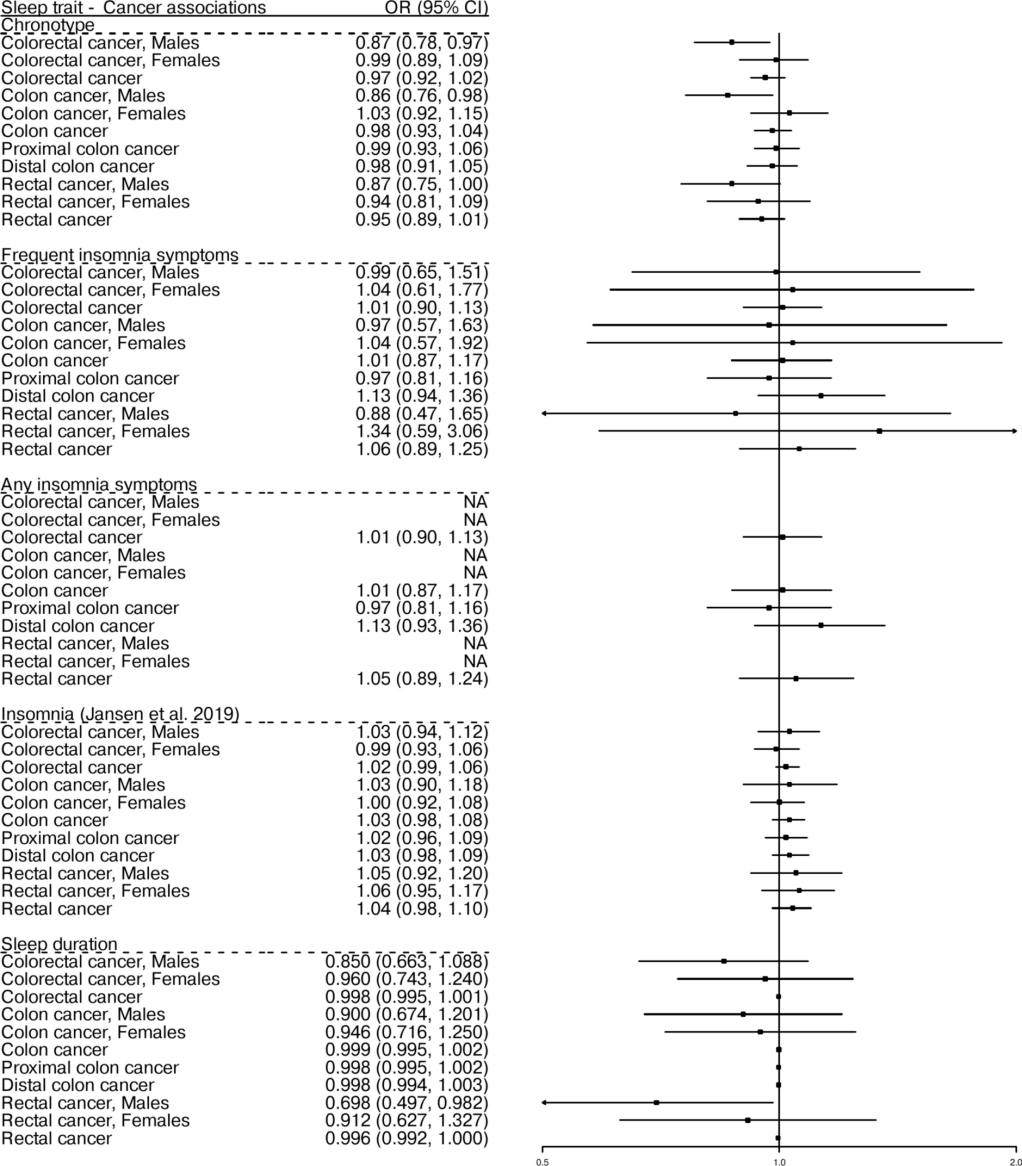
Mendelian randomization (MR) estimates for the association of sleep traits with colorectal cancer outcomes in primary MR analyses. Forest plot of two-sample MR random-effects inverse-variance weighted estimates for the association between sleep traits and risk of colorectal, colon, proximal colon, distal colon, and rectal cancer in both sexes combined and in sex-stratified analyses. CI: confidence interval; NA: Non Applicable; OR: odds ratio.

An outline of our study design is presented in Figure 1. Associations of the genetic instruments with the exposures/outcomes are shown in Supplementary Tables S4, S5 (primary analyses) and Supplementary Tables S13, S14, S21, S29-S33, S36, S42 (secondary analyses). Figure 2 shows all primary MR associations. Table 1 depicts all primary IVW MR associations and two sensitivity analyses for the assessment of MR assumptions (Weighted median, MR-Egger). The random-effects IVW estimate is reported throughout the manuscript (OR_IVW_), unless otherwise specified where there is under-dispersion in the association estimates (OR_IVW-fixed_). All two-sample MR and corresponding sensitivity analyses are detailed in Supplementary Tables S7-S12 (primary analyses) and Supplementary Tables S16-S20, S23-S27, S34, S35, S37-S41, S44-S48 (secondary analyses). Diagnostic scatter, funnel and forest plots for the primary analyses are shown in supplementary figures (Supplementary Fig. S2-S148).

**Table 1:** Two-sample Mendelian randomization estimates for the association between sleep traits and risk of colorectal, colon, proximal colon, distal colon, and rectal cancer in both sexes combined and in sex-stratified analyses.

Exposure		Chronotype			Frequent insomnia symptoms			Any insomnia symptoms			Insomnia—Jansen et al*.* (2019)			Sleep duration		
**Outcome**	**Method**	**GVs**	**OR (95% CI)**	***P***	**GVs**	**OR (95% CI)**	***P***	**GVs**	**OR (95% CI)**	***P***	**GVs**	**OR (95% CI)**	***P***	**GVs**	**OR (95% CI)**	***P***
**Colorectal cancer, Males**	IVW random-effects model	289	0.87 (0.78, 0.97)	**0.01**	38	0.99 (0.65, 1.51)	0.96	NA	NA	NA	190	1.03 (0.94, 1.12)	0.53	76	0.850 (0.663, 1.088)	0.20
	Weighted median method		0.91 (0.79, 1.05)	0.20		1.04 (0.58, 1.89)	0.89		NA	NA		0.94 (0.83, 1.07)	0.37		1.003 (0.728, 1.381)	0.99
	MR-Egger method		0.92 (0.69, 1.23)	0.56		0.34 (0.07, 1.66)	0.18		NA	NA		0.81 (0.61, 1.07)	0.14		0.760 (0.336, 1.721)	0.51
**Colorectal cancer, Females**	IVW random-effects model	290	0.99 (0.89, 1.09)	0.78	38	1.04 (0.61, 1.77)	0.89	NA	NA	NA	190	0.99 (0.93, 1.06)	0.85	76	0.960 (0.743, 1.240)	0.75
	Weighted median method		0.95 (0.82, 1.09)	0.44		0.71 (0.36, 1.37)	0.31		NA	NA		0.94 (0.86, 1.04)	0.24		1.246 (0.915, 1.697)	0.16
	MR-Egger method		0.77 (0.60, 0.99)	**0.04**		0.34 (0.05, 2.24)	0.26		NA	NA		1.12 (0.86, 1.47)	0.40		0.842 (0.359, 1.973)	0.69
**Colorectal cancer**	IVW random-effects model	290	0.97 (0.92, 1.02)	0.19	38	1.01 (0.90, 1.13)	0.87	38	1.01 (0.90, 1.13)	0.88	190	1.02 (0.99, 1.06)	0.25	76	0.998 (0.995, 1.001)	0.27
	Weighted median method		0.96 (0.90, 1.02)	0.16		1.02 (0.87, 1.19)	0.85		1.01 (0.86, 1.19)	0.89		1.02 (0.97, 1.08)	0.38		0.998 (0.994, 1.002)	0.29
	MR-Egger method		0.95 (0.83, 1.08)	0.42		0.81 (0.54, 1.20)	0.29		0.81 (0.54, 1.20)	0.29		1.13 (0.98, 1.31)	0.09		0.995 (0.983, 1.007)	0.41
**Colon cancer, Males**	IVW random-effects model	289	0.86 (0.76, 0.98)	**0.02**	38	0.97 (0.57, 1.63)	0.90	NA	NA	NA	190	1.03 (0.90, 1.18)	0.63	76	0.900 (0.674, 1.201)	0.47
	Weighted median method		0.95 (0.80, 1.14)	0.61		1.01 (0.48, 2.14)	0.98		NA	NA		0.93 (0.79, 1.08)	0.33		1.133 (0.757, 1.695)	0.54
	MR-Egger method		0.97 (0.69, 1.37)	0.87		0.41 (0.06, 2.91)	0.37		NA	NA		0.75 (0.49, 1.13)	0.17		0.872 (0.336, 2.260)	0.78
**Colon cancer, Females**	IVW random-effects model	290	1.03 (0.92, 1.15)	0.62	38	1.04 (0.57, 1.92)	0.89	NA	NA	NA	190	1.00 (0.92, 1.08)	0.93	76	0.946 (0.716, 1.250)	0.70
	Weighted median method		1.07 (0.91, 1.26)	0.43		1.23 (0.57, 2.65)	0.61		NA	NA		0.94 (0.84, 1.05)	0.31		0.972 (0.680, 1.388)	0.88
	MR-Egger method		0.80 (0.60, 1.08)	0.15		0.32 (0.04, 2.75)	0.30		NA	NA		1.08 (0.78, 1.49)	0.63		0.691 (0.276, 1.731)	0.43
**Colon cancer**	IVW random-effects model	290	0.98 (0.93, 1.04)	0.50	38	1.01 (0.87, 1.17)	0.92	38	1.01 (0.87, 1.17)	0.93	190	1.03 (0.98, 1.08)	0.24	76	0.999 (0.995, 1.002)	0.45
	Weighted median method		1.00 (0.93, 1.08)	0.91		1.07 (0.88, 1.29)	0.51		1.06 (0.87, 1.28)	0.58		1.04 (0.98, 1.11)	0.18		1.001 (0.996, 1.005)	0.82
	MR-Egger method		0.99 (0.85, 1.15)	0.91		0.75 (0.45, 1.25)	0.27		0.75 (0.45, 1.23)	0.26		1.11 (0.93, 1.34)	0.25		0.993 (0.980, 1.006)	0.28
**Proximal colon cancer**	IVW random-effects model	290	0.99 (0.93, 1.06)	0.82	38	0.97 (0.81, 1.16)	0.77	38	0.97 (0.81, 1.16)	0.75	190	1.02 (0.96, 1.09)	0.50	76	0.998 (0.995, 1.002)	0.34
	Weighted median method		1.01 (0.92, 1.11)	0.76		1.02 (0.80, 1.30)	0.87		1.01 (0.80, 1.29)	0.92		1.06 (0.98, 1.15)	0.13		0.999 (0.993, 1.004)	0.63
	MR-Egger method		1.01 (0.83, 1.21)	0.95		0.46 (0.26, 0.84)	**0.01**		0.47 (0.26, 0.84)	**0.01**		1.06 (0.83, 1.35)	0.63		0.993 (0.979, 1.007)	0.30
**Distal colon cancer**	IVW random-effects model	290	0.98 (0.91, 1.05)	0.60	38	1.13 (0.94, 1.36)	0.20	38	1.13 (0.93, 1.36)	0.21	190	1.03 (0.98, 1.09)	0.27	76	0.998 (0.994, 1.003)	0.50
	Weighted median method		1.00 (0.91, 1.10)	0.98		1.30 (1.01, 1.66)	**0.04**		1.26 (0.98, 1.62)	0.07		1.04 (0.96, 1.13)	0.35		0.998 (0.993, 1.004)	0.58
	MR-Egger method		0.97 (0.80, 1.19)	0.79		1.35 (0.70, 2.63)	0.37		1.29 (0.67, 2.49)	0.45		1.12 (0.90, 1.39)	0.32		0.992 (0.975, 1.008)	0.32
**Rectal cancer, Males**	IVW random-effects model	289	0.87 (0.75, 1.00)	0.06	38	0.88 (0.47, 1.65)	0.70	NA	NA	NA	190	1.05 (0.92, 1.20)	0.44	76	0.698 (0.497, 0.982)	**0.04**
	Weighted median method		0.84 (0.68, 1.04)	0.12		0.94 (0.39, 2.26)	0.89		NA	NA		0.99 (0.83, 1.18)	0.90		0.799 (0.497, 1.284)	0.35
	MR-Egger method		0.81 (0.55, 1.20)	0.30		1.05 (0.10, 11.03)	0.97		NA	NA		0.96 (0.63, 1.47)	0.87		0.895 (0.291, 2.759)	0.85
**Rectal cancer, Females**	IVW random-effects model	290	0.94 (0.81, 1.09)	0.43	38	1.34 (0.59, 3.06)	0.48	NA	NA	NA	190	1.06 (0.95, 1.17)	0.31	76	0.912 (0.627, 1.327)	0.63
	Weighted median method		0.87 (0.70, 1.10)	0.24		1.07 (0.34, 3.32)	0.91		NA	NA		1.08 (0.92, 1.26)	0.33		0.884 (0.528, 1.480)	0.64
	MR-Egger method		0.91 (0.62, 1.35)	0.64		0.64 (0.03, 12.25)	0.77		NA	NA		1.69 (1.09, 2.61)	**0.02**		1.099 (0.317, 3.814)	0.88
**Rectal cancer**	IVW random-effects model	290	0.95 (0.89, 1.01)	0.13	38	1.06 (0.89, 1.25)	0.52	38	1.05 (0.89, 1.24)	0.58	190	1.04 (0.98, 1.10)	0.18	76	0.996 (0.992, 1.000)	0.06
	Weighted median method		0.98 (0.89, 1.08)	0.72		1.19 (0.93, 1.51)	0.16		1.18 (0.92, 1.50)	0.18		1.03 (0.95, 1.11)	0.45		0.996 (0.991, 1.002)	0.20
	MR-Egger method		0.93 (0.77, 1.12)	0.43		1.40 (0.78, 2.52)	0.26		1.27 (0.71, 2.27)	0.42		1.23 (0.99, 1.52)	0.06		0.999 (0.983, 1.015)	0.89

CI: Confidence Interval; GVs: Genetic Variants; IVW: Inverse-Variance Weighted; MR: Mendelian Randomization; NA: Non Applicable; OR: Odds Ratio

### Primary MR analyses of sleep traits and CRC and assessment of MR assumptions

The F-statistics for the eligible genetic variants did not indicate the presence of weak instruments (F-statistic range = 30–430 for chronotype, 16–217 for insomnia, 30–221 for sleep duration) (Supplementary Table S4).

Two-sample MR suggested a 13% lower risk of CRC in men with morning preference in comparison with evening preference chronotype [OR_IVW_ = 0.87, 95% confidence interval (CI) = 0.78, 0.97, *P* = 0.01]. This finding was consistent in site-specific analysis of colon cancer (OR_IVW_ = 0.86, 95% CI = 0.76, 0.98, *P* = 0.02) and rectal cancer (OR_IVW-fixed_ = 0.87, 95% CI = 0.75, 1.00, *P* = 0.04) in males. However, there was no evidence of an association for chronotype with CRC risk in women (OR_IVW_ = 0.99, 95% CI = 0.89, 1.09, *P* = 0.78), with an 83.2% of the variation in the effect estimates between men and women being attributed to heterogeneity rather than chance (Cochrane’s Q = 5.97, Cochrane’s Q test *P* = 0.02). Similarly, there was no evidence of an association in both sexes combined (OR_IVW_ = 0.97, 95% CI = 0.92, 1.02, *P* = 0.19) or in site-specific analyses (Table 1, Supplementary Table S7 and Fig. 2). There was strong evidence for heterogeneity that cannot be explained by sampling variation alone among the causal estimates, suggesting potential violation of the MR assumptions (Supplementary Table S9; smallest Cochran’s Q test *P* = 6.80 × 10^–11^). Some indication of horizontal pleiotropy based on the MR-Egger intercept test was evident in the association of morning preference with CRC in females (intercept *P* = 0.04, OR_MR-Egger_ = 0.77, 95% CI = 0.60, 0.99, *P* = 0.04) (Table 1, Supplementary Tables S7, S8), however, the association was not supported by any other primary or sensitivity analyses. Results were consistent in terms of direction in most of the applied sensitivity analyses (Table 1, Supplementary Table S7), although the association of chronotype with CRC, colon and rectal cancer in males attenuated in the weighted median analysis. The MR-PRESSO analysis identified two outliers for chronotype (i.e., rs45597035, rs6007594) (Supplementary Table S10). A PhenoScanner search revealed that the variants used as genetic instruments for MR analyses (Supplementary Table S12) and the outlying genetic instruments from MR-PRESSO (Supplementary Table S11) were associated at *P* < 5 × 10^–5^ with several secondary phenotypic traits relevant to CRC. However, after removal of the outlying variants, the IVW effect estimates for the association of chronotype with CRC (MR-PRESSO OR_IVW_ = 0.89, 95% CI = 0.78, 0.99, *P* = 0.02) and with colon cancer in males (MR-PRESSO OR_IVW_ = 0.87, 95% CI = 0.74, 1.00, *P* = 0.03) remained largely unchanged (Supplementary Table S10).

There was no evidence of an association for genetically predicted frequent/any insomnia symptoms with CRC or its subsites in both sexes combined and in sex-stratified analyses (Table 1, Supplementary Table S7 and Fig. 2). The results were largely consistent in sensitivity analyses (Table 1, Supplementary Tables S7-S10). Some indication of horizontal pleiotropy based on the MR-Egger intercept test was evident in the association of insomnia with proximal cancer in both sexes combined (intercept *P* = 0.01, OR_MR-Egger_ = 0.46, 95% CI = 0.26, 0.84, *P* = 0.01) and with rectal cancer in females (intercept *P* = 0.03, OR_MR-Egger_ = 1.69, 95% CI = 1.09, 2.61, *P* = 0.02) (Table 1, Supplementary Tables S7, S8), however, these associations were not consistent among other sensitivity analysis methods.

Two-sample IVW MR analysis found some evidence of an association of sleep duration with rectal cancer in males (OR_IVW_ = 0.70, 95% CI = 0.50, 0.98 per hour increase, *P* = 0.04), however, this finding was not supported by sensitivity analyses. There was no evidence of an association for genetically predicted sleep duration with CRC or its subsites in both sexes combined and in other sex-stratified analyses (Table 1, Supplementary Table S7 and Fig. 2).

### Secondary analyses

Two-sample MR showed no clear evidence that either short (OR_IVW_ = 1.02, 95% CI = 0.75, 1.38 per doubling of genetic liability for short sleep duration, *P* = 0.91) or long sleep duration (OR_IVW-fixed_ = 1.03, 95% CI = 0.91, 1.17 per doubling of genetic liability for long sleep duration, *P* = 0.62) compared to 7–8 h of sleep were associated with CRC in both sexes combined (F-statistic range = 16–57 for short sleep duration, 16–46 for long sleep duration). Likewise, there was no evidence of an association for either short or long sleep duration and CRC in females (Supplementary Table S16). Corresponding data in males were not available. The results were consistent in sensitivity analyses (Supplementary Tables S16-S20).

In analyses using variants associated with accelerometer-derived equivalents of sleep measures (F-statistic range = 30–155), there was no evidence of an association for L5 timing (OR_IVW-fixed_ = 1.04, 95% CI = 0.86, 1.27 per hour elapsed since previous midpoint, *P* = 0.67), M10 timing (OR_IVW-fixed_ = 1.23, 95% CI = 0.68, 2.23 per hour elapsed since previous midpoint, *P* = 0.49), sleep midpoint (Wald ratio estimator = 1.39, 95% CI = 0.70, 2.76 per hour increase, *P* = 0.34), actigraphy nocturnal sleep duration (OR_IVW-fixed_ = 0.95, 95% CI = 0.81, 1.12 per hour increase, *P* = 0.53), sleep efficiency (OR_IVW-fixed_ = 0.97, 95% CI = 0.76, 1.25 per 1% increase, *P* = 0.83), diurnal inactivity (Wald ratio estimator = 1.32, 95% CI = 0.89, 1.97 per hour increase, *P* = 0.17) and number of sleep episodes (OR_IVW-fixed_ = 0.96, 95% CI = 0.84, 1.09 per sleep episode, *P* = 0.51) and CRC using both the IVW method and in sensitivity analyses. We further assessed the association of accelerometer-derived measures with colon and rectal cancer, but the results did not change (Supplementary Tables S23-S27). Although the findings for L5, M10 and sleep midpoint have wide CIs, they are consistent with the association of evening preference with an increased CRC risk.

Results from the MVMR analysis on CRC in both sexes combined were largely unchanged in terms of direction and increased in magnitude when accounting for BMI, alcohol, smoking or T2DM compared to the unadjusted association estimates. After adjusting for educational attainment, a direct effect of chronotype on CRC was observed (OR_educational attainment-adj_ = 0.80, 95% CI = 0.65, 0.98, *P* = 0.03) (Supplementary Table S34). In bi-directional MR analysis, a SD increase in educational attainment was associated with a decrease in morning preference chronotype and CRC risk (OR_IVW_ = 0.98, 95% CI = 0.97, 1.00, *P* = 0.01 and OR_IVW_ = 0.75, 95% CI = 0.69, 0.82, *P* = 3.88 × 10^–10^, respectively), and morning compared to evening preference was associated with a decrease in years of education by 0.98 SD (Supplementary Table S37). The bi-directional association between chronotype and educational attainment supports that morning preference is associated with a lower educational attainment and vice versa, however, it does not preclude a potential role of educational attainment as a confounder in the association of chronotype with CRC.

Finally, the association of morning preference with a decrease in CRC risk in males was consistent (but with wider CIs) in MR analyses using instruments identified in sex-specific GWASs from UKB (Supplementary Table S44).

## Discussion

In the current study, we employed genetic variants as IVs in a two-sample MR framework to investigate potential associations of three modifiable sleep traits, including chronotype, the endogenous circadian rhythm, insomnia symptoms and sleep duration with CRC risk. Morning compared with an evening preference was suggestively associated with lower CRC and colon cancer risk among men. This finding was consistent in some, but not all, sensitivity analysis methods. Site-specific analyses and secondary analyses using alternative exposures/instruments predominantly supported this association, although with wider CIs. There was no evidence of an association between chronotype and CRC risk in women or in both sexes combined. There was also no evidence of an association between insomnia and sleep duration and CRC or its subsites.

The epidemiological evidence for an association between sleep traits and CRC risk is limited and, in most cases, inconsistent. The suggestive association of morning preference with a decreased risk of CRC and colon cancer in men is supported by previous findings from a population-based case–control study, according to which colon cancer risk was elevated among men who ever worked at night compared to men who never worked at night^17^, and a cohort of Korean male professional emergency responders (ERs), whose standardized incidence ratios showed an increase with reference to the general population^18^. The adverse effect of light-at-night exposure, inherent to night-shift work, on carcinogenesis is aligned with animal study observations^50^. In addition, the lack of an association between chronotype and CRC risk in women is in accordance with an earlier female cohort of radio and telegraph operators working evening or night shifts^19^, and with two female cohorts of nurses working rotating night shifts, the Nurses´ Health Study (NHS)^20^ and the NHS2^20,21^. In a previous NHS analysis, a positive association between night shift work for over 15 years and CRC risk was documented^51^; however, in a most recent analysis of the NHS cohort by Papantoniou et al*.* (2018), which expanded both the follow-up time and sample size, the former relationship was attenuated^20^. It is worth noting that circadian misalignment and its potential adverse effects caused by night shift work might vary depending on diurnal preference, with Vetter et al*.* (2015) reporting an increased risk in T2DM in women with timing discrepancy between their chronotype and work schedule^52^. Moreover, according to the Bureau of Labor Statistics of the U.S. Department of Labor, 15% of full-time wage and salary employees worked on alternative shifts in 2004, but only 11.5% of them worked a non-day schedule out of personal preference^53^. In this context and given the shortage of epidemiological evidence evaluating the effect of chronotype per se on carcinogenesis and the insufficient evaluation in existing epidemiological studies of the participants’ socioeconomic status impact on the potential interconnection between shift work and colorectal malignancies, shift work should be used with prudence as a proxy for chronotype for lack of a more suitable measure, considering also that chronotype could potentially be acting as an effect modifier for shift work i.e., the magnitude of the effect of shift work on cancer risk might differ depending on an individual’s chronotype. The speculated difference in the effect of chronotype by sex is augmented by molecular studies on clock gene expression, according to which daily rhythms of *PER2*, *PER3* and *ARNTL1* genes are delayed in men compared to women^22^ as well as by evidence supporting an earlier pineal melatonin secretion in women compared to men relative to their sleep time^54^. Thus, the observed association of morning preference with CRC and colon cancer risk in men could potentially be attributed to inherent sex differences and not to sample variation alone.

We observed a lack of an association between insomnia and risk of CRC in the current study using both self- and accelerometer-derived measures of insomnia. The existing literature is limited to examining insomnia symptoms as a disease aftermath in cancer patients or survivors and is accordingly short on exposure data prior to the onset of the malignancy. Therefore, there is a need for the accumulation of exposure data long before cancer diagnosis to assess whether insomnia is a potential risk factor for CRC in an observational setting.

No evidence of an association between sleep duration and CRC risk was observed both in this comprehensive MR study and in the literature. Specifically, two cohorts in women^21,55^, one cohort in both sexes combined^56^, and an MR study using data from the UKB^16^ reported no evidence of an association between sleep duration and CRC. Our secondary analyses using accelerometer-derived measures for sleep duration showed no evidence for an association of sleep duration equivalents, with CRC, colon and rectal cancer and our analysis assessing potential non-linear effects of sleep duration also indicated that neither short nor long sleep duration associated with CRC risk relative to 7–8 h of sleep. These findings partly support the results from a recent MR study of 367,586 UKB participants reporting no evidence of an effect for long sleep (*P* = 0.66) and 48% higher odds of CRC (*P* = 0.01) for short sleep duration. However, this study included only 5,486 CRC cases from UKB and its results were not supported in replication analysis using data from the FinnGen consortium^16^. In contrast to our findings, some observational studies reported an adverse effect of sleep duration. Among those, three are case–control studies^57–59^, prone to recall bias and further limitations due to their design, and two are cohorts but with data on specific population groups (postmenopausal women receiving hormone replacement therapy and overweight individuals who snorted) and therefore ambiguous in terms of generalizability^60,61^. Moreover, according to a previous study, there is a moderate correlation between self-reported and measured sleep duration^62^, introducing potential information bias in the arising role of sleep duration as a potential risk factor for CRC. Consequently, the observed variation in the literature could be partially attributed to the potentially high levels of participants’ misclassification, and hence any future studies should focus on the accurate characterization of sleep traits.

To our knowledge, this study constitutes the first comprehensive MR investigation of the association of five self-reported sleep traits and seven accelerometer-derived equivalents with the risk of CRC and its subsites in both sex-combined and in sex-stratified analyses. Among its principal strengths is the implementation of MR, a methodology which counteracts by design some of the main limitations inherent to observational studies, namely exposure measurement error, reverse causation, and residual confounding, provided that certain assumptions are met^36,37^. Although it is not possible to eliminate potential violations of the MR assumptions, we used several sensitivity analyses, with differences in statistical power and assumptions to be satisfied, to address this issue, and the interpretation of our findings was based on a thorough co-assessment of these methods. To account for potential heterogeneity, we implemented additional stratified analyses by sex and CRC anatomical subsites. We further conducted analyses to explore potential pleiotropic effects and detect outlying variants using MR-PRESSO. MVMR was implemented to assess the direct effects of sleep traits on CRC and to account for potential horizontal pleiotropy due to suspected confounders arising from our PhenoScanner search. Another key strength is the use of a large number of genetic variants robustly associated with sleep traits and identified in meta-analysis GWASs of well-established epidemiological cohorts with the largest available sample size to date, as well as the inclusion of colorectal cancer data from the GECCO, CORECT and CCFR consortia. In the case of insomnia, two meta-analytic summary association datasets were used to account for winner’s curse, i.e., the effect sizes for the genetic variants being larger in the discovery sample compared to the general population, the published GWAS by Jansen et al*.* (2019)^7^ and the second largest available dataset by Lane et al*.* (2019)^6^. However, there is an overlap of UKB participants in these datasets. As genetic variants for all analyses were identified in GWASs of both sexes combined, we also examined whether our findings were supported by genetic variants identified in sex-specific sleep trait GWASs. To assist the reproducibility of results and the evaluation of the harmonization process, all post harmonization datasets are available as supplementary material.

One potential limitation of the current study is the use of self-reported exposure data in the conducted GWASs. To mitigate this source of bias, we conducted secondary analyses using accelerometer-derived equivalents to the self-reported traits as more objective measures. However, given the limited number of variants associated with accelerometer-derived sleep traits, the smaller datasets and the lack of sex-specific data, the resulting effect estimates were of low precision. In addition, the use of summary data poses a restriction to the examination of potential non-linear effects and the stratification by other potential risk factors of the exposure-outcome association. However, we did try to account for potential deviation from linearity using instruments robustly associated with short and long sleep duration. Also, the use of smaller datasets to assess the site-specific effects of sleep traits might be limiting the precision of the corresponding analyses. Our study was also restricted to participants of European ancestry, so further studies are required to explore the associations of sleep traits in non-European ancestry groups. Another limitation related to the selection of participants is a small sample overlap between the exposure and outcome datasets. However according to simulation studies this bias should be considered negligible especially in the case of no weak genetic instruments^63^. It should be noted that although we did not explicitly correct for multiple testing, we interpreted our findings in a conservative way to account for the large number of exposures and outcomes evaluated in this study. Furthermore, results from MVMR and bi-directional analyses suggested a potential role of educational attainment in the chronotype-CRC association, which remains to be investigated in future studies.

This study provides little to no evidence of an association between genetically predicted sleep traits and CRC risk. The suggestive association observed between genetically predicted chronotype and CRC in men was consistent in terms of direction in some sensitivity and secondary analyses, however, due to their lack in precision and the aforementioned limitations of our study, any observations should be interpreted tentatively. Considering the ever-increasing circadian rhythm dysregulation imposed by modern lifestyle and the emerging evidence supporting its role in carcinogenesis, it is important to expand upon our findings and investigate potential underlying biological mechanisms that might inform on future interventions for CRC prevention.

## Supplementary Information

Supplementary Information 1.

Supplementary Information 2.

Supplementary Information 3.

Supplementary Information 4.

Supplementary Information 5.

Supplementary Information 6.

Supplementary Information 7.

## Data Availability

All data generated or analysed during this study are included in this published article (and its supplementary information files). Further information is available available from the corresponding author on reasonable request.
