# Supplementary material for: Mendelian randomization study of sleep traits and risk of colorectal cancer
Source: Sci Rep. 2025 Apr 18;15:13478. doi: 10.1038/s41598-024-83693-w (PMC12008275; doi:10.1038/s41598-024-83693-w)
Supplement: Supplementary file 1 — Supplementary Information 1. [file 41598_2024_83693_MOESM1_ESM.pdf]

Mendelian randomization study of sleep traits and risk of colorectal cancer

Olympia Dimopoulou*[1,2], Harriett Fuller[3], Rebecca C Richmond[1,2], Emmanouil Bouras[4], Bryony Hayes[1,2], Niki Dimou[5], Neil Murphy[5], Hermann Brenner[6-8], Andrea Gsur[9], Loic Le Marchand[10], Victor Moreno[11-14], Rish K Pai[15], Amanda I Phipps[16,17], Caroline Y Um[18], Franzel JB van Duijnhoven[19], Pavel Vodicka[20-22], Richard M Martin[1,2,23], Elizabeth A Platz[24,25], Marc J Gunter[5], Ulrike Peters[16,17], Sarah J Lewis[1], Yin Cao[26-28], Konstantinos K Tsilidis[3,4]

Supplementary figures

Supplementary Figure S1. Flow chart of genetic variant selection

Primary MR analyses: Chronotype

Supplementary Figure S2. Scatter plot of chronotype and colorectal cancer association in males
Supplementary Figure S3. Scatter plot of chronotype and colorectal cancer association in females
Supplementary Figure S4. Scatter plot of chronotype and colorectal cancer association
Supplementary Figure S5. Scatter plot of chronotype and colon cancer association in males
Supplementary Figure S6. Scatter plot of chronotype and colon cancer association in females
Supplementary Figure S7. Scatter plot of chronotype and colon cancer association
Supplementary Figure S8. Scatter plot of chronotype and proximal colon cancer association
Supplementary Figure S9. Scatter plot of chronotype and distal colon cancer association
Supplementary Figure S10. Scatter plot of chronotype and rectal cancer association in males
Supplementary Figure S11. Scatter plot of chronotype and rectal cancer association in females
Supplementary Figure S12. Scatter plot of chronotype and rectal cancer association

Supplementary Figure S13. Forest plot of chronotype and colorectal cancer association in males
Supplementary Figure S14. Forest plot of chronotype and colorectal cancer association in females
Supplementary Figure S15. Forest plot of chronotype and colorectal cancer association
Supplementary Figure S16. Forest plot of chronotype and colon cancer association in males
Supplementary Figure S17. Forest plot of chronotype and colon cancer association in females
Supplementary Figure S18. Forest plot of chronotype and colon cancer association
Supplementary Figure S19. Forest plot of chronotype and proximal colon cancer association
Supplementary Figure S20. Forest plot of chronotype and distal colon cancer association
Supplementary Figure S21. Forest plot of chronotype and rectal cancer association in males
Supplementary Figure S22. Forest plot of chronotype and rectal cancer association in females
Supplementary Figure S23. Forest plot of chronotype and rectal cancer association

Supplementary Figure S24. Funnel plot of chronotype and colorectal cancer association in males
Supplementary Figure S25. Funnel plot of chronotype and colorectal cancer association in females
Supplementary Figure S26. Funnel plot of chronotype and colorectal cancer association
Supplementary Figure S27. Funnel plot of chronotype and colon cancer association in males
Supplementary Figure S28. Funnel plot of chronotype and colon cancer association in females
Supplementary Figure S29. Funnel plot of chronotype and colon cancer association
Supplementary Figure S30. Funnel plot of chronotype and proximal colon cancer association
Supplementary Figure S31. Funnel plot of chronotype and distal colon cancer association
Supplementary Figure S32. Funnel plot of chronotype and rectal cancer association in males
Supplementary Figure S33. Funnel plot of chronotype and rectal cancer association in females
Supplementary Figure S34. Funnel plot of chronotype and rectal cancer association

Primary MR analyses: Frequent insomnia symptoms [Lane *et al.* (2019)]
Supplementary Figure S35. Scatter plot of frequent insomnia symptoms [Lane *et al.* (2019)] and colorectal cancer association in males
Supplementary Figure S36. Scatter plot of frequent insomnia symptoms [Lane *et al.* (2019)] and colorectal cancer association in females
Supplementary Figure S37. Scatter plot of frequent insomnia symptoms [Lane *et al.* (2019)] and colorectal cancer association
Supplementary Figure S38. Scatter plot of frequent insomnia symptoms [Lane *et al.* (2019)] and colon cancer association in males
Supplementary Figure S39. Scatter plot of frequent insomnia symptoms [Lane *et al.* (2019)] and colon cancer association in females
Supplementary Figure S40. Scatter plot of frequent insomnia symptoms [Lane *et al.* (2019)] and colon cancer association
Supplementary Figure S41. Scatter plot of frequent insomnia symptoms [Lane *et al.* (2019)] and proximal colon cancer association
Supplementary Figure S42. Scatter plot of frequent insomnia symptoms [Lane *et al.* (2019)] and distal colon cancer association
Supplementary Figure S43. Scatter plot of frequent insomnia symptoms [Lane *et al.* (2019)] and rectal cancer association in males
Supplementary Figure S44. Scatter plot of frequent insomnia symptoms [Lane *et al.* (2019)] and rectal cancer association in females
Supplementary Figure S45. Scatter plot of frequent insomnia symptoms [Lane *et al.* (2019)] and rectal cancer association

Supplementary Figure S46. Forest plot of frequent insomnia symptoms [Lane *et al.* (2019)] and colorectal cancer association in males
Supplementary Figure S47. Forest plot of frequent insomnia symptoms [Lane *et al.* (2019)] and colorectal cancer association in females
Supplementary Figure S48. Forest plot of frequent insomnia symptoms [Lane *et al.* (2019)] and colorectal cancer association
Supplementary Figure S49. Forest plot of frequent insomnia symptoms [Lane *et al.* (2019)] and colon cancer association in males
Supplementary Figure S50. Forest plot of frequent insomnia symptoms [Lane *et al.* (2019)] and colon cancer association in females
Supplementary Figure S51. Forest plot of frequent insomnia symptoms [Lane *et al.* (2019)] and colon cancer association
Supplementary Figure S52. Forest plot of frequent insomnia symptoms [Lane *et al.* (2019)] and proximal colon cancer association
Supplementary Figure S53. Forest plot of frequent insomnia symptoms [Lane *et al.* (2019)] and distal colon cancer association
Supplementary Figure S54. Forest plot of frequent insomnia symptoms [Lane *et al.* (2019)] and rectal cancer association in males

Supplementary Figure S55. Forest plot of frequent insomnia symptoms [Lane *et al.* (2019)] and rectal cancer association in females
Supplementary Figure S56. Forest plot of frequent insomnia symptoms [Lane *et al.* (2019)] and rectal cancer association

Supplementary Figure S57. Funnel plot of frequent insomnia symptoms [Lane *et al.* (2019)] and colorectal cancer association in males
Supplementary Figure S58. Funnel plot of frequent insomnia symptoms [Lane *et al.* (2019)] and colorectal cancer association in females
Supplementary Figure S59. Funnel plot of frequent insomnia symptoms [Lane *et al.* (2019)] and colorectal cancer association
Supplementary Figure S60. Funnel plot of frequent insomnia symptoms [Lane *et al.* (2019)] and colon cancer association in males
Supplementary Figure S61. Funnel plot of frequent insomnia symptoms [Lane *et al.* (2019)] and colon cancer association in females
Supplementary Figure S62. Funnel plot of frequent insomnia symptoms [Lane *et al.* (2019)] and colon cancer association
Supplementary Figure S63. Funnel plot of frequent insomnia symptoms [Lane *et al.* (2019)] and proximal colon cancer association
Supplementary Figure S64. Funnel plot of frequent insomnia symptoms [Lane *et al.* (2019)] and distal colon cancer association
Supplementary Figure S65. Funnel plot of frequent insomnia symptoms [Lane *et al.* (2019)] and rectal cancer association in males
Supplementary Figure S66. Funnel plot of frequent insomnia symptoms [Lane *et al.* (2019)] and rectal cancer association in females
Supplementary Figure S67. Funnel plot of frequent insomnia symptoms [Lane *et al.* (2019)] and rectal cancer association

Primary MR analyses: Any insomnia symptoms [Lane *et al.* (2019)]
Supplementary Figure S68. Scatter plot of any insomnia symptoms [Lane *et al.* (2019)] and colorectal cancer association
Supplementary Figure S69. Scatter plot of any insomnia symptoms [Lane *et al.* (2019)] and colon cancer association
Supplementary Figure S70. Scatter plot of any insomnia symptoms [Lane *et al.* (2019)] and proximal colon cancer association
Supplementary Figure S71. Scatter plot of any insomnia symptoms [Lane *et al.* (2019)] and distal colon cancer association
Supplementary Figure S72. Scatter plot of any insomnia symptoms [Lane *et al.* (2019)] and rectal cancer association

Supplementary Figure S73. Forest plot of any insomnia symptoms [Lane *et al.* (2019)] and colorectal cancer association
Supplementary Figure S74. Forest plot of any insomnia symptoms [Lane *et al.* (2019)] and colon cancer association
Supplementary Figure S75. Forest plot of any insomnia symptoms [Lane *et al.* (2019)] and proximal colon cancer association
Supplementary Figure S76. Forest plot of any insomnia symptoms [Lane *et al.* (2019)] and distal colon cancer association
Supplementary Figure S77. Forest plot of any insomnia symptoms [Lane *et al.* (2019)] and rectal cancer association

Supplementary Figure S78. Funnel plot of any insomnia symptoms [Lane *et al.* (2019)] and colorectal cancer association
Supplementary Figure S79. Funnel plot of any insomnia symptoms [Lane *et al.* (2019)] and colon cancer association
Supplementary Figure S80. Funnel plot of any insomnia symptoms [Lane *et al.* (2019)] and proximal colon cancer association
Supplementary Figure S81. Funnel plot of any insomnia symptoms [Lane *et al.* (2019)] and distal colon cancer association
Supplementary Figure S82. Funnel plot of any insomnia symptoms [Lane *et al.* (2019)] and rectal cancer association

Primary MR analyses: Insomnia [Jansen *et al.* (2019)]

Supplementary Figure S83. Scatter plot of frequent insomnia symptoms [Jansen *et al.* (2019)] and colorectal cancer association in males
Supplementary Figure S84. Scatter plot of frequent insomnia symptoms [Jansen *et al.* (2019)] and colorectal cancer association in females
Supplementary Figure S85. Scatter plot of frequent insomnia symptoms [Jansen *et al.* (2019)] and colorectal cancer association
Supplementary Figure S86. Scatter plot of frequent insomnia symptoms [Jansen *et al.* (2019)] and colon cancer association in males
Supplementary Figure S87. Scatter plot of frequent insomnia symptoms [Jansen *et al.* (2019)] and colon cancer association in females
Supplementary Figure S88. Scatter plot of frequent insomnia symptoms [Jansen *et al.* (2019)] and colon cancer association
Supplementary Figure S89. Scatter plot of frequent insomnia symptoms [Jansen *et al.* (2019)] and proximal colon cancer association
Supplementary Figure S90. Scatter plot of frequent insomnia symptoms [Jansen *et al.* (2019)] and distal colon cancer association
Supplementary Figure S91. Scatter plot of frequent insomnia symptoms [Jansen *et al.* (2019)] and rectal cancer association in males
Supplementary Figure S92. Scatter plot of frequent insomnia symptoms [Jansen *et al.* (2019)] and rectal cancer association in females
Supplementary Figure S93. Scatter plot of frequent insomnia symptoms [Jansen *et al.* (2019)] and rectal cancer association

Supplementary Figure S94. Forest plot of frequent insomnia symptoms [Jansen *et al.* (2019)] and colorectal cancer association in males
Supplementary Figure S95. Forest plot of frequent insomnia symptoms [Jansen *et al.* (2019)] and colorectal cancer association in females
Supplementary Figure S96. Forest plot of frequent insomnia symptoms [Jansen *et al.* (2019)] and colorectal cancer association
Supplementary Figure S97. Forest plot of frequent insomnia symptoms [Jansen *et al.* (2019)] and colon cancer association in males
Supplementary Figure S98. Forest plot of frequent insomnia symptoms [Jansen *et al.* (2019)] and colon cancer association in females
Supplementary Figure S99. Forest plot of frequent insomnia symptoms [Jansen *et al.* (2019)] and colon cancer association
Supplementary Figure S100. Forest plot of frequent insomnia symptoms [Jansen *et al.* (2019)] and proximal colon cancer association
Supplementary Figure S101. Forest plot of frequent insomnia symptoms [Jansen *et al.* (2019)] and distal colon cancer association
Supplementary Figure S102. Forest plot of frequent insomnia symptoms [Jansen *et al.* (2019)] and rectal cancer association in males
Supplementary Figure S103. Forest plot of frequent insomnia symptoms [Jansen *et al.* (2019)] and rectal cancer association in females
Supplementary Figure S104. Forest plot of frequent insomnia symptoms [Jansen *et al.* (2019)] and rectal cancer association

Supplementary Figure S105. Funnel plot of frequent insomnia symptoms [Jansen *et al.* (2019)] and colorectal cancer association in males
Supplementary Figure S106. Funnel plot of frequent insomnia symptoms [Jansen *et al.* (2019)] and colorectal cancer association in females
Supplementary Figure S107. Funnel plot of frequent insomnia symptoms [Jansen *et al.* (2019)] and colorectal cancer association
Supplementary Figure S108. Funnel plot of frequent insomnia symptoms [Jansen *et al.* (2019)] and colon cancer association in males
Supplementary Figure S109. Funnel plot of frequent insomnia symptoms [Jansen *et al.* (2019)] and colon cancer association in females
Supplementary Figure S110. Funnel plot of frequent insomnia symptoms [Jansen *et al.* (2019)] and colon cancer association
Supplementary Figure S111. Funnel plot of frequent insomnia symptoms [Jansen *et al.* (2019)] and proximal colon cancer association
Supplementary Figure S112. Funnel plot of frequent insomnia symptoms [Jansen *et al.* (2019)] and distal colon cancer association
Supplementary Figure S113. Funnel plot of frequent insomnia symptoms [Jansen *et al.* (2019)] and rectal cancer association in males
Supplementary Figure S114. Funnel plot of frequent insomnia symptoms [Jansen *et al.* (2019)] and rectal cancer association in females
Supplementary Figure S115. Funnel plot of frequent insomnia symptoms [Jansen *et al.* (2019)] and rectal cancer association

Primary MR analyses: Sleep duration

Supplementary Figure S116. Scatter plot of sleep duration and colorectal cancer association in males
Supplementary Figure S117. Scatter plot of sleep duration and colorectal cancer association in females
Supplementary Figure S118. Scatter plot of sleep duration and colorectal cancer association
Supplementary Figure S119. Scatter plot of sleep duration and colon cancer association in males
Supplementary Figure S120. Scatter plot of sleep duration and colon cancer association in females
Supplementary Figure S121. Scatter plot of sleep duration and colon cancer association
Supplementary Figure S122. Scatter plot of sleep duration and proximal colon cancer association
Supplementary Figure S123. Scatter plot of sleep duration and distal colon cancer association
Supplementary Figure S124. Scatter plot of sleep duration and rectal cancer association in males
Supplementary Figure S125. Scatter plot of sleep duration and rectal cancer association in females
Supplementary Figure S126. Scatter plot of sleep duration and rectal cancer association

Supplementary Figure S127. Forest plot of sleep duration and colorectal cancer association in males
Supplementary Figure S128. Forest plot of sleep duration and colorectal cancer association in females
Supplementary Figure S129. Forest plot of sleep duration and colorectal cancer association
Supplementary Figure S130. Forest plot of sleep duration and colon cancer association in males
Supplementary Figure S131. Forest plot of sleep duration and colon cancer association in females
Supplementary Figure S132. Forest plot of sleep duration and colon cancer association
Supplementary Figure S133. Forest plot of sleep duration and proximal colon cancer association
Supplementary Figure S134. Forest plot of sleep duration and distal colon cancer association
Supplementary Figure S135. Forest plot of sleep duration and rectal cancer association in males
Supplementary Figure S136. Forest plot of sleep duration and rectal cancer association in females
Supplementary Figure S137. Forest plot of sleep duration and rectal cancer association

Supplementary Figure S138. Funnel plot of sleep duration and colorectal cancer association in males
Supplementary Figure S139. Funnel plot of sleep duration and colorectal cancer association in females
Supplementary Figure S140. Funnel plot of sleep duration and colorectal cancer association
Supplementary Figure S141. Funnel plot of sleep duration and colon cancer association in males
Supplementary Figure S142. Funnel plot of sleep duration and colon cancer association in females
Supplementary Figure S143. Funnel plot of sleep duration and colon cancer association
Supplementary Figure S144. Funnel plot of sleep duration and proximal colon cancer association
Supplementary Figure S145. Funnel plot of sleep duration and distal colon cancer association
Supplementary Figure S146. Funnel plot of sleep duration and rectal cancer association in males

Supplementary Figure S147. Funnel plot of sleep duration and rectal cancer association in females
Supplementary Figure S148. Funnel plot of sleep duration and rectal cancer association

Supplementary Figure S1. Flow chart of genetic variant selection

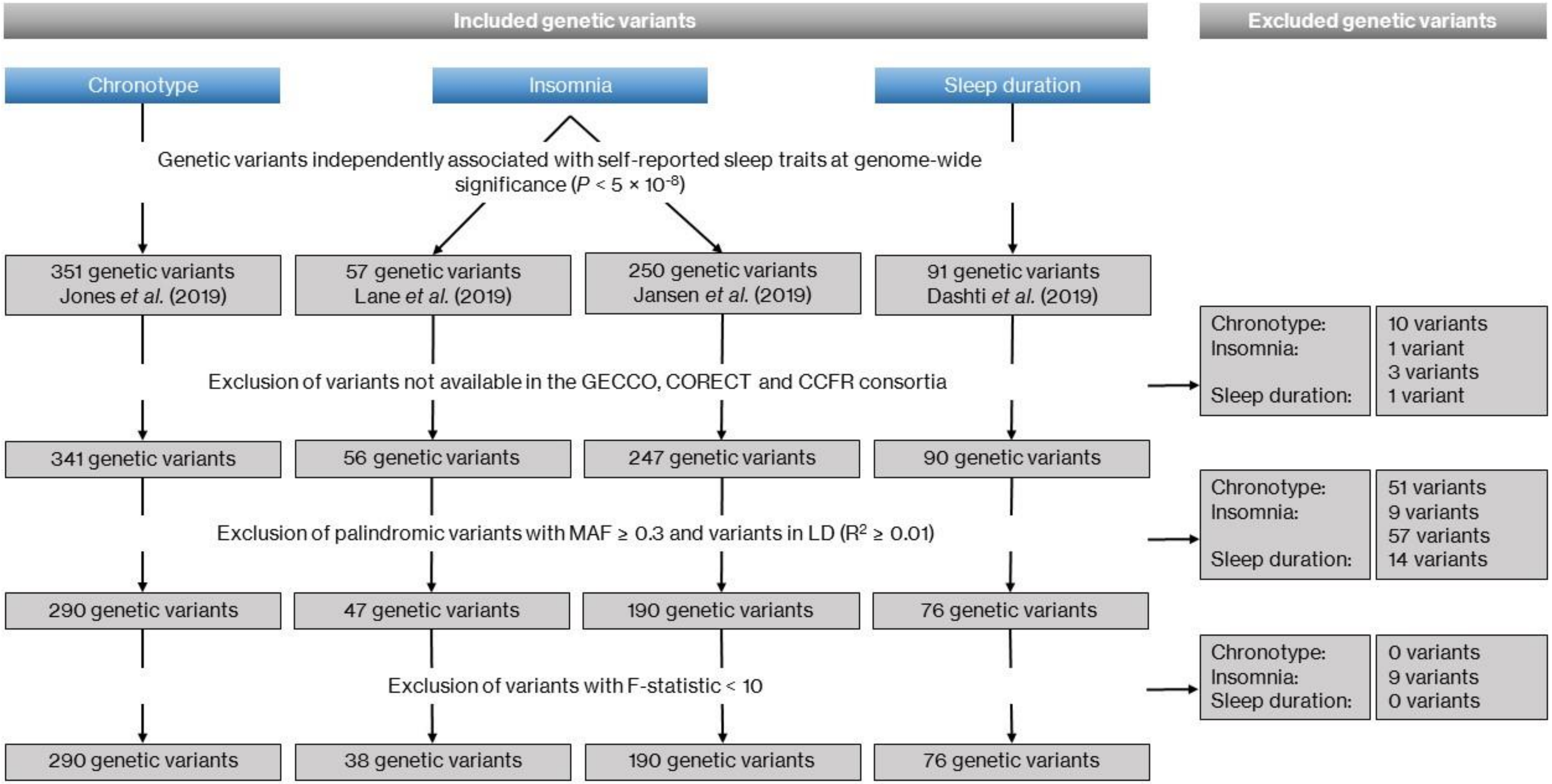

Abbreviations: CCFR: Colon Cancer Family Registry; CORECT: Colorectal Cancer Transdisciplinary Study; GECCO: Genetics and Epidemiology of Colorectal Cancer Consortium; LD: linkage disequilibrium; MAF: minor allele frequency

Primary MR analyses: Chronotype

Supplementary Figure S2. Scatter plot of chronotype and colorectal cancer association in males

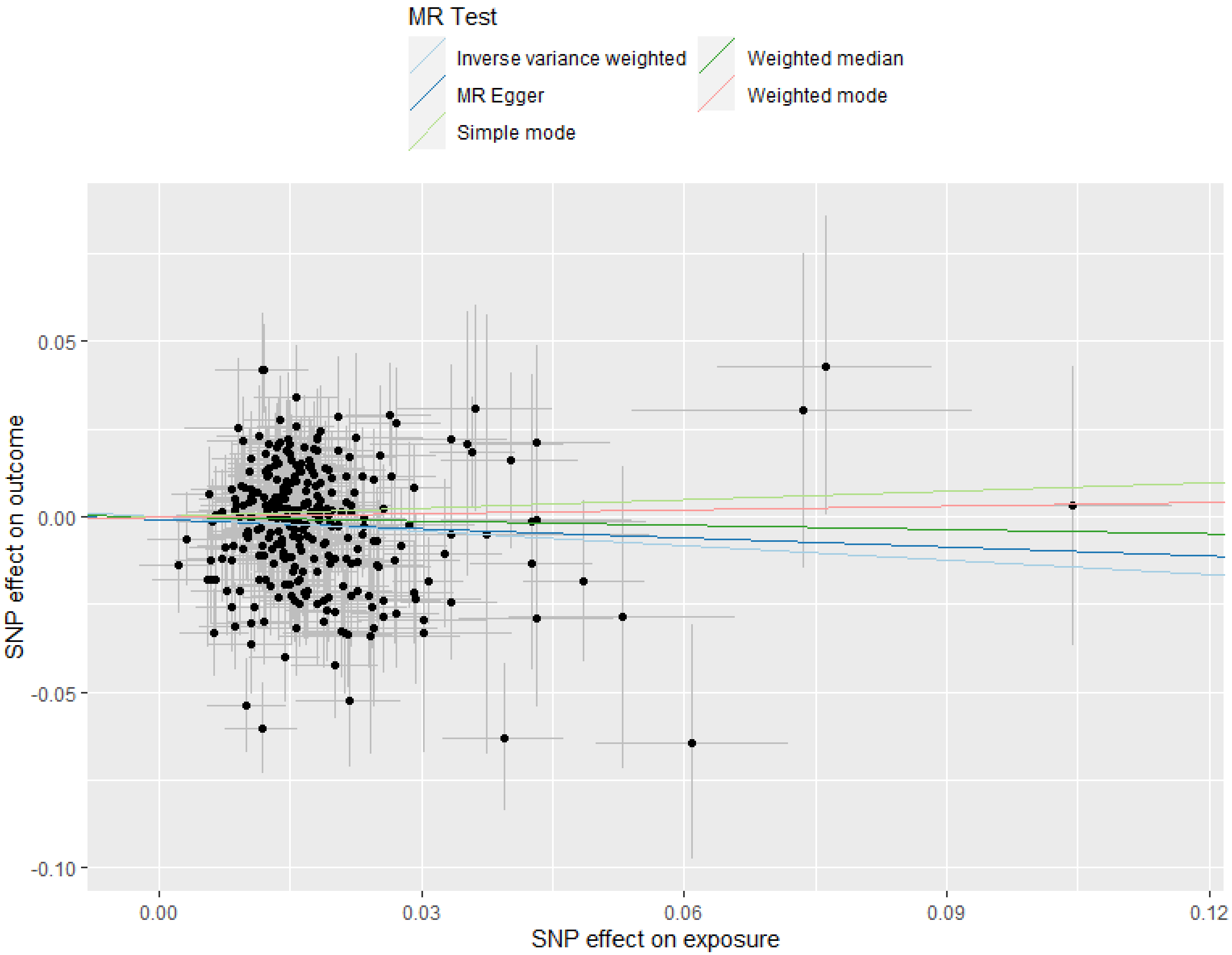

Abbreviations: MR: Mendelian randomization; SNP: Single Nucleotide Polymorphism

Supplementary Figure S3. Scatter plot of chronotype and colorectal cancer association in females

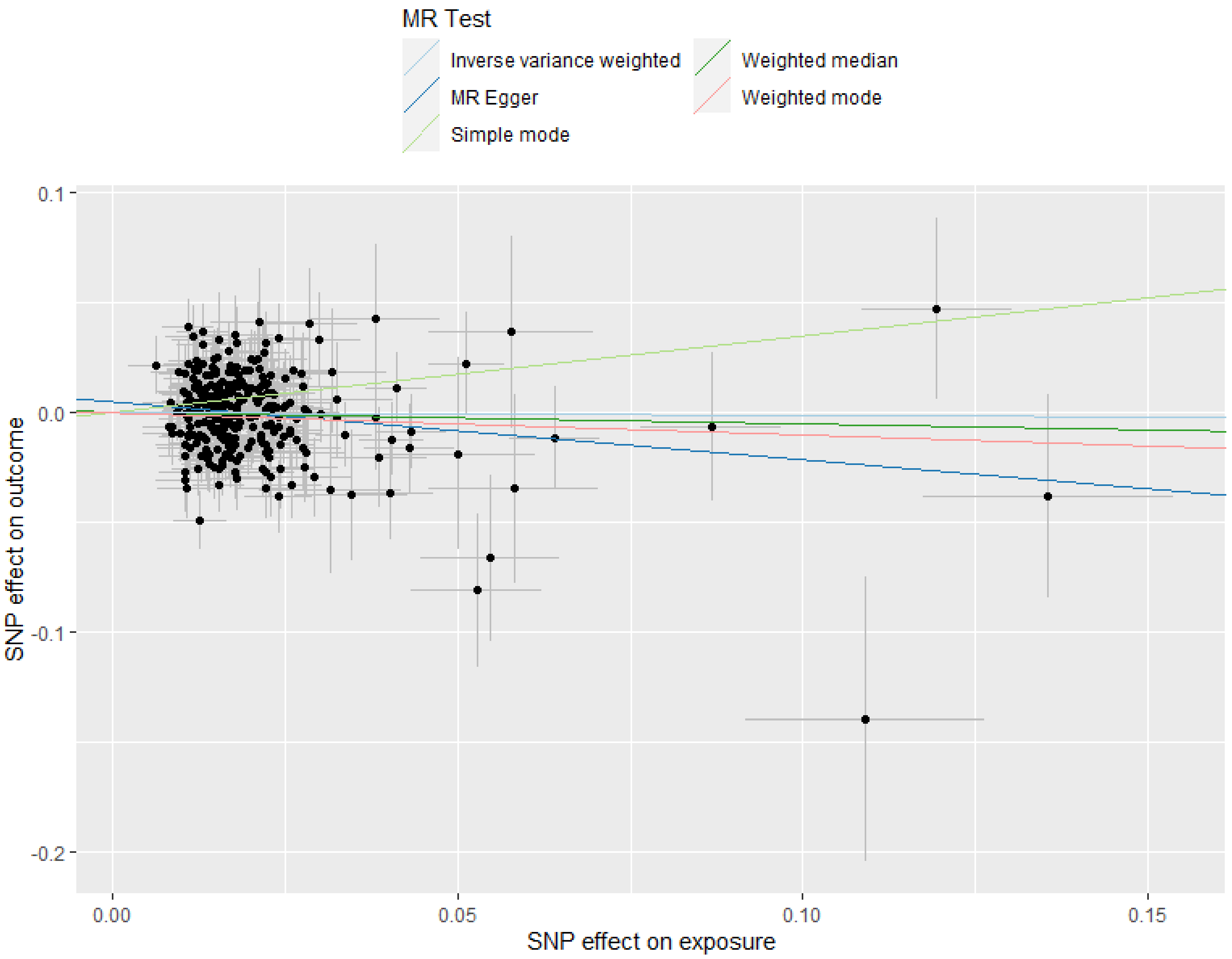

Abbreviations: MR: Mendelian randomization; SNP: Single Nucleotide Polymorphism

Supplementary Figure S4. Scatter plot of chronotype and colorectal cancer association

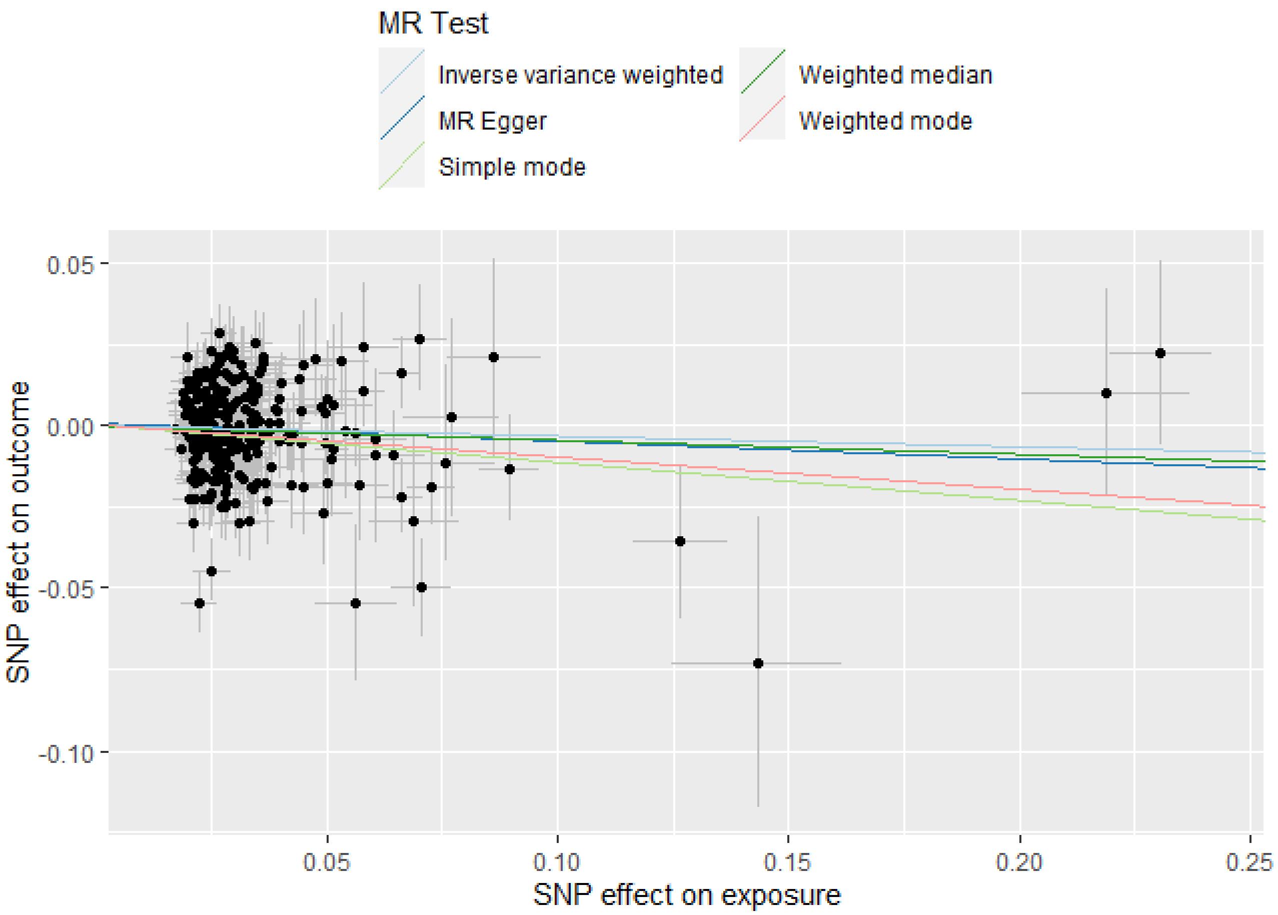

Abbreviations: MR: Mendelian randomization; SNP: Single Nucleotide Polymorphism

Supplementary Figure S5. Scatter plot of chronotype and colon cancer association in males

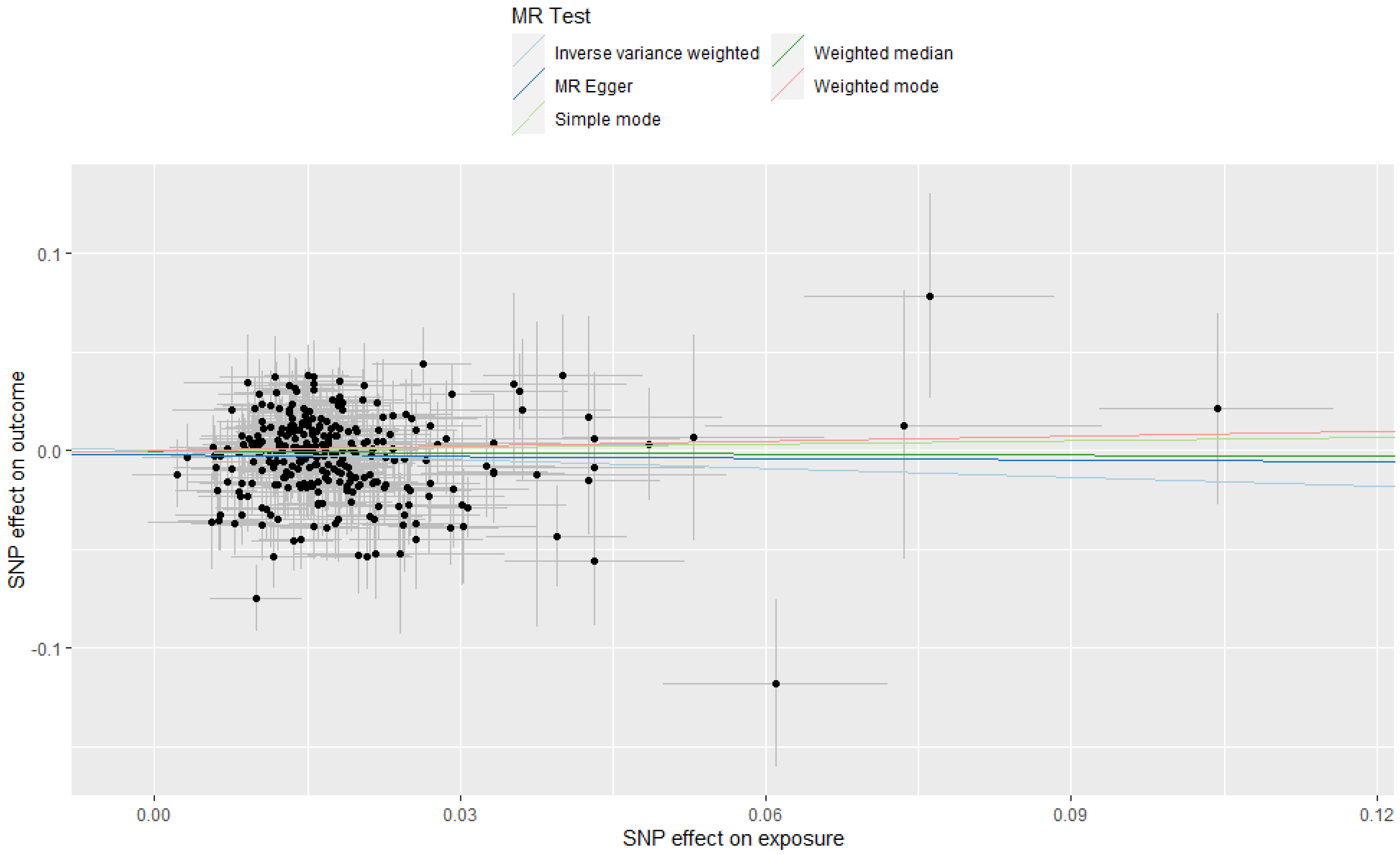

Abbreviations: MR: Mendelian randomization; SNP: Single Nucleotide Polymorphism

Supplementary Figure S6. Scatter plot of chronotype and colon cancer association in females

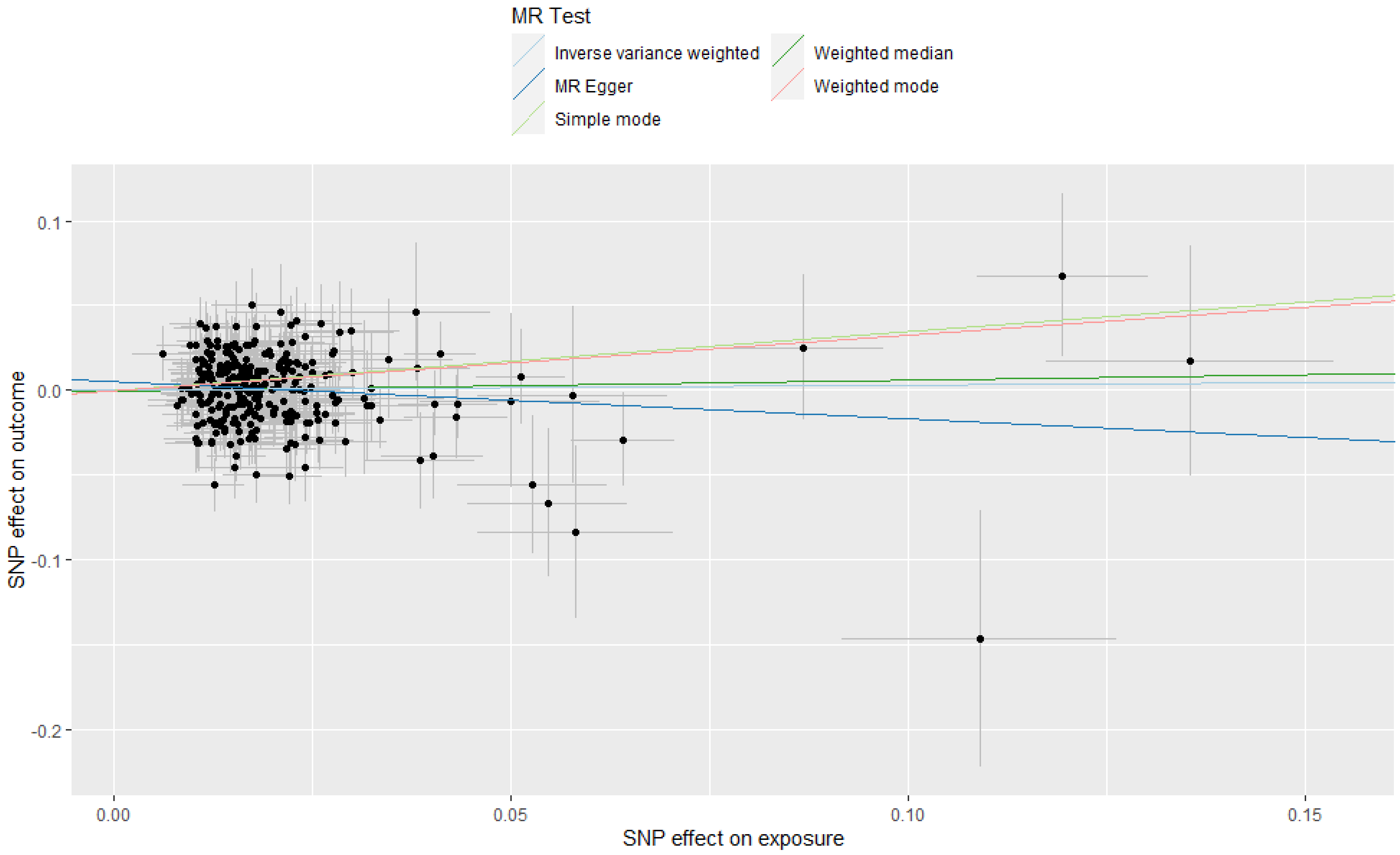

Abbreviations: MR: Mendelian randomization; SNP: Single Nucleotide Polymorphism

Supplementary Figure S7. Scatter plot of chronotype and colon cancer association

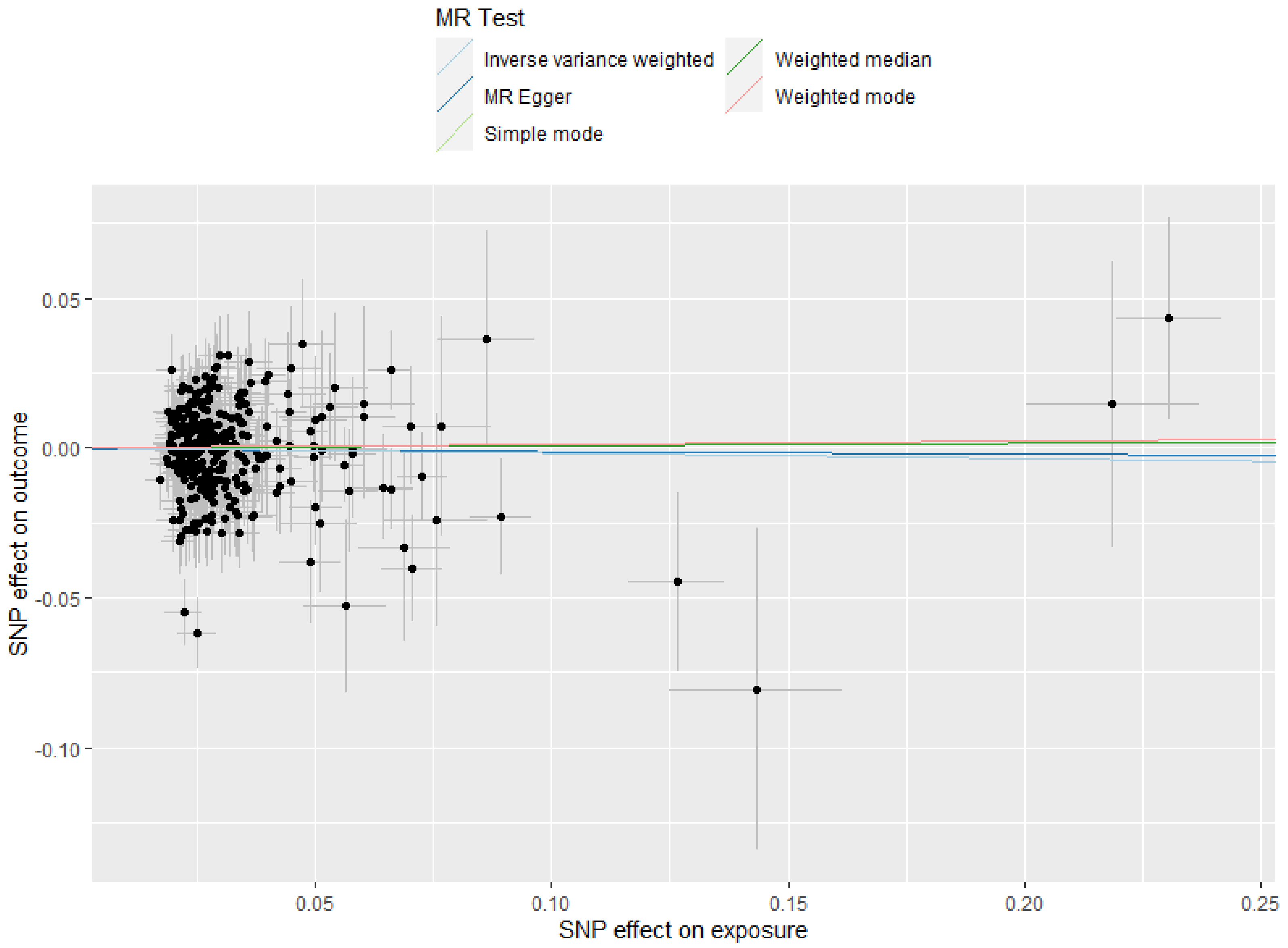

Abbreviations: MR: Mendelian randomization; SNP: Single Nucleotide Polymorphism

Supplementary Figure S8. Scatter plot of chronotype and proximal colon cancer association

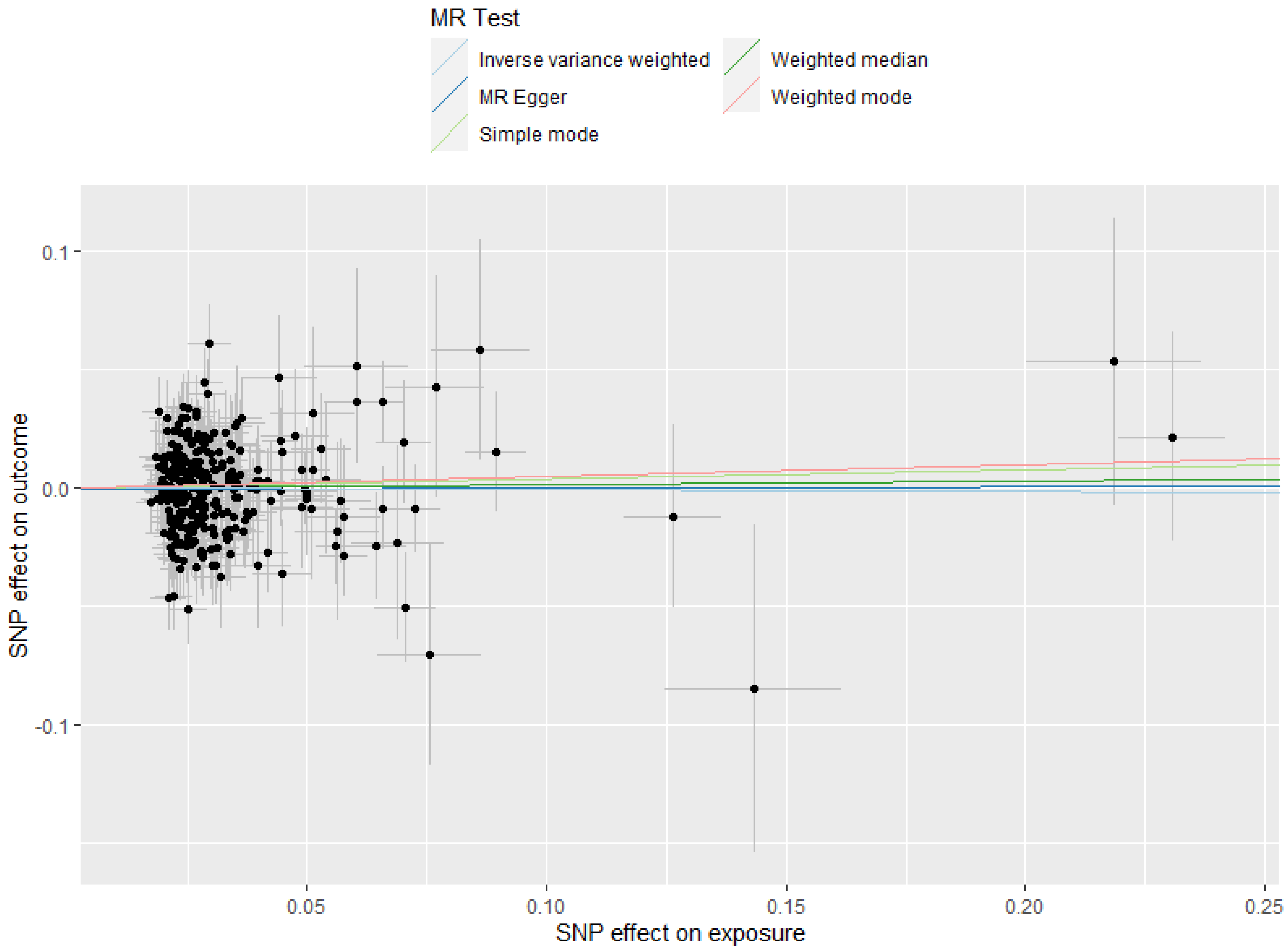

Abbreviations: MR: Mendelian randomization; SNP: Single Nucleotide Polymorphism

Supplementary Figure S9. Scatter plot of chronotype and distal colon cancer association

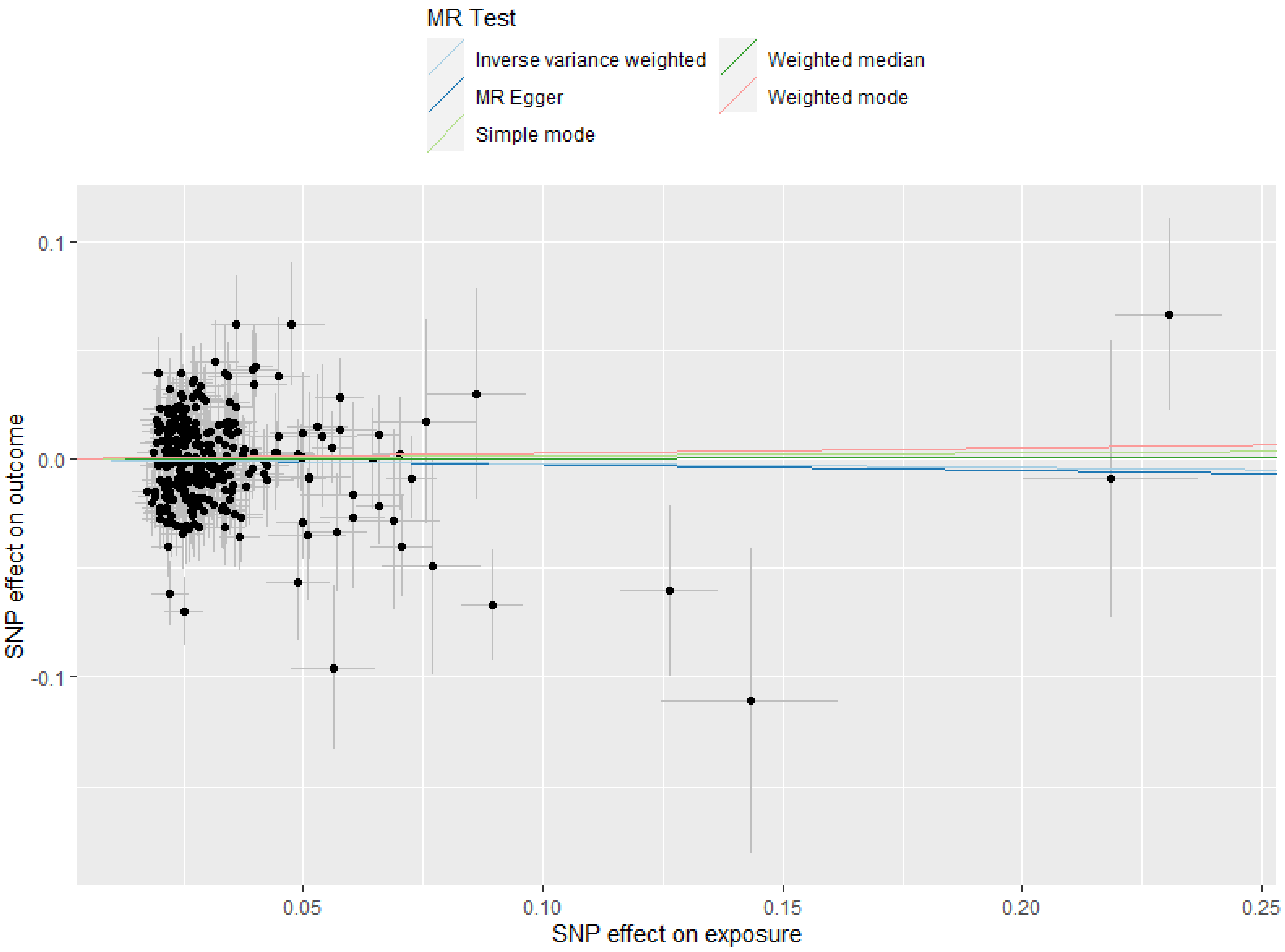

Abbreviations: MR: Mendelian randomization; SNP: Single Nucleotide Polymorphism

Supplementary Figure S10. Scatter plot of chronotype and rectal cancer association in males

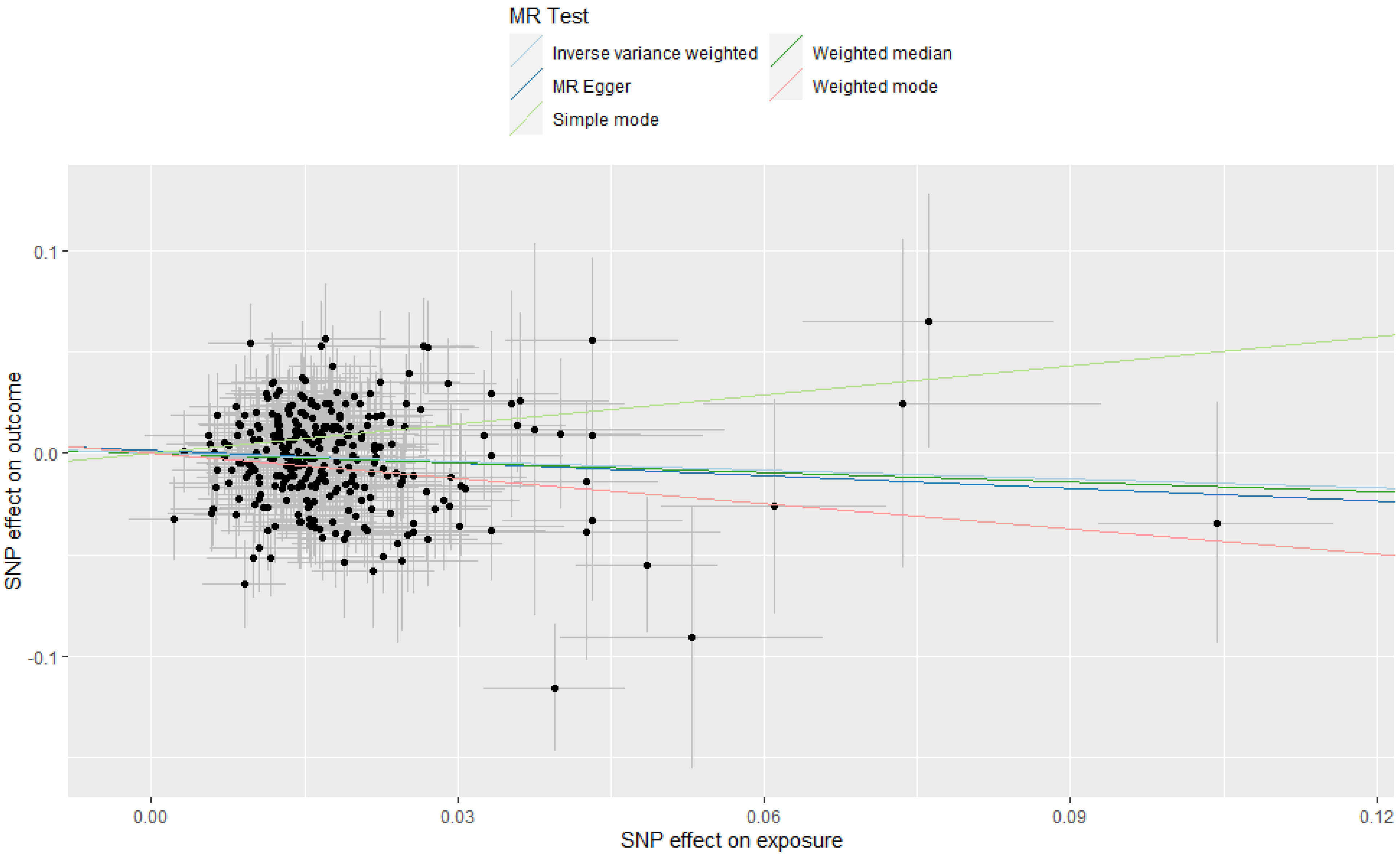

Abbreviations: MR: Mendelian randomization; SNP: Single Nucleotide Polymorphism

Supplementary Figure S11. Scatter plot of chronotype and rectal cancer association in females

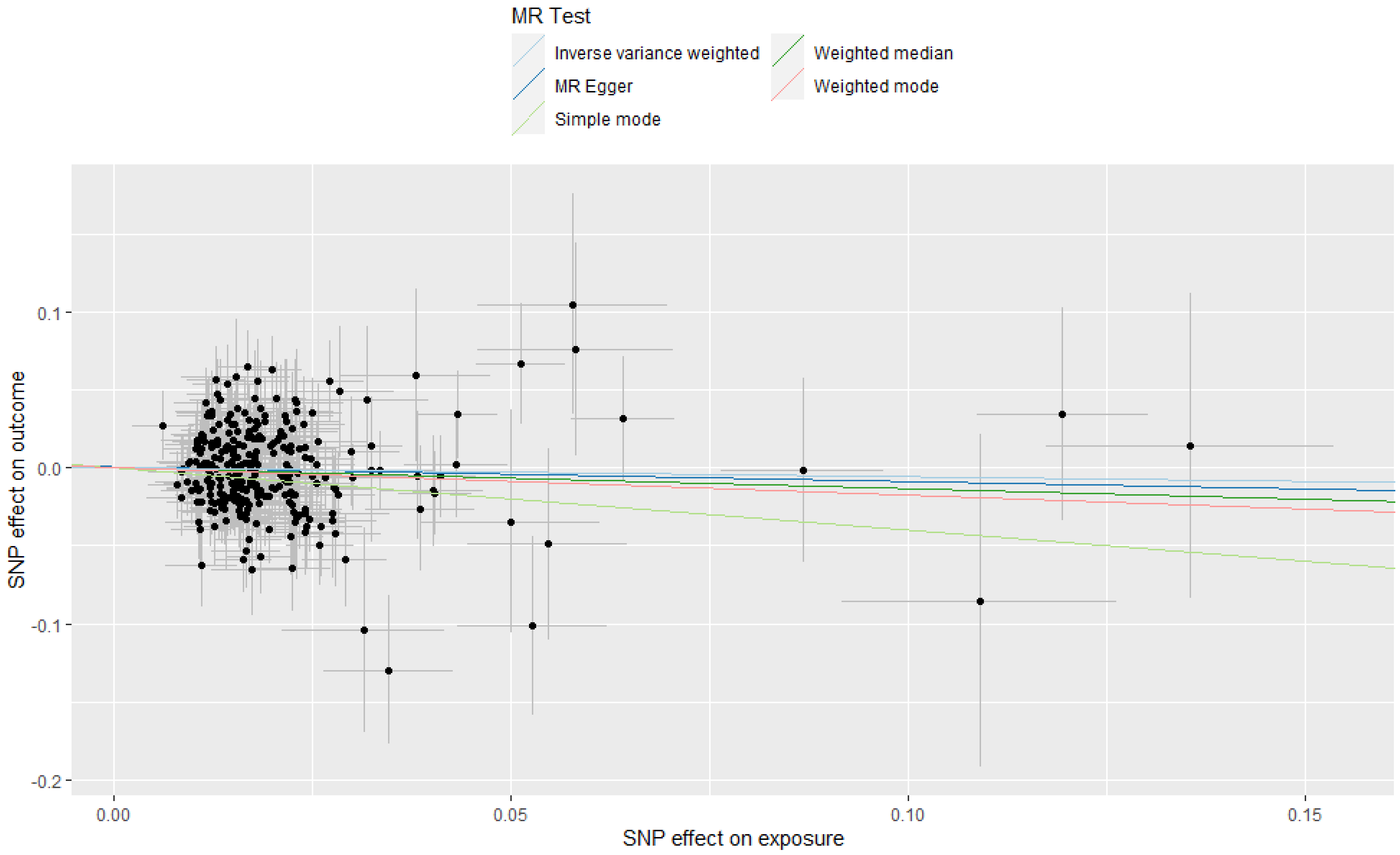

Abbreviations: MR: Mendelian randomization; SNP: Single Nucleotide Polymorphism

Supplementary Figure S12. Scatter plot of chronotype and rectal cancer association

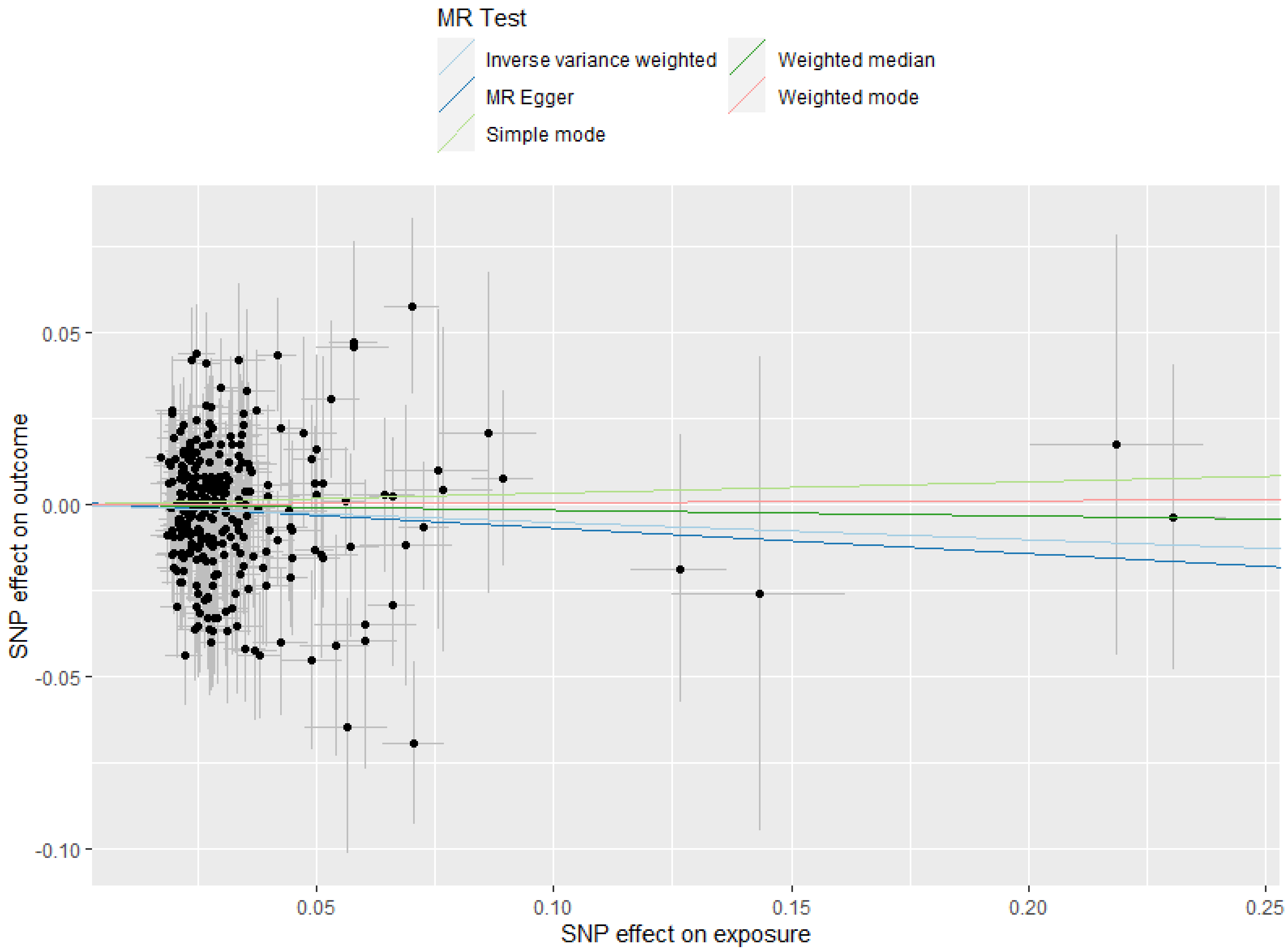

Abbreviations: MR: Mendelian randomization; SNP: Single Nucleotide Polymorphism

Supplementary Figure S13. Forest plot of chronotype and colorectal cancer association in males

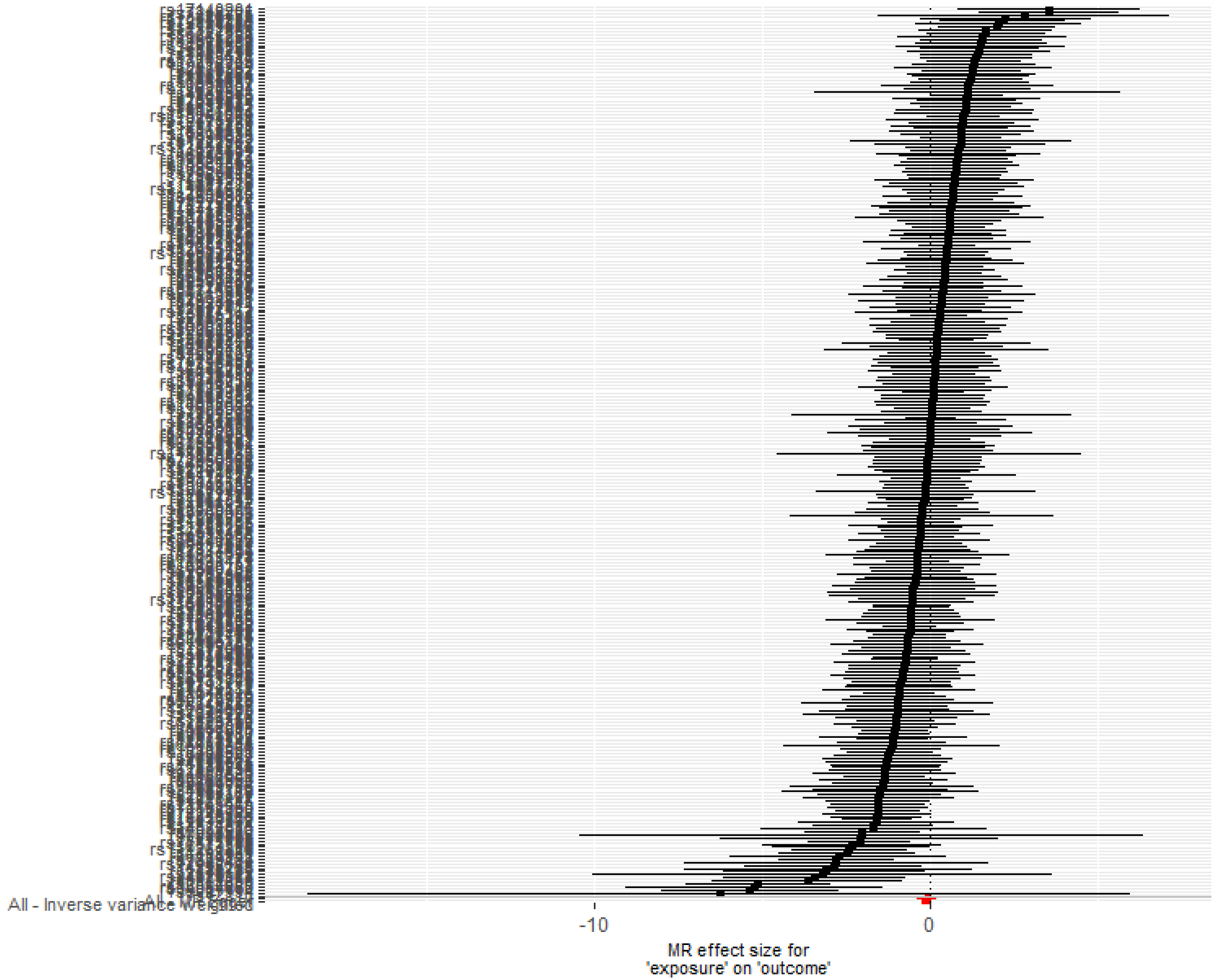

Abbreviations: MR: Mendelian randomization

Supplementary Figure S14. Forest plot of chronotype and colorectal cancer association in females

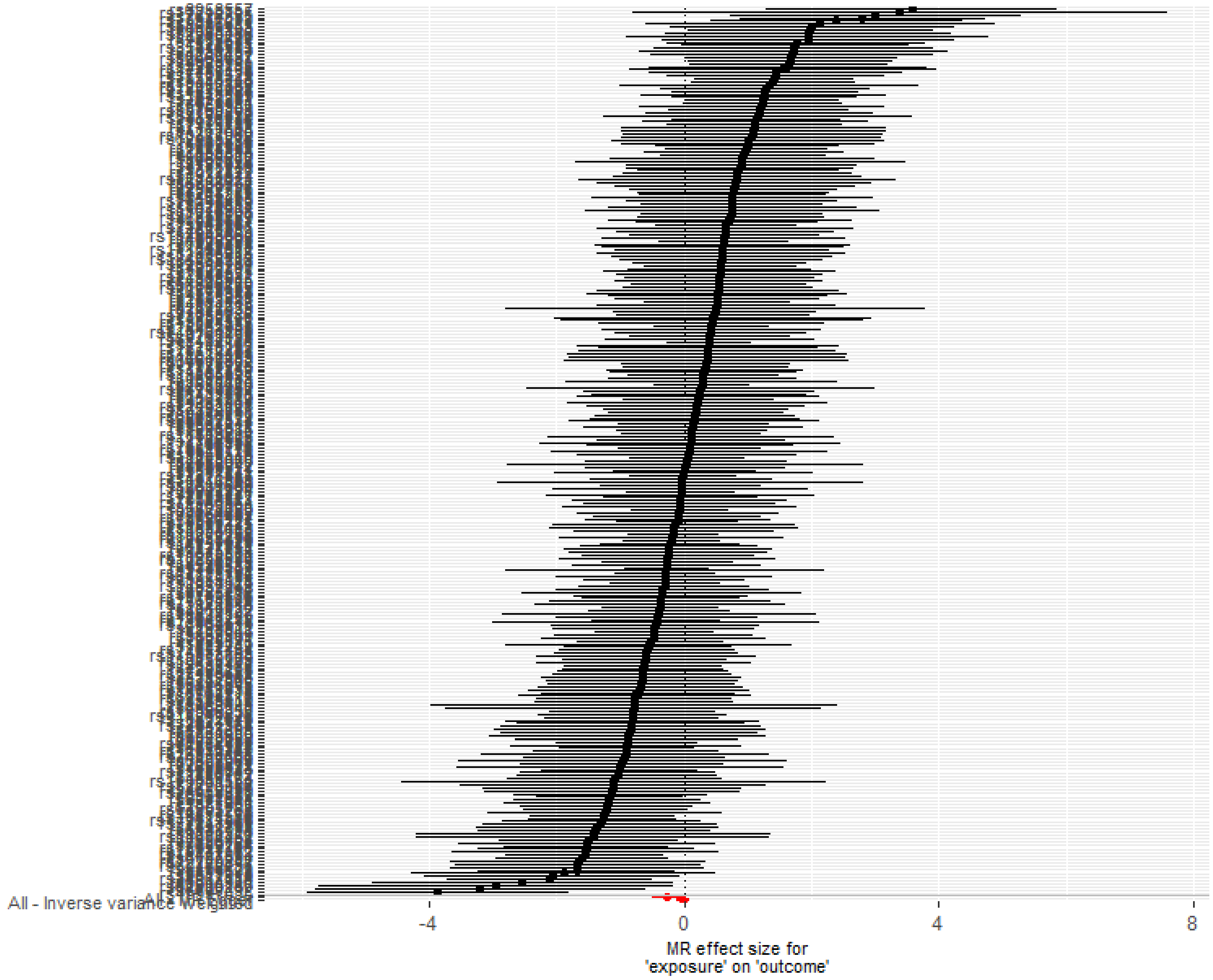

Abbreviations: MR: Mendelian randomization

Supplementary Figure S15. Forest plot of chronotype and colorectal cancer association

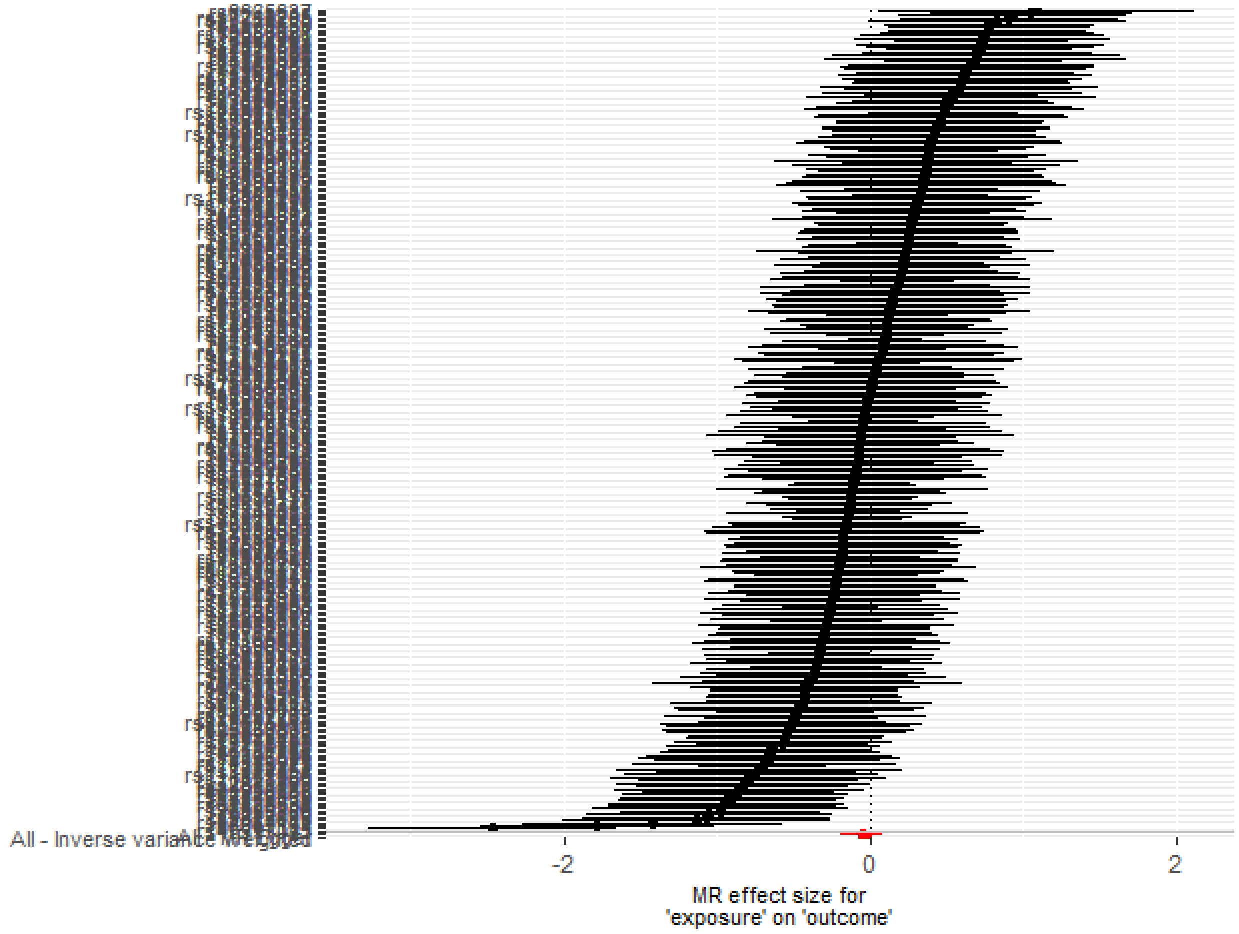

Abbreviations: MR: Mendelian randomization

Supplementary Figure S16. Forest plot of chronotype and colon cancer association in males

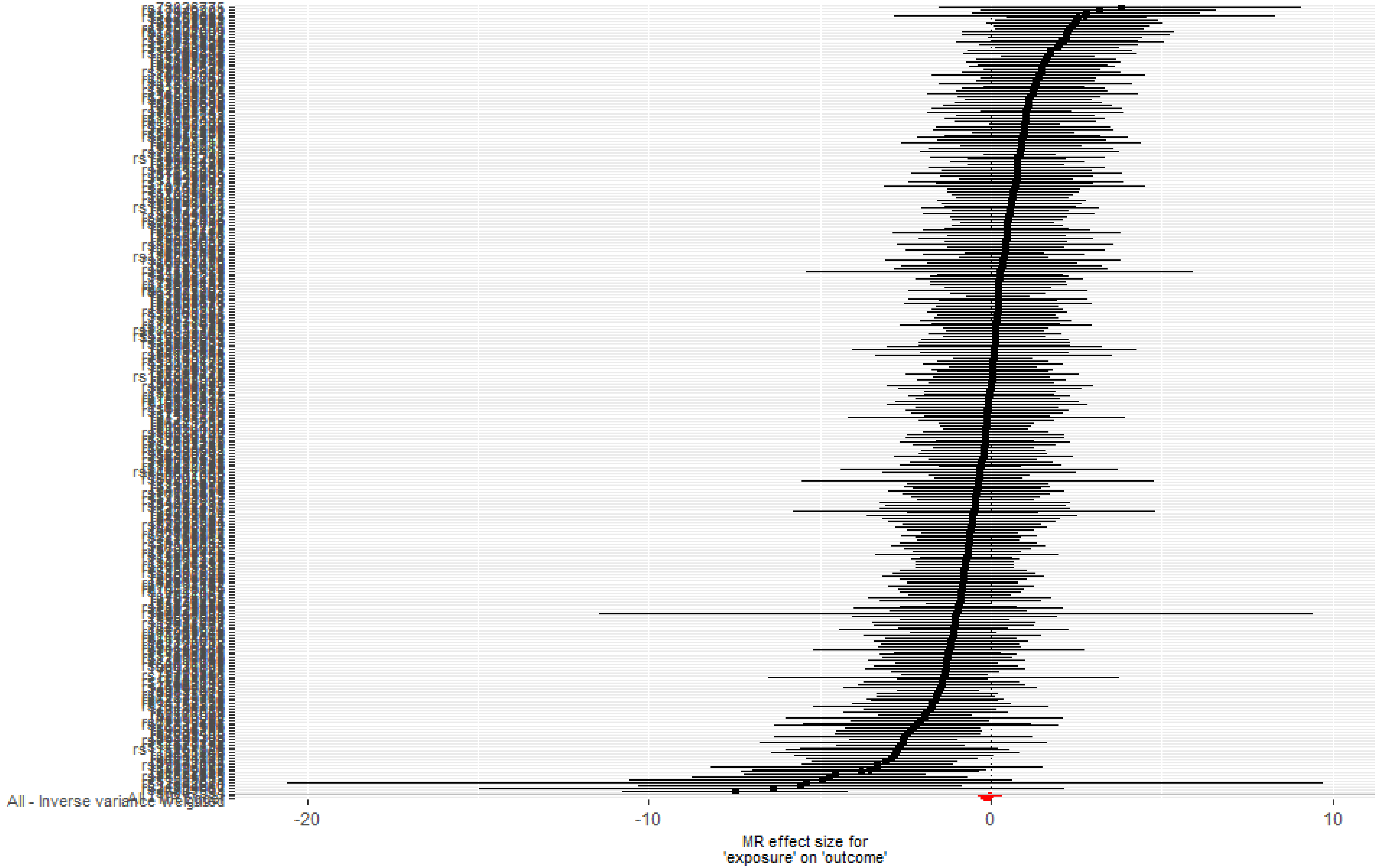

Abbreviations: MR: Mendelian randomization

Supplementary Figure S17. Forest plot of chronotype and colon cancer association in females

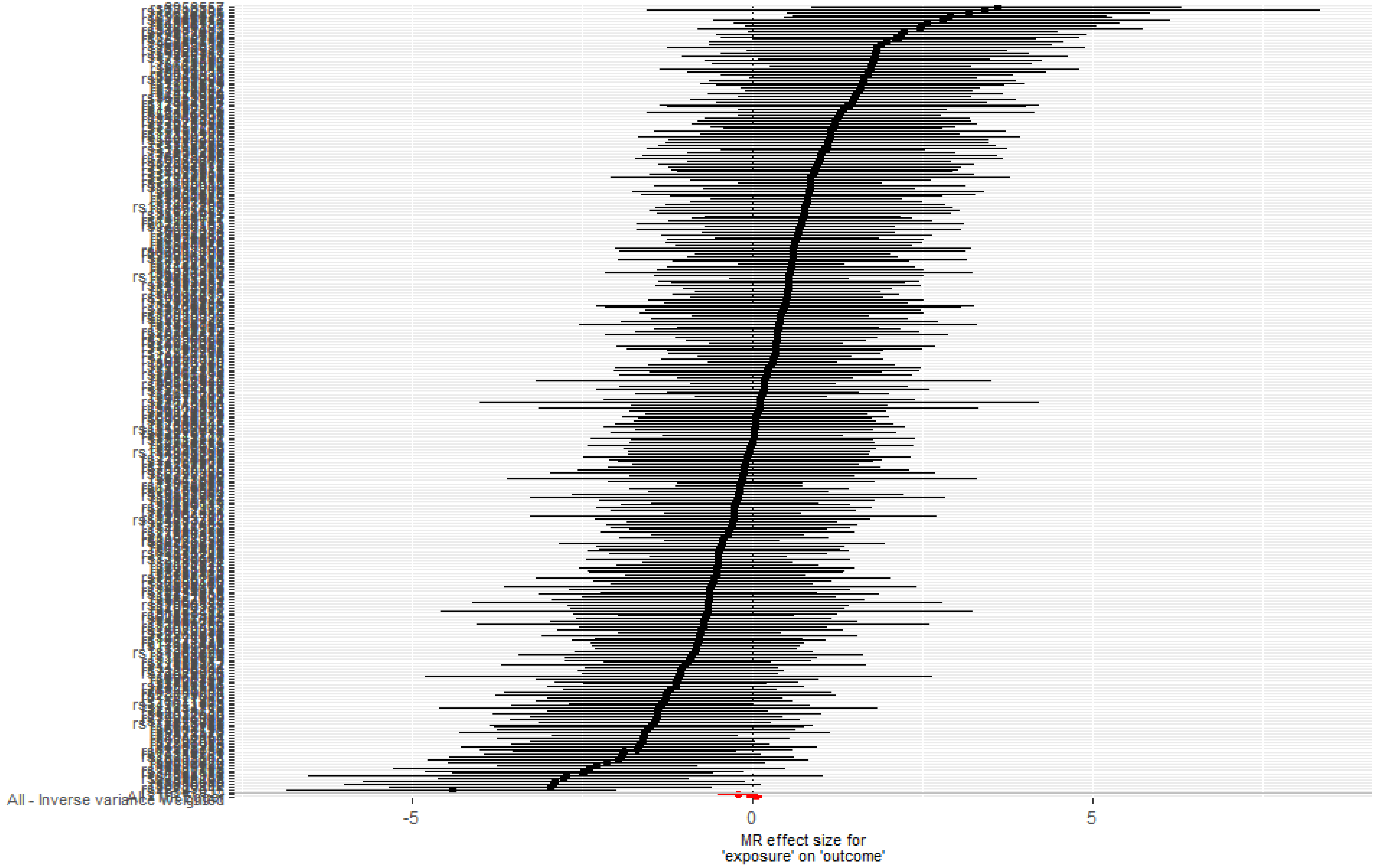

Abbreviations: MR: Mendelian randomization

Supplementary Figure S18. Forest plot of chronotype and colon cancer association

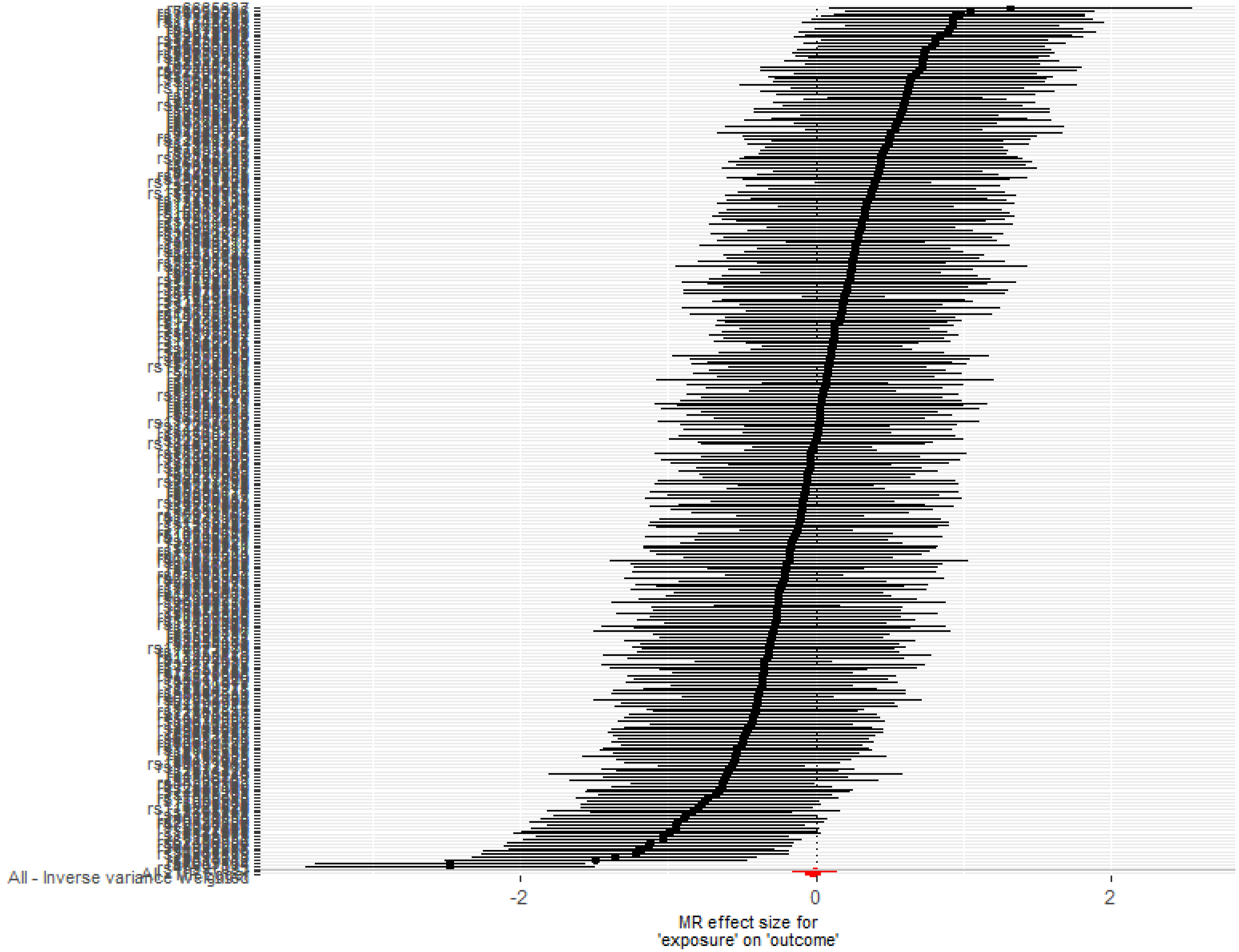

Abbreviations: MR: Mendelian randomization

Supplementary Figure S19. Forest plot of chronotype and proximal colon cancer association

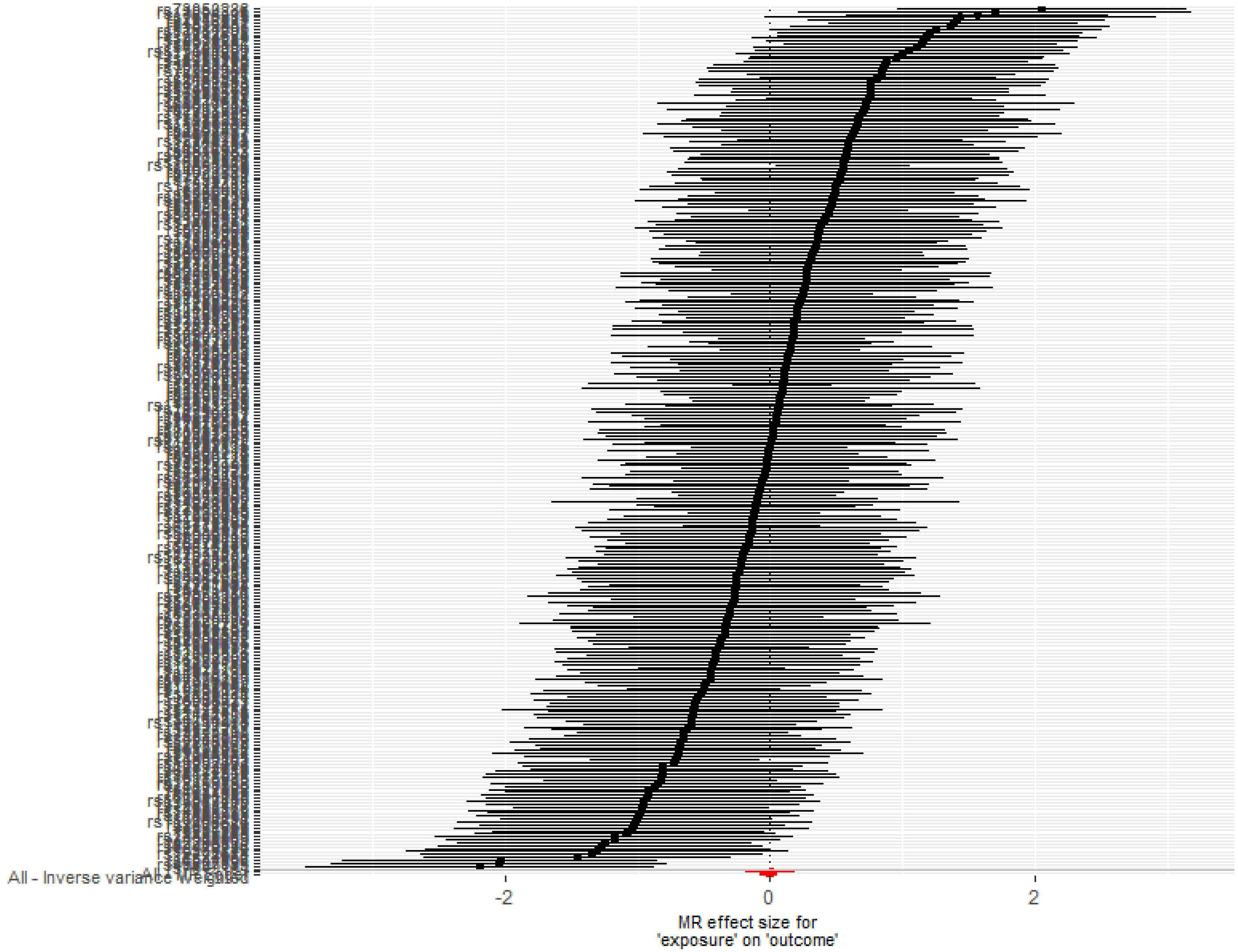

Abbreviations: MR: Mendelian randomization

Supplementary Figure S20. Forest plot of chronotype and distal colon cancer association

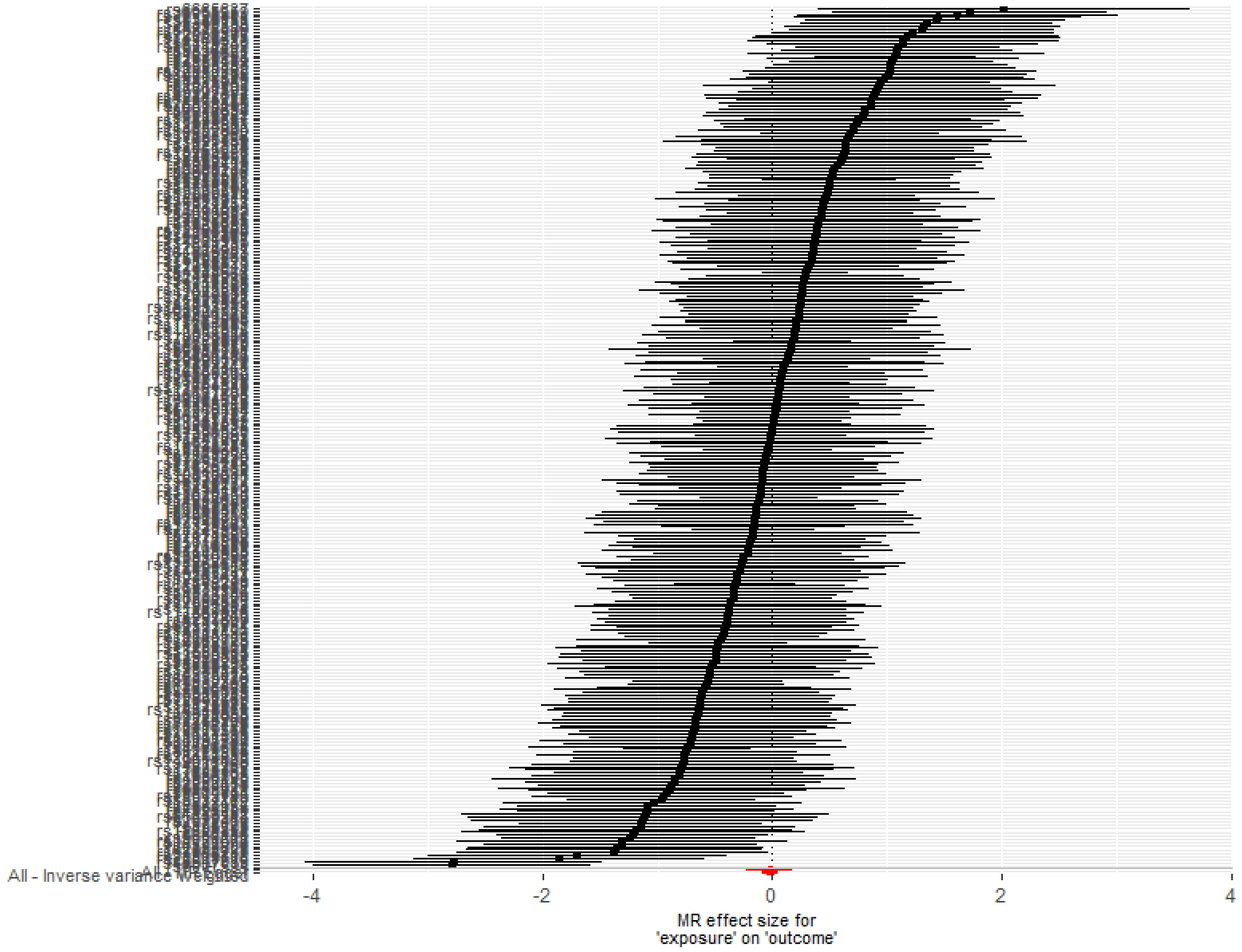

Abbreviations: MR: Mendelian randomization

Supplementary Figure S21. Forest plot of chronotype and rectal cancer association in males

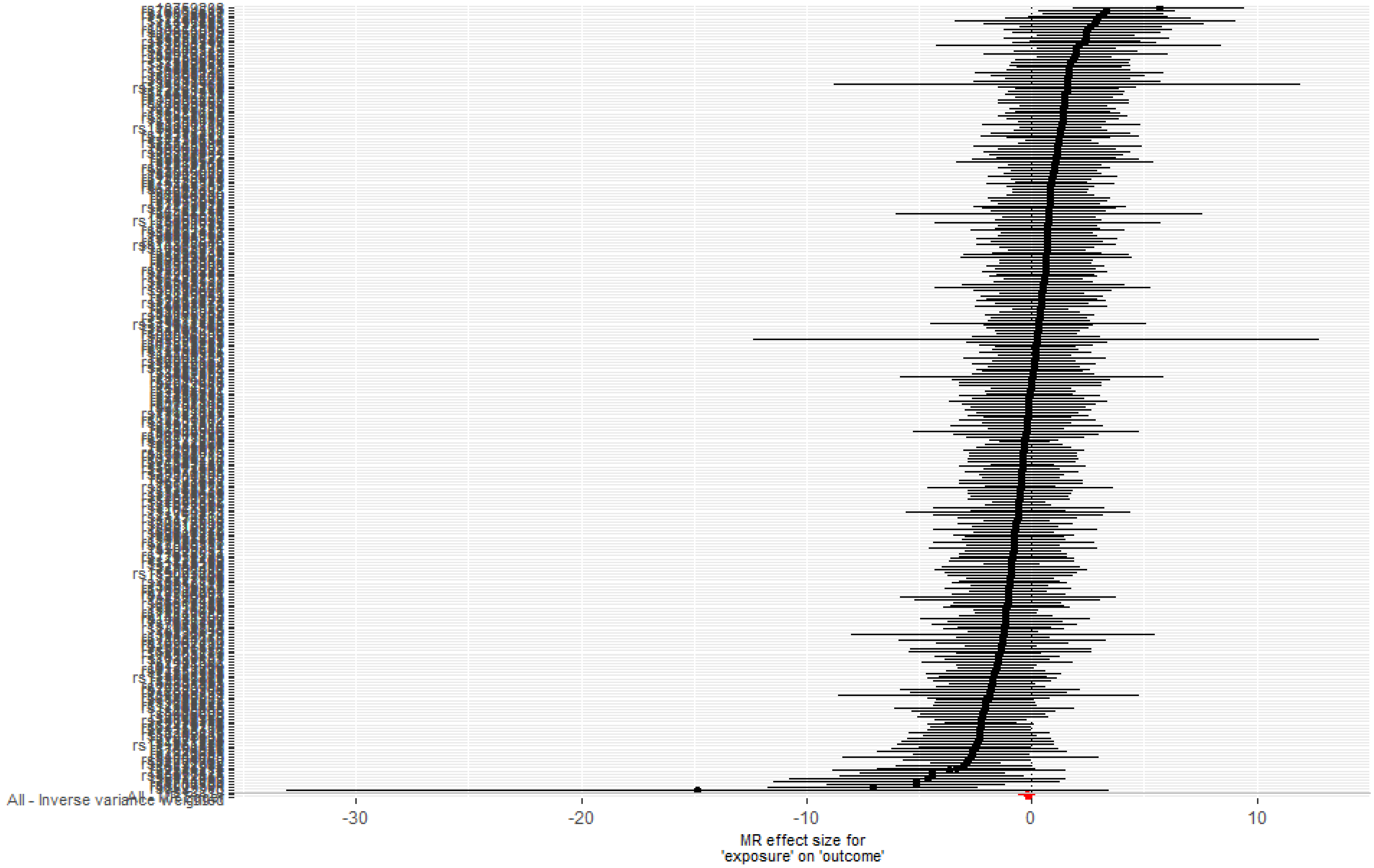

Abbreviations: MR: Mendelian randomization

Supplementary Figure S22. Forest plot of chronotype and rectal cancer association in females

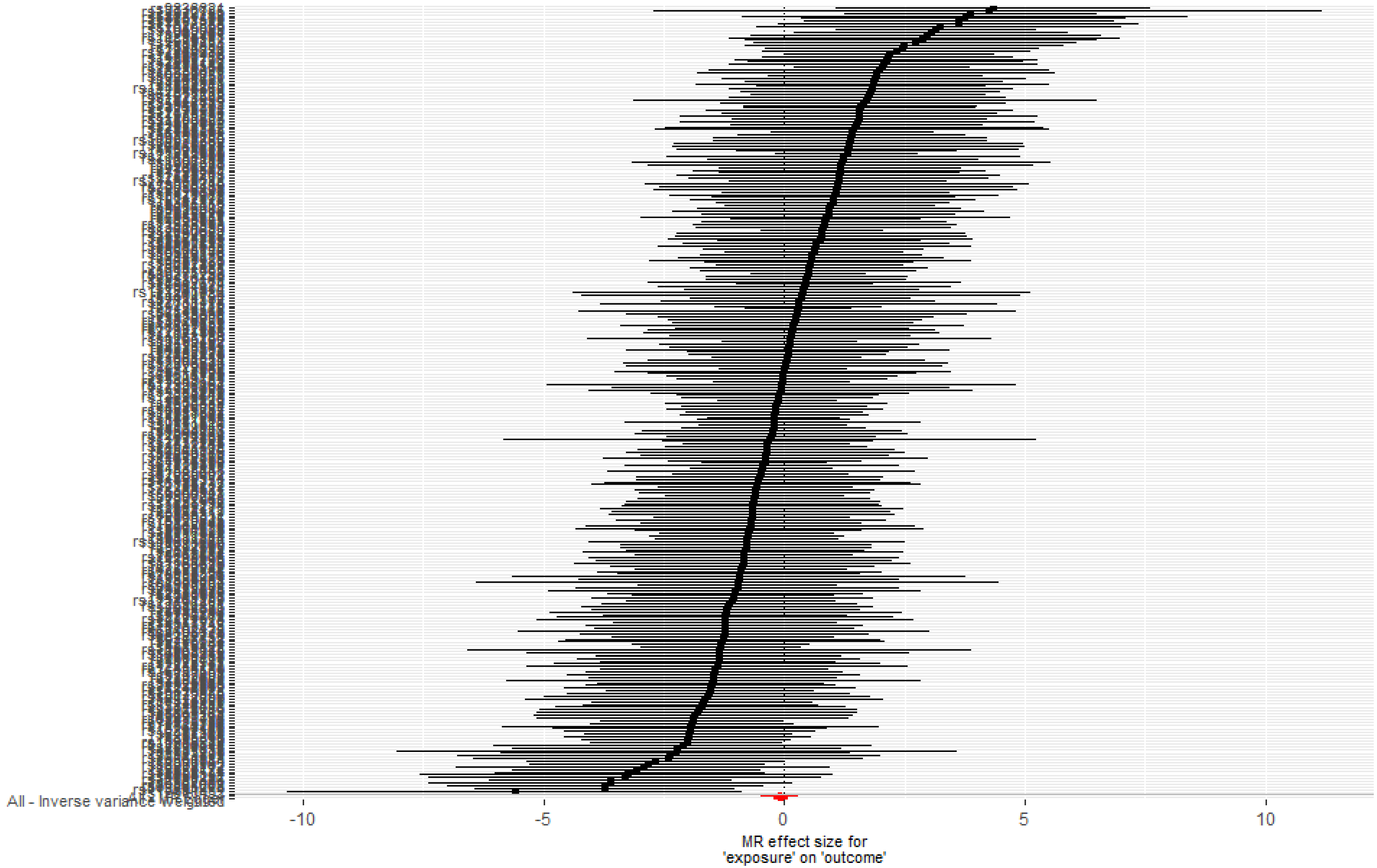

Abbreviations: MR: Mendelian randomization

Supplementary Figure S23. Forest plot of chronotype and rectal cancer association

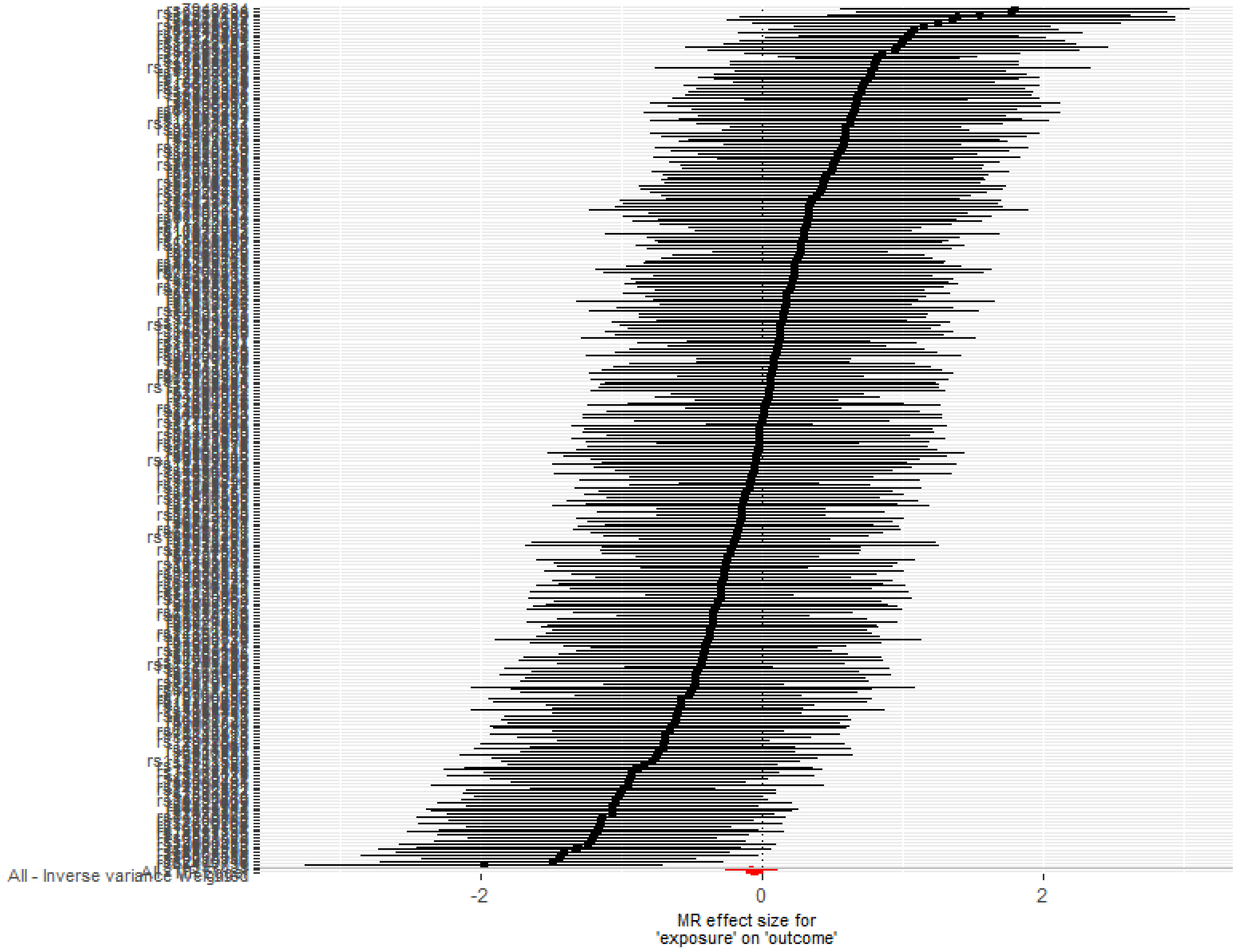

Abbreviations: MR: Mendelian randomization

Supplementary Figure S24. Funnel plot of chronotype and colorectal cancer association in males

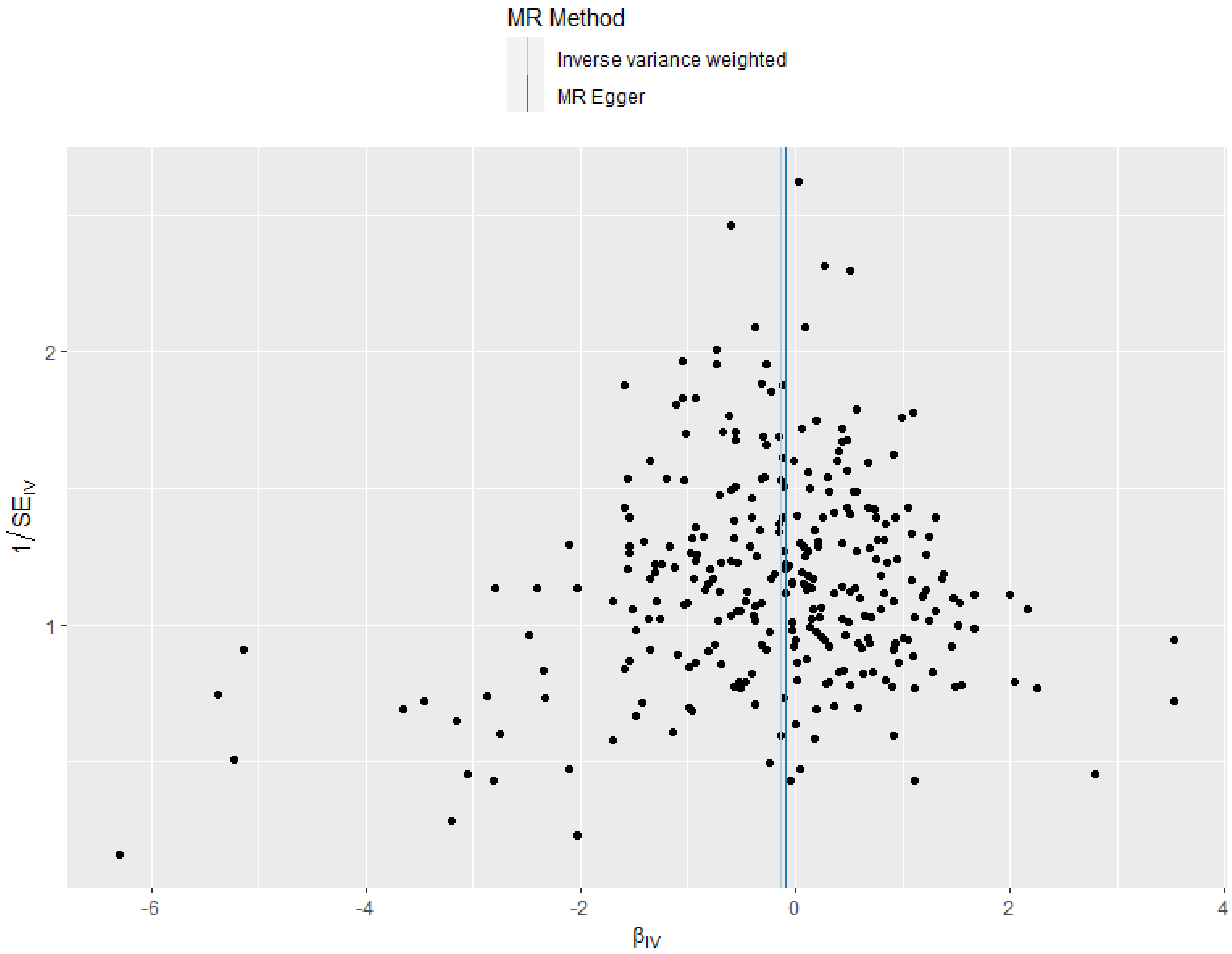

Abbreviations: MR: Mendelian randomization; SE_{IV}: Inverse-variance Standard Error; β_{IV}: Inverse-variance beta coefficient

Supplementary Figure S25. Funnel plot of chronotype and colorectal cancer association in females

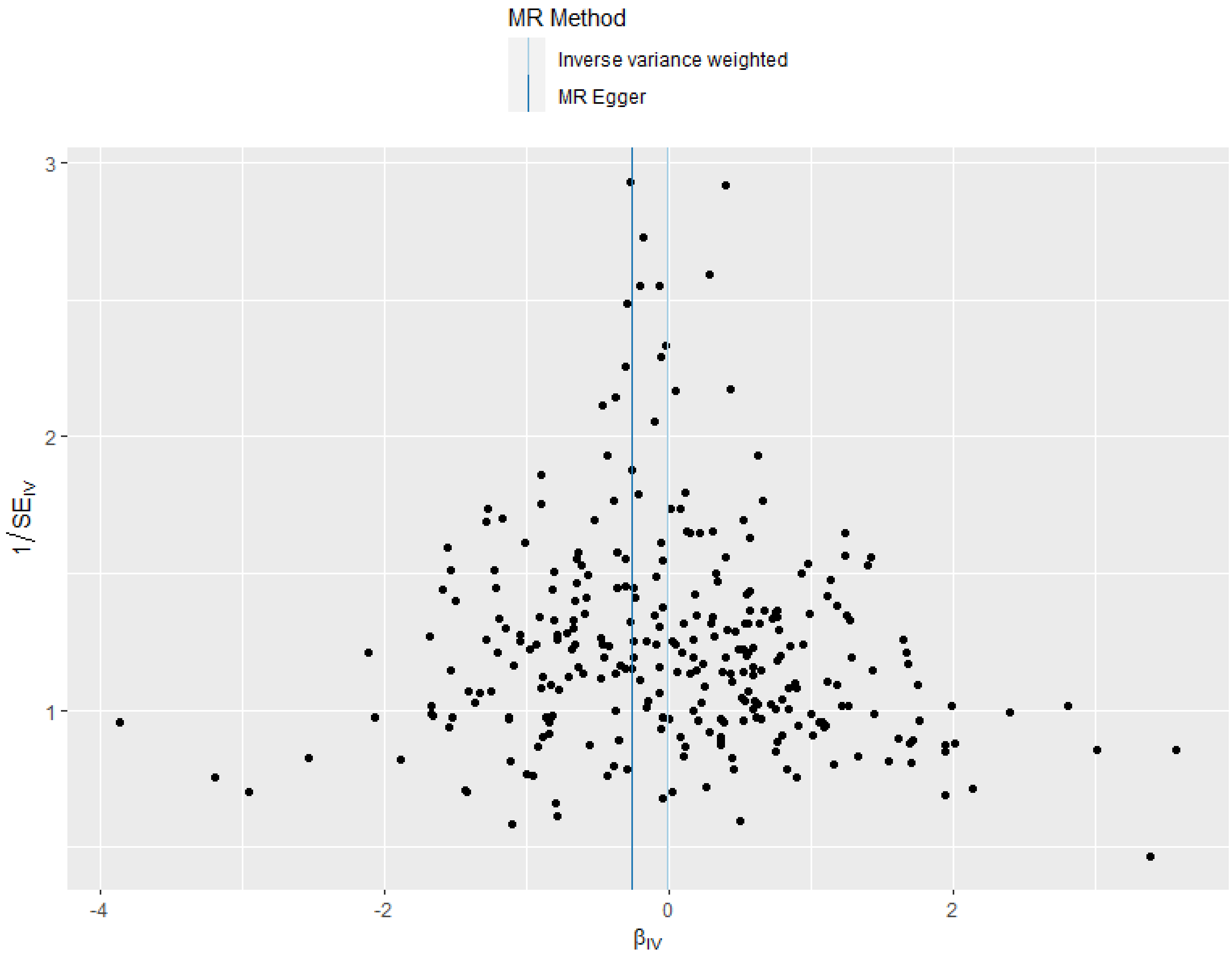

Abbreviations: MR: Mendelian randomization; SE_{IV}: Inverse-variance Standard Error; β_{IV}: Inverse-variance beta coefficient

Supplementary Figure S26. Funnel plot of chronotype and colorectal cancer association

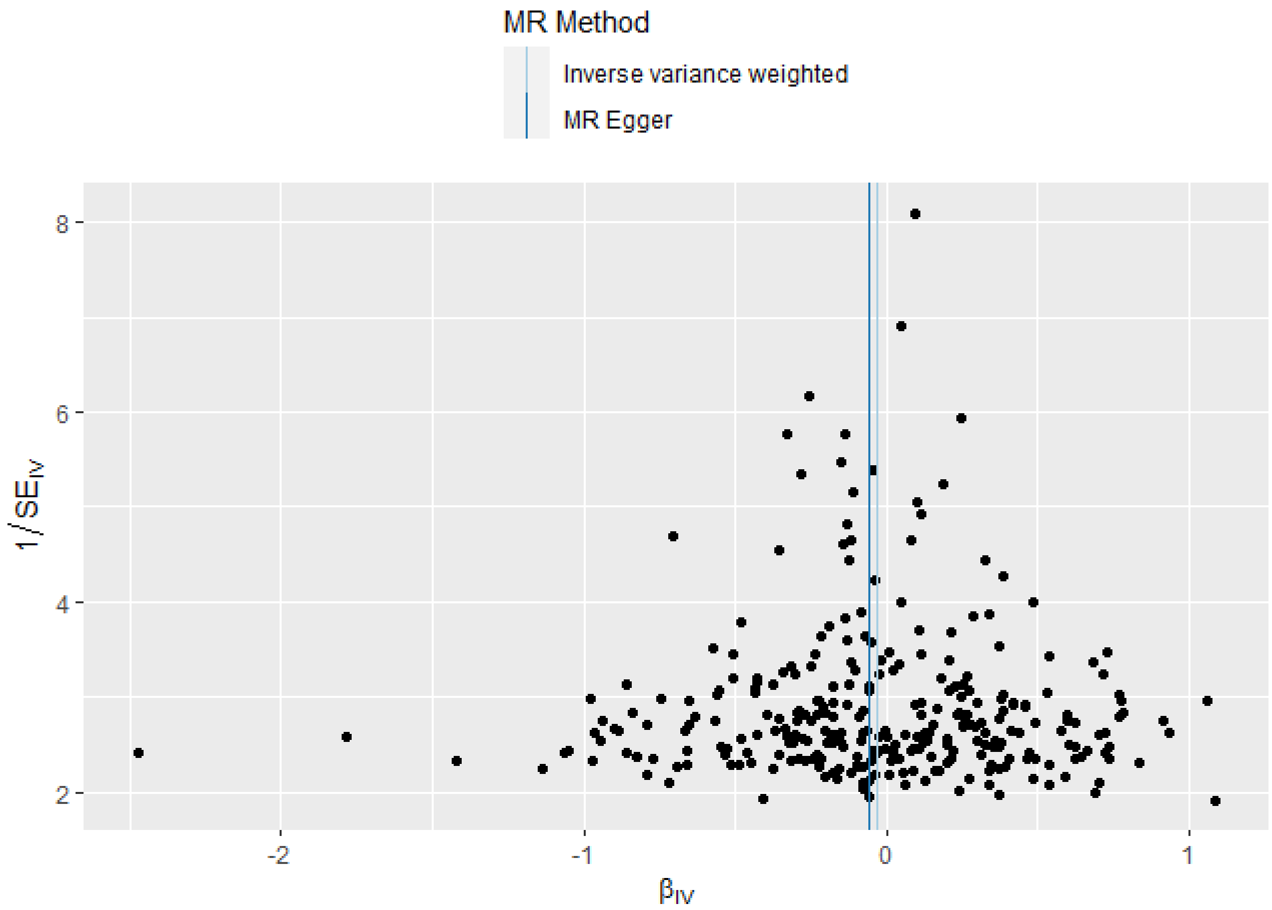

Abbreviations: MR: Mendelian randomization; SE_{IV}: Inverse-variance Standard Error; β_{IV}: Inverse-variance beta coefficient

Supplementary Figure S27. Funnel plot of chronotype and colon cancer association in males

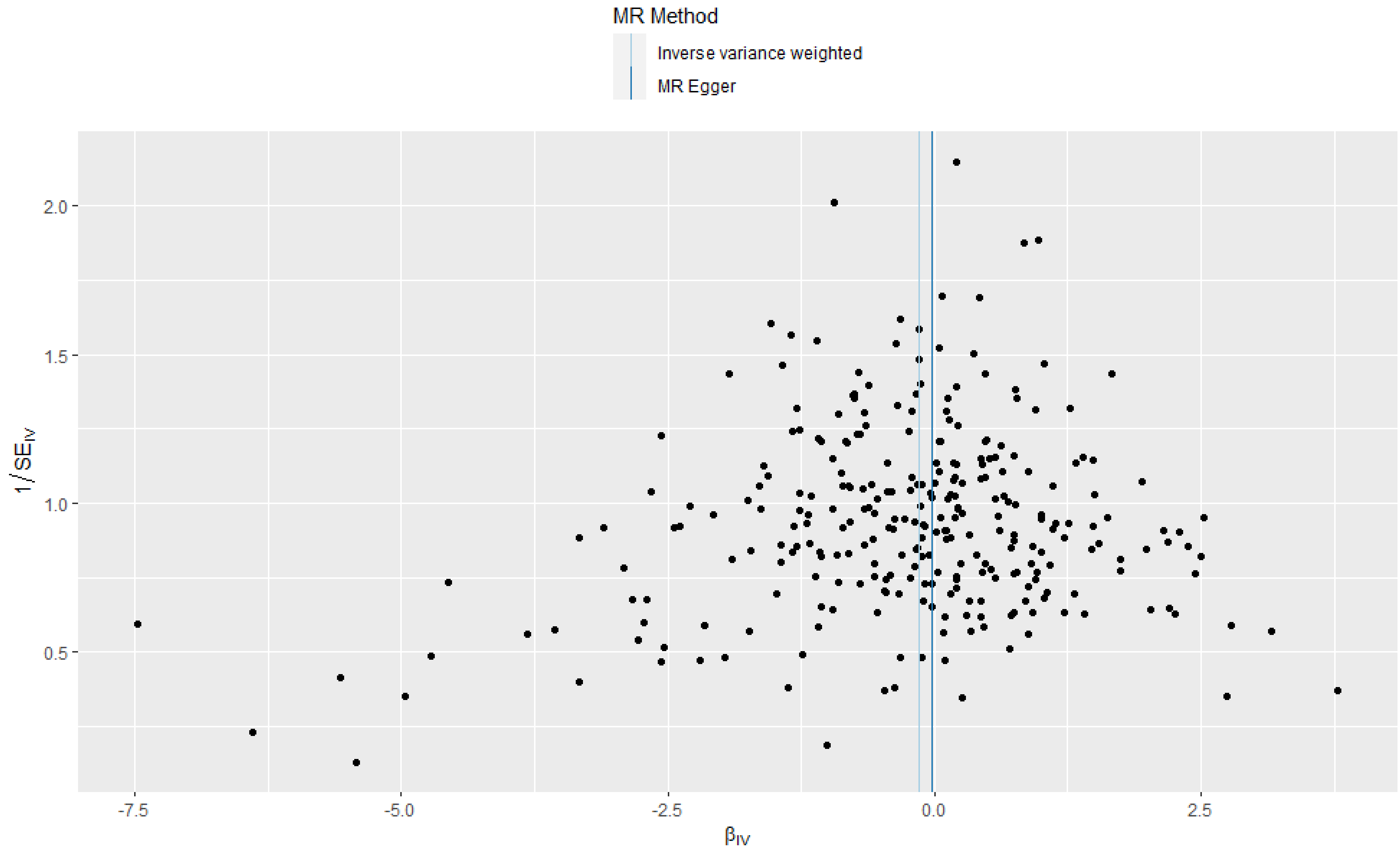

Abbreviations: MR: Mendelian randomization; SE_{IV}: Inverse-variance Standard Error; β_{IV}: Inverse-variance beta coefficient

Supplementary Figure S28. Funnel plot of chronotype and colon cancer association in females

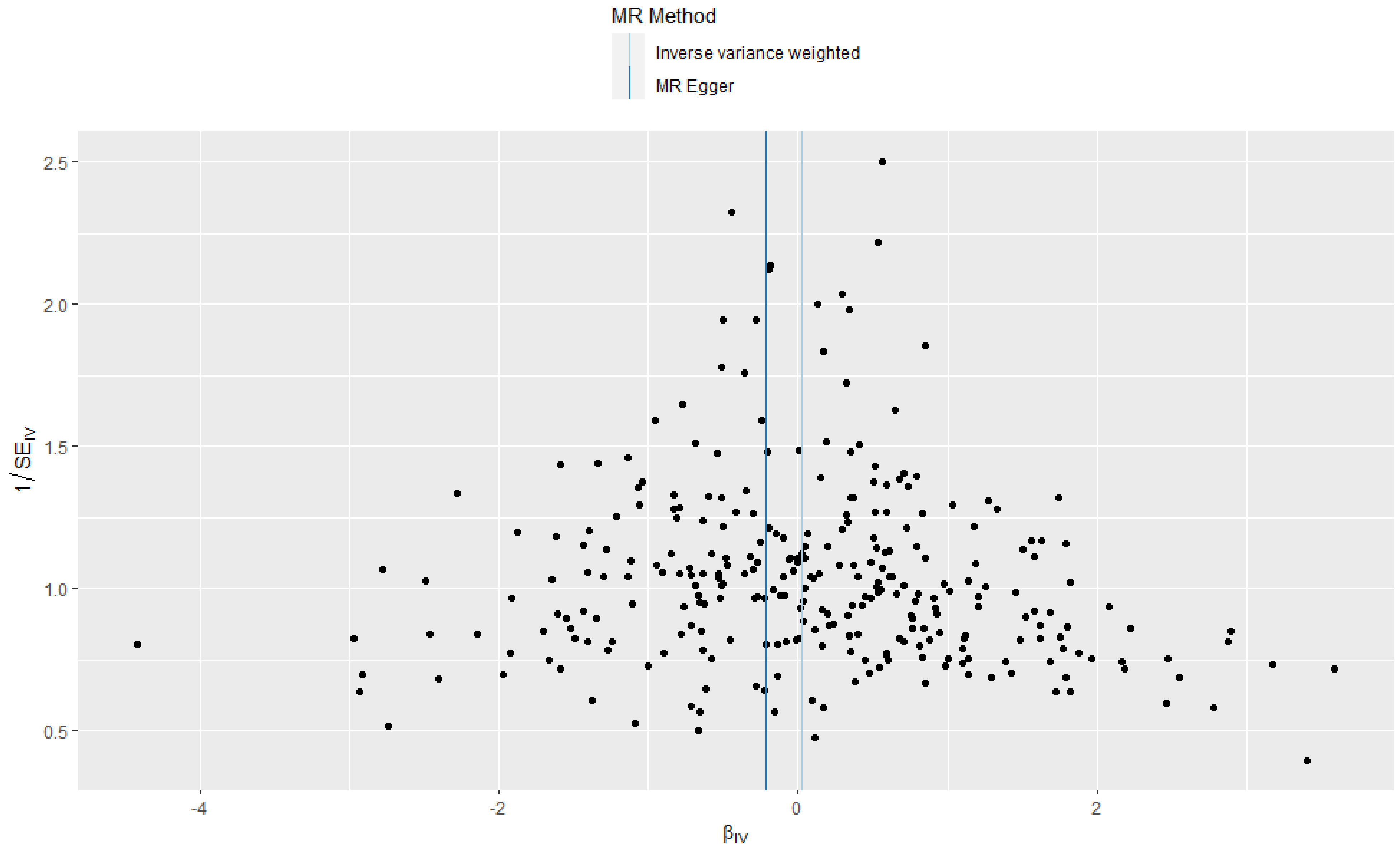

Abbreviations: MR: Mendelian randomization; SE_{IV}: Inverse-variance Standard Error; β_{IV}: Inverse-variance beta coefficient

Supplementary Figure S29. Funnel plot of chronotype and colon cancer association

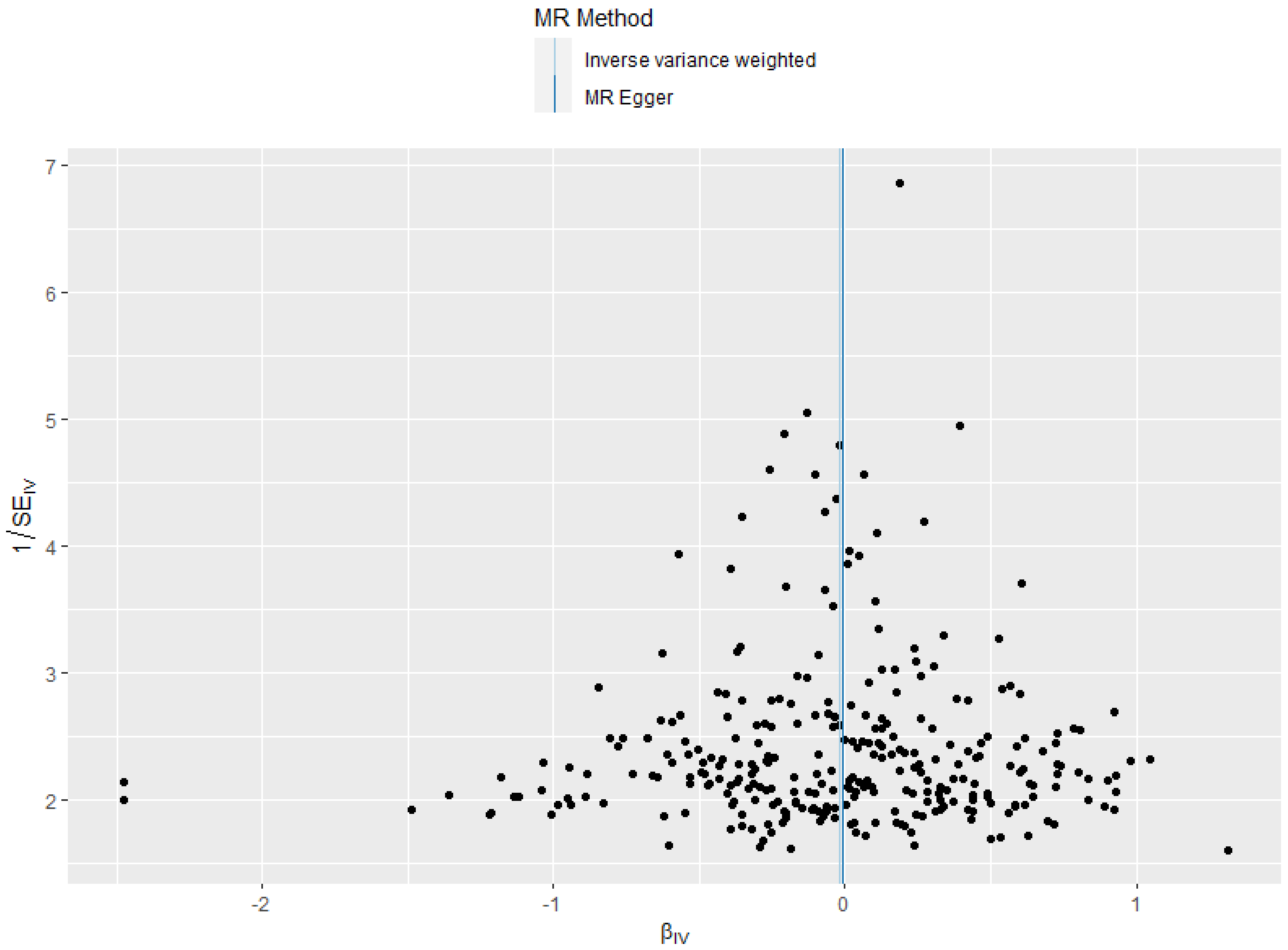

Abbreviations: MR: Mendelian randomization; SE$_{IV}$: Inverse-variance Standard Error; β_{IV}: Inverse-variance beta coefficient

Supplementary Figure S30. Funnel plot of chronotype and proximal colon cancer association

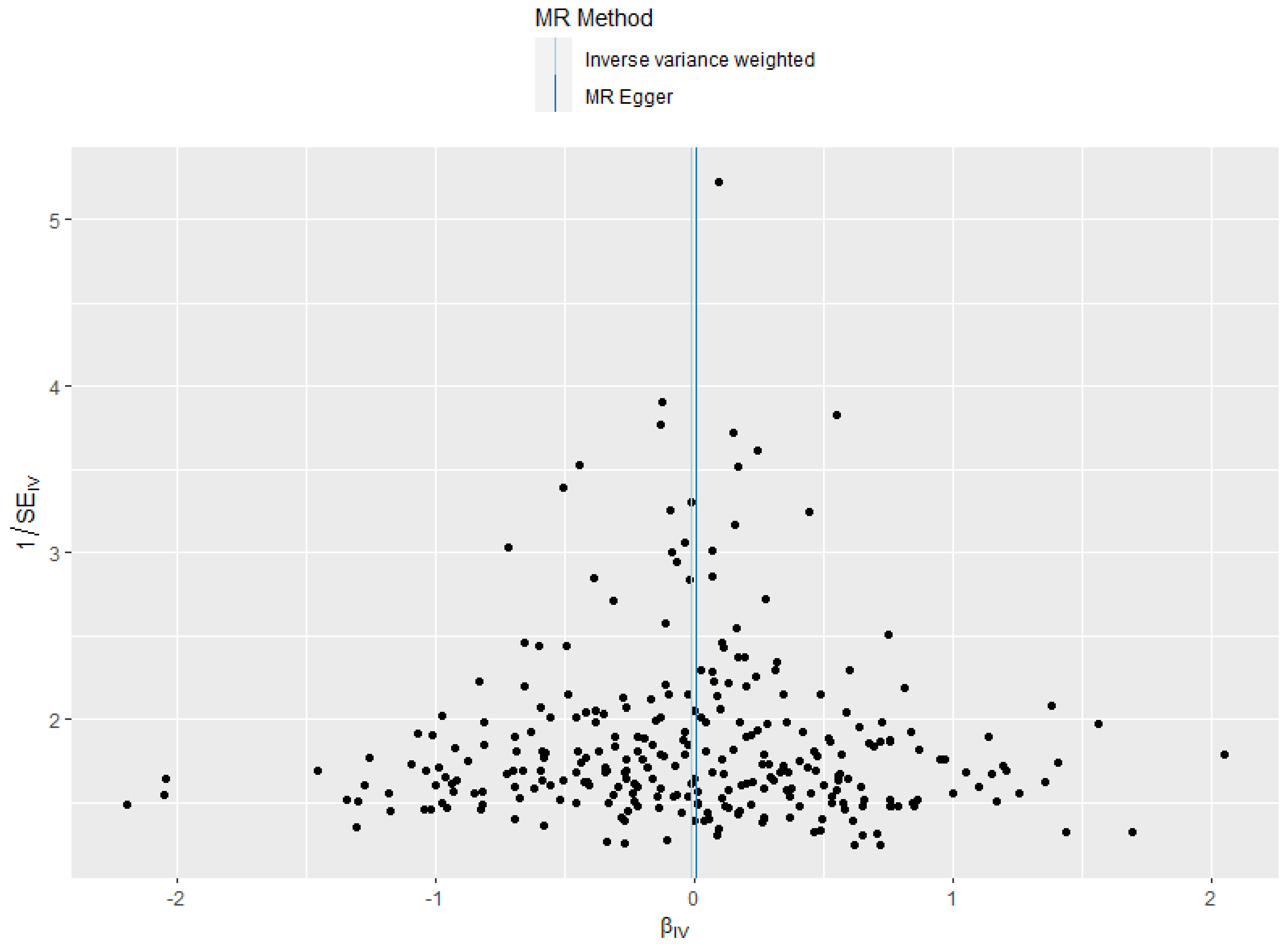

Abbreviations: MR: Mendelian randomization; SE_{IV}: Inverse-variance Standard Error; β_{IV}: Inverse-variance beta coefficient

Supplementary Figure S31. Funnel plot of chronotype and distal colon cancer association

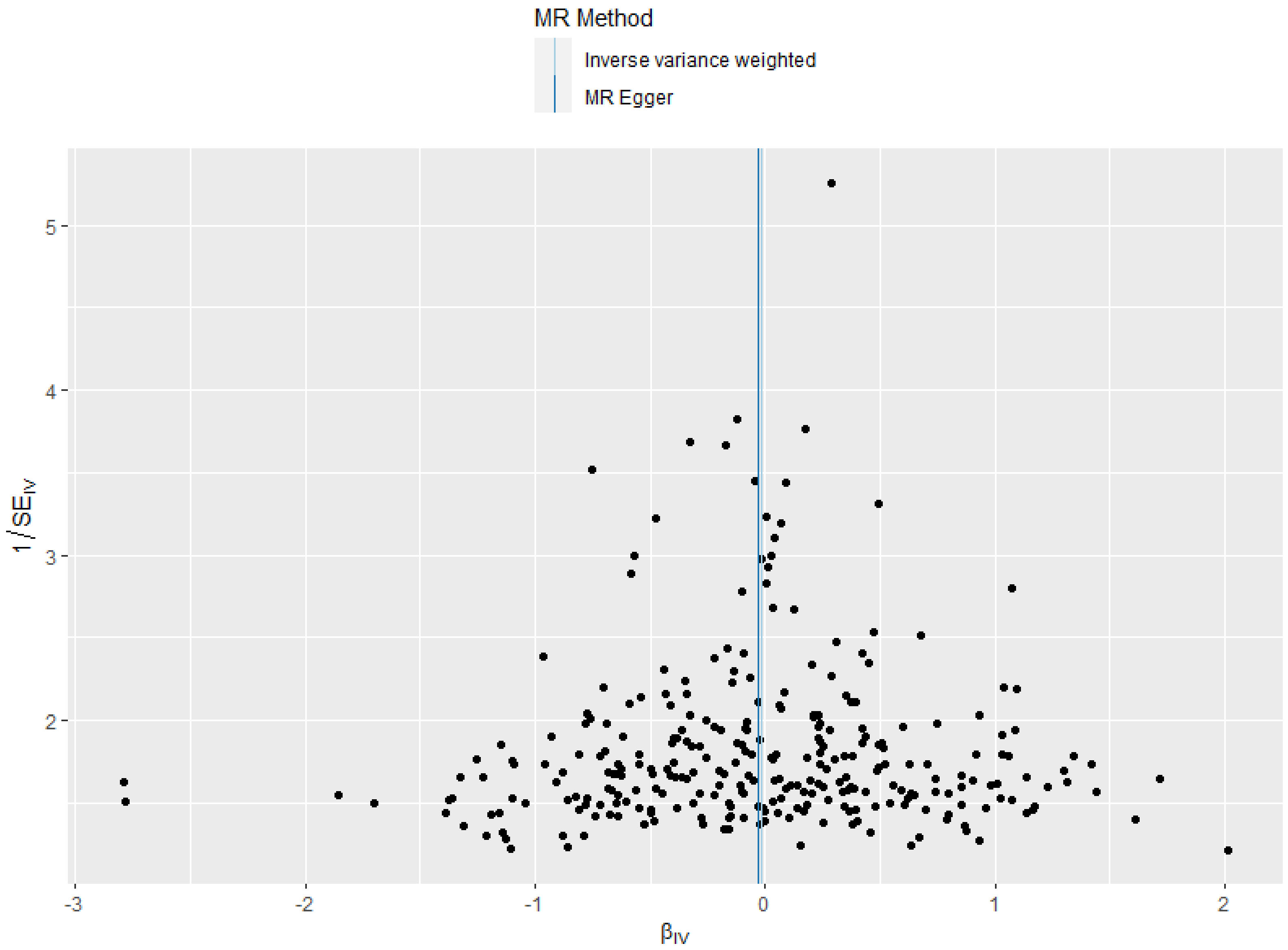

Abbreviations: MR: Mendelian randomization; SE_{IV}: Inverse-variance Standard Error; β_{IV}: Inverse-variance beta coefficient

Supplementary Figure S32. Funnel plot of chronotype and rectal cancer association in males

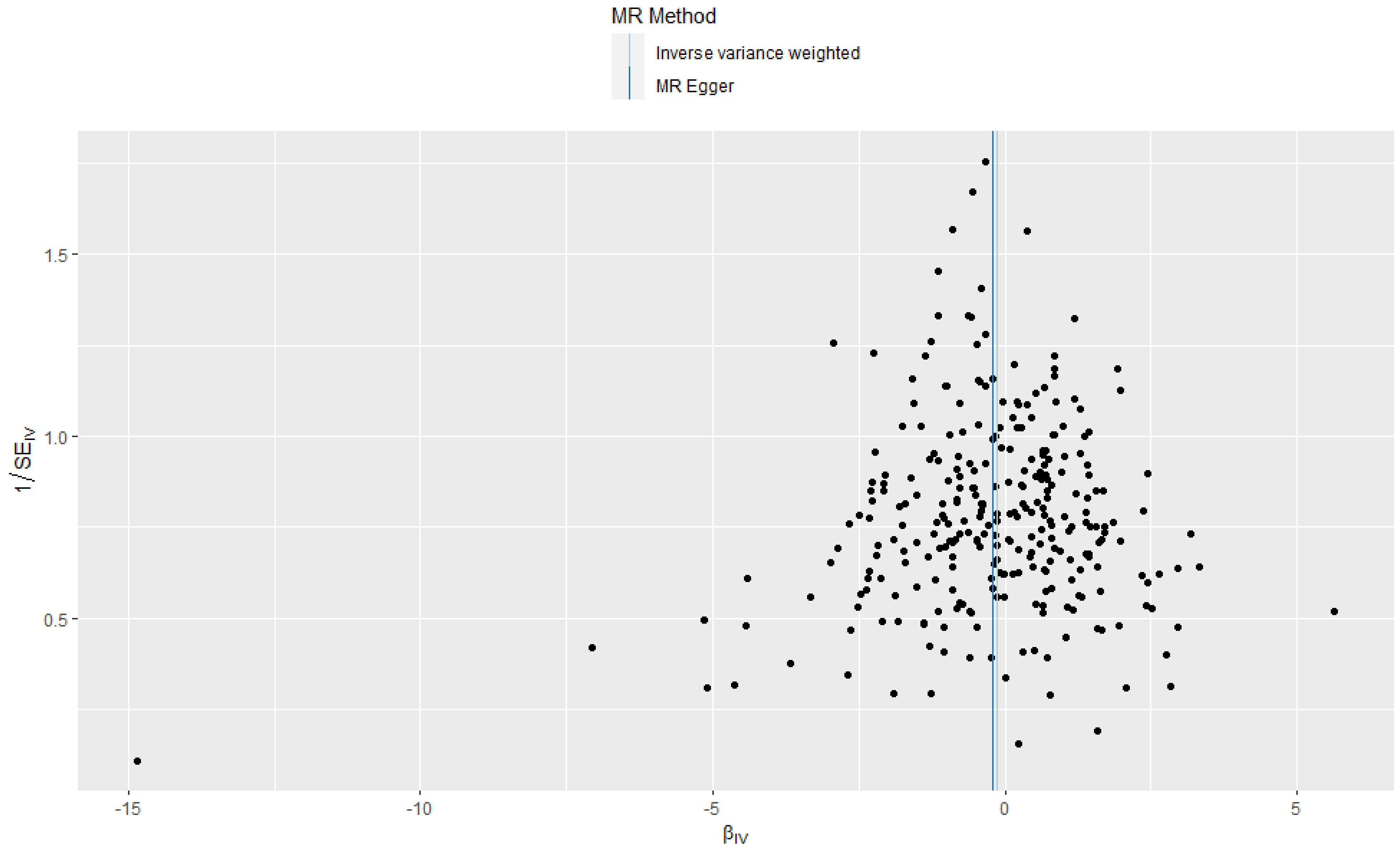

Abbreviations: MR: Mendelian randomization; SE_{IV}: Inverse-variance Standard Error; β_{IV}: Inverse-variance beta coefficient

Supplementary Figure S33. Funnel plot of chronotype and rectal cancer association in females

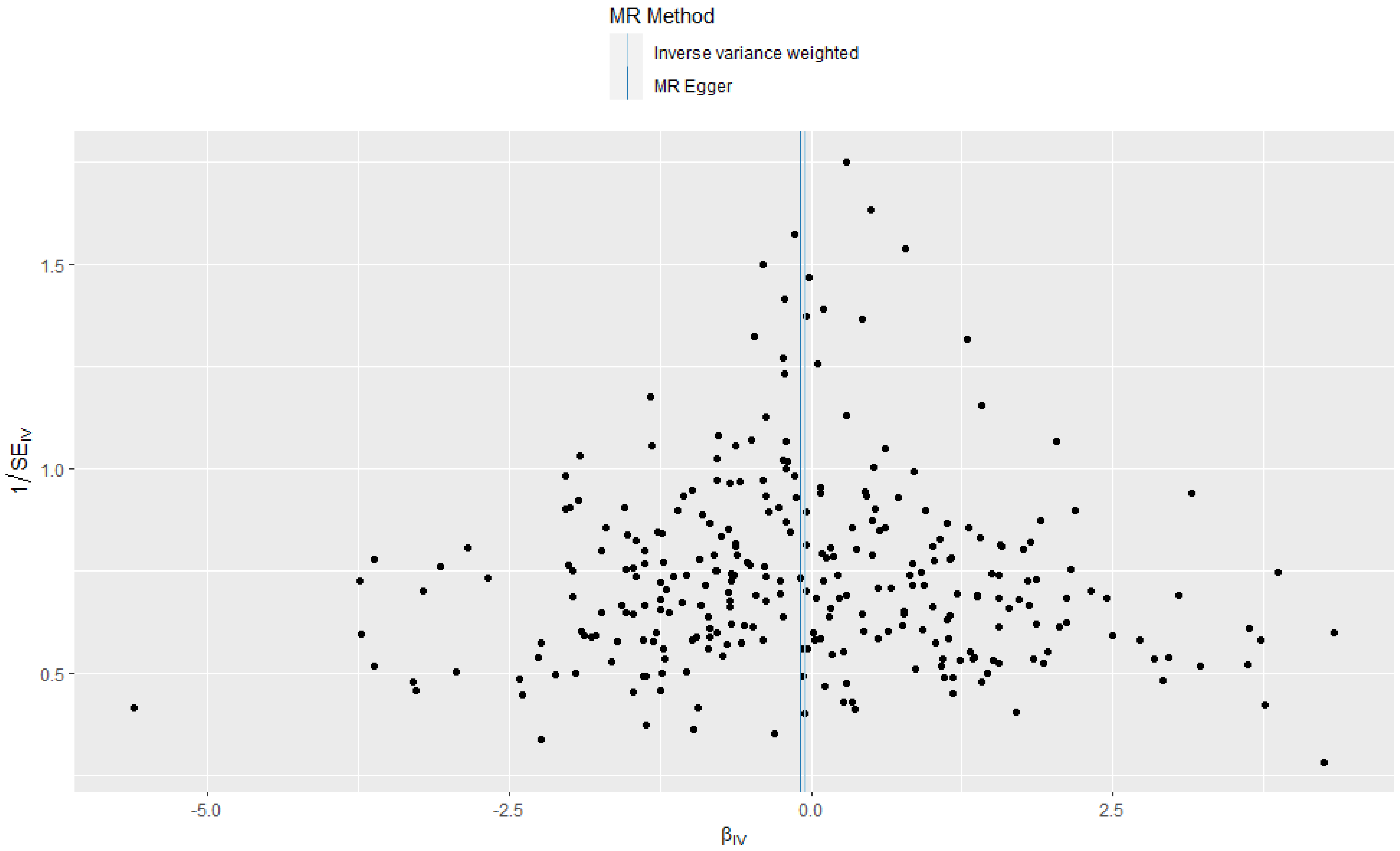

Abbreviations: MR: Mendelian randomization; SE_{IV}: Inverse-variance Standard Error; β_{IV}: Inverse-variance beta coefficient

Supplementary Figure S34. Funnel plot of chronotype and rectal cancer association

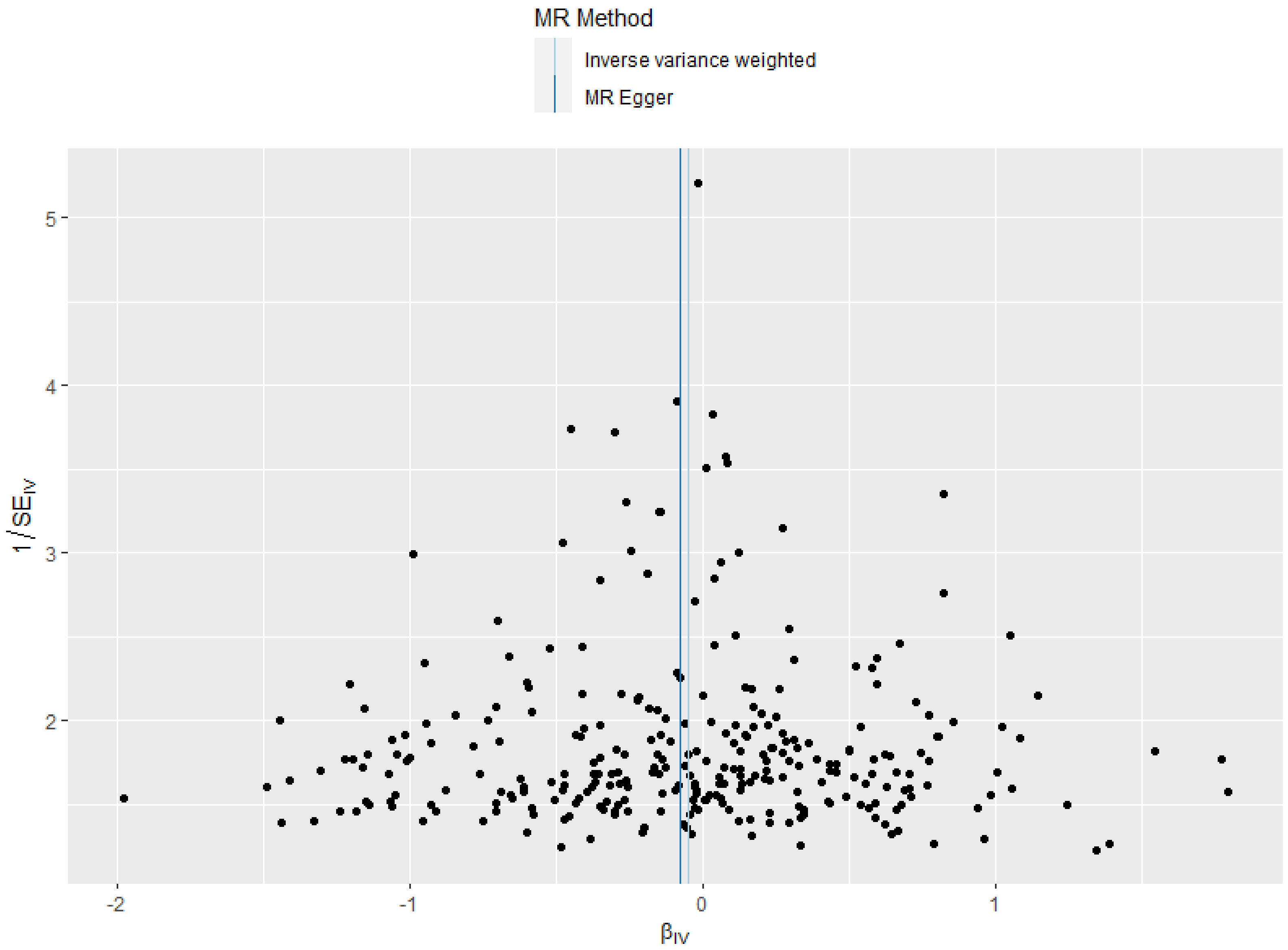

Abbreviations: MR: Mendelian randomization; SE_{IV}: Inverse-variance Standard Error; β_{IV}: Inverse-variance beta coefficient

Primary MR analyses: Frequent insomnia symptoms [Lane *et al.* (2019)]

Supplementary Figure S35. Scatter plot of frequent insomnia symptoms [Lane *et al.* (2019)] and colorectal cancer association in males

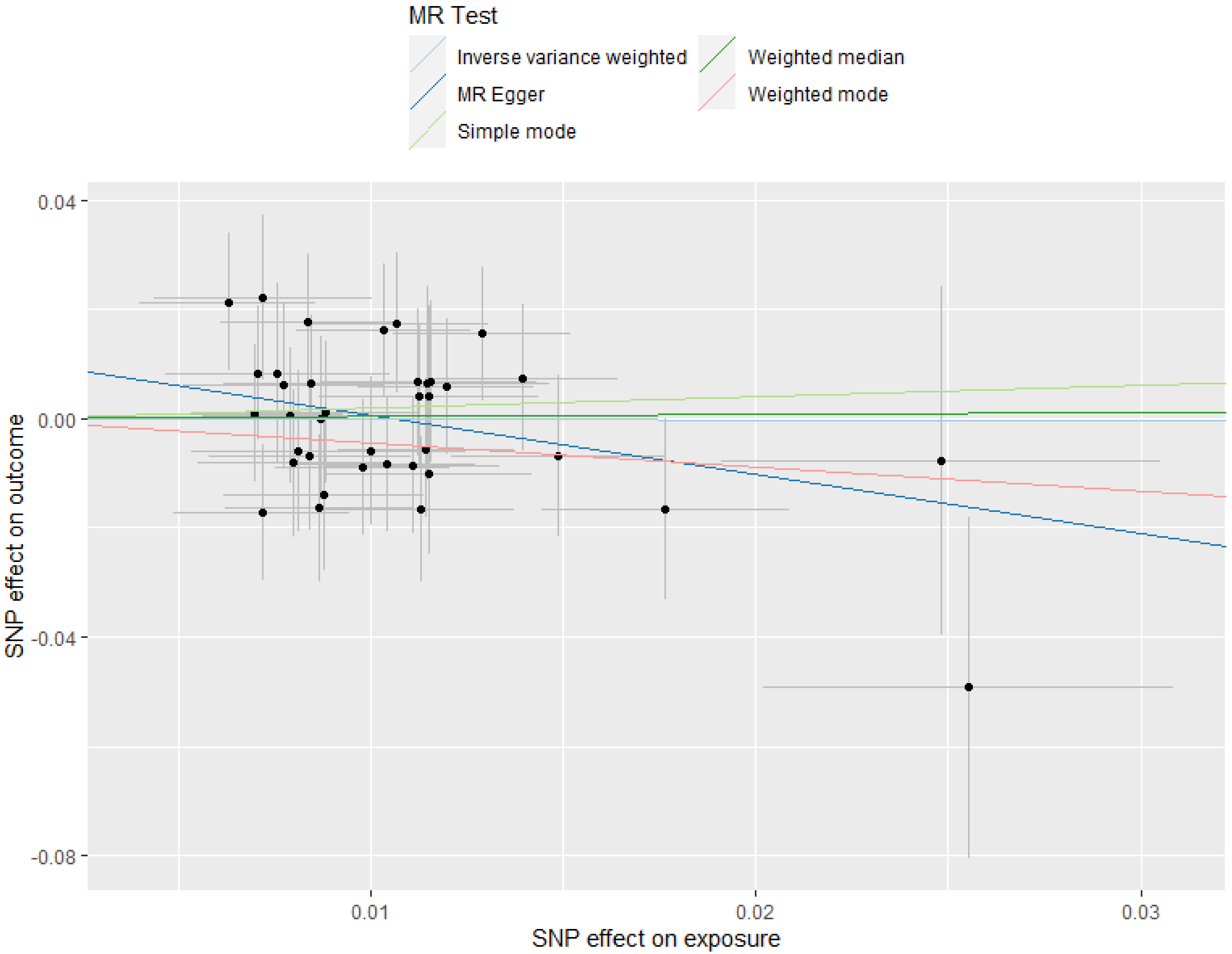

Abbreviations: MR: Mendelian randomization; SNP: Single Nucleotide Polymorphism

Supplementary Figure S36. Scatter plot of frequent insomnia symptoms [Lane *et al.* (2019)] and colorectal cancer association in females

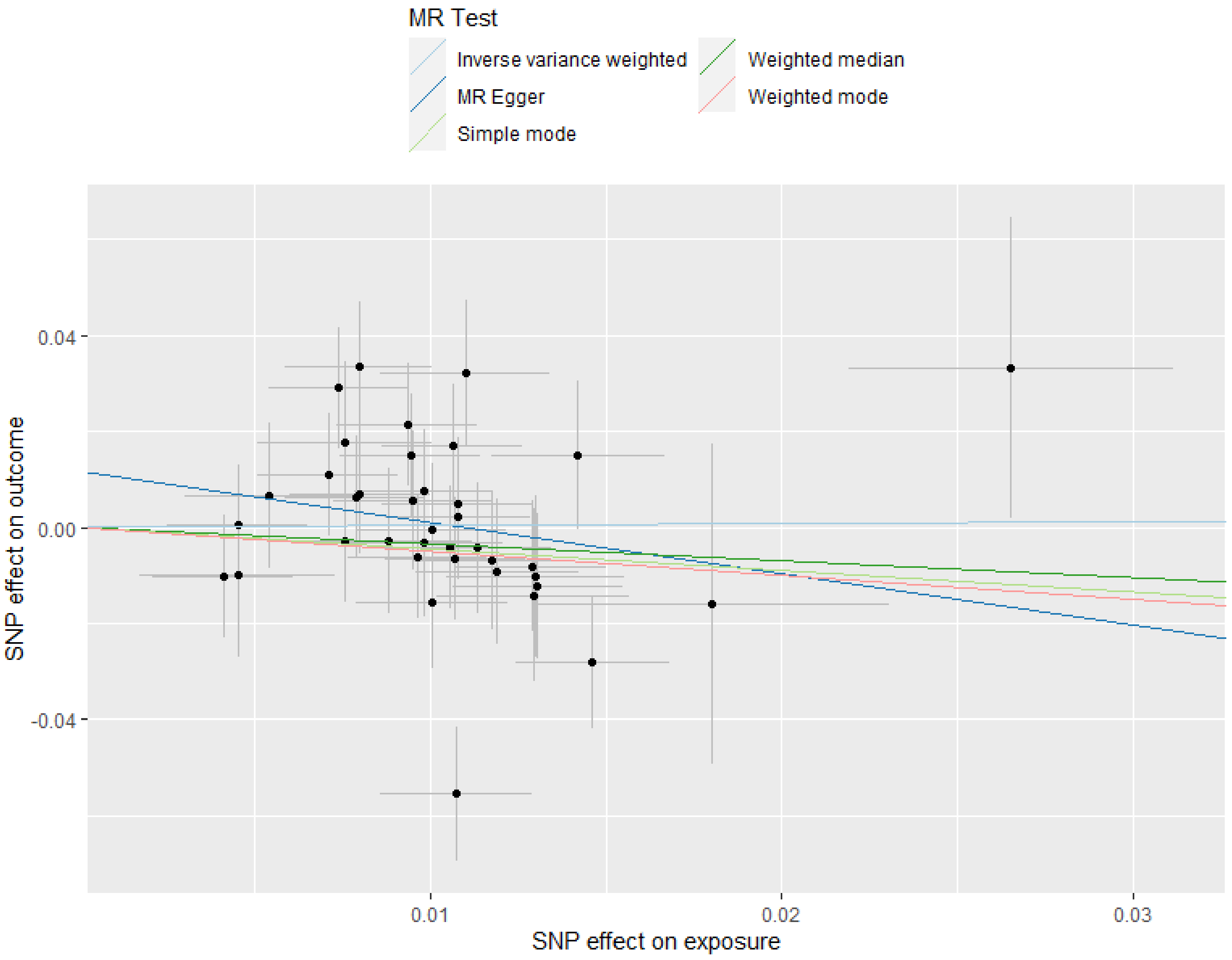

Abbreviations: MR: Mendelian randomization; SNP: Single Nucleotide Polymorphism

Supplementary Figure S37. Scatter plot of frequent insomnia symptoms [Lane *et al.* (2019)] and colorectal cancer association

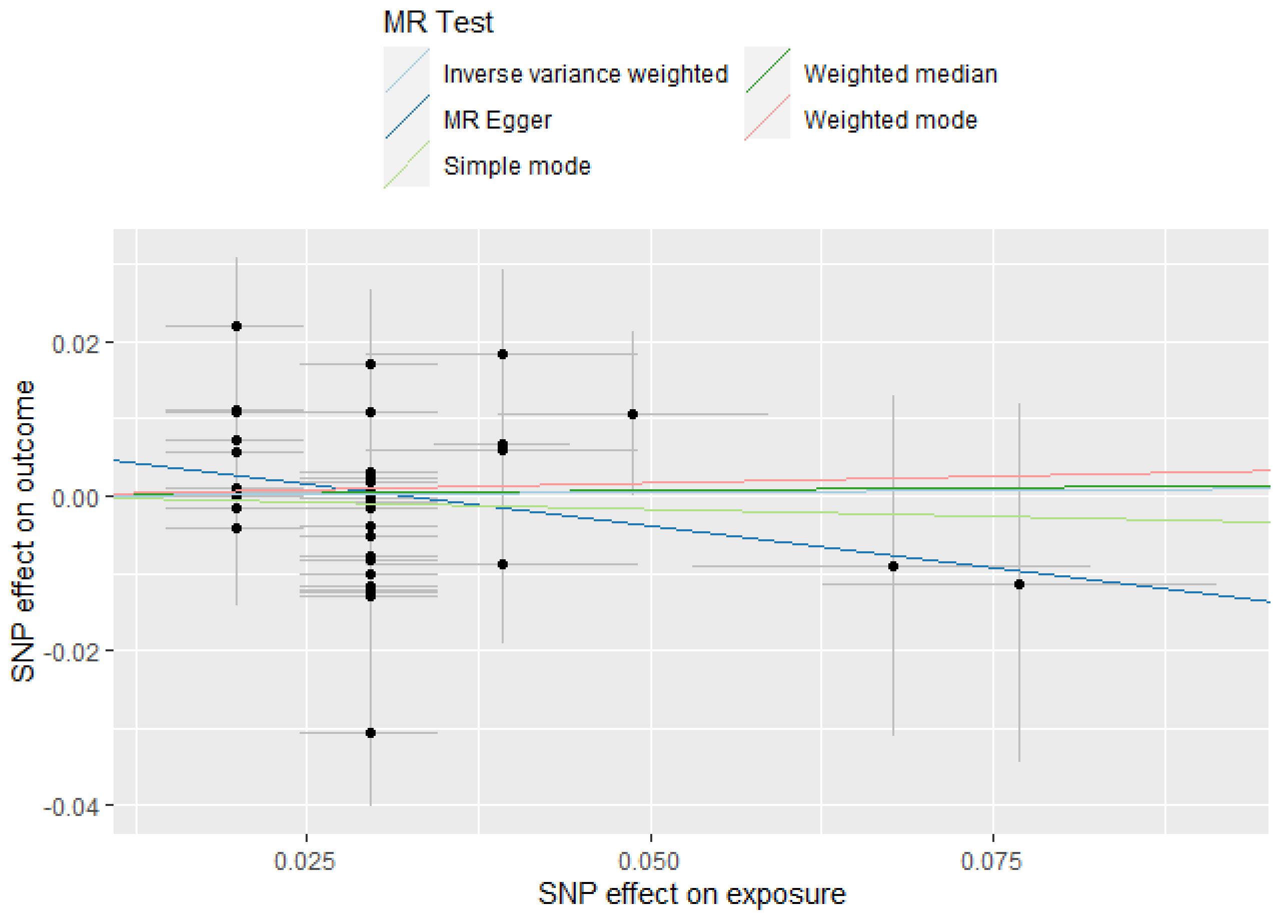

Abbreviations: MR: Mendelian randomization; SNP: Single Nucleotide Polymorphism

Supplementary Figure S38. Scatter plot of frequent insomnia symptoms [Lane *et al.* (2019)] and colon cancer association in males

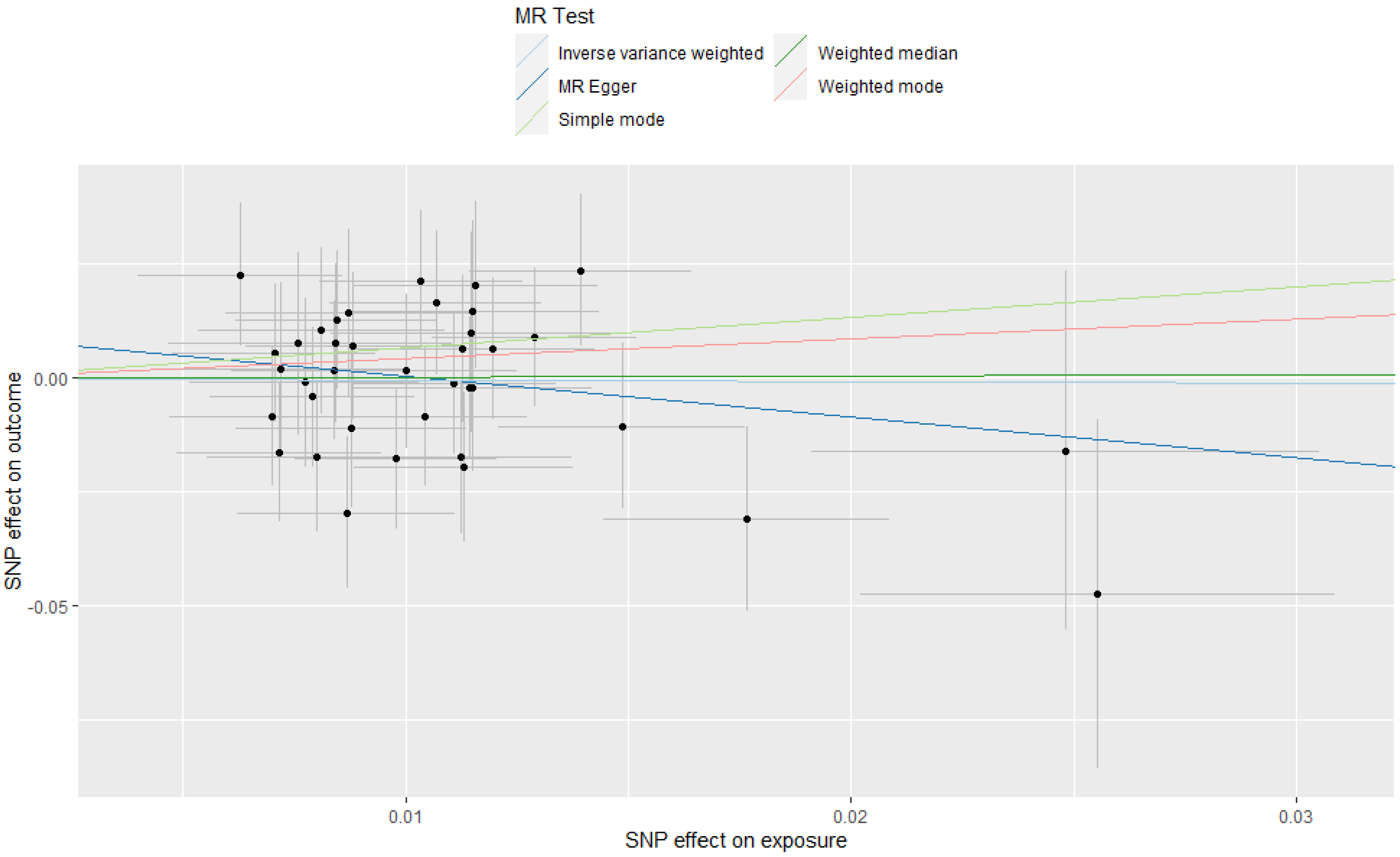

Abbreviations: MR: Mendelian randomization; SNP: Single Nucleotide Polymorphism

Supplementary Figure S39. Scatter plot of frequent insomnia symptoms [Lane *et al.* (2019)] and colon cancer association in females

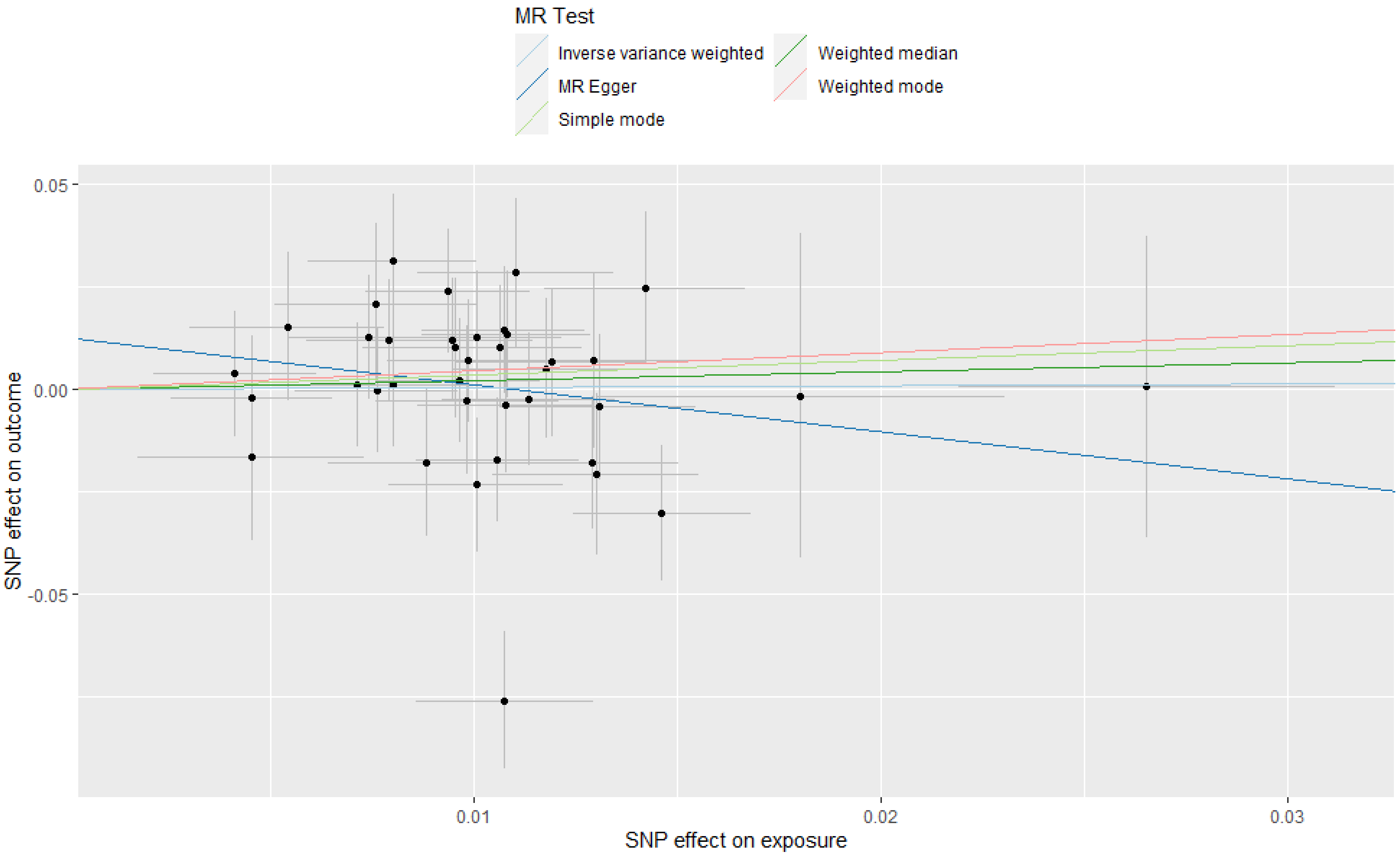

Abbreviations: MR: Mendelian randomization; SNP: Single Nucleotide Polymorphism

Supplementary Figure S40. Scatter plot of frequent insomnia symptoms [Lane *et al.* (2019)] and colon cancer association

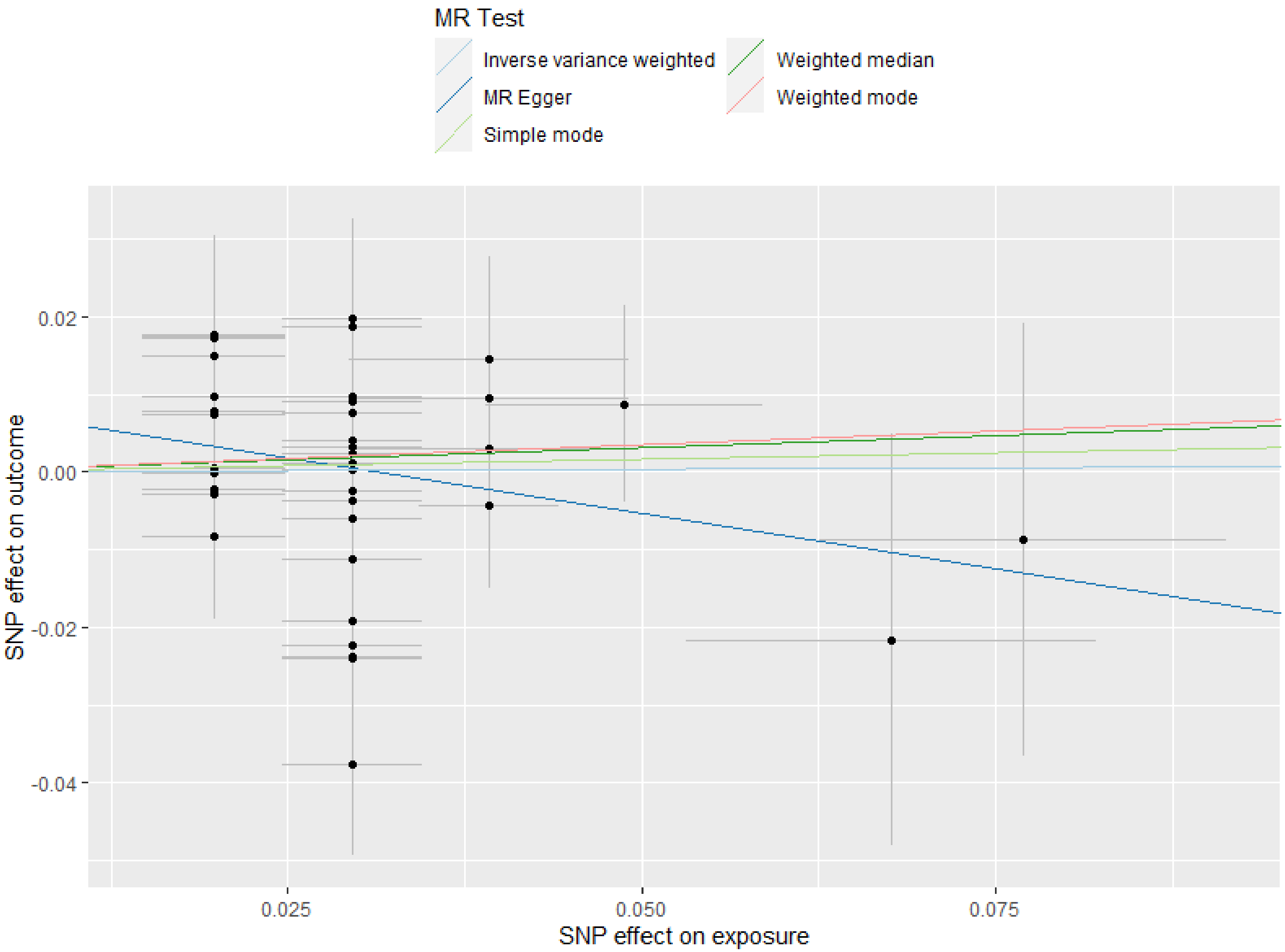

Abbreviations: MR: Mendelian randomization; SNP: Single Nucleotide Polymorphism

Supplementary Figure S41. Scatter plot of frequent insomnia symptoms [Lane *et al.* (2019)] and proximal colon cancer association

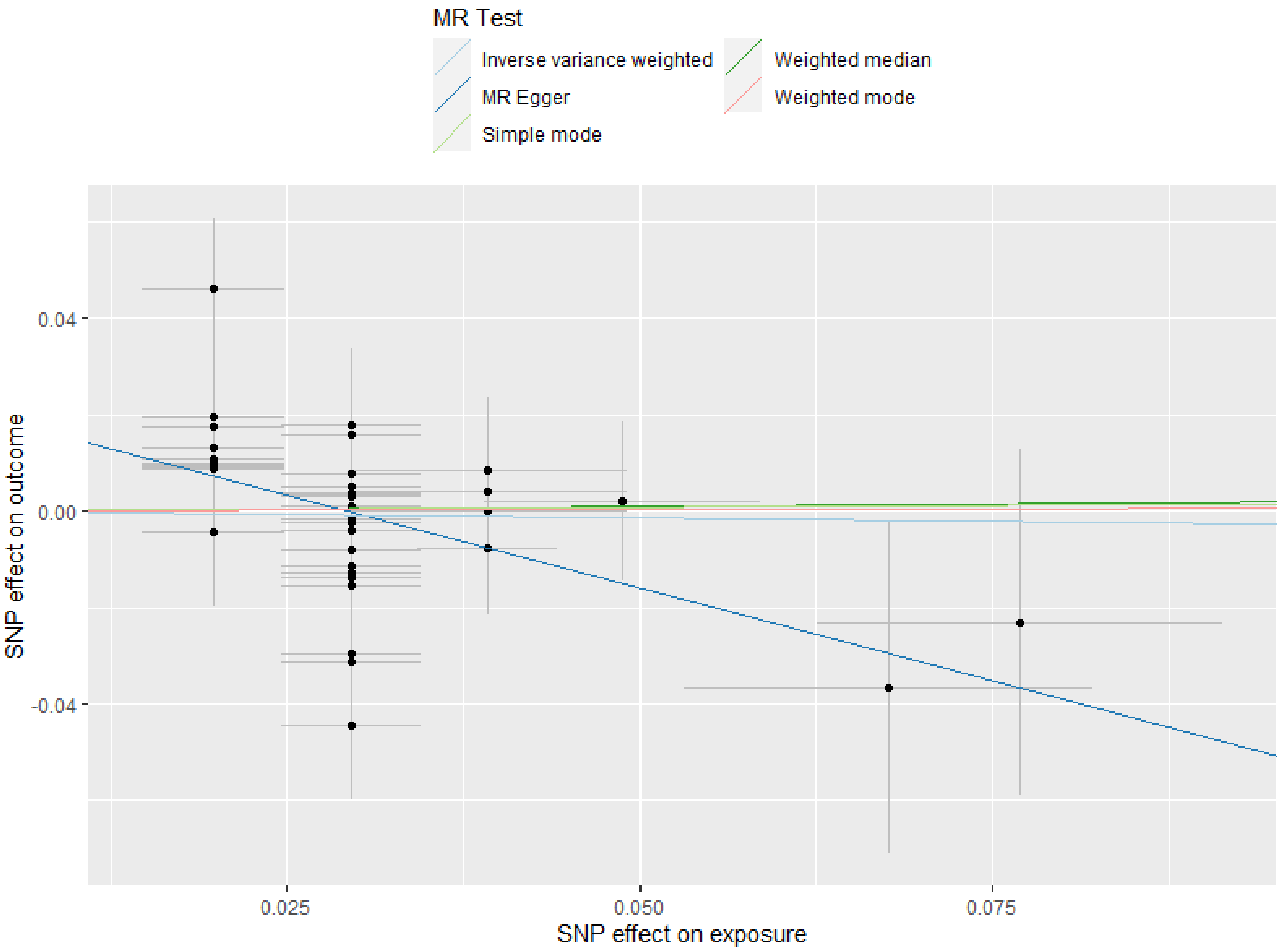

Abbreviations: MR: Mendelian randomization; SNP: Single Nucleotide Polymorphism

Supplementary Figure S42. Scatter plot of frequent insomnia symptoms [Lane *et al.* (2019)] and distal colon cancer association

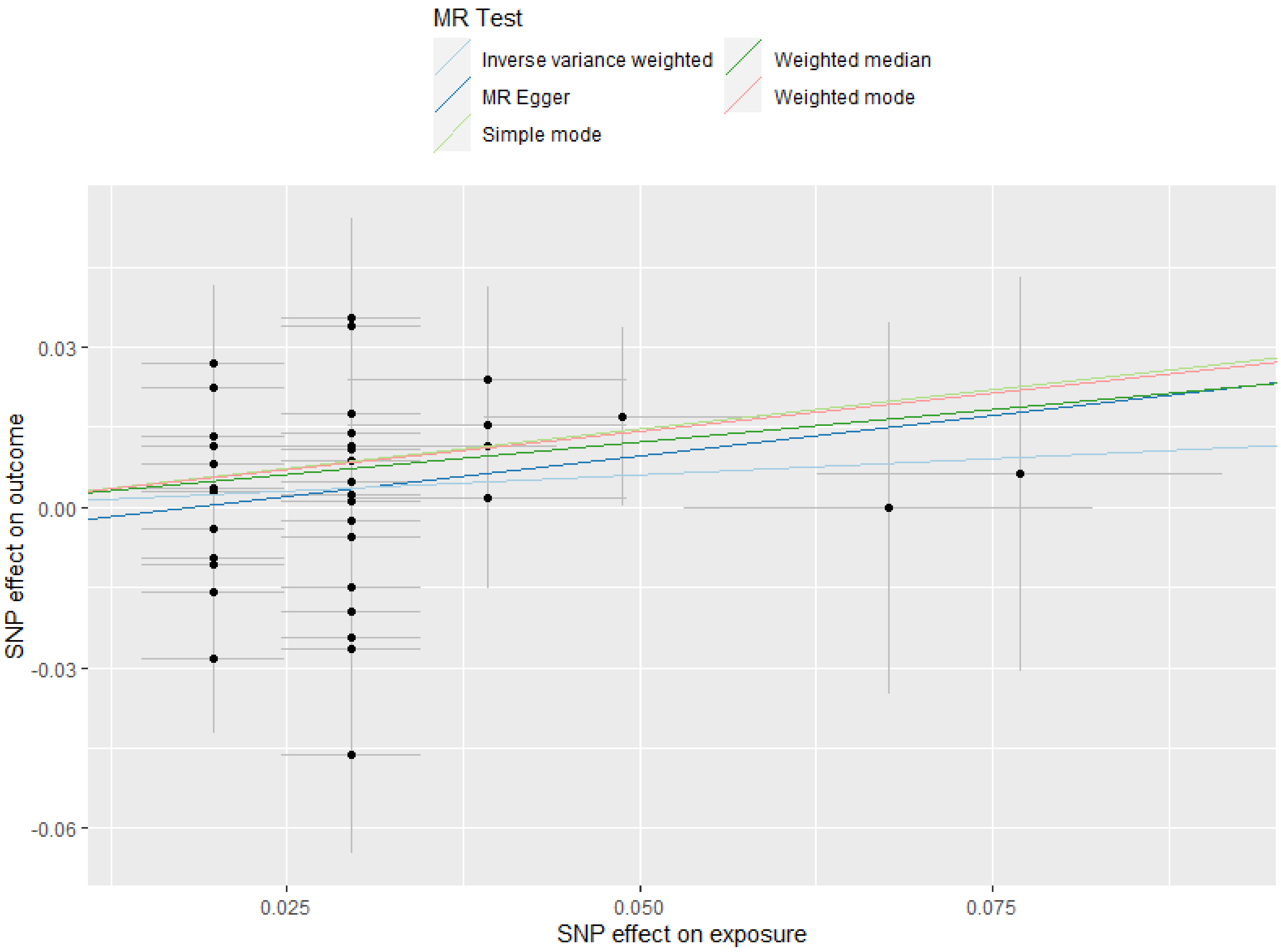

Abbreviations: MR: Mendelian randomization; SNP: Single Nucleotide Polymorphism

Supplementary Figure S43. Scatter plot of frequent insomnia symptoms [Lane *et al.* (2019)] and rectal cancer association in males

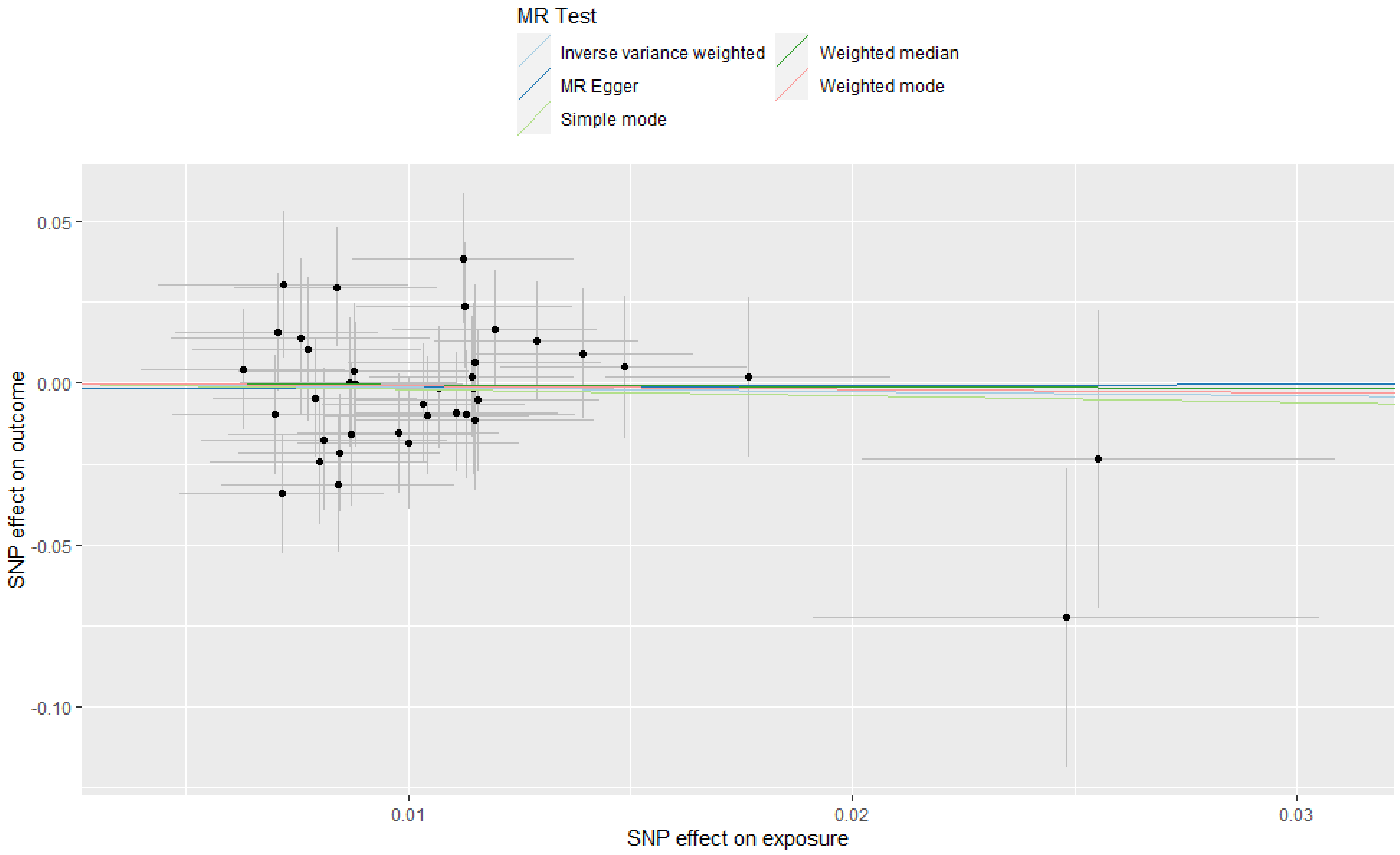

Abbreviations: MR: Mendelian randomization; SNP: Single Nucleotide Polymorphism

Supplementary Figure S44. Scatter plot of frequent insomnia symptoms [Lane *et al.* (2019)] and rectal cancer association in females

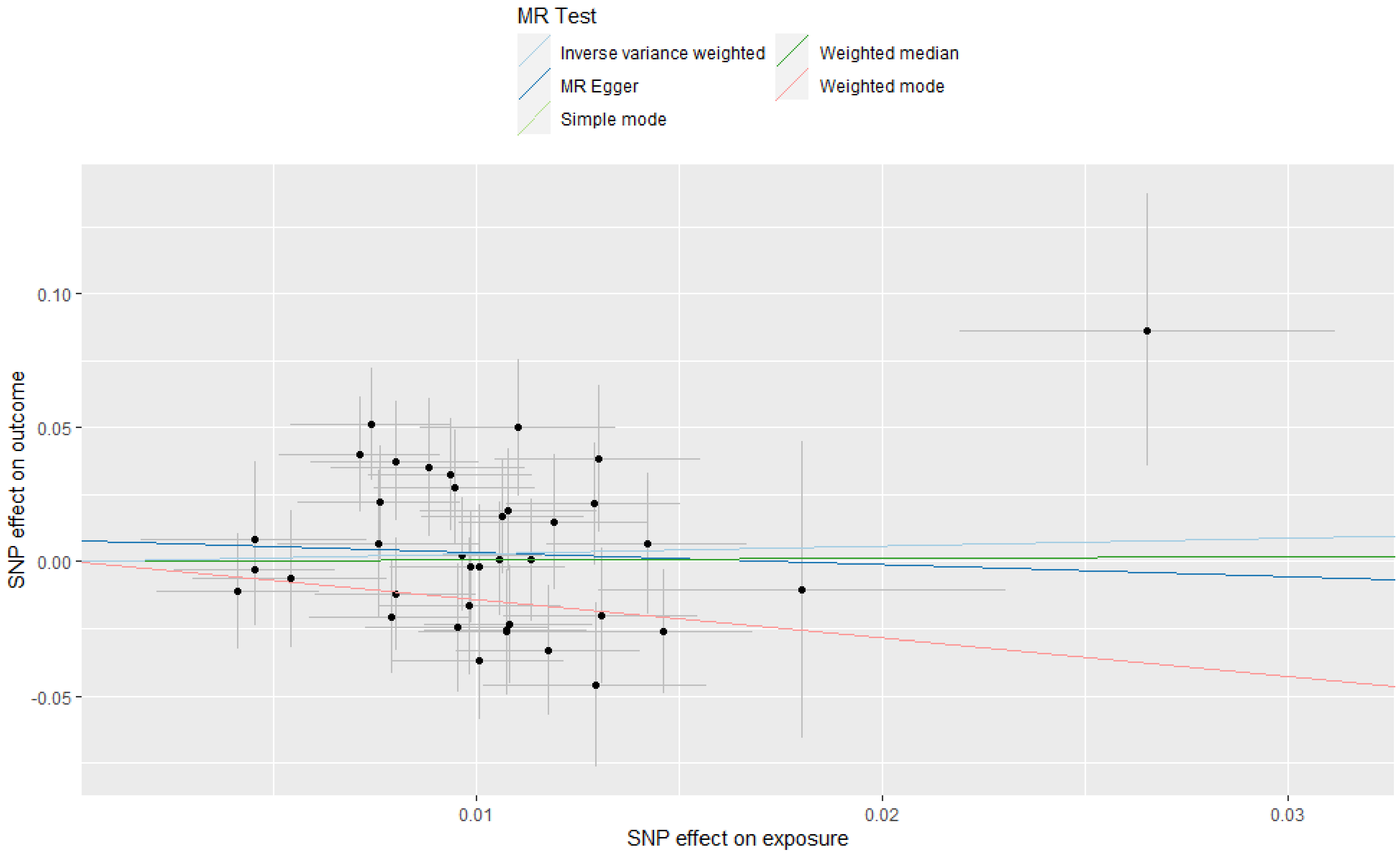

Abbreviations: MR: Mendelian randomization; SNP: Single Nucleotide Polymorphism

Supplementary Figure S45. Scatter plot of frequent insomnia symptoms [Lane *et al.* (2019)] and rectal cancer association

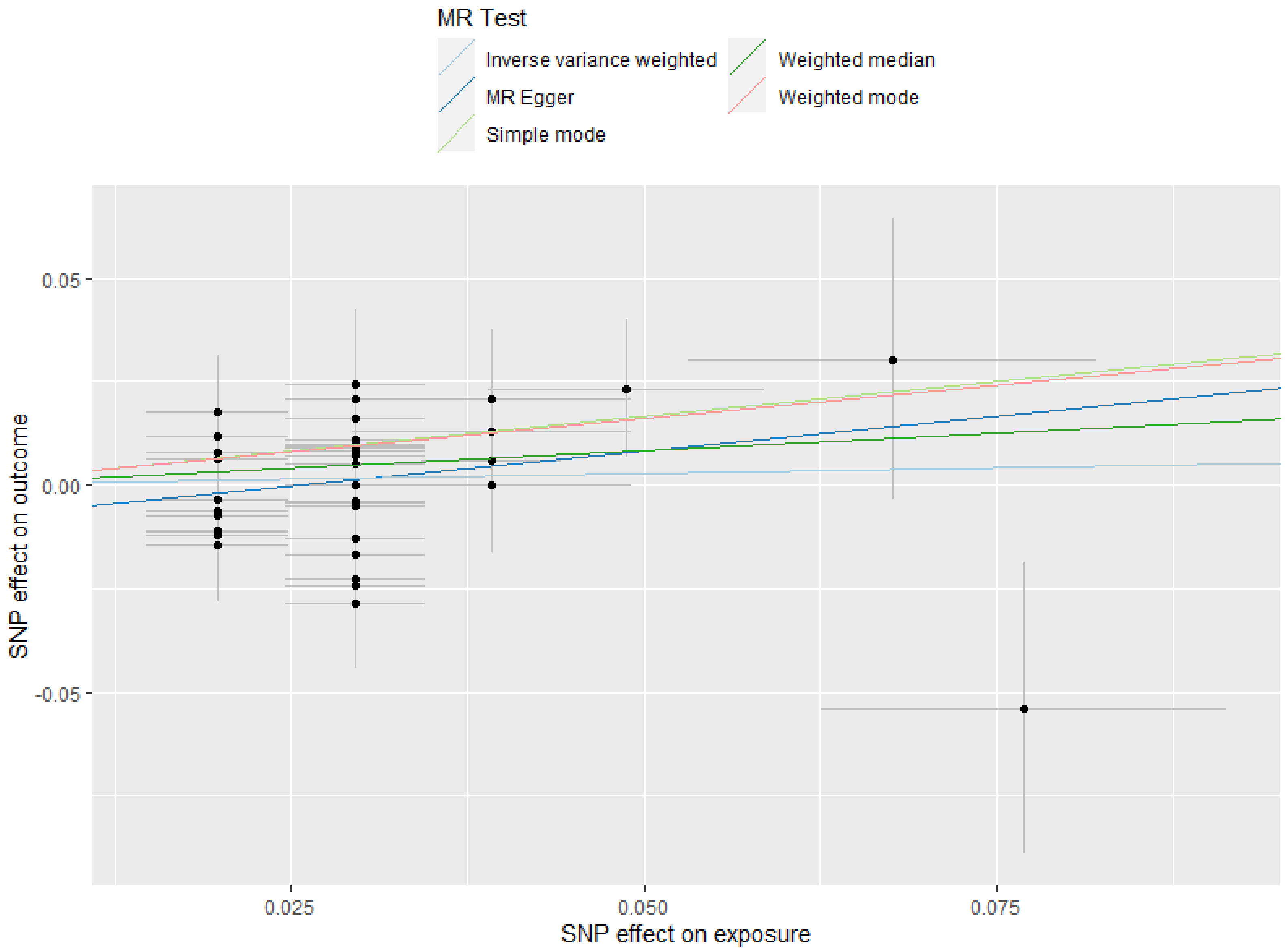

Abbreviations: MR: Mendelian randomization; SNP: Single Nucleotide Polymorphism

Supplementary Figure S46. Forest plot of frequent insomnia symptoms [Lane *et al.* (2019)] and colorectal cancer association in males

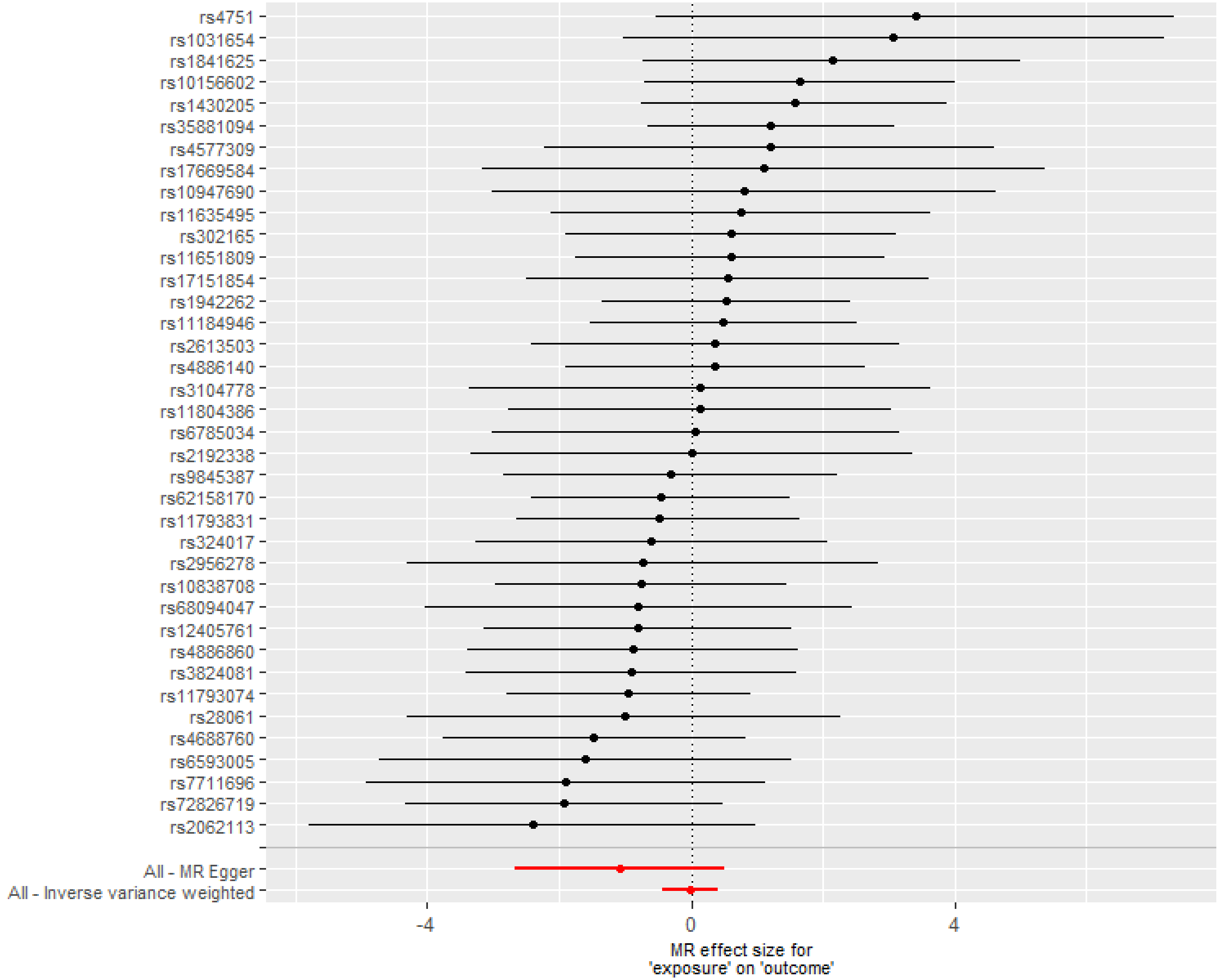

Abbreviations: MR: Mendelian randomization

Supplementary Figure S47. Forest plot of frequent insomnia symptoms [Lane *et al.* (2019)] and colorectal cancer association in females

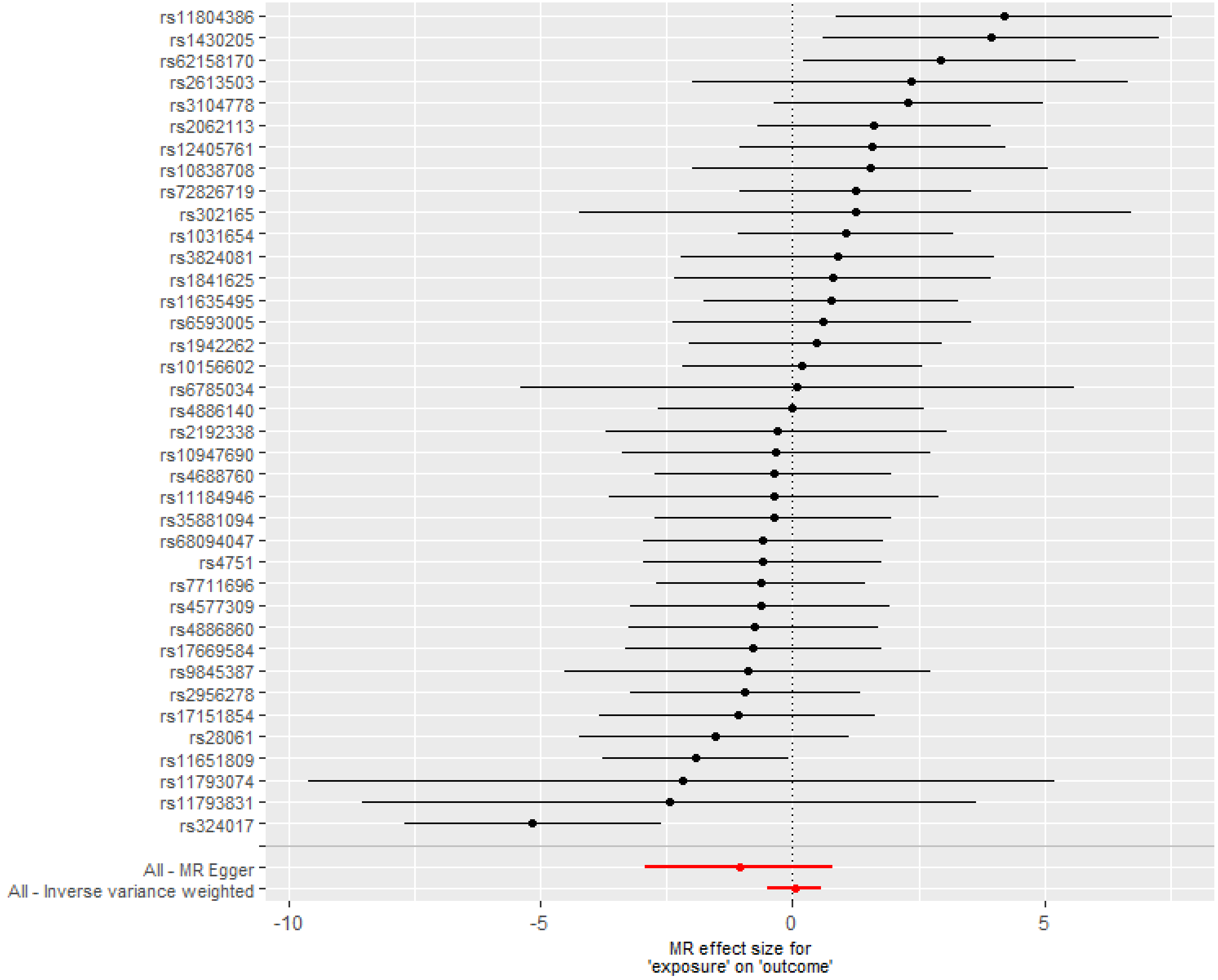

Abbreviations: MR: Mendelian randomization

Supplementary Figure S48. Forest plot of frequent insomnia symptoms [Lane *et al.* (2019)] and colorectal cancer association

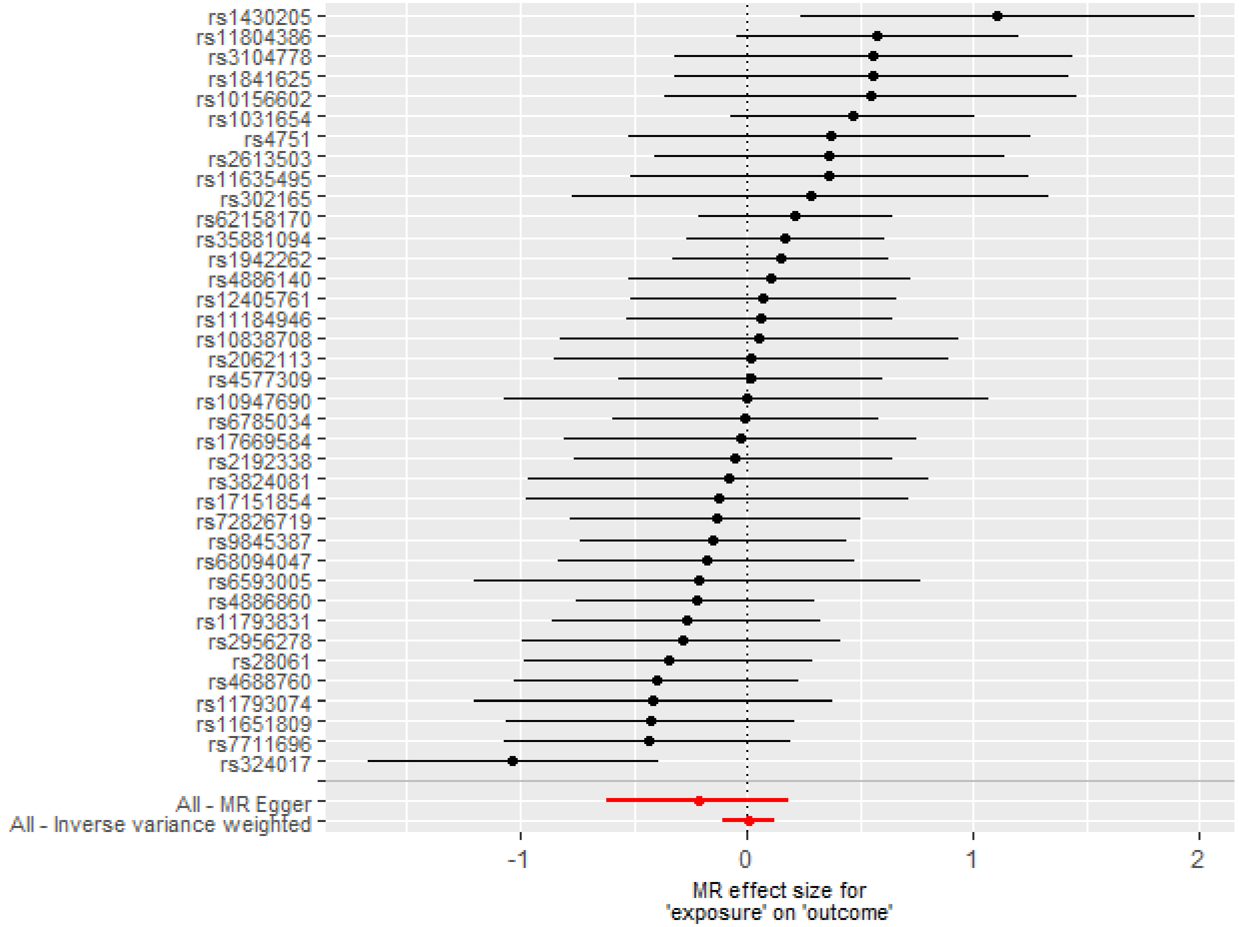

Abbreviations: MR: Mendelian randomization

Supplementary Figure S49. Forest plot of frequent insomnia symptoms [Lane *et al.* (2019)] and colon cancer association in males

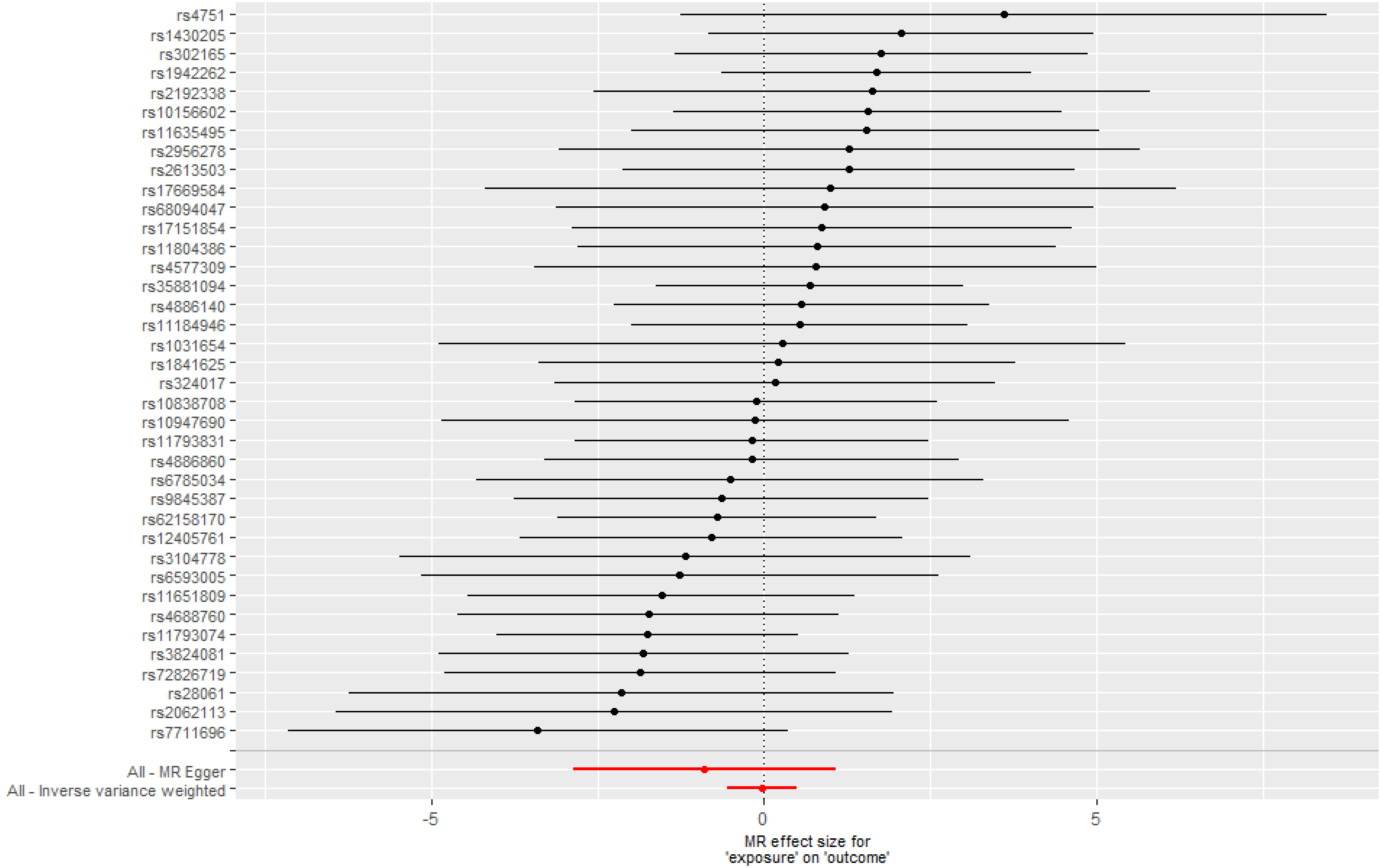

Abbreviations: MR: Mendelian randomization

Supplementary Figure S50. Forest plot of frequent insomnia symptoms [Lane *et al.* (2019)] and colon cancer association in females

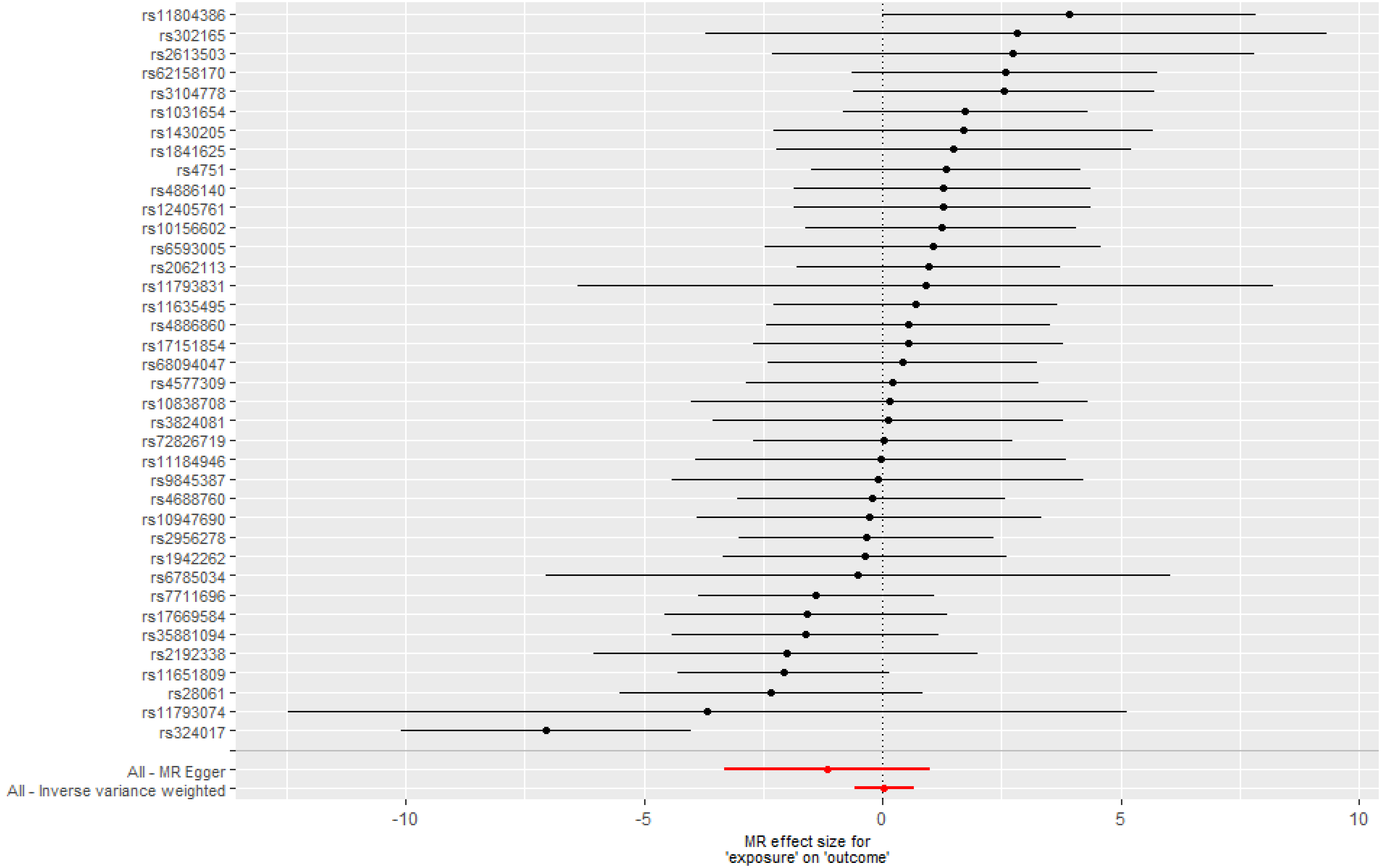

Abbreviations: MR: Mendelian randomization

Supplementary Figure S51. Forest plot of frequent insomnia symptoms [Lane *et al.* (2019)] and colon cancer association

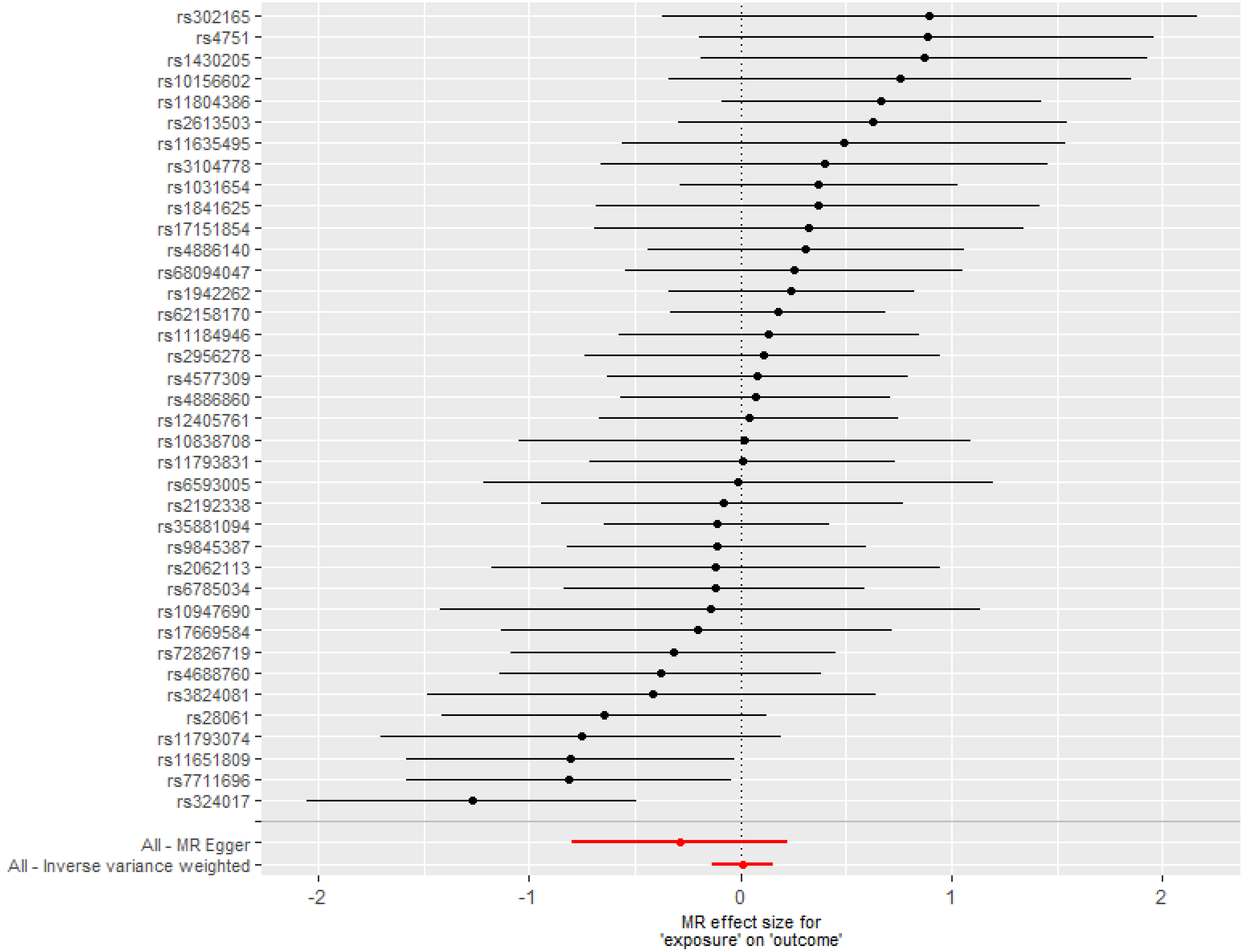

Abbreviations: MR: Mendelian randomization

Supplementary Figure S52. Forest plot of frequent insomnia symptoms [Lane *et al.* (2019)] and proximal colon cancer association

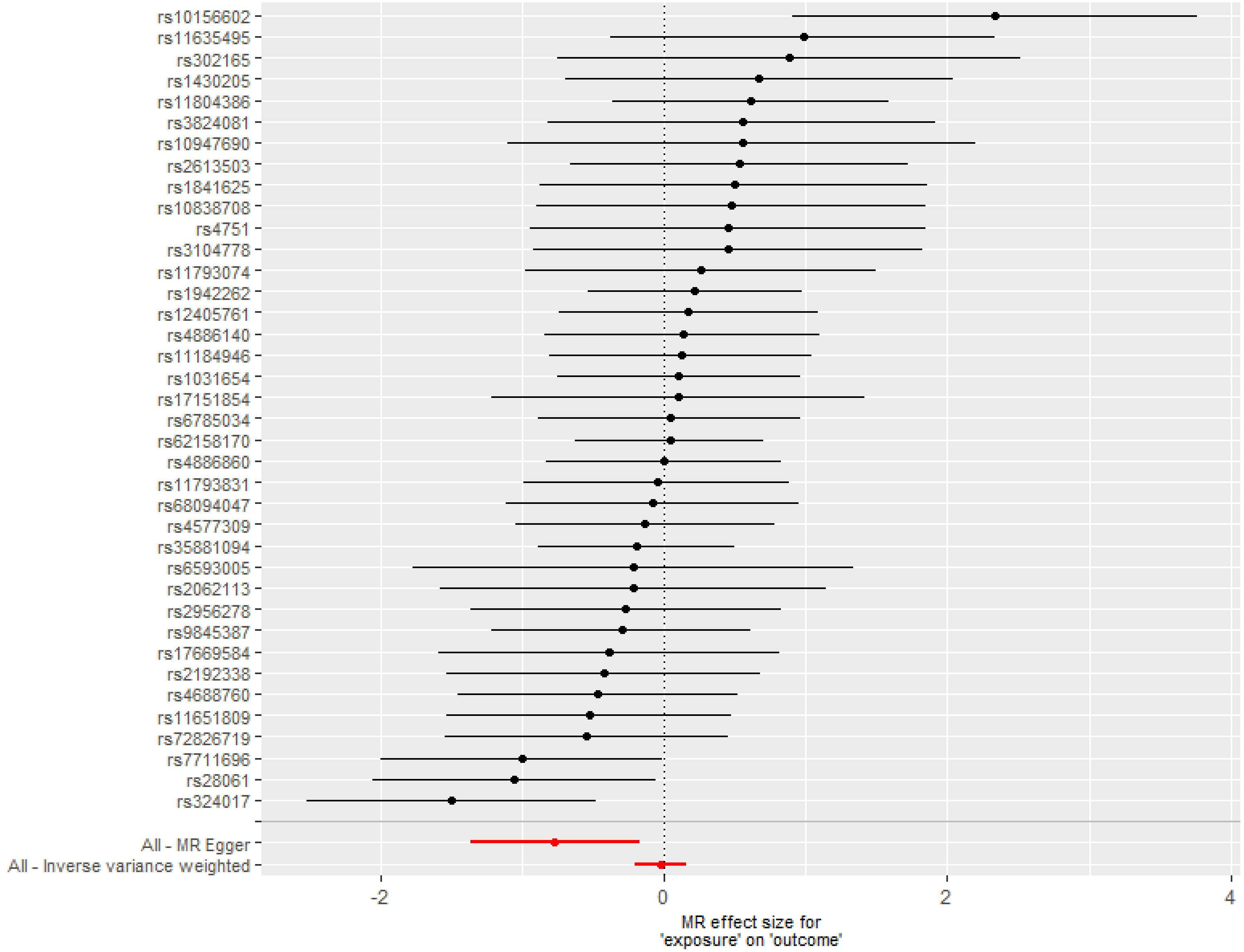

Abbreviations: MR: Mendelian randomization

Supplementary Figure S53. Forest plot of frequent insomnia symptoms [Lane *et al.* (2019)] and distal colon cancer association

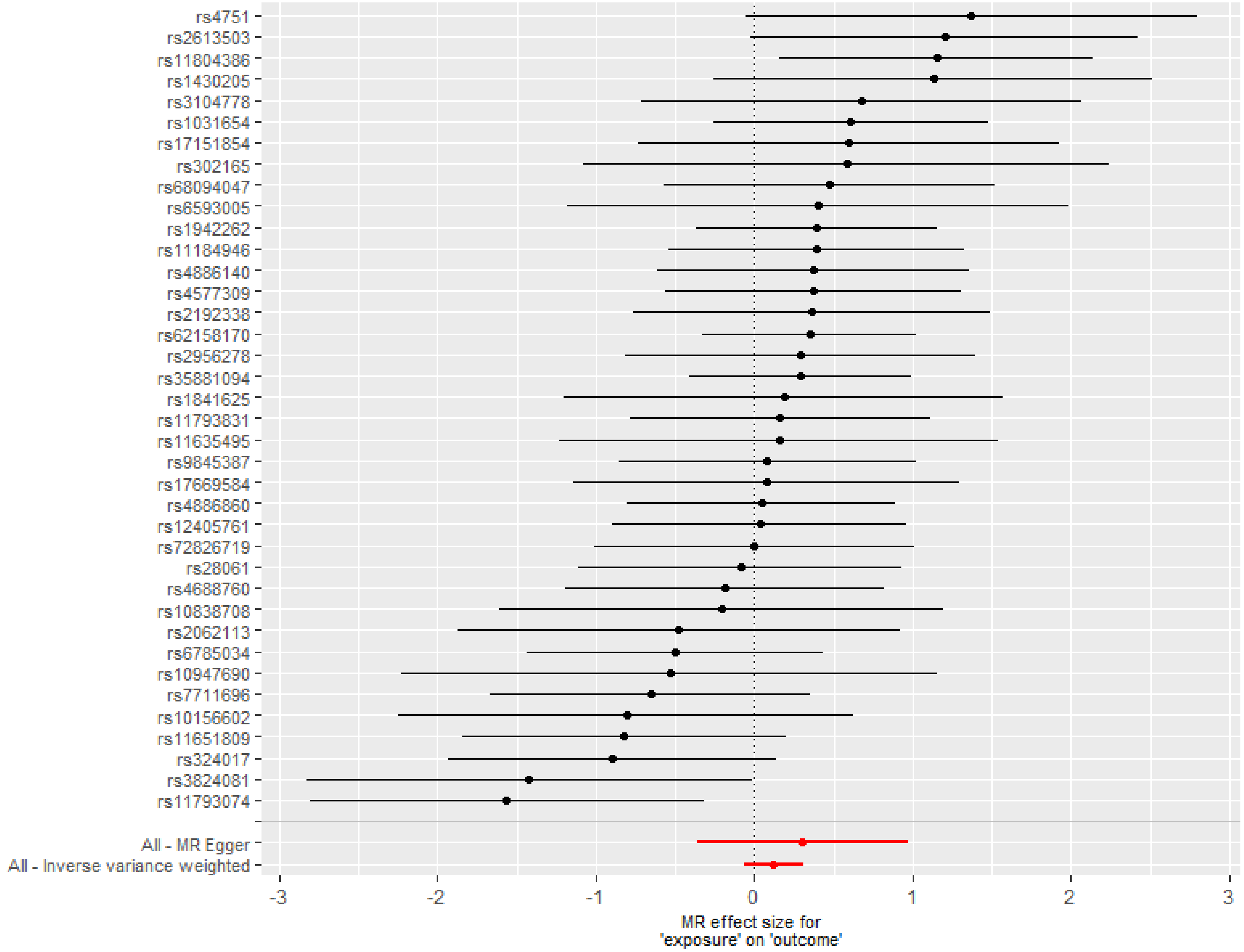

Abbreviations: MR: Mendelian randomization

Supplementary Figure S54. Forest plot of frequent insomnia symptoms [Lane *et al.* (2019)] and rectal cancer association in males

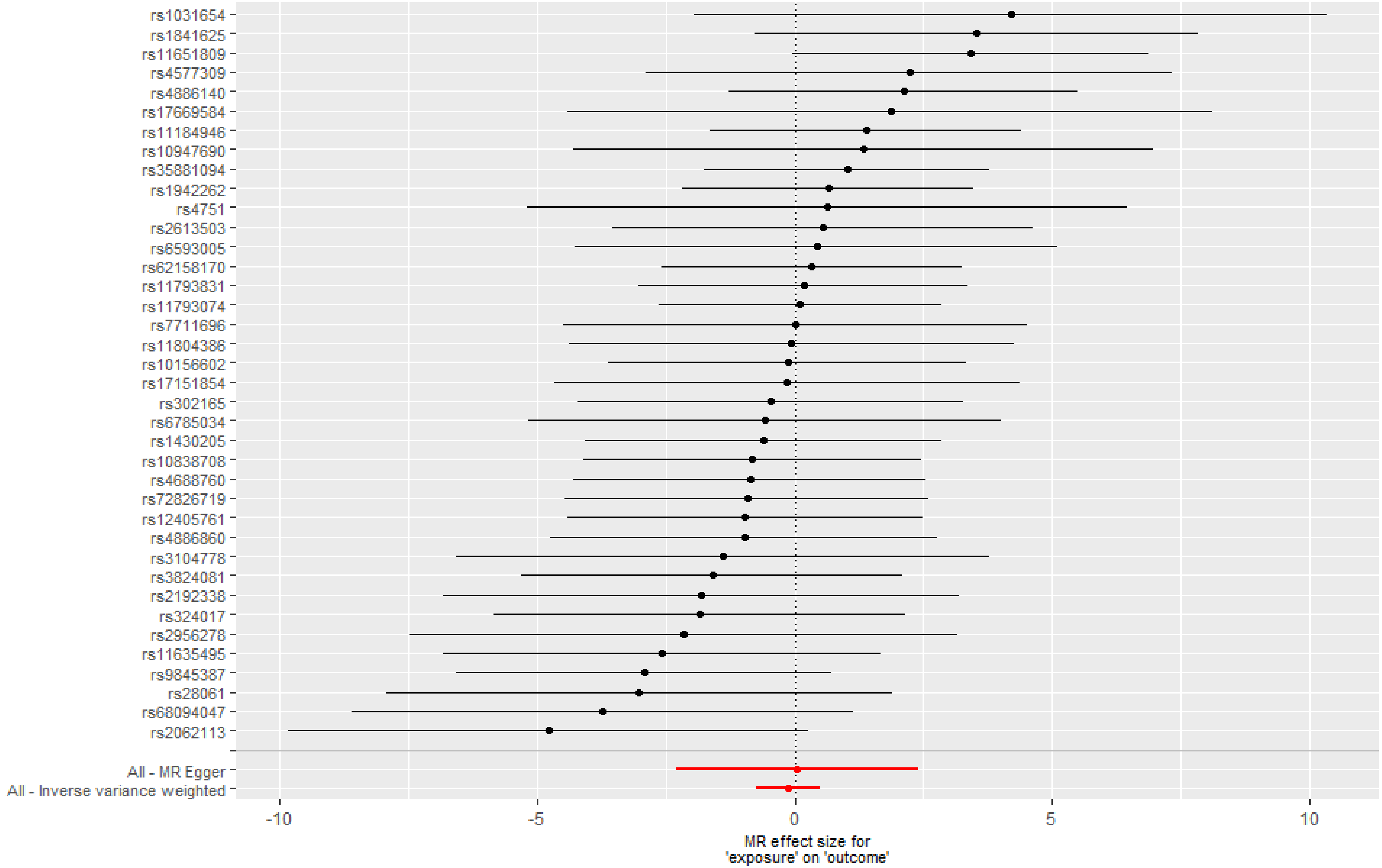

Abbreviations: MR: Mendelian randomization

Supplementary Figure S55. Forest plot of frequent insomnia symptoms [Lane *et al.* (2019)] and rectal cancer association in females

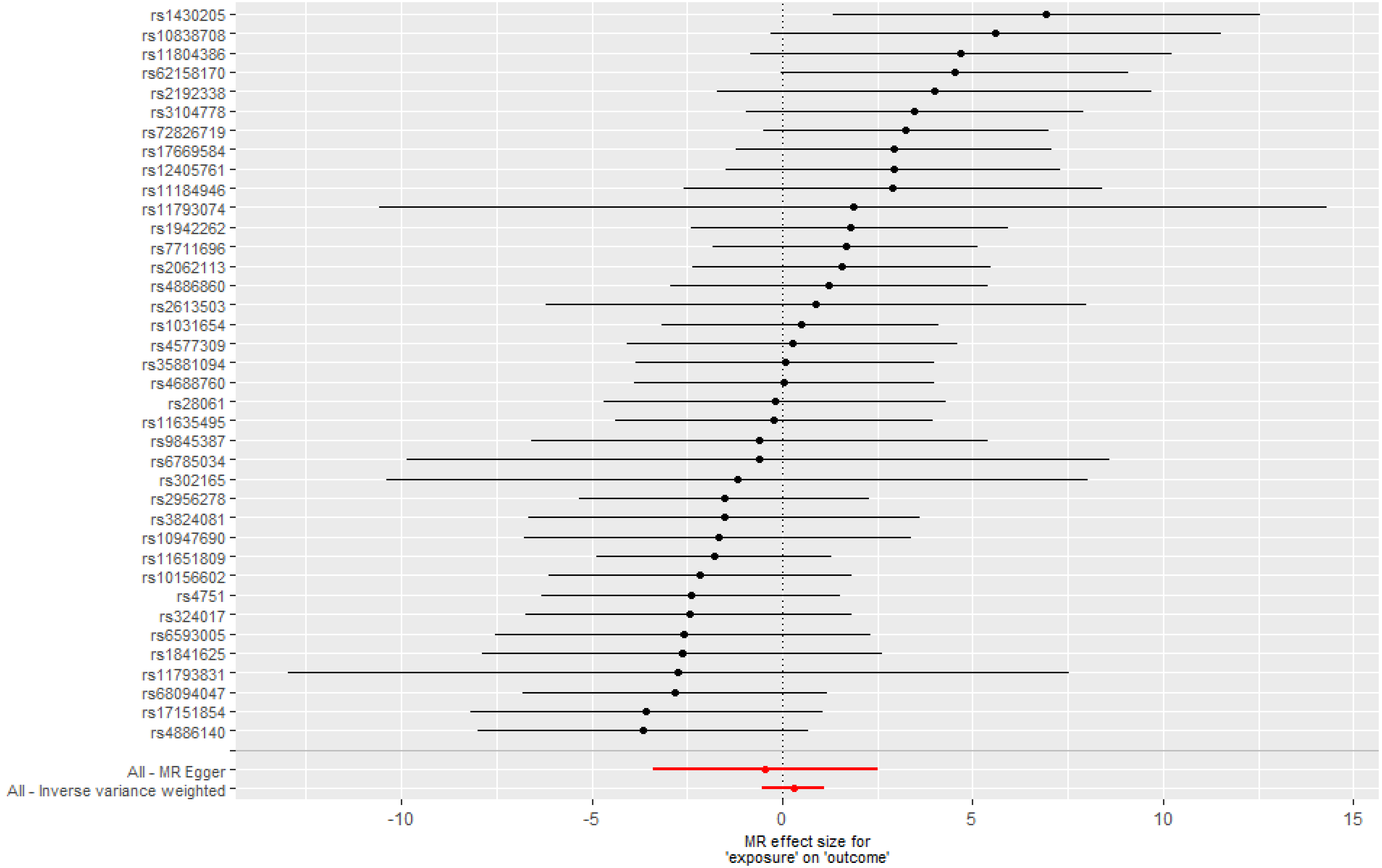

Abbreviations: MR: Mendelian randomization

Supplementary Figure S56. Forest plot of frequent insomnia symptoms [Lane *et al.* (2019)] and rectal cancer association

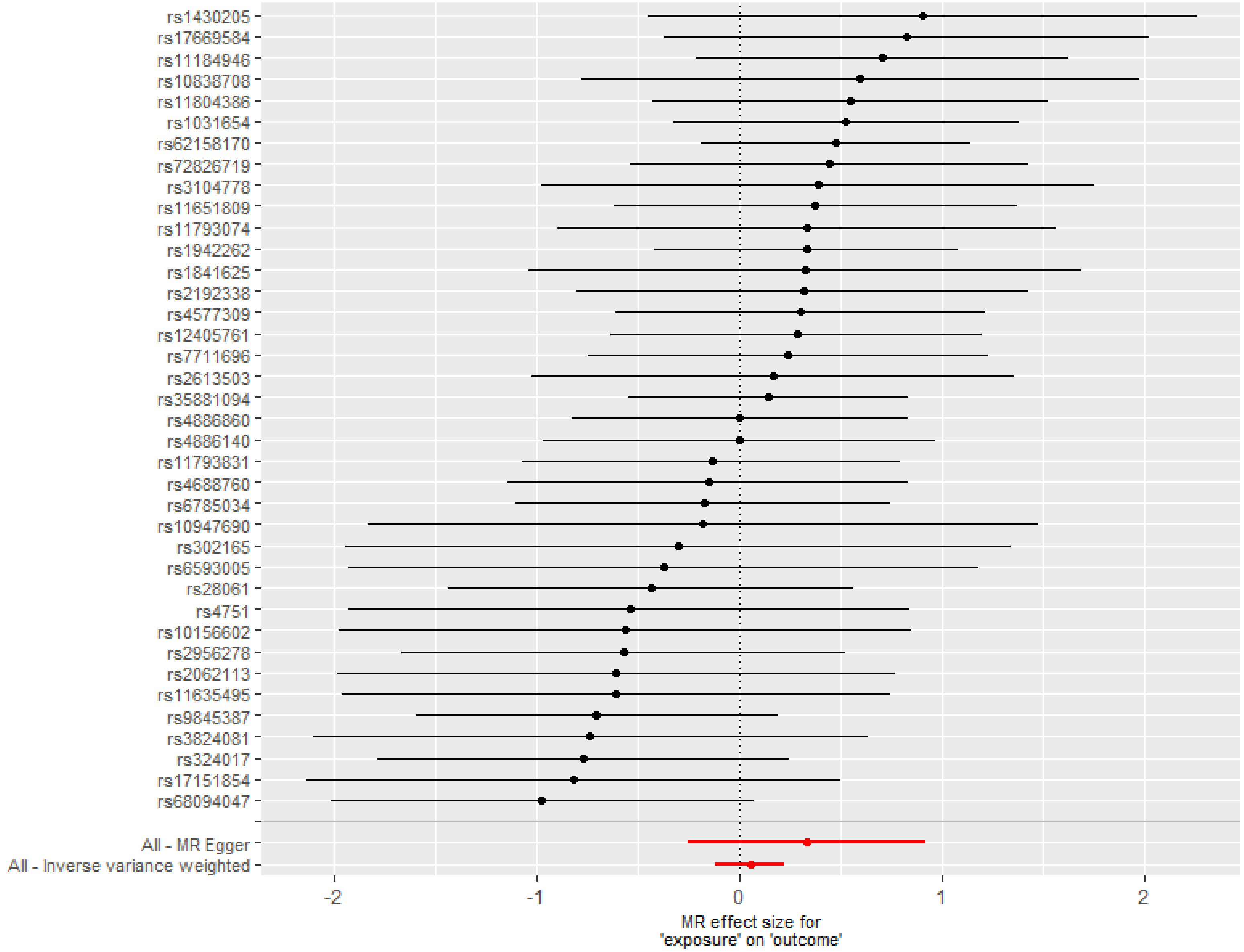

Abbreviations: MR: Mendelian randomization

Supplementary Figure S57. Funnel plot of frequent insomnia symptoms [Lane *et al.* (2019)] and colorectal cancer association in males

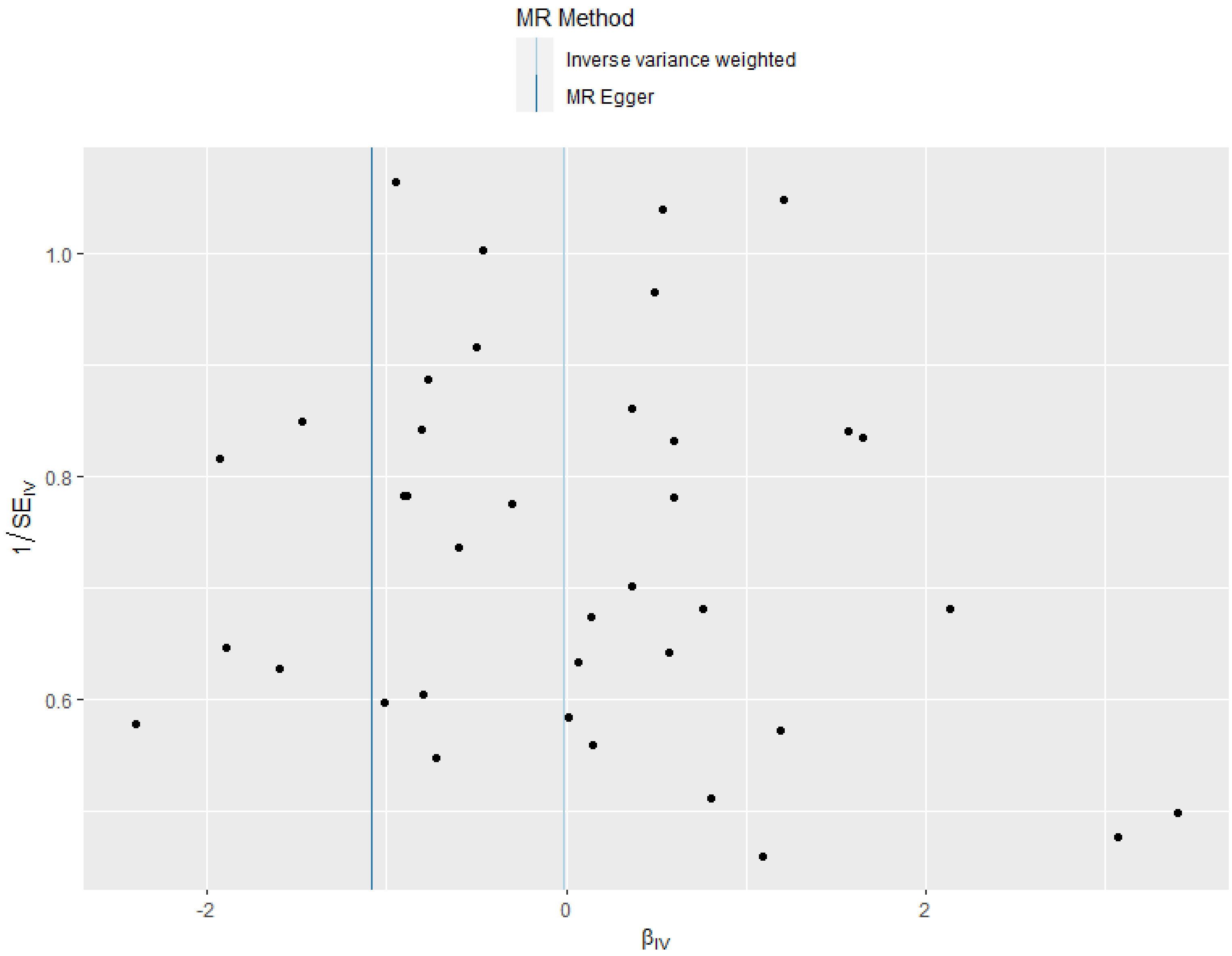

Abbreviations: MR: Mendelian randomization; SE_{IV}: Inverse-variance Standard Error; β_{IV}: Inverse-variance beta coefficient

Supplementary Figure S58. Funnel plot of frequent insomnia symptoms [Lane *et al.* (2019)] and colorectal cancer association in females

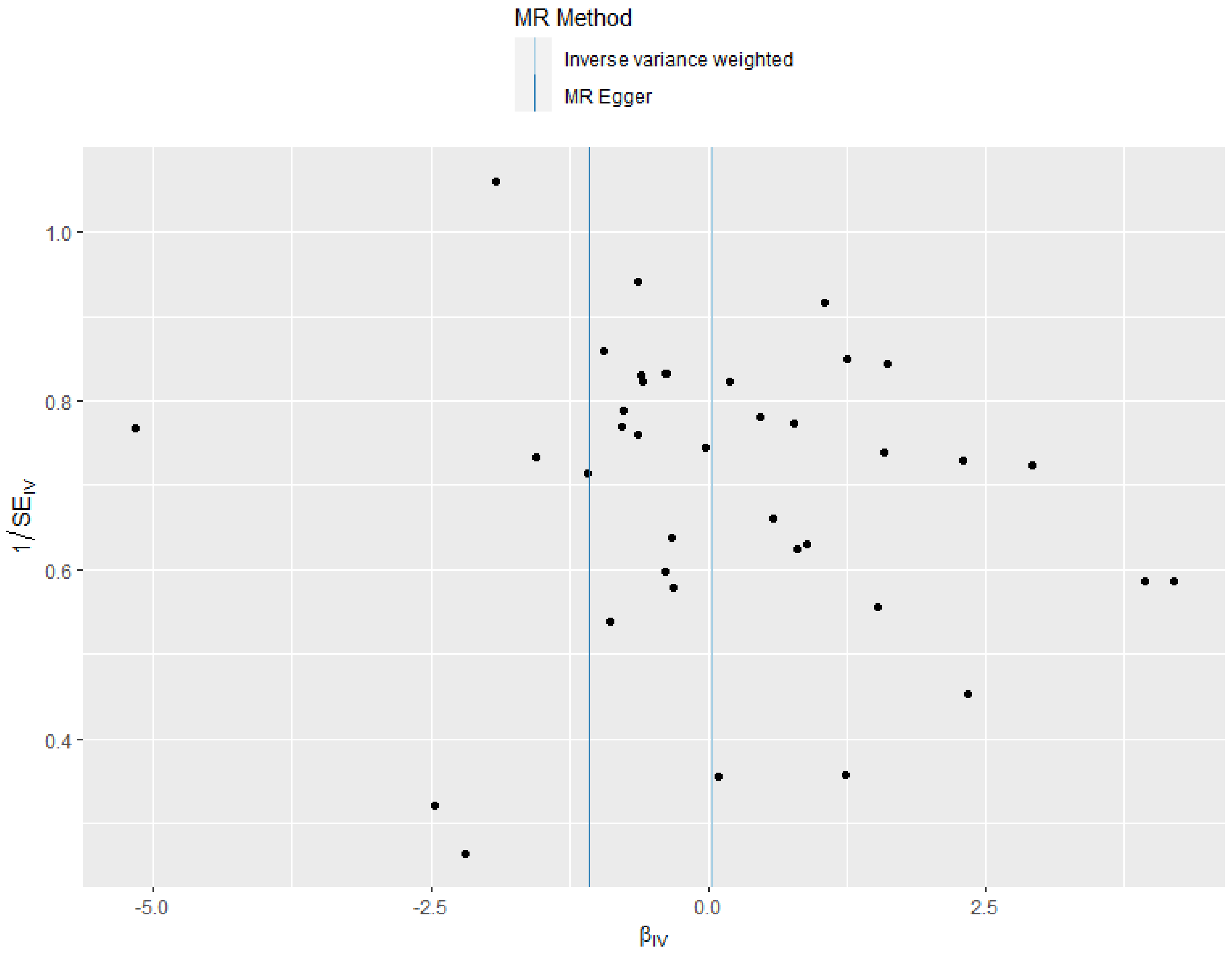

Abbreviations: MR: Mendelian randomization; SE_{IV}: Inverse-variance Standard Error; β_{IV}: Inverse-variance beta coefficient

Supplementary Figure S59. Funnel plot of frequent insomnia symptoms [Lane *et al.* (2019)] and colorectal cancer association

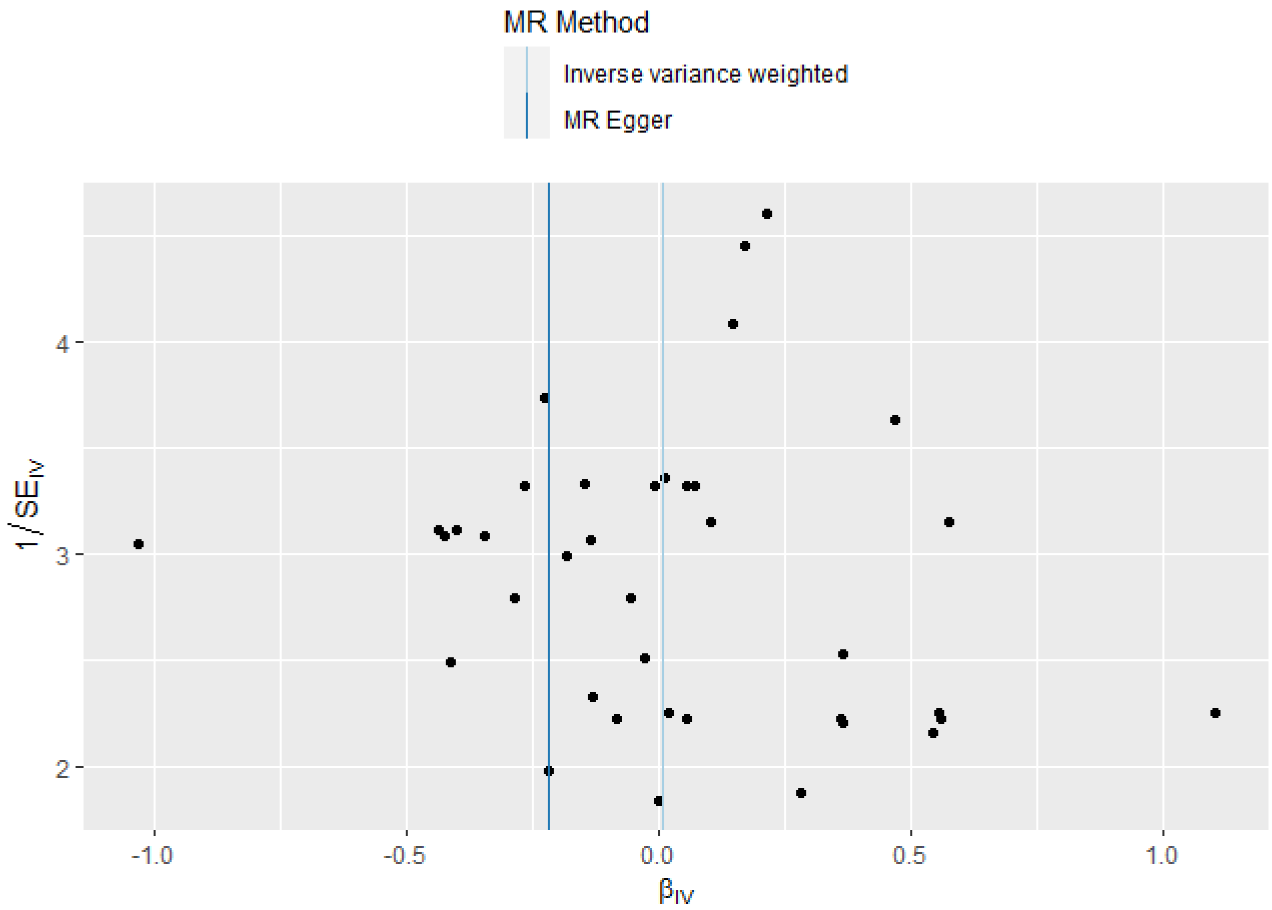

Abbreviations: MR: Mendelian randomization; SE_{IV}: Inverse-variance Standard Error; β_{IV}: Inverse-variance beta coefficient

Supplementary Figure S60. Funnel plot of frequent insomnia symptoms [Lane *et al.* (2019)] and colon cancer association in males

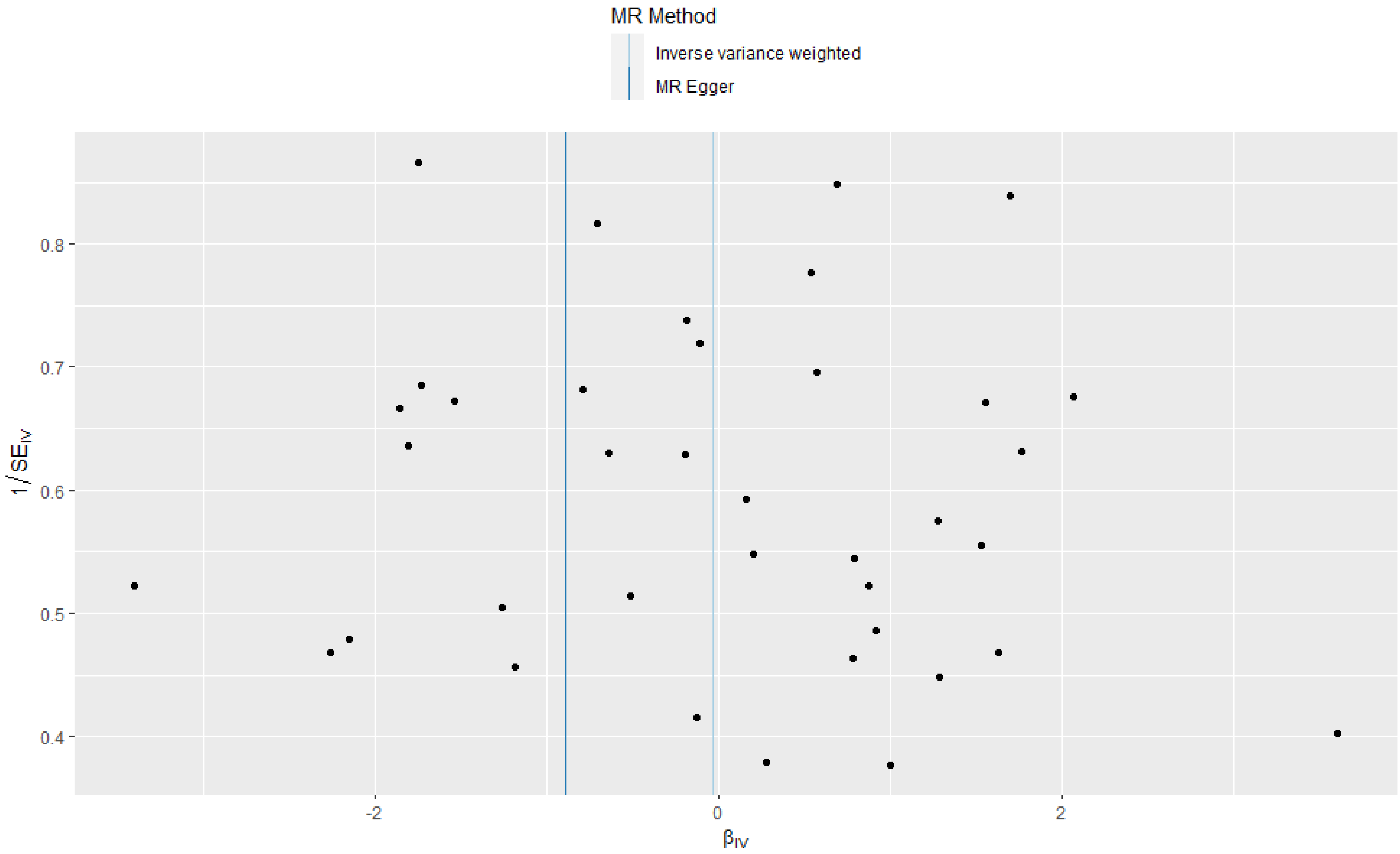

Abbreviations: MR: Mendelian randomization; SE_{IV}: Inverse-variance Standard Error; β_{IV}: Inverse-variance beta coefficient

Supplementary Figure S61. Funnel plot of frequent insomnia symptoms [Lane *et al.* (2019)] and colon cancer association in females

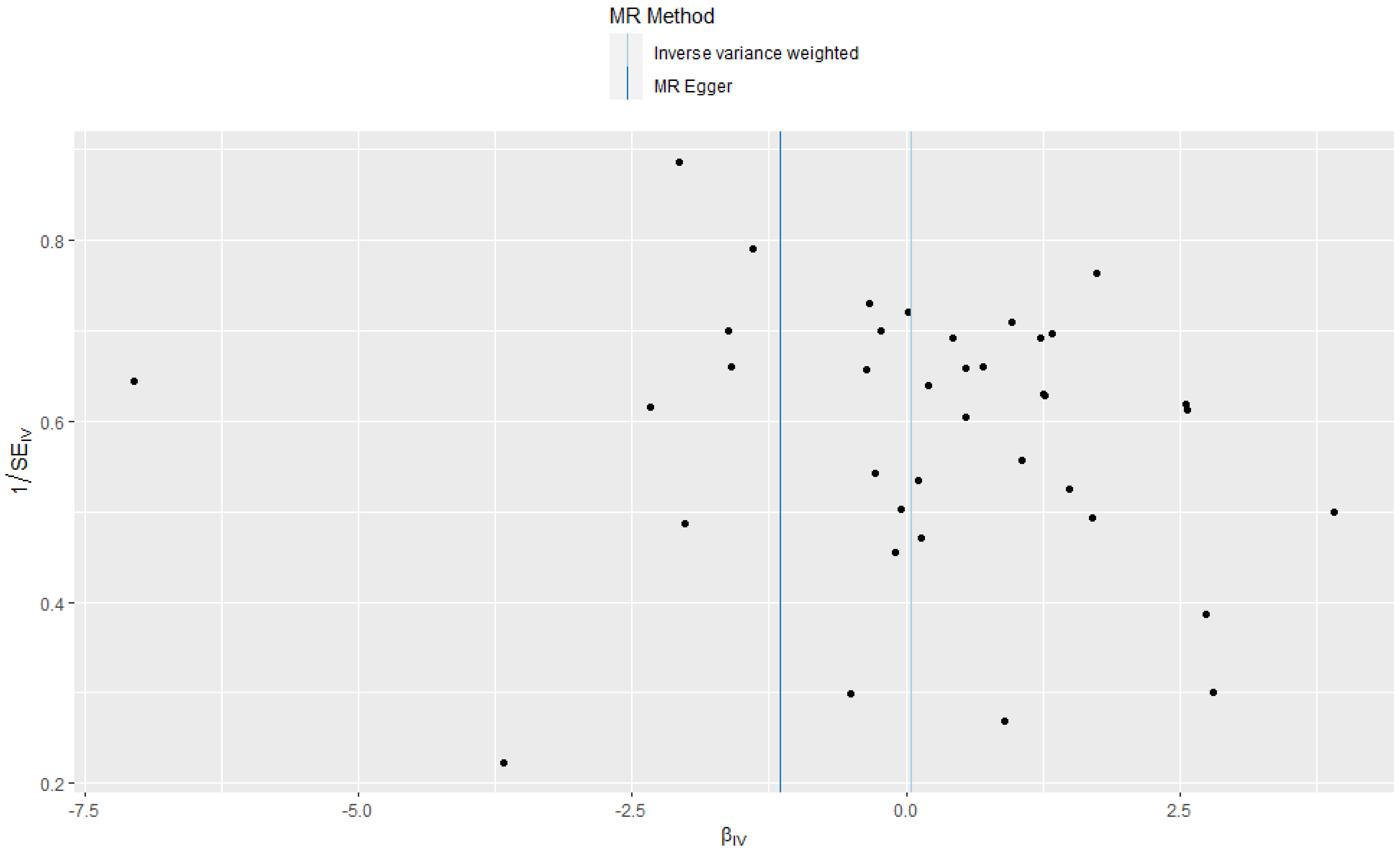

Abbreviations: MR: Mendelian randomization; SE_{IV}: Inverse-variance Standard Error; β_{IV}: Inverse-variance beta coefficient

Supplementary Figure S62. Funnel plot of frequent insomnia symptoms [Lane *et al.* (2019)] and colon cancer association

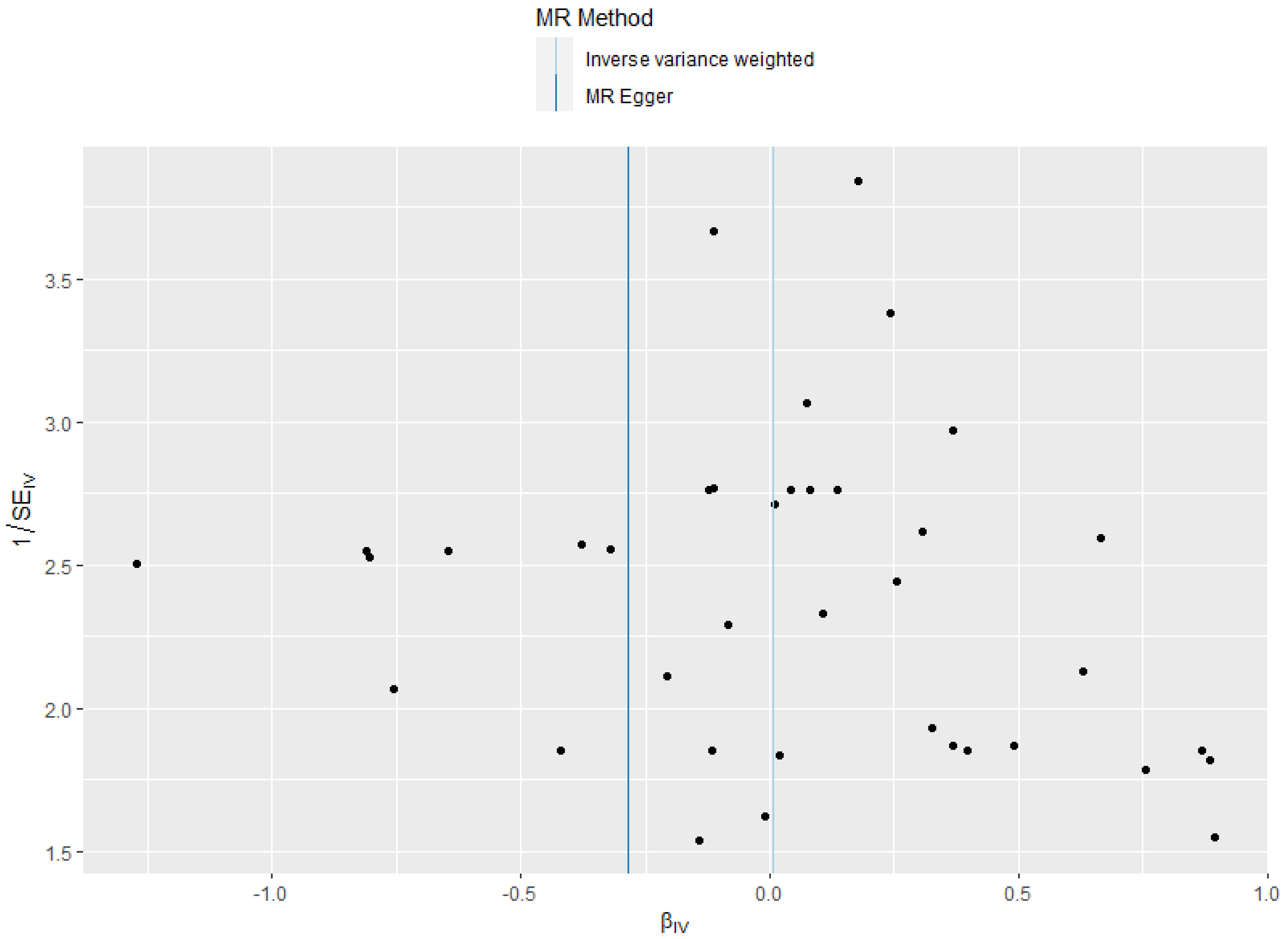

Abbreviations: MR: Mendelian randomization; SE_{IV}: Inverse-variance Standard Error; β_{IV}: Inverse-variance beta coefficient

Supplementary Figure S63. Funnel plot of frequent insomnia symptoms [Lane *et al.* (2019)] and proximal colon cancer association

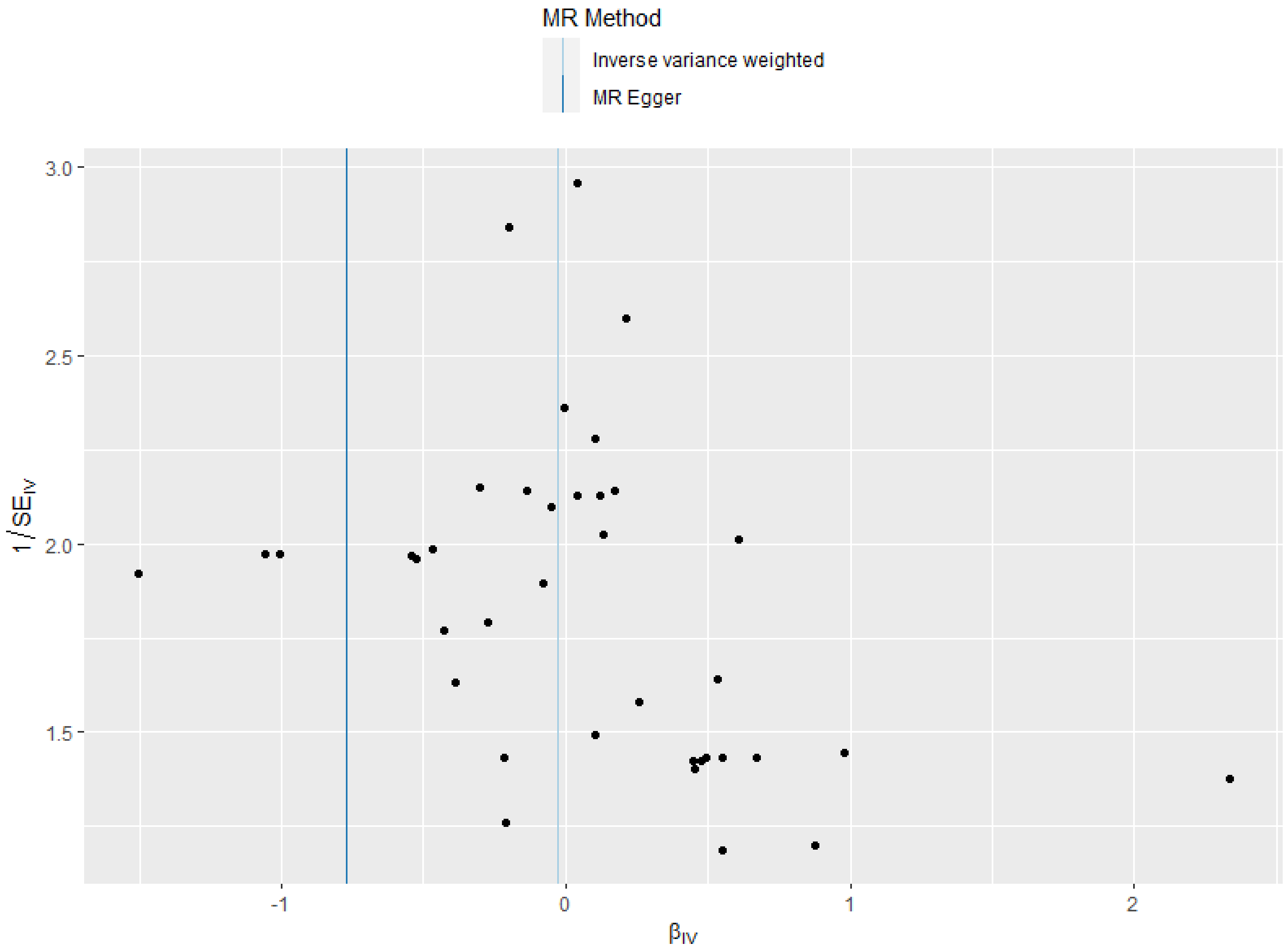

Abbreviations: MR: Mendelian randomization; SE_{IV}: Inverse-variance Standard Error; β_{IV}: Inverse-variance beta coefficient

Supplementary Figure S64. Funnel plot of frequent insomnia symptoms [Lane *et al.* (2019)] and distal colon cancer association

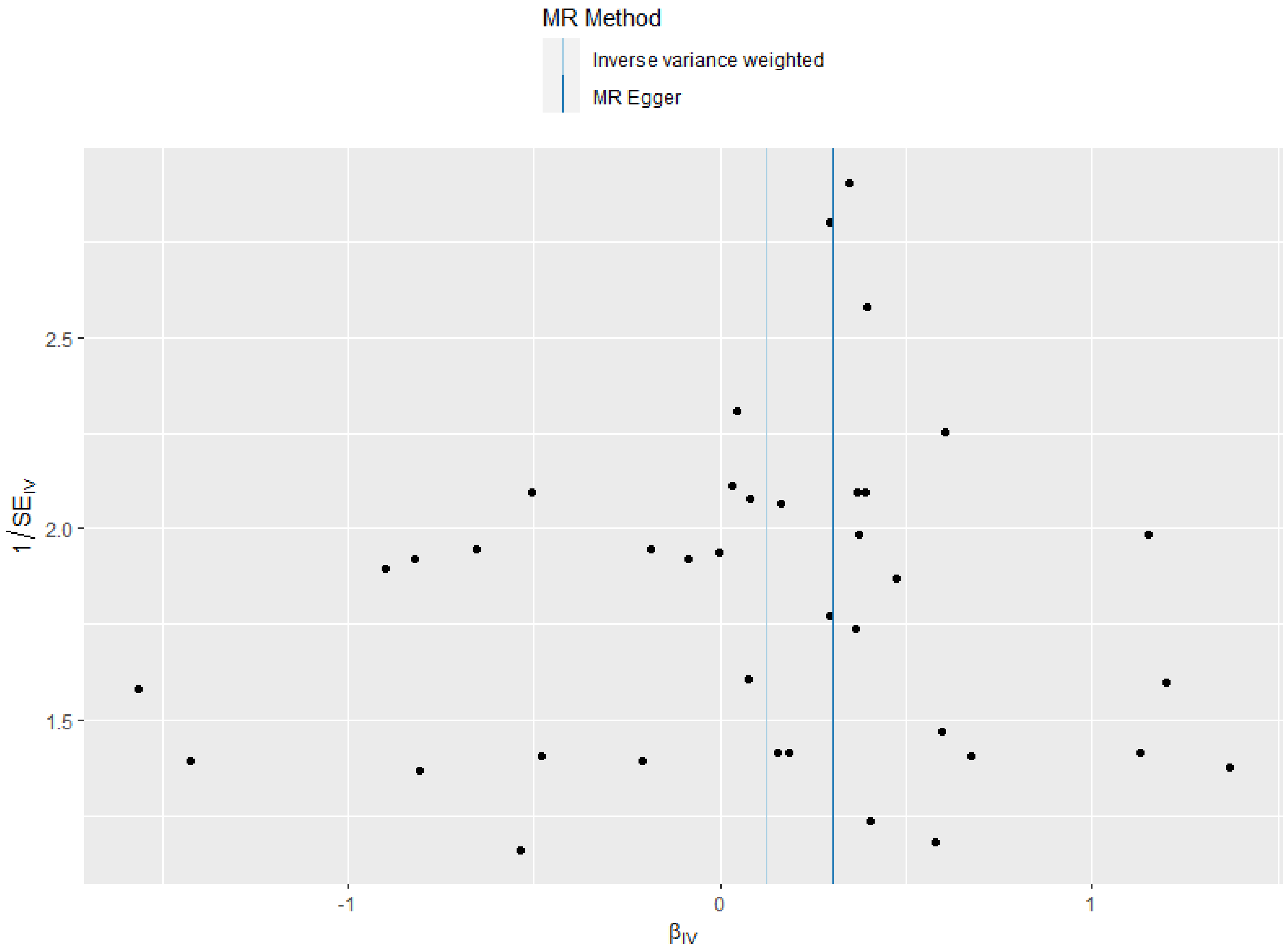

Abbreviations: MR: Mendelian randomization; SE_{IV}: Inverse-variance Standard Error; β_{IV}: Inverse-variance beta coefficient

Supplementary Figure S65. Funnel plot of frequent insomnia symptoms [Lane *et al.* (2019)] and rectal cancer association in males

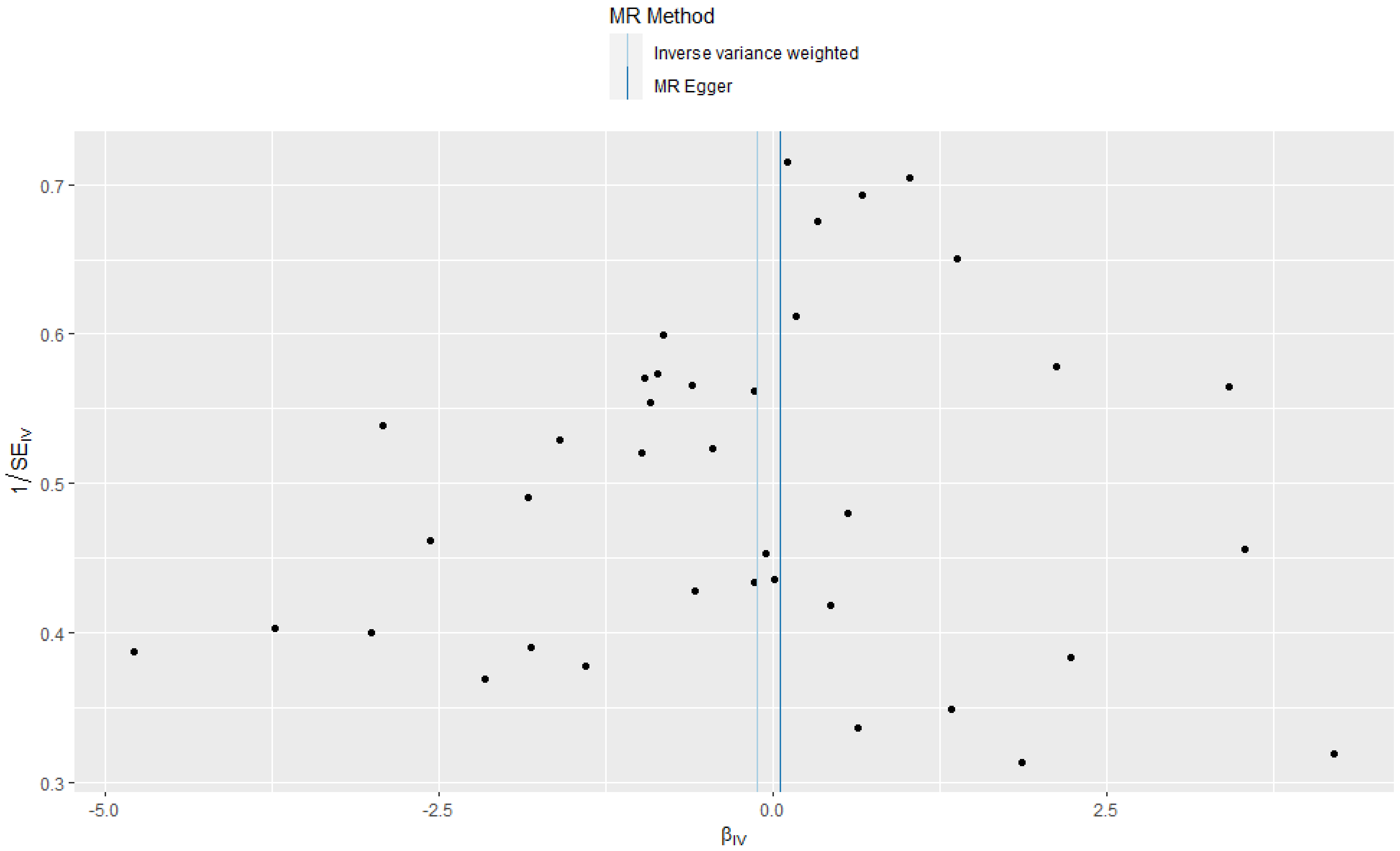

Abbreviations: MR: Mendelian randomization; SE_{IV}: Inverse-variance Standard Error; β_{IV}: Inverse-variance beta coefficient

Supplementary Figure S66. Funnel plot of frequent insomnia symptoms [Lane *et al.* (2019)] and rectal cancer association in females

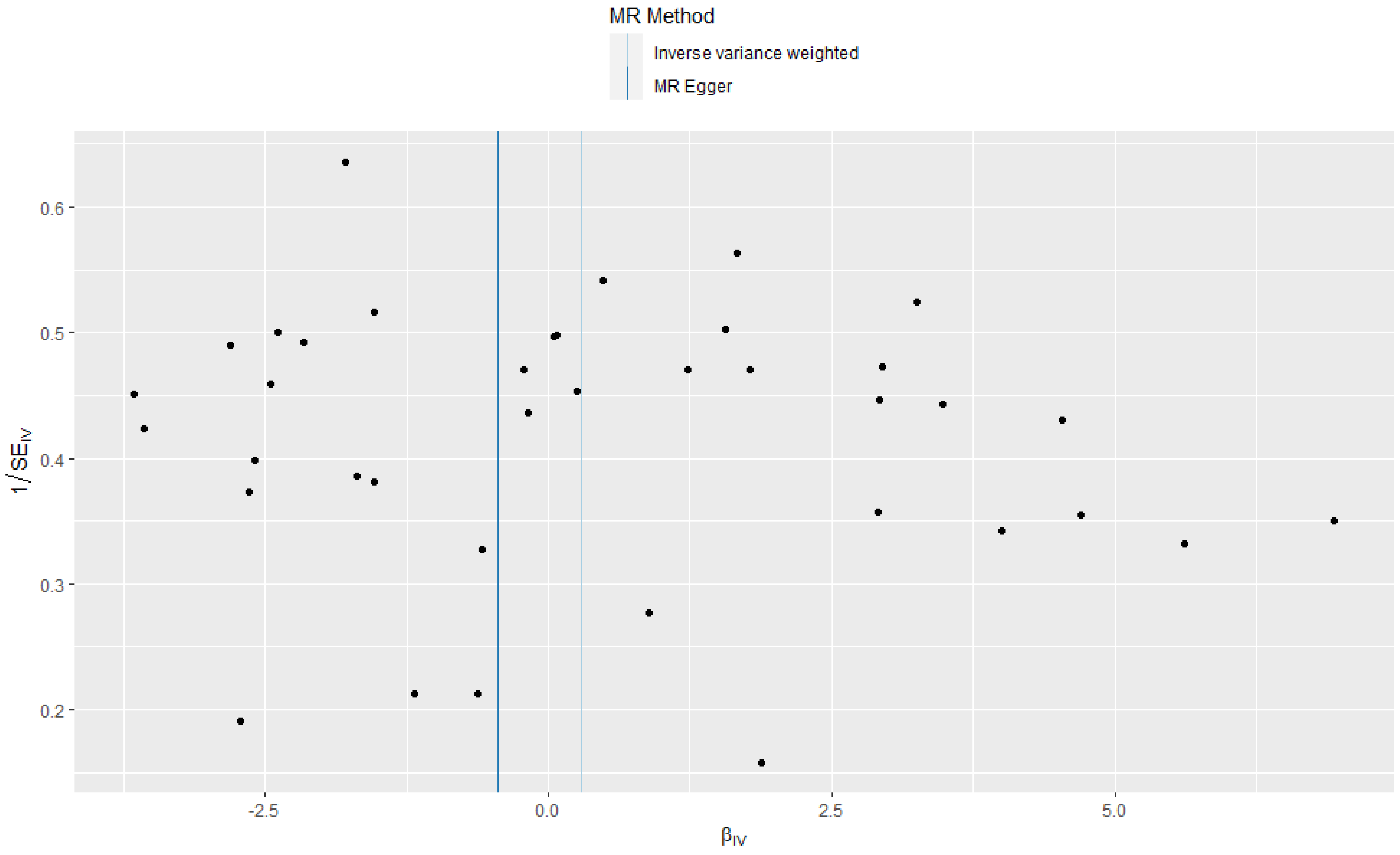

Abbreviations: MR: Mendelian randomization; SE_{IV}: Inverse-variance Standard Error; β_{IV}: Inverse-variance beta coefficient

Supplementary Figure S67. Funnel plot of frequent insomnia symptoms [Lane *et al.* (2019)] and rectal cancer association

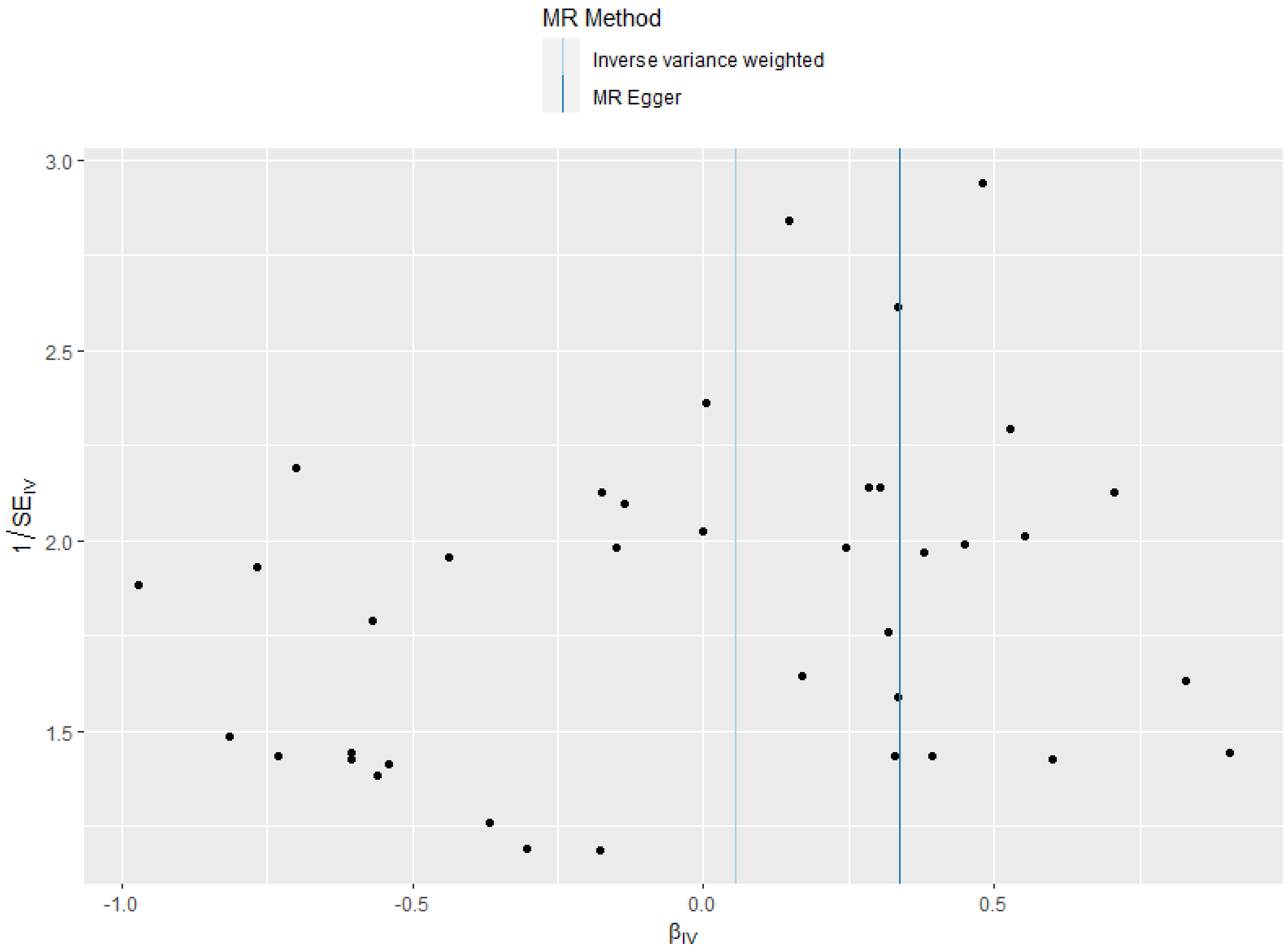

Abbreviations: MR: Mendelian randomization; SE_{IV}: Inverse-variance Standard Error; β_{IV}: Inverse-variance beta coefficient

Primary MR analyses: Any insomnia symptoms [Lane *et al.* (2019)]

Supplementary Figure S68. Scatter plot of any insomnia symptoms [Lane *et al.* (2019)] and colorectal cancer association

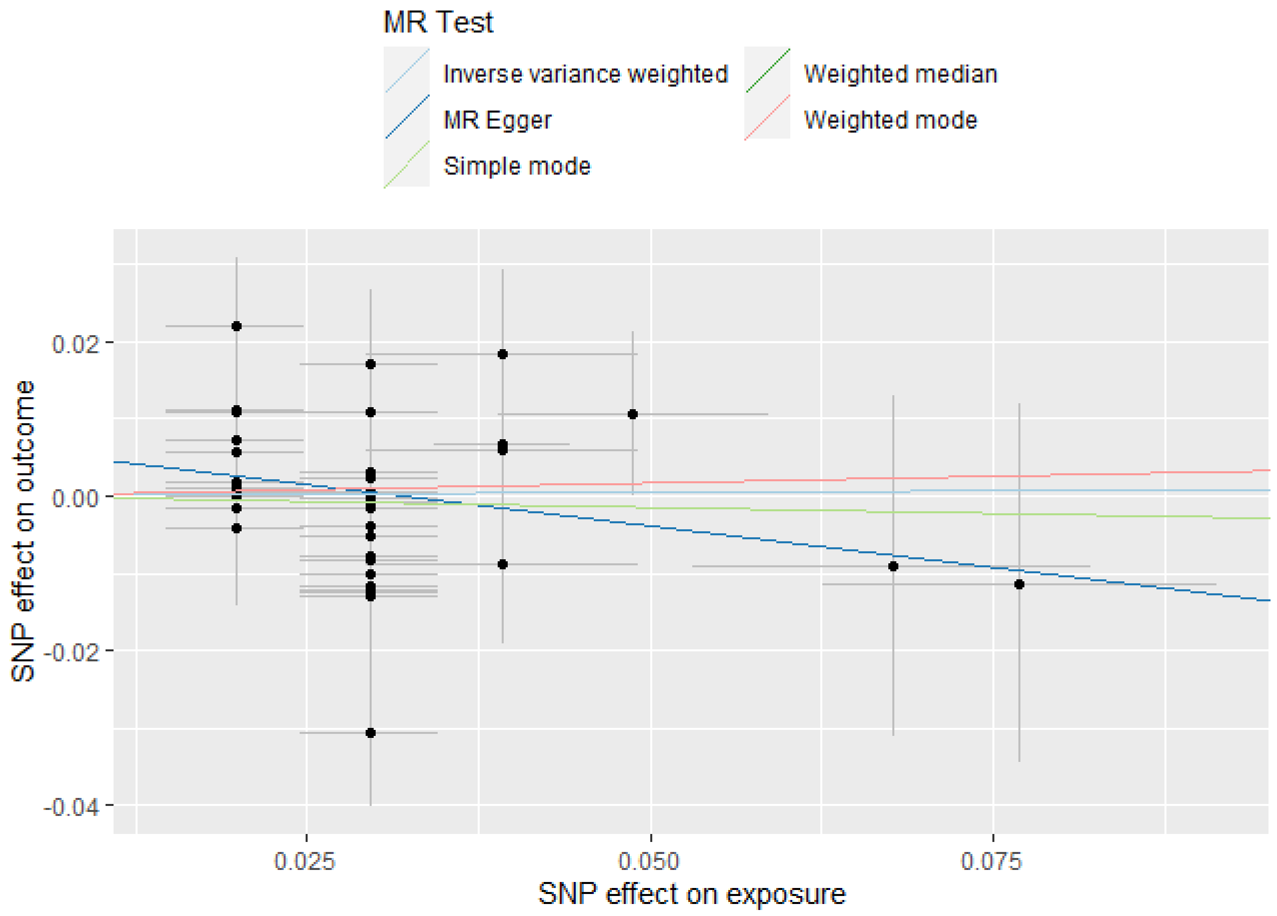

Abbreviations: MR: Mendelian randomization; SNP: Single Nucleotide Polymorphism

Supplementary Figure S69. Scatter plot of any insomnia symptoms [Lane *et al.* (2019)] and colon cancer association

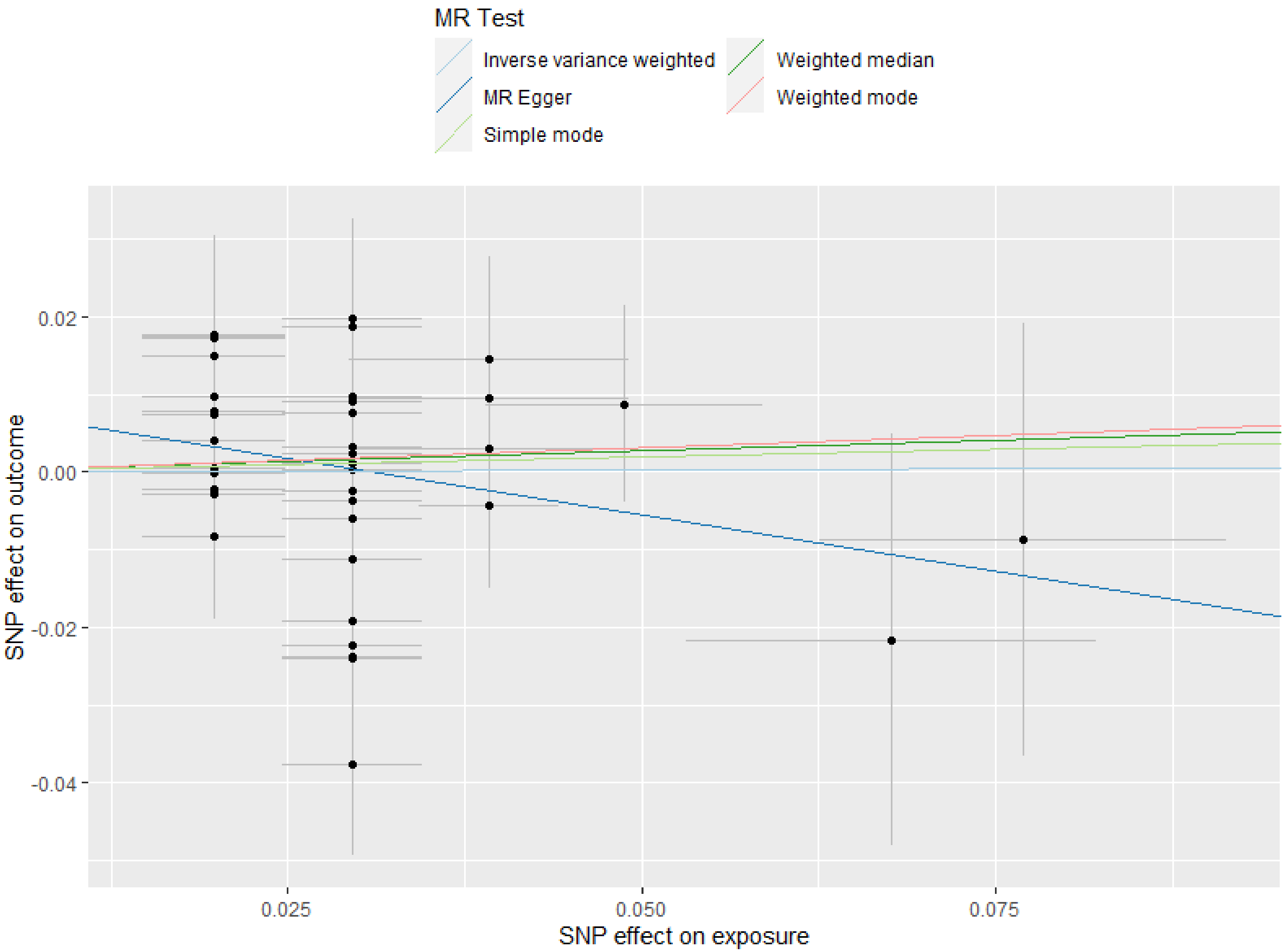

Abbreviations: MR: Mendelian randomization; SNP: Single Nucleotide Polymorphism

Supplementary Figure S70. Scatter plot of any insomnia symptoms [Lane *et al.* (2019)] and proximal colon cancer association

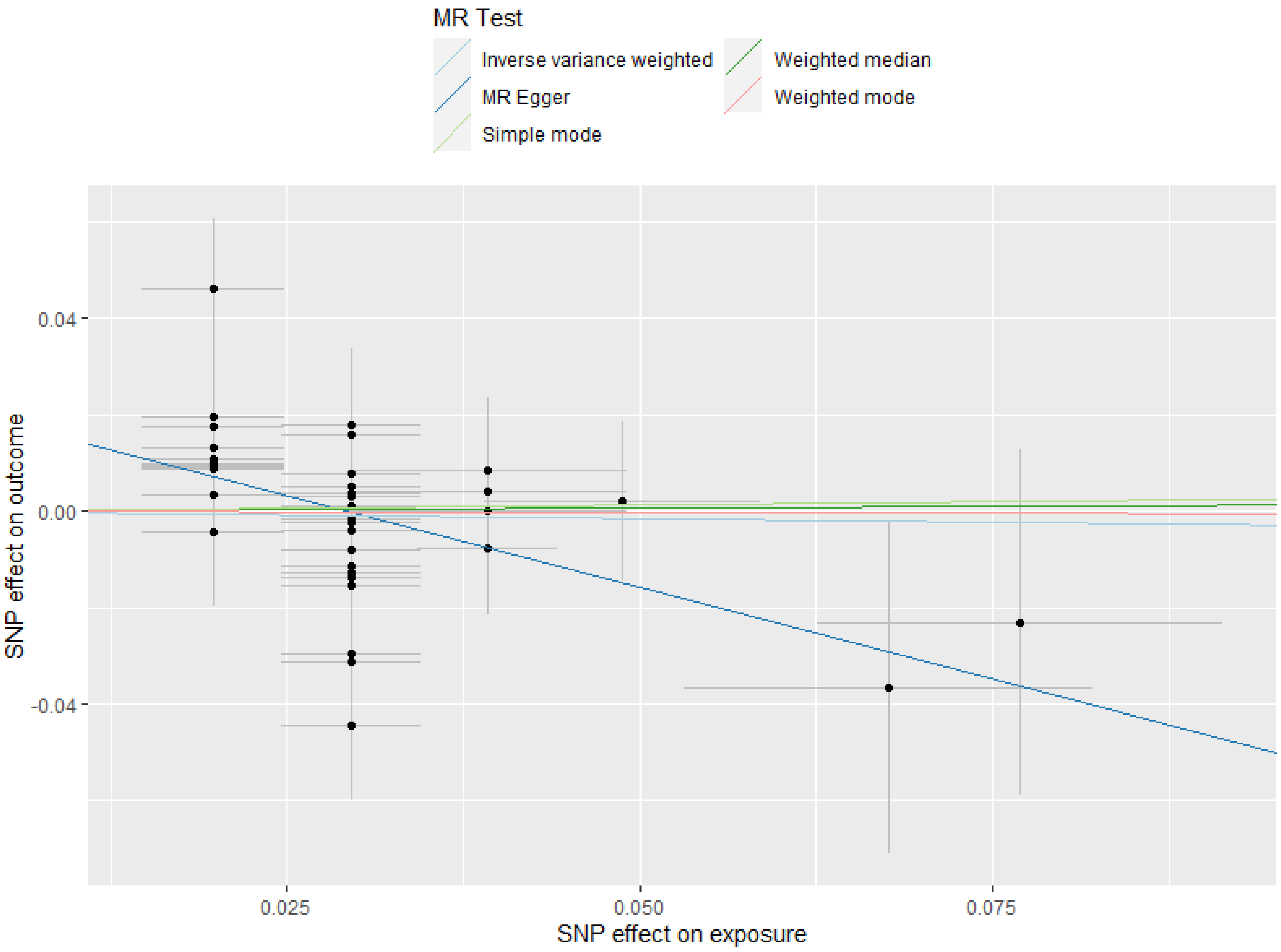

Abbreviations: MR: Mendelian randomization; SNP: Single Nucleotide Polymorphism

Supplementary Figure S71. Scatter plot of any insomnia symptoms [Lane *et al.* (2019)] and distal colon cancer association

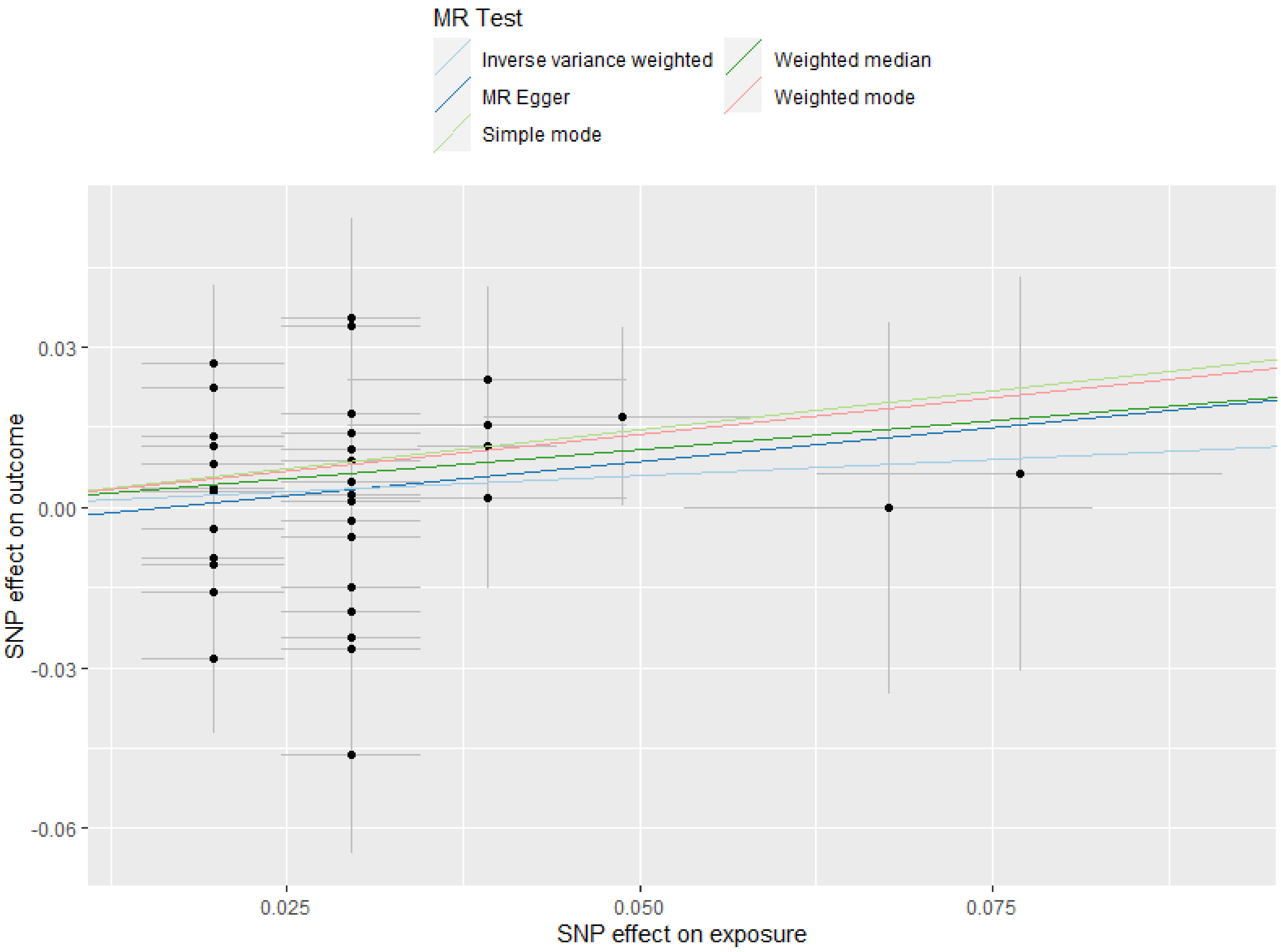

Abbreviations: MR: Mendelian randomization; SNP: Single Nucleotide Polymorphism

Supplementary Figure S72. Scatter plot of any insomnia symptoms [Lane *et al.* (2019)] and rectal cancer association

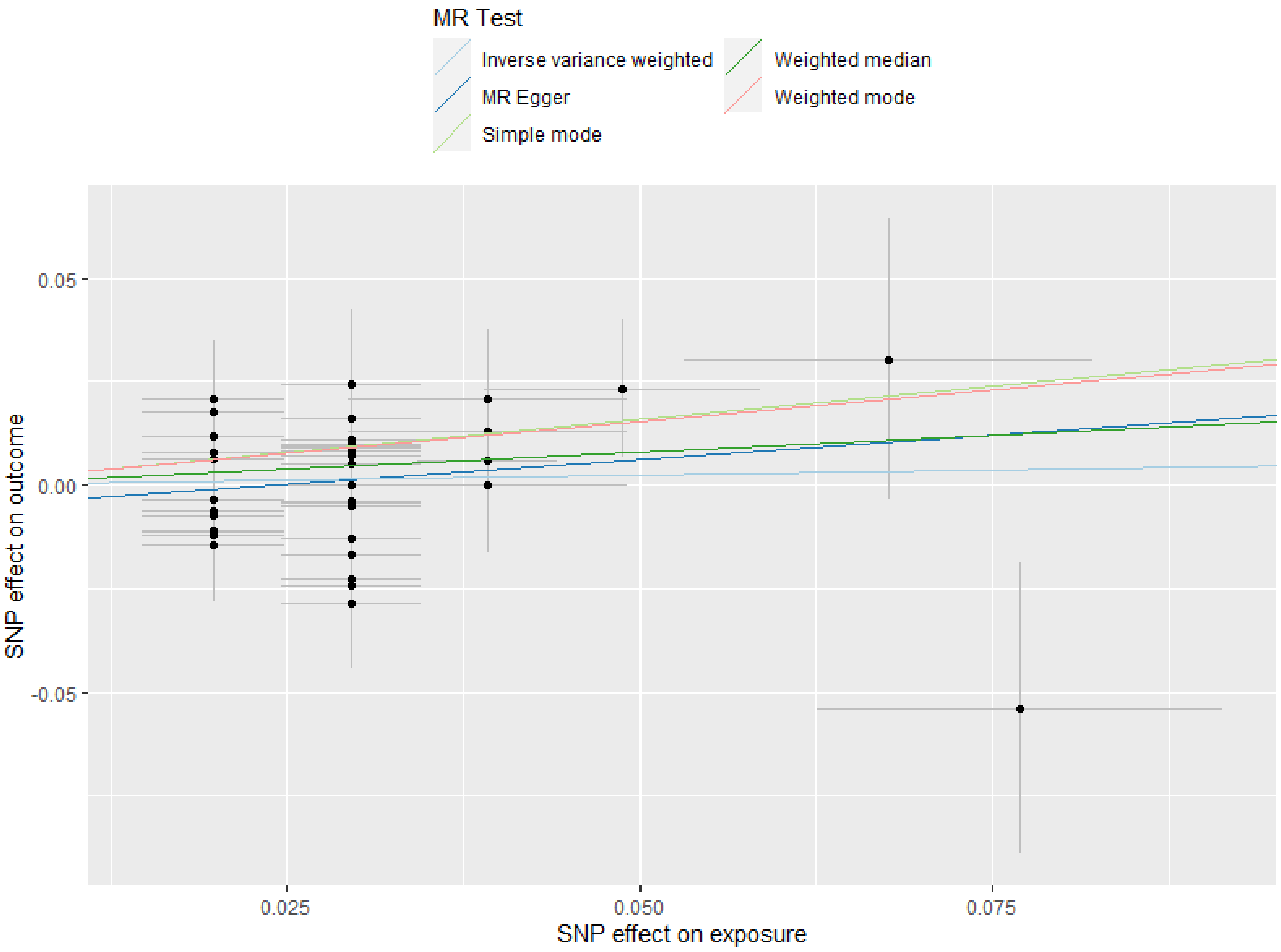

Abbreviations: MR: Mendelian randomization; SNP: Single Nucleotide Polymorphism

Supplementary Figure S73. Forest plot of any insomnia symptoms [Lane *et al.* (2019)] and colorectal cancer association

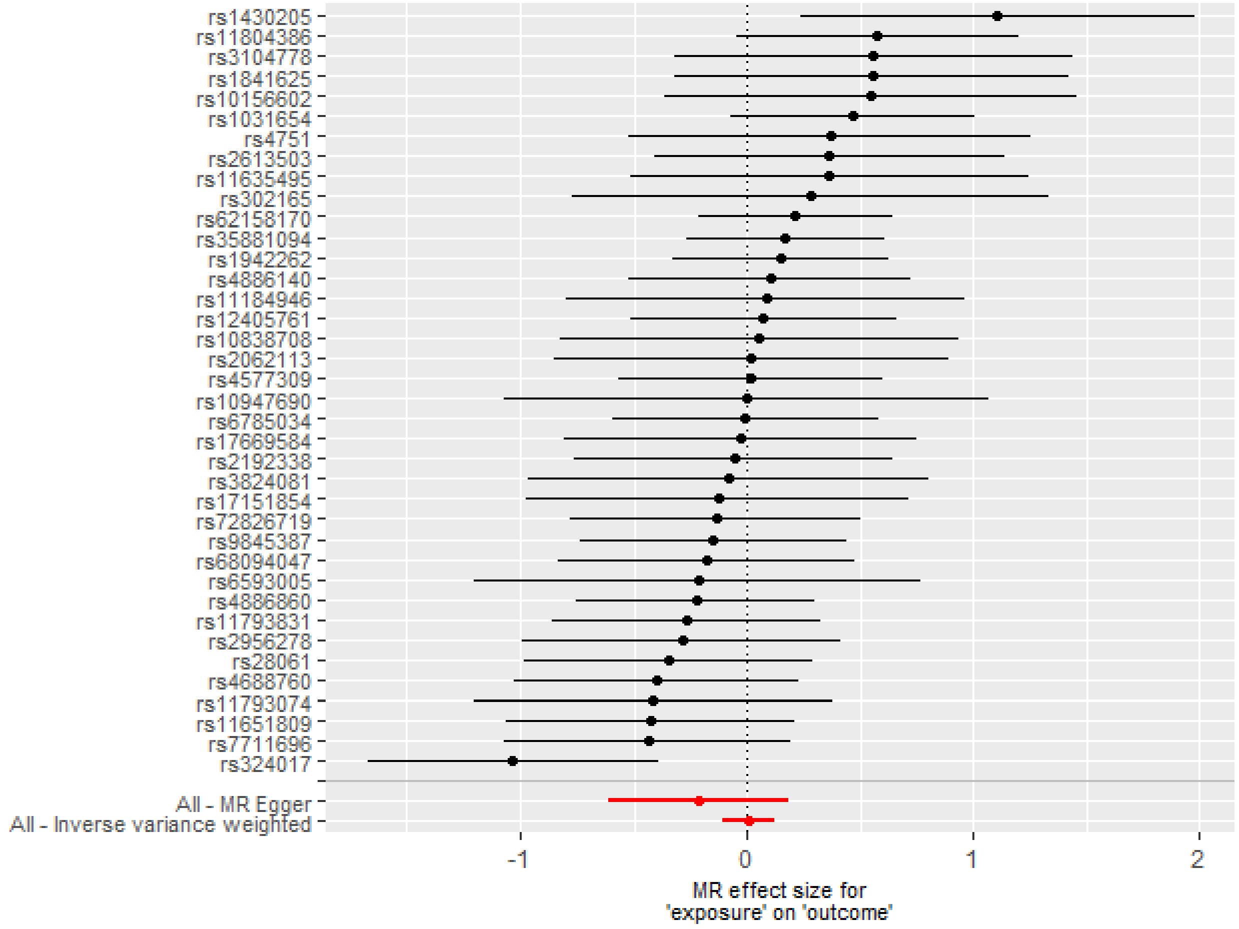

Abbreviations: MR: Mendelian randomization

Supplementary Figure S74. Forest plot of any insomnia symptoms [Lane *et al.* (2019)] and colon cancer association

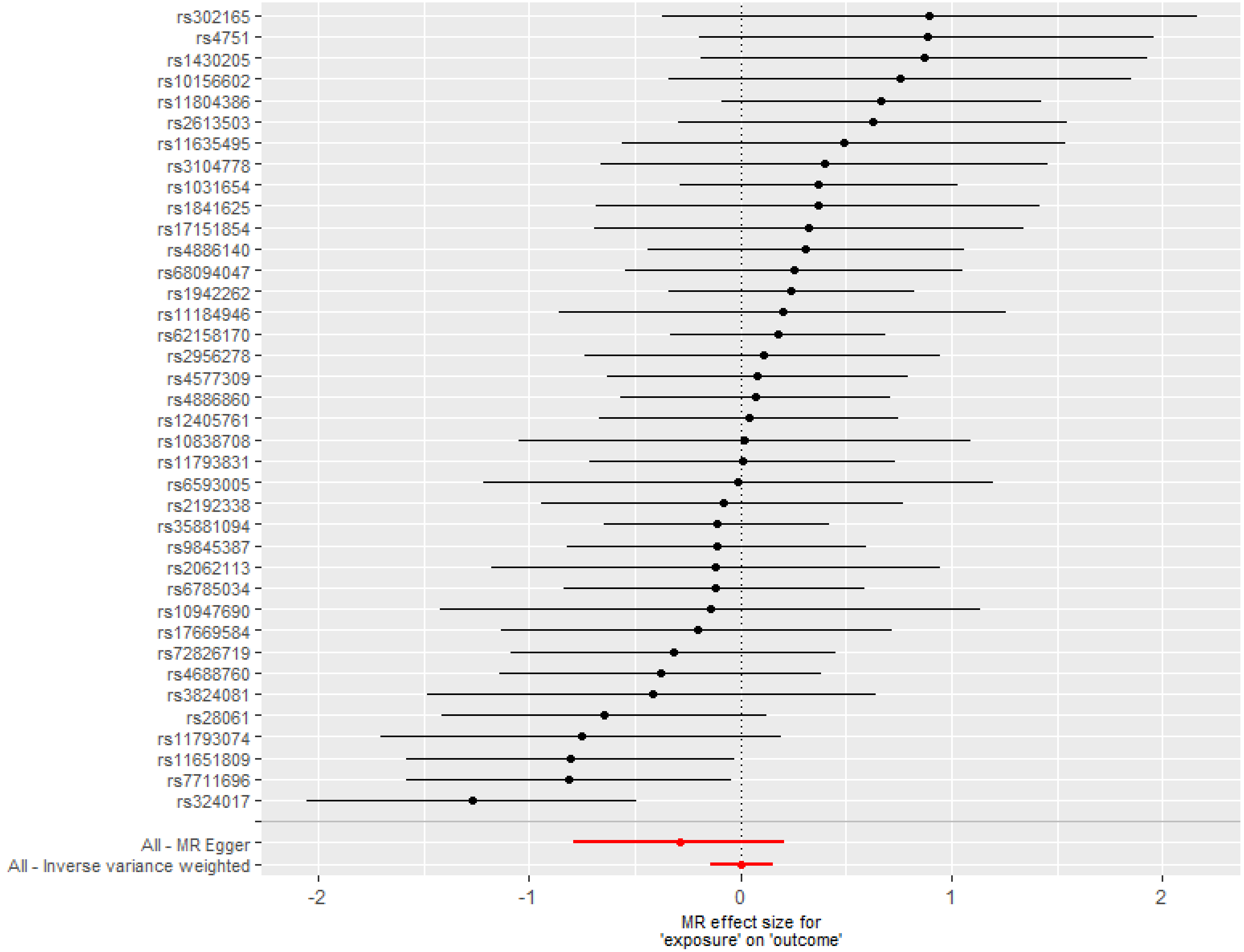

Abbreviations: MR: Mendelian randomization

Supplementary Figure S75. Forest plot of any insomnia symptoms [Lane *et al.* (2019)] and proximal colon cancer association

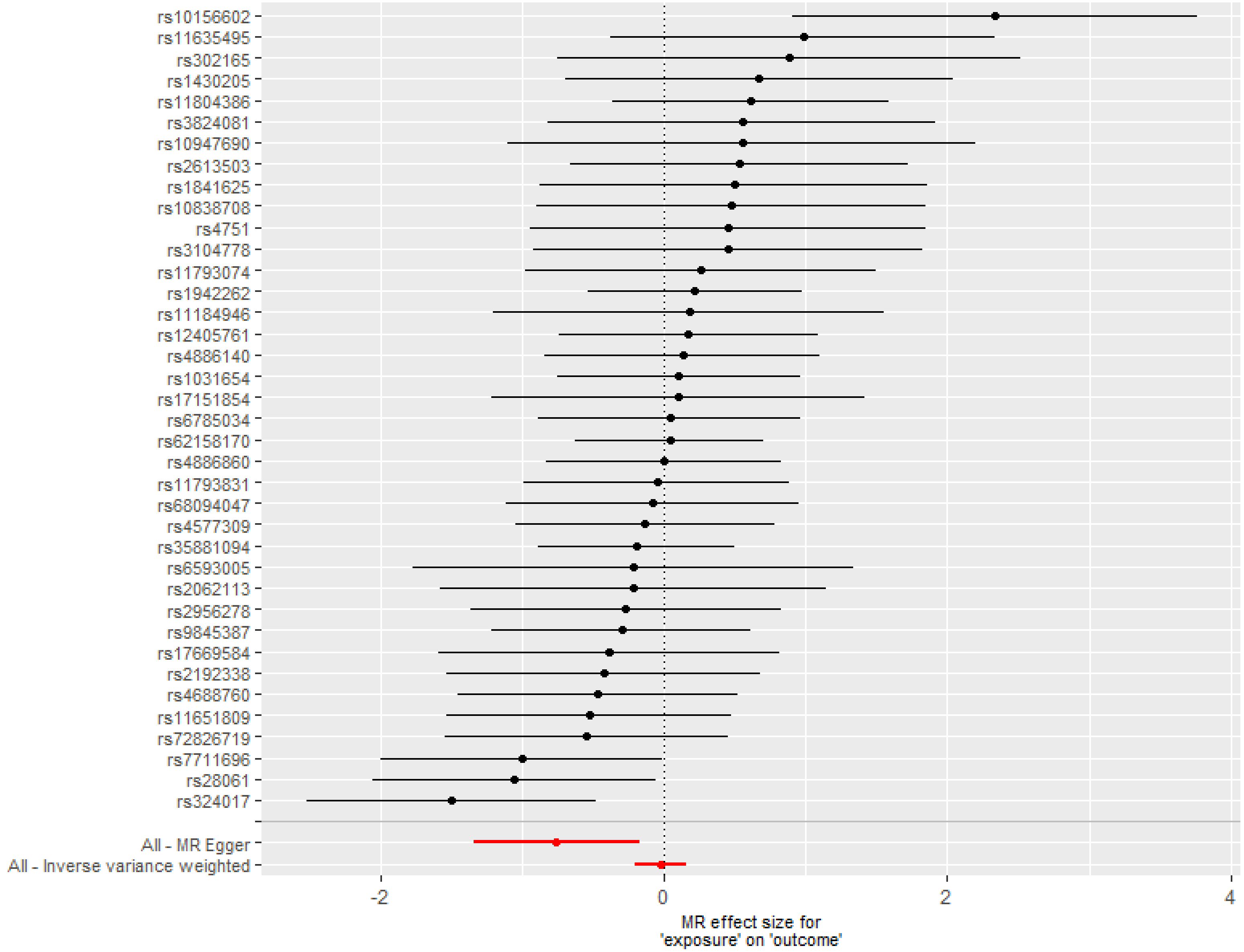

Abbreviations: MR: Mendelian randomization

Supplementary Figure S76. Forest plot of any insomnia symptoms [Lane *et al.* (2019)] and distal colon cancer association

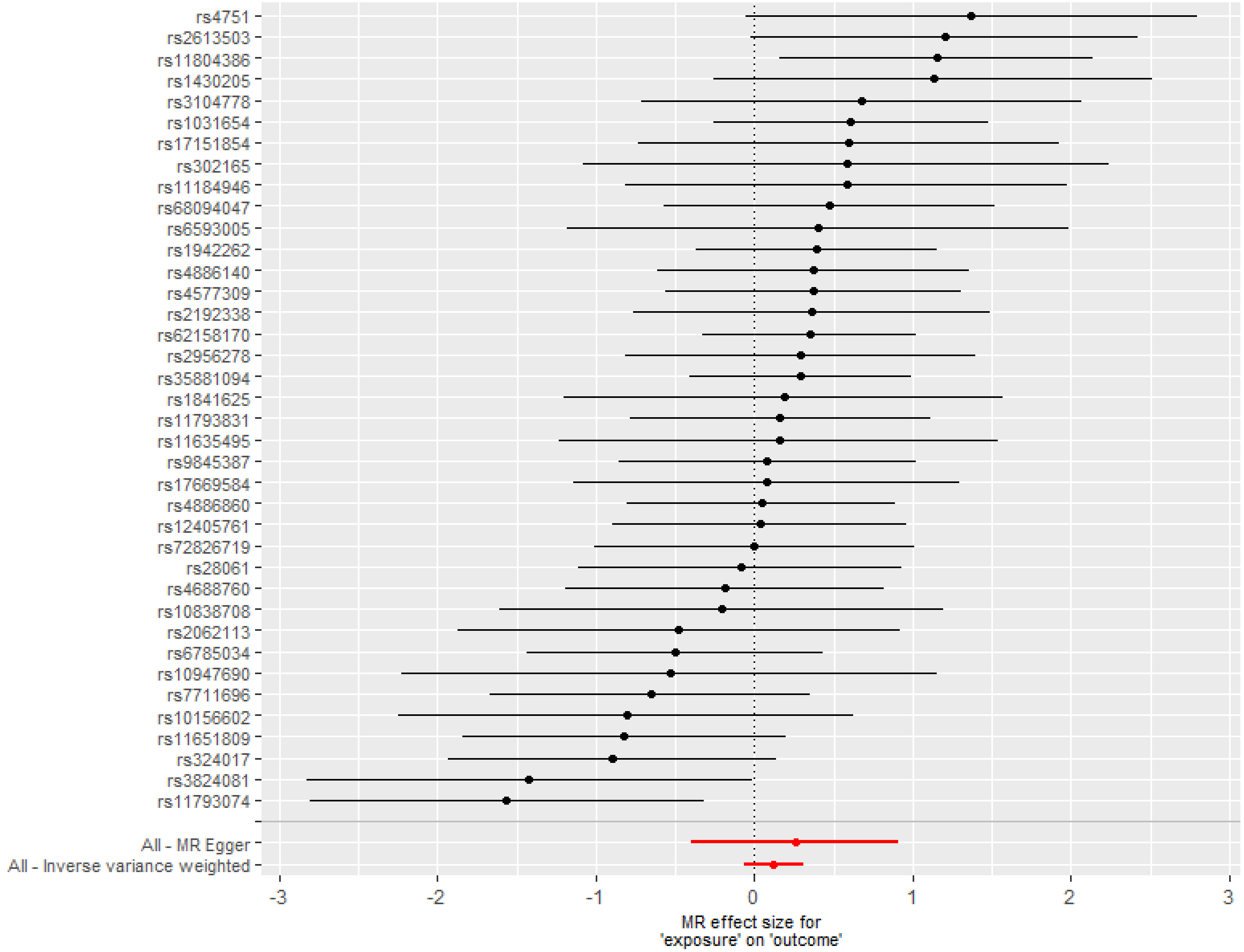

Abbreviations: MR: Mendelian randomization

Supplementary Figure S77. Forest plot of any insomnia symptoms [Lane *et al.* (2019)] and rectal cancer association

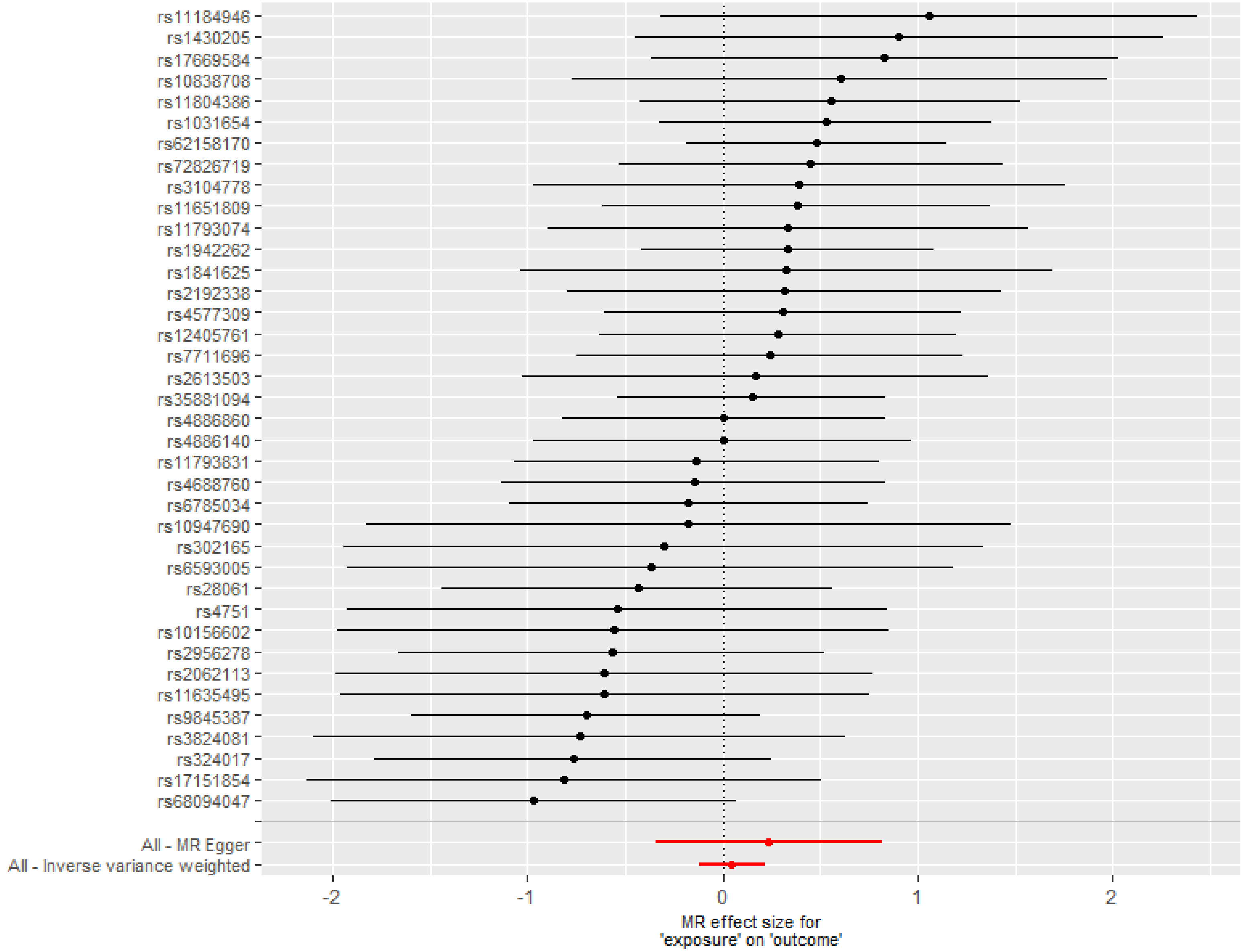

Abbreviations: MR: Mendelian randomization

Supplementary Figure S78. Funnel plot of any insomnia symptoms [Lane *et al.* (2019)] and colorectal cancer association

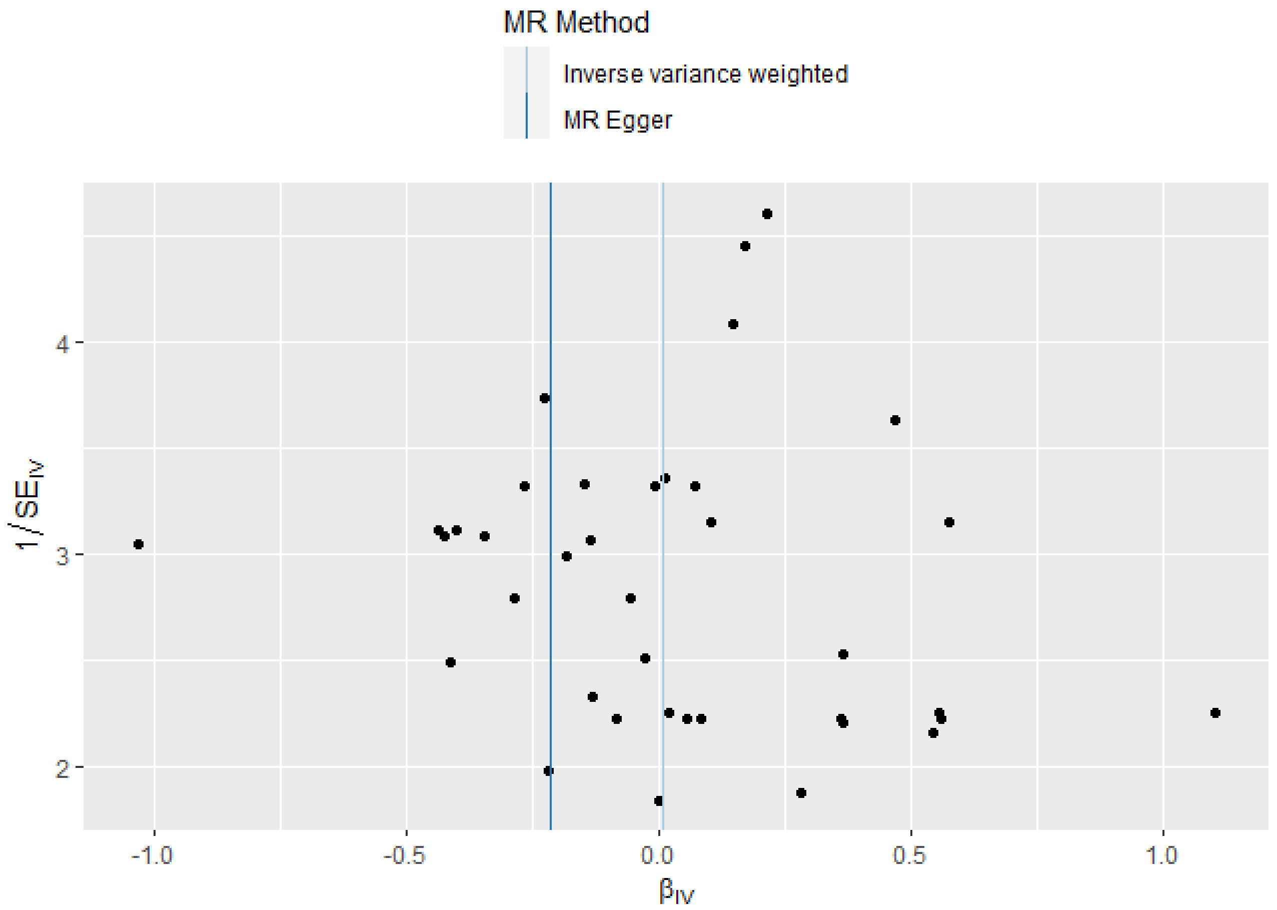

Abbreviations: MR: Mendelian randomization; SE_{IV}: Inverse-variance Standard Error; β_{IV}: Inverse-variance beta coefficient

Supplementary Figure S79. Funnel plot of any insomnia symptoms [Lane *et al.* (2019)] and colon cancer association

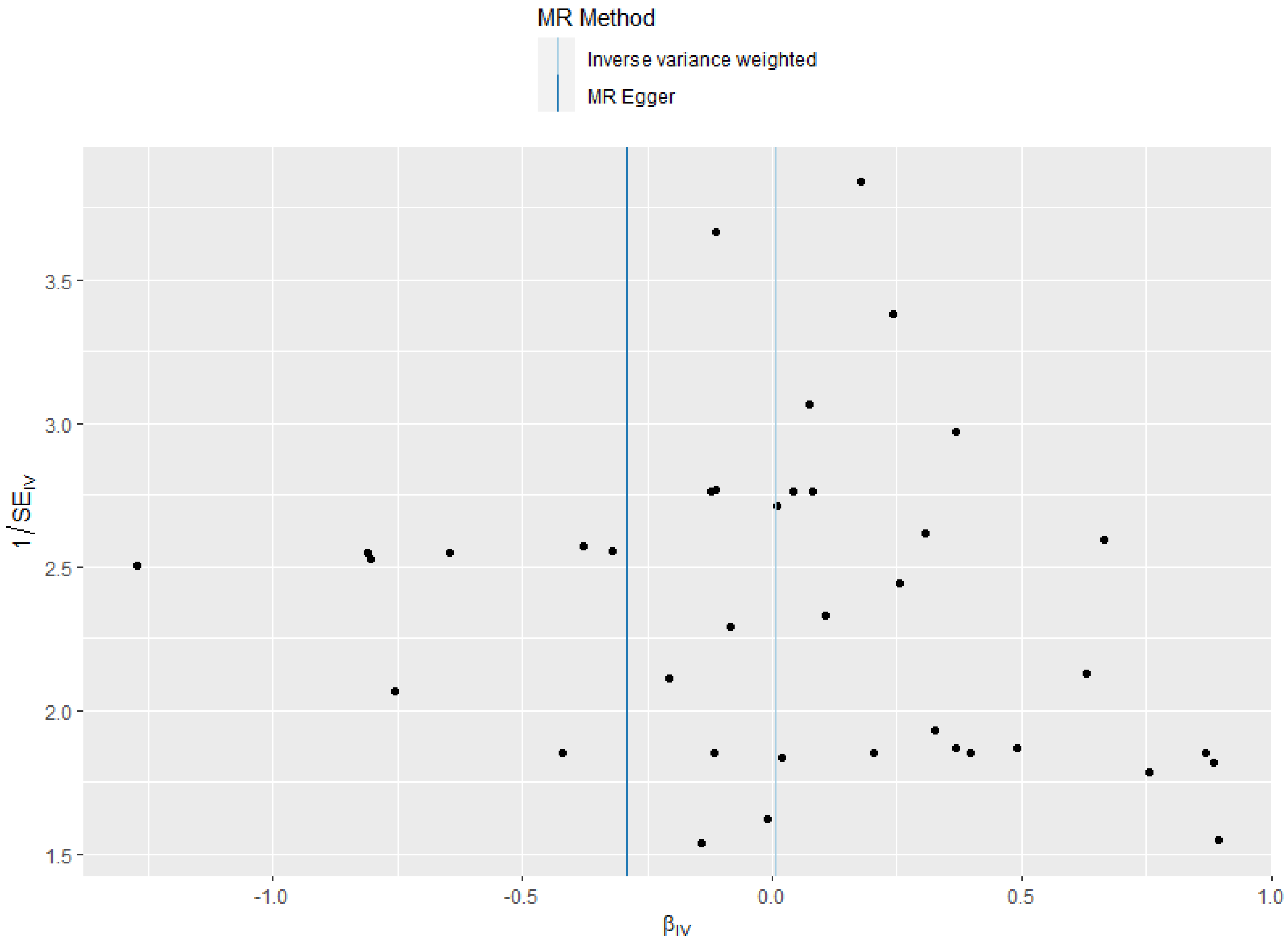

Abbreviations: MR: Mendelian randomization; SE_{IV}: Inverse-variance Standard Error; β_{IV}: Inverse-variance beta coefficient

Supplementary Figure S80. Funnel plot of any insomnia symptoms [Lane *et al.* (2019)] and proximal colon cancer association

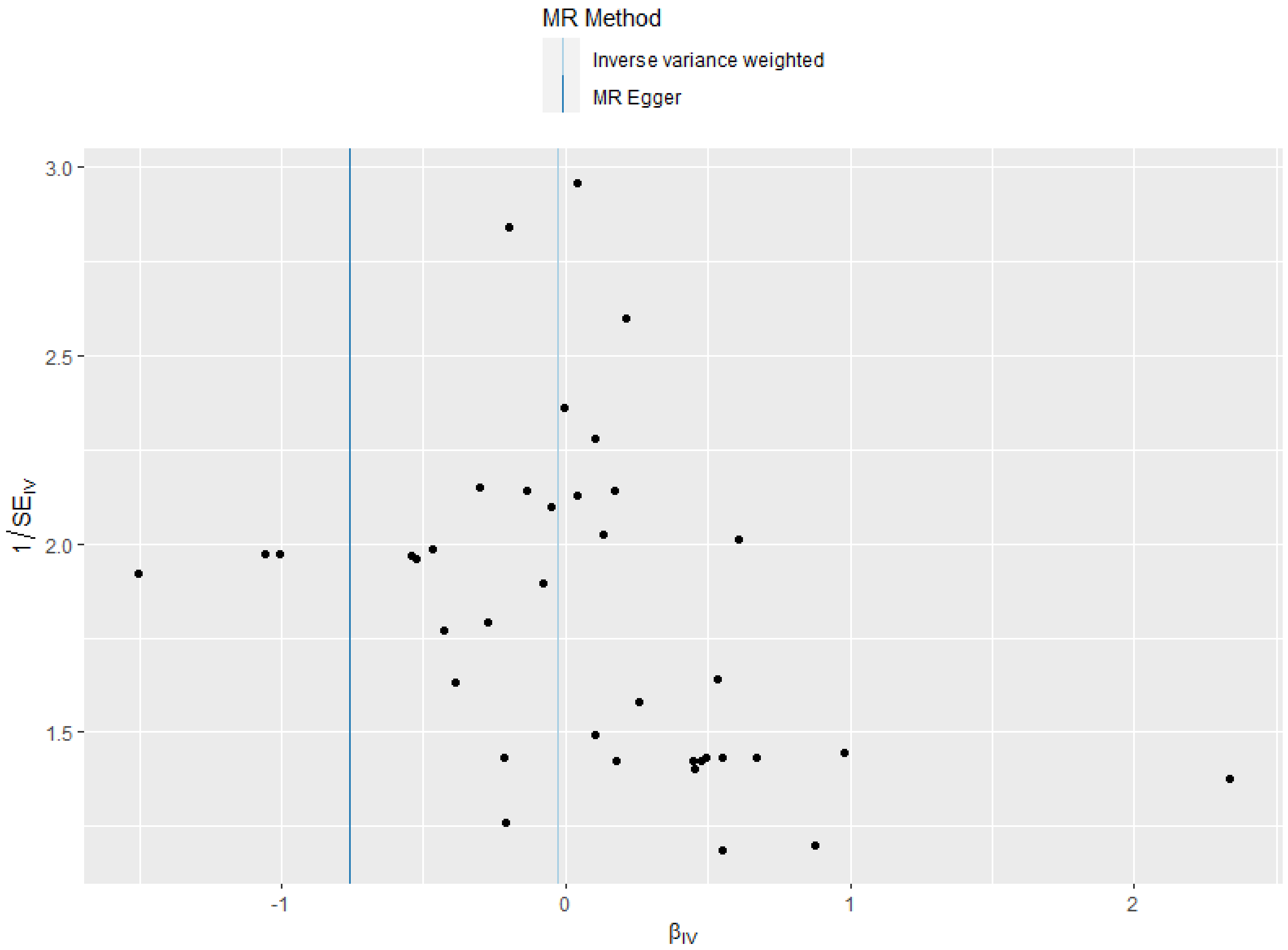

Abbreviations: MR: Mendelian randomization; SE_{IV}: Inverse-variance Standard Error; β_{IV}: Inverse-variance beta coefficient

Supplementary Figure S81. Funnel plot of any insomnia symptoms [Lane *et al.* (2019)] and distal colon cancer association

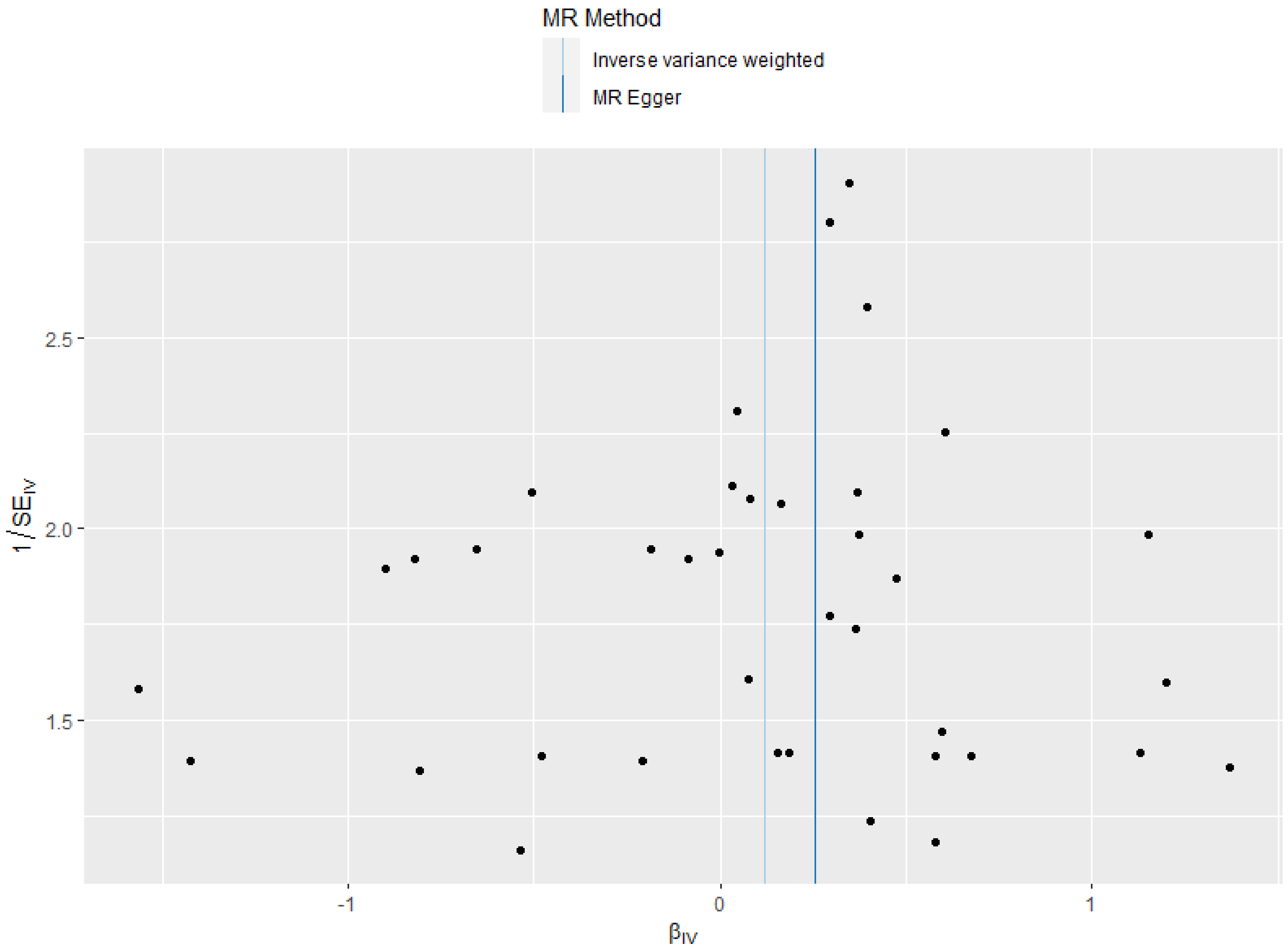

Abbreviations: MR: Mendelian randomization; SE$_{IV}$: Inverse-variance Standard Error; β_{IV}: Inverse-variance beta coefficient

Supplementary Figure S82. Funnel plot of any insomnia symptoms [Lane *et al.* (2019)] and rectal cancer association

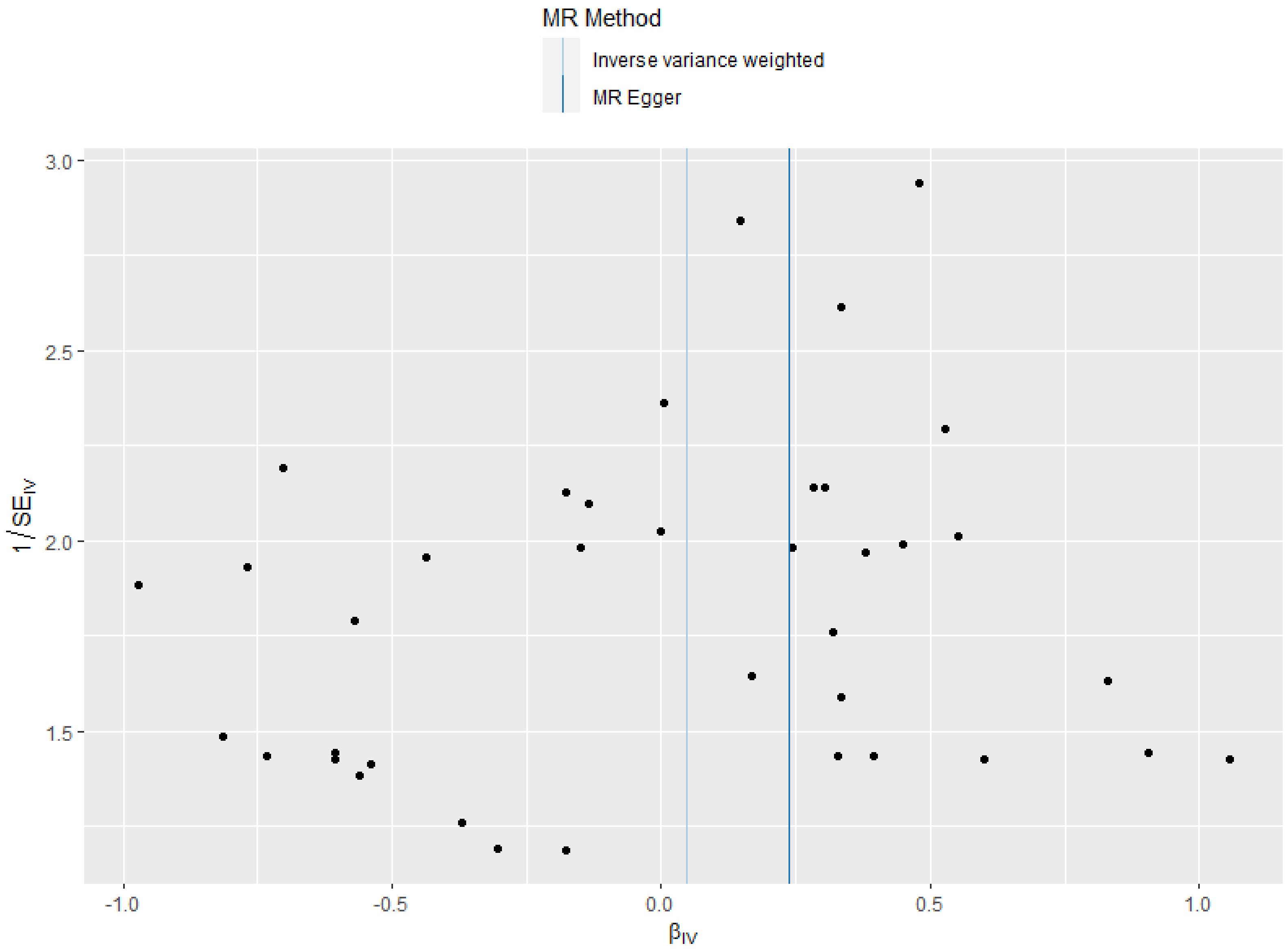

Abbreviations: MR: Mendelian randomization; SE_{IV}: Inverse-variance Standard Error; β_{IV}: Inverse-variance beta coefficient

Primary MR analyses: Insomnia [Jansen *et al.* (2019)]

Supplementary Figure S83. Scatter plot of frequent insomnia symptoms [Jansen *et al.* (2019)] and colorectal cancer association in males

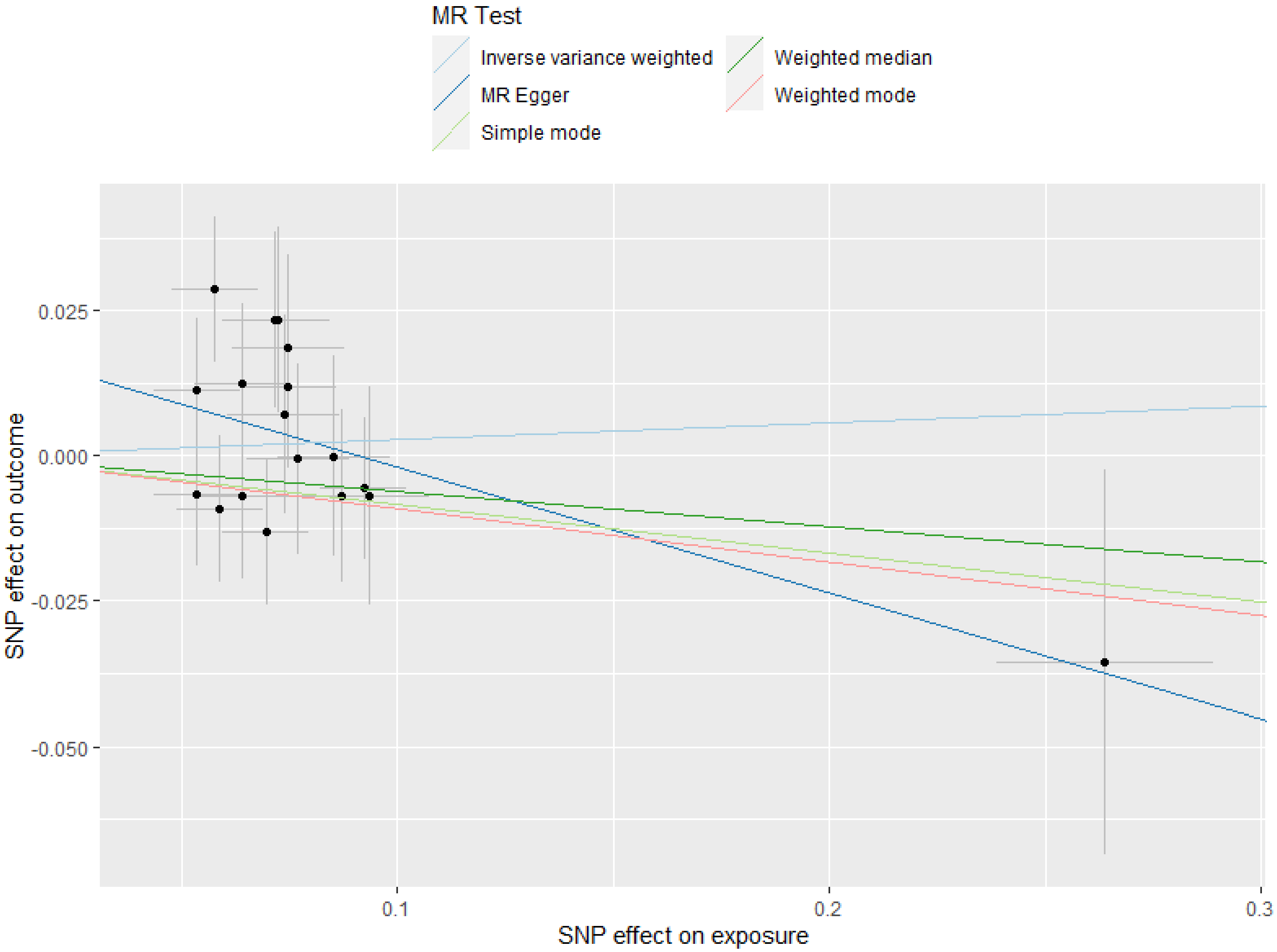

Abbreviations: MR: Mendelian randomization; SNP: Single Nucleotide Polymorphism

Supplementary Figure S84. Scatter plot of frequent insomnia symptoms [Jansen *et al.* (2019)] and colorectal cancer association in females

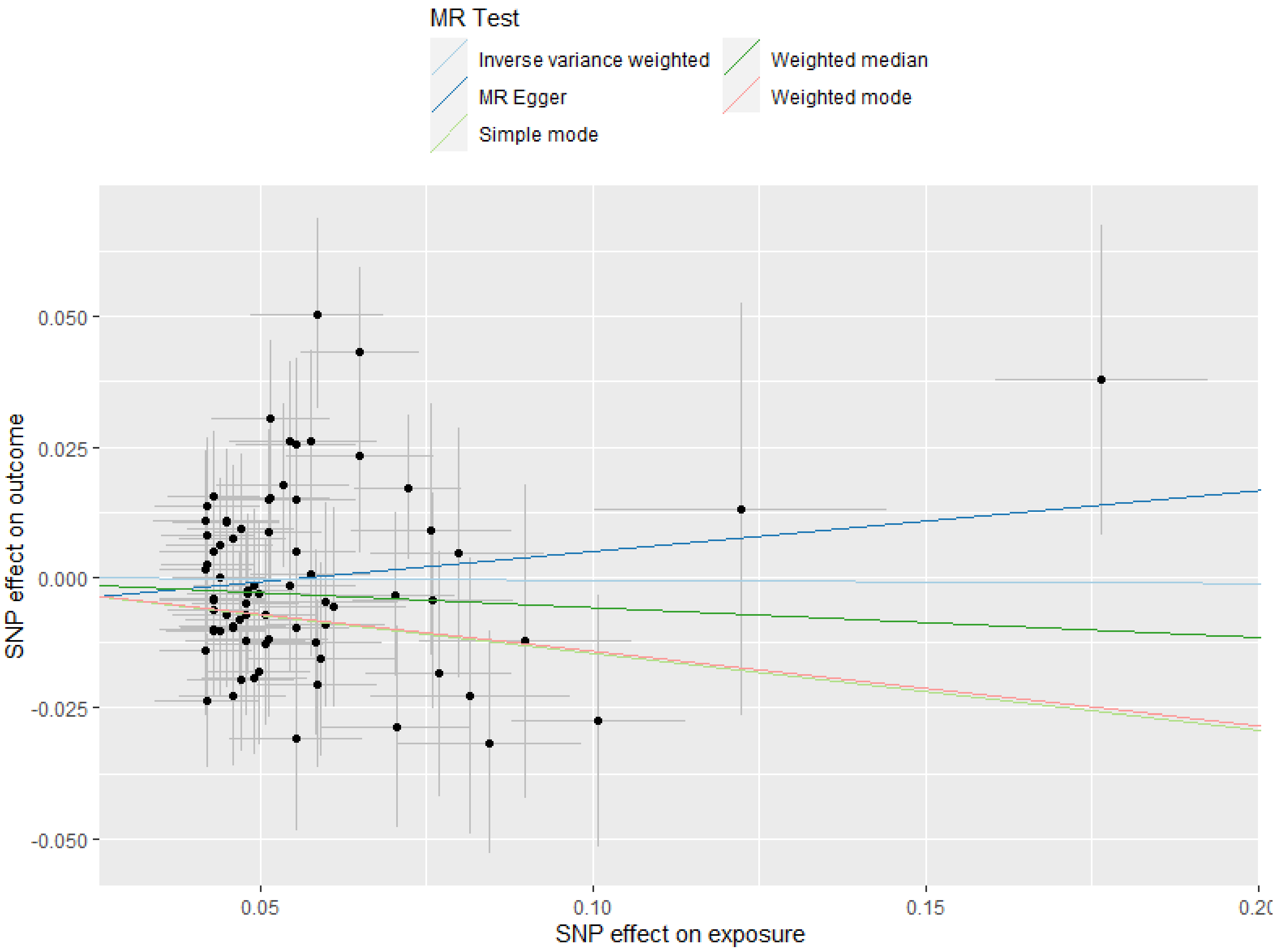

Abbreviations: MR: Mendelian randomization; SNP: Single Nucleotide Polymorphism

Supplementary Figure S85. Scatter plot of frequent insomnia symptoms [Jansen *et al.* (2019)] and colorectal cancer association

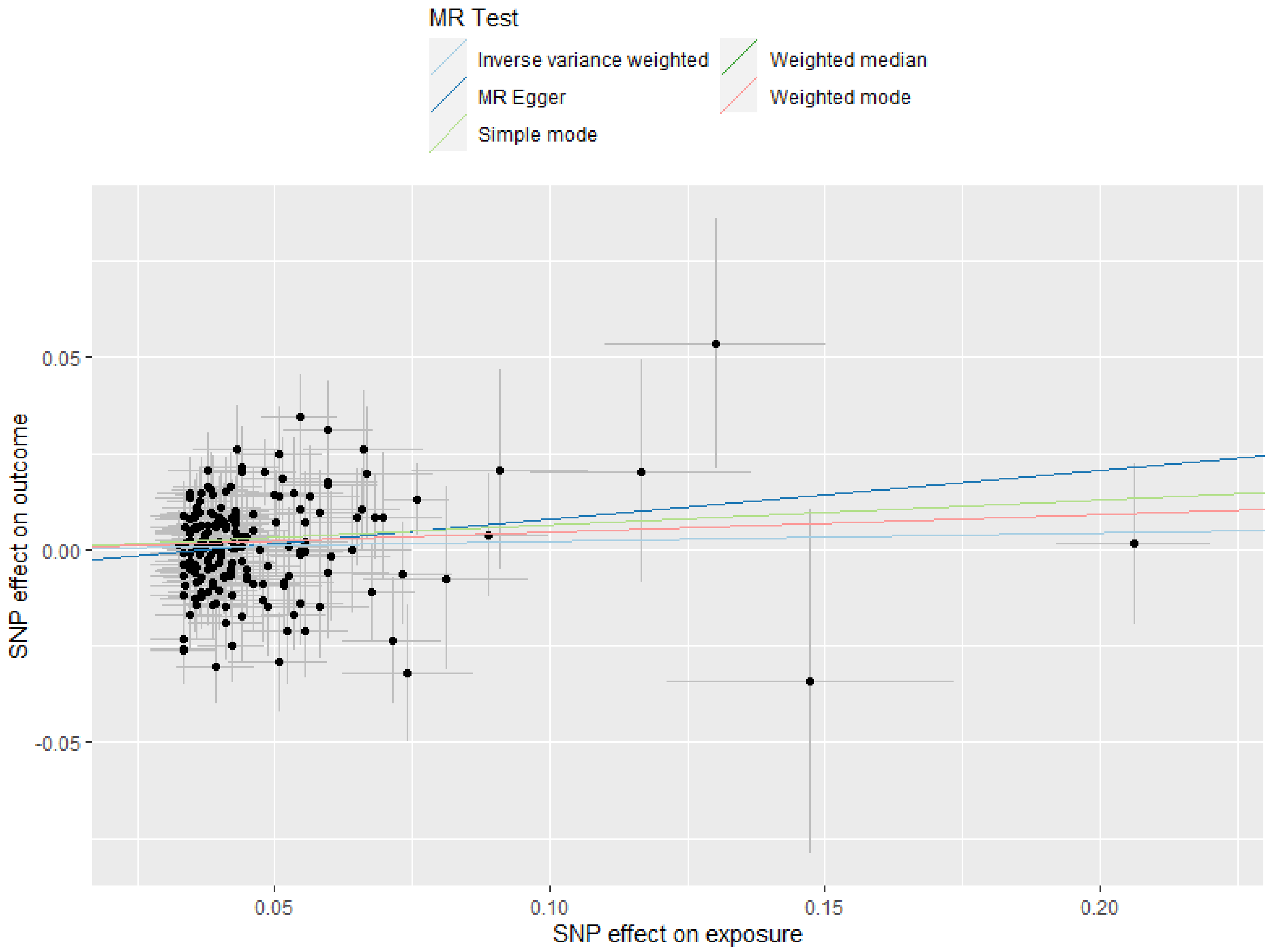

Abbreviations: MR: Mendelian randomization; SNP: Single Nucleotide Polymorphism

Supplementary Figure S86. Scatter plot of frequent insomnia symptoms [Jansen *et al.* (2019)] and colon cancer association in males

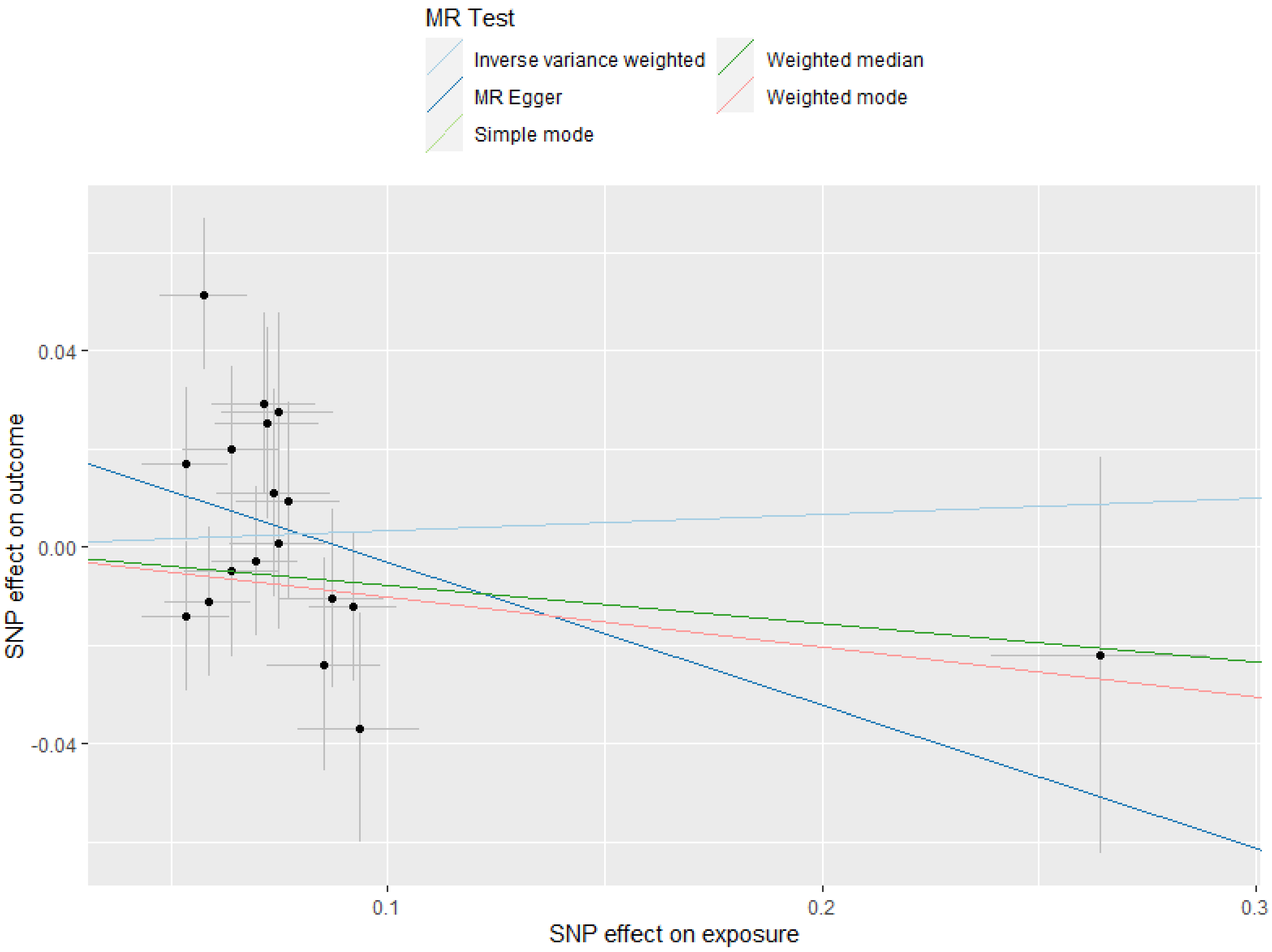

Abbreviations: MR: Mendelian randomization; SNP: Single Nucleotide Polymorphism

Supplementary Figure S87. Scatter plot of frequent insomnia symptoms [Jansen *et al.* (2019)] and colon cancer association in females

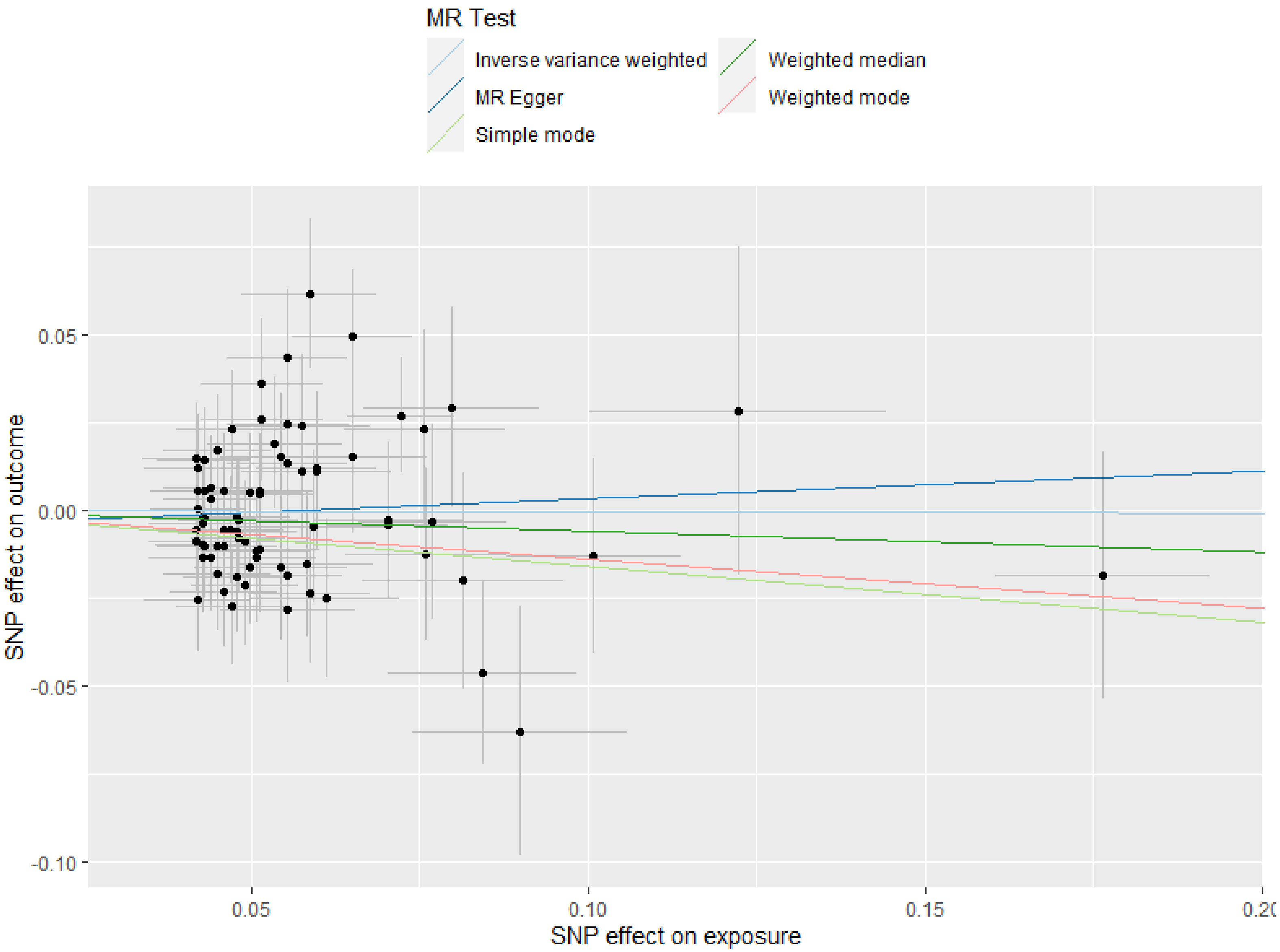

Abbreviations: MR: Mendelian randomization; SNP: Single Nucleotide Polymorphism

Supplementary Figure S88. Scatter plot of frequent insomnia symptoms [Jansen *et al.* (2019)] and colon cancer association

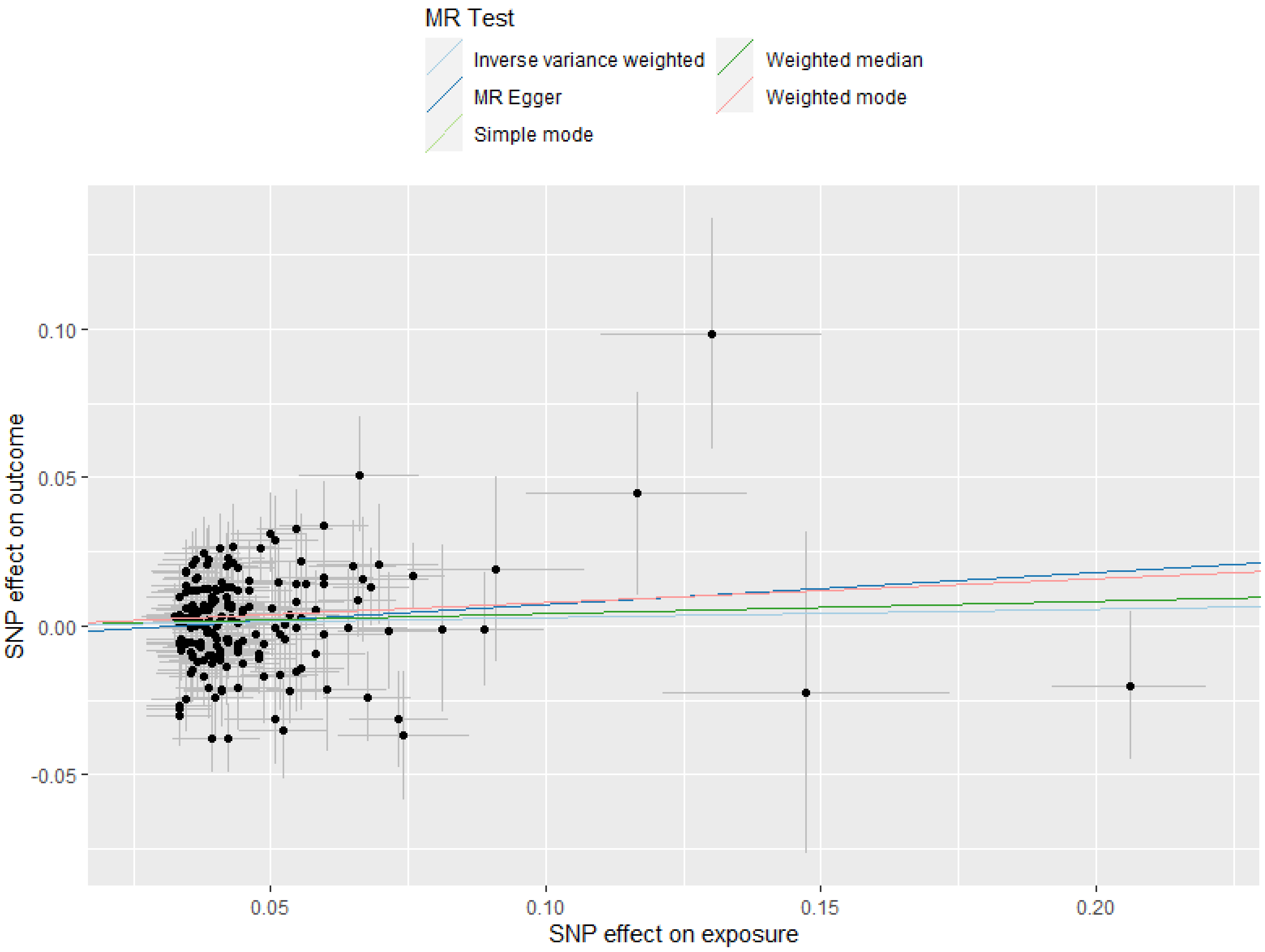

Abbreviations: MR: Mendelian randomization; SNP: Single Nucleotide Polymorphism

Supplementary Figure S89. Scatter plot of frequent insomnia symptoms [Jansen *et al.* (2019)] and proximal colon cancer association

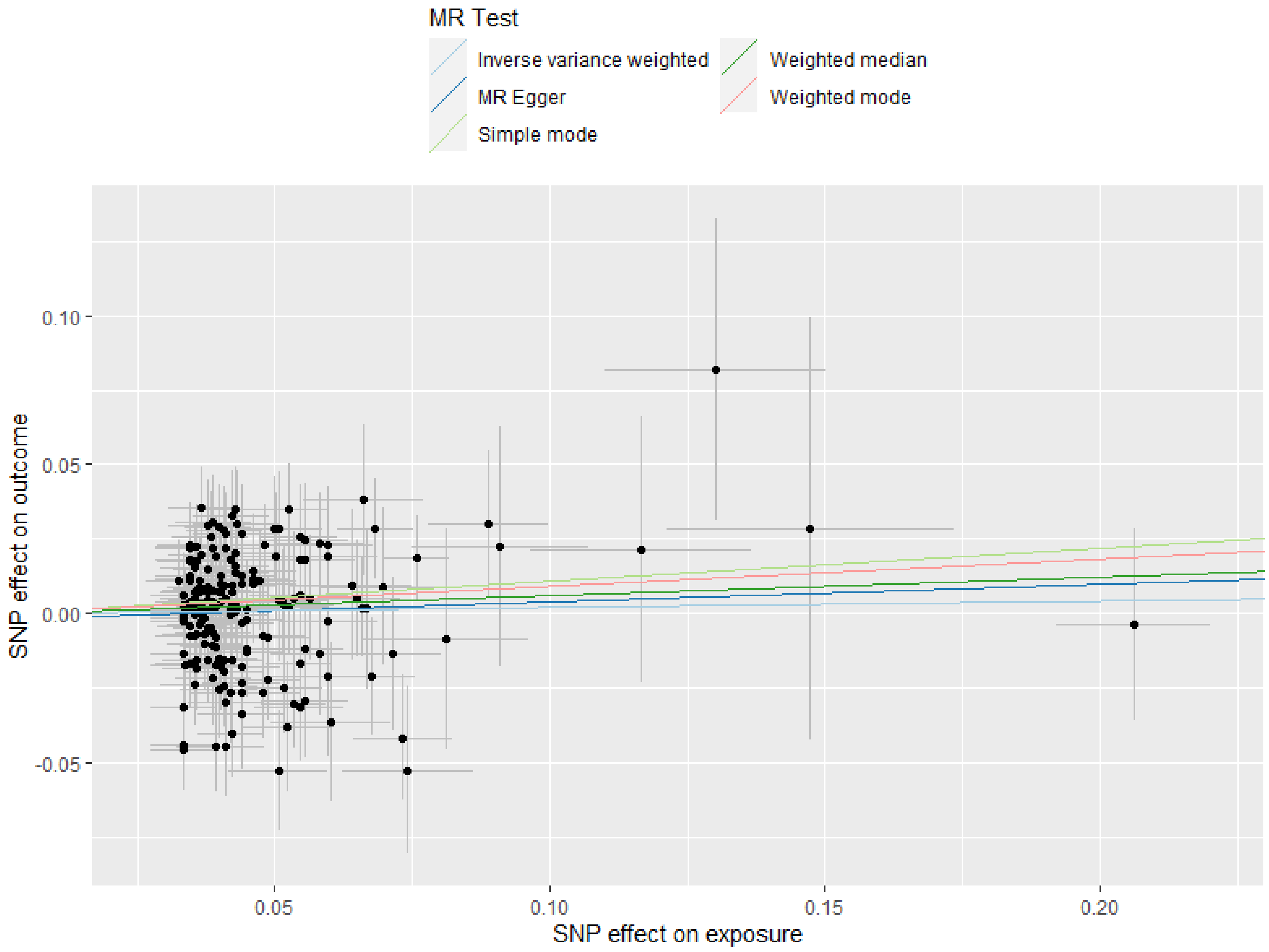

Abbreviations: MR: Mendelian randomization; SNP: Single Nucleotide Polymorphism

Supplementary Figure S90. Scatter plot of frequent insomnia symptoms [Jansen *et al.* (2019)] and distal colon cancer association

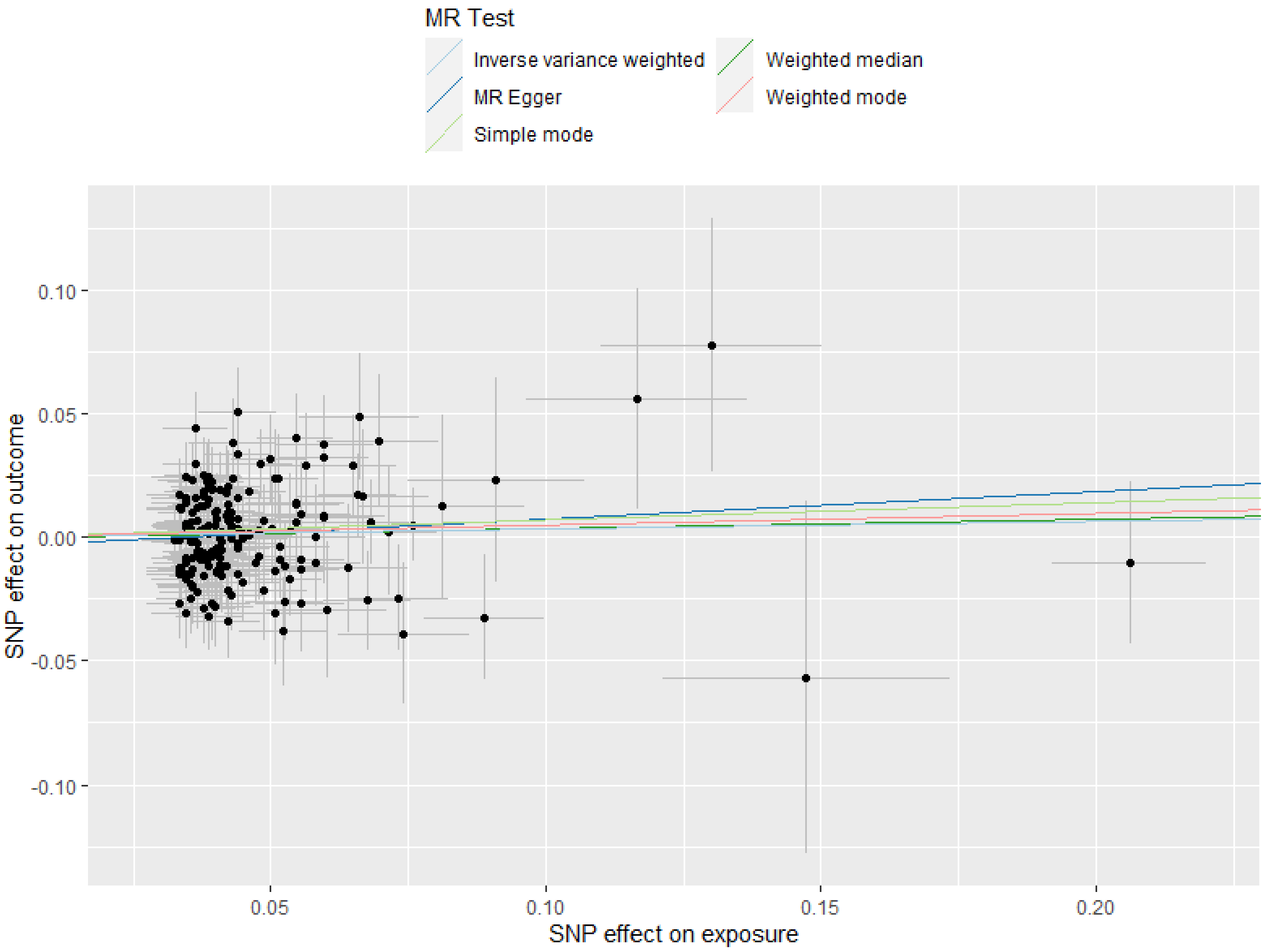

Abbreviations: MR: Mendelian randomization; SNP: Single Nucleotide Polymorphism

Supplementary Figure S91. Scatter plot of frequent insomnia symptoms [Jansen *et al.* (2019)] and rectal cancer association in males

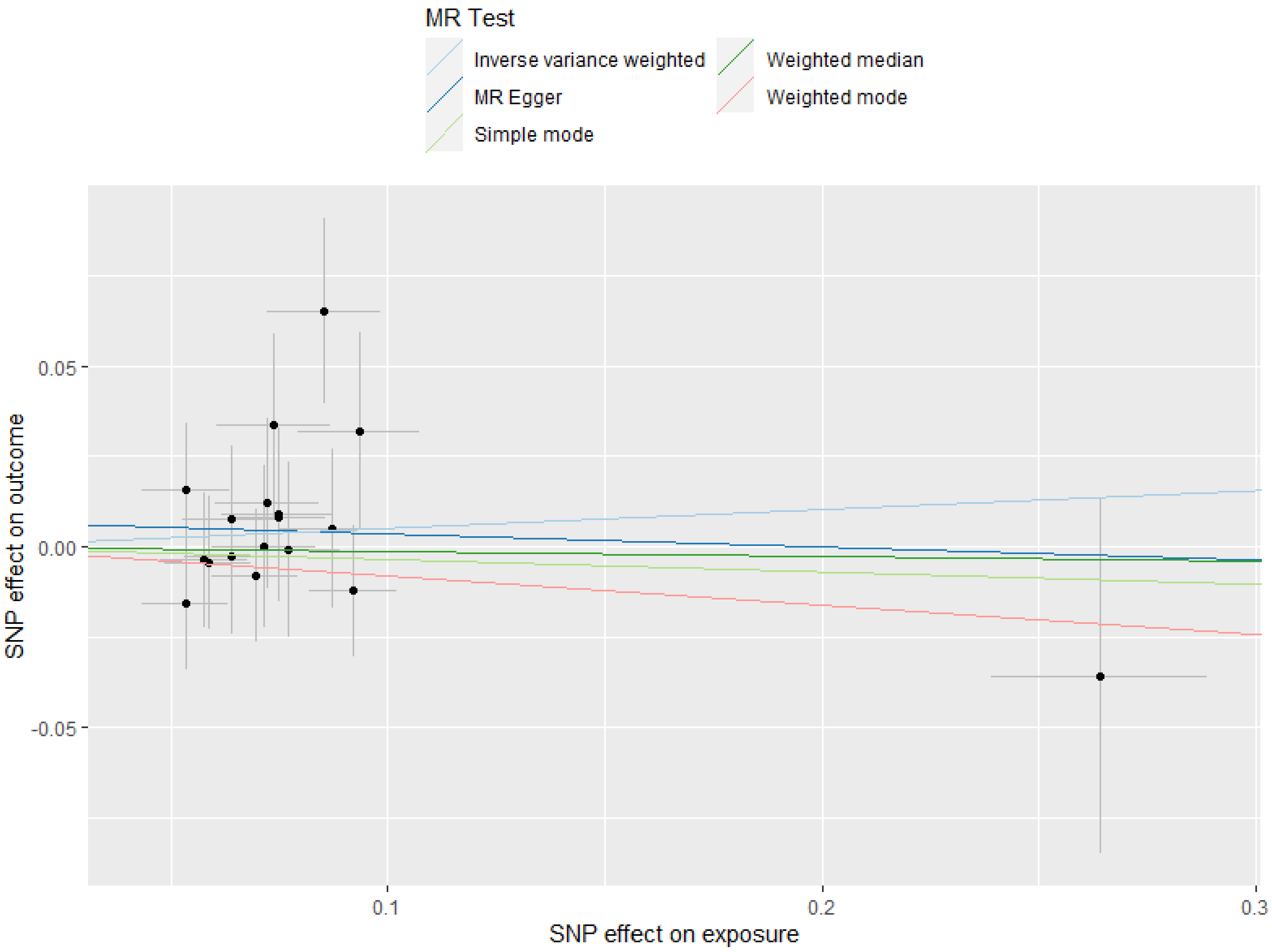

Abbreviations: MR: Mendelian randomization; SNP: Single Nucleotide Polymorphism

Supplementary Figure S92. Scatter plot of frequent insomnia symptoms [Jansen *et al.* (2019)] and rectal cancer association in females

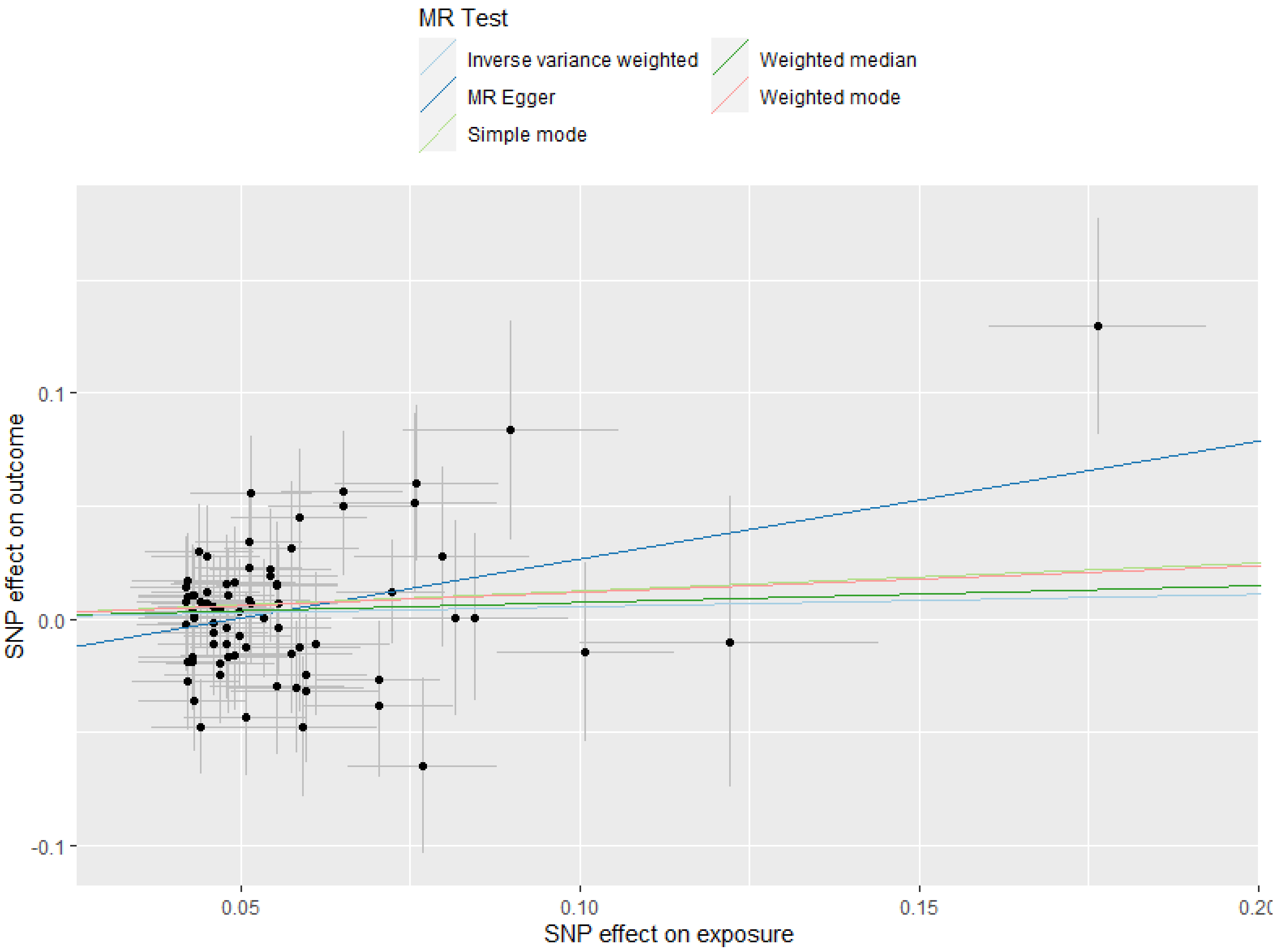

Abbreviations: MR: Mendelian randomization; SNP: Single Nucleotide Polymorphism

Supplementary Figure S93. Scatter plot of frequent insomnia symptoms [Jansen *et al.* (2019)] and rectal cancer association

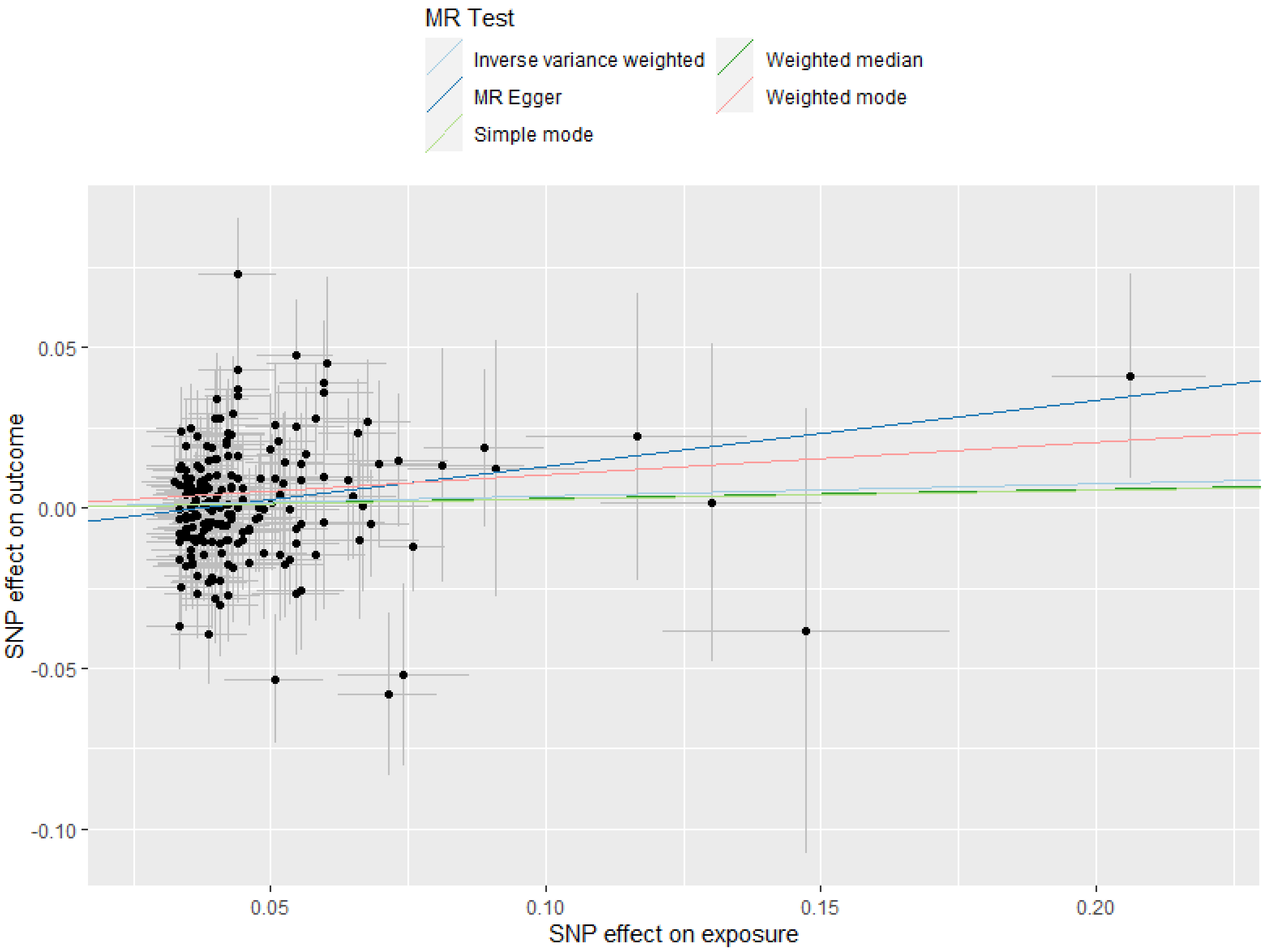

Abbreviations: MR: Mendelian randomization; SNP: Single Nucleotide Polymorphism

Supplementary Figure S94. Forest plot of frequent insomnia symptoms [Jansen *et al.* (2019)] and colorectal cancer association in males

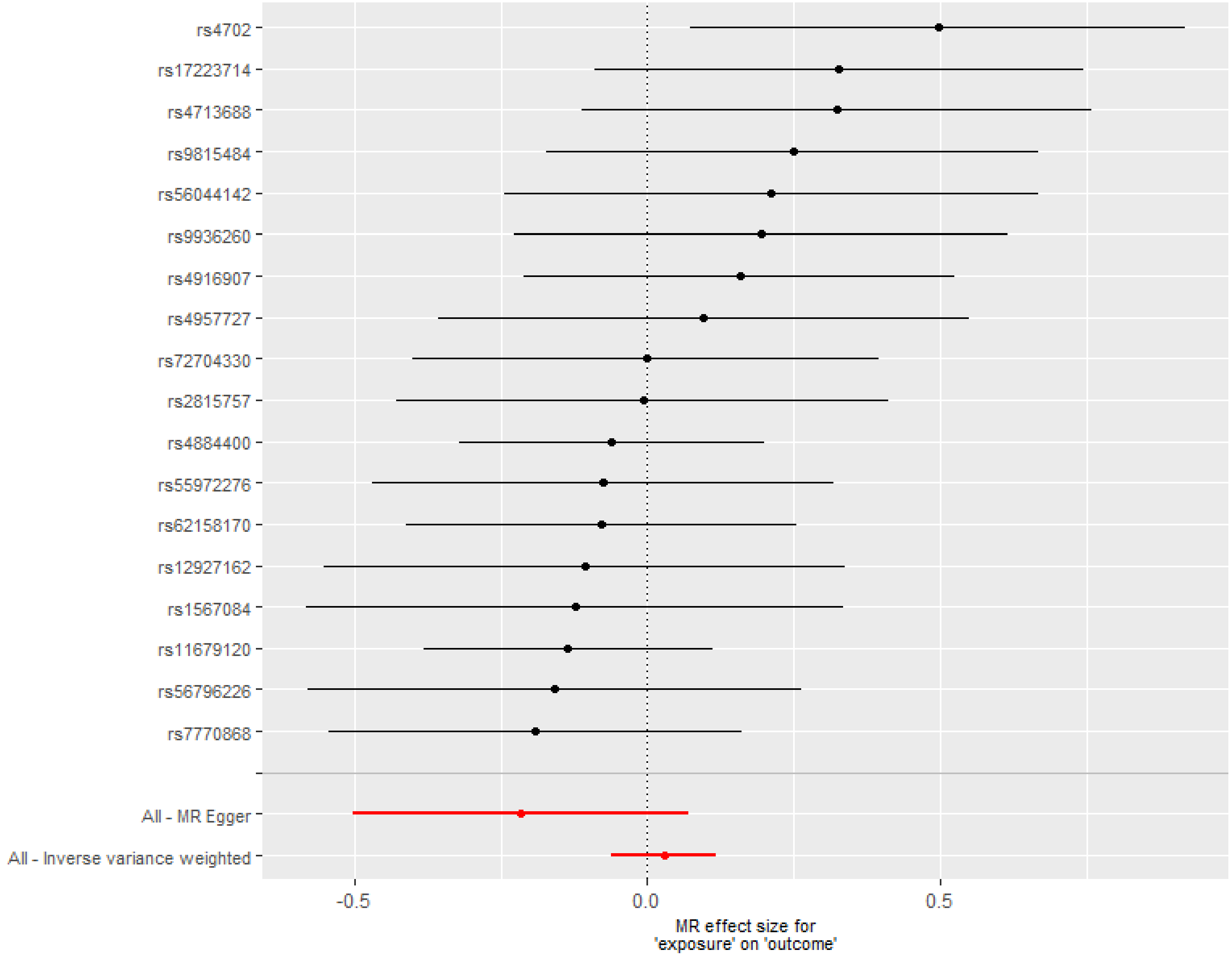

Abbreviations: MR: Mendelian randomization

Supplementary Figure S95. Forest plot of frequent insomnia symptoms [Jansen *et al.* (2019)] and colorectal cancer association in females

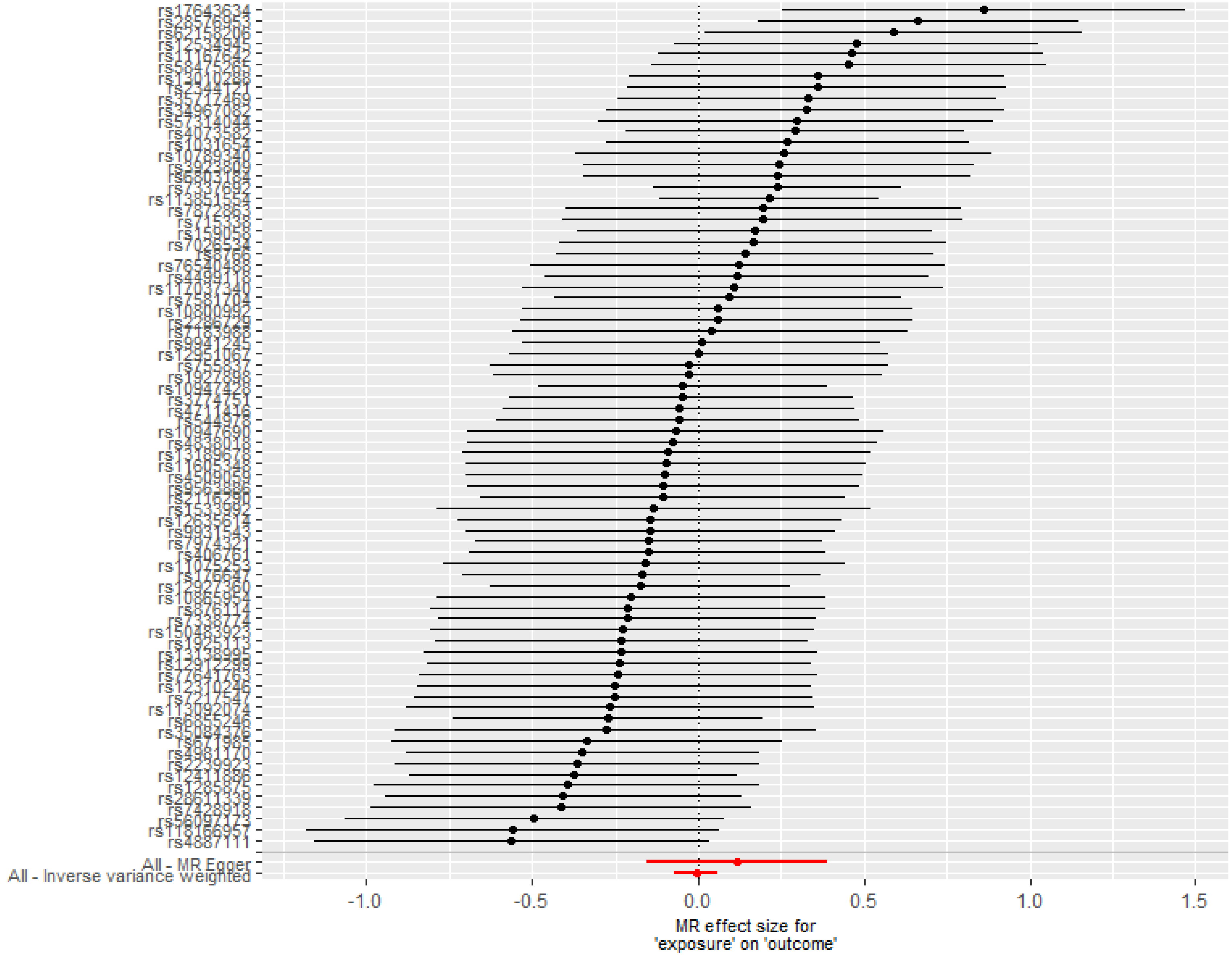

Abbreviations: MR: Mendelian randomization

Supplementary Figure S96. Forest plot of frequent insomnia symptoms [Jansen *et al.* (2019)] and colorectal cancer association

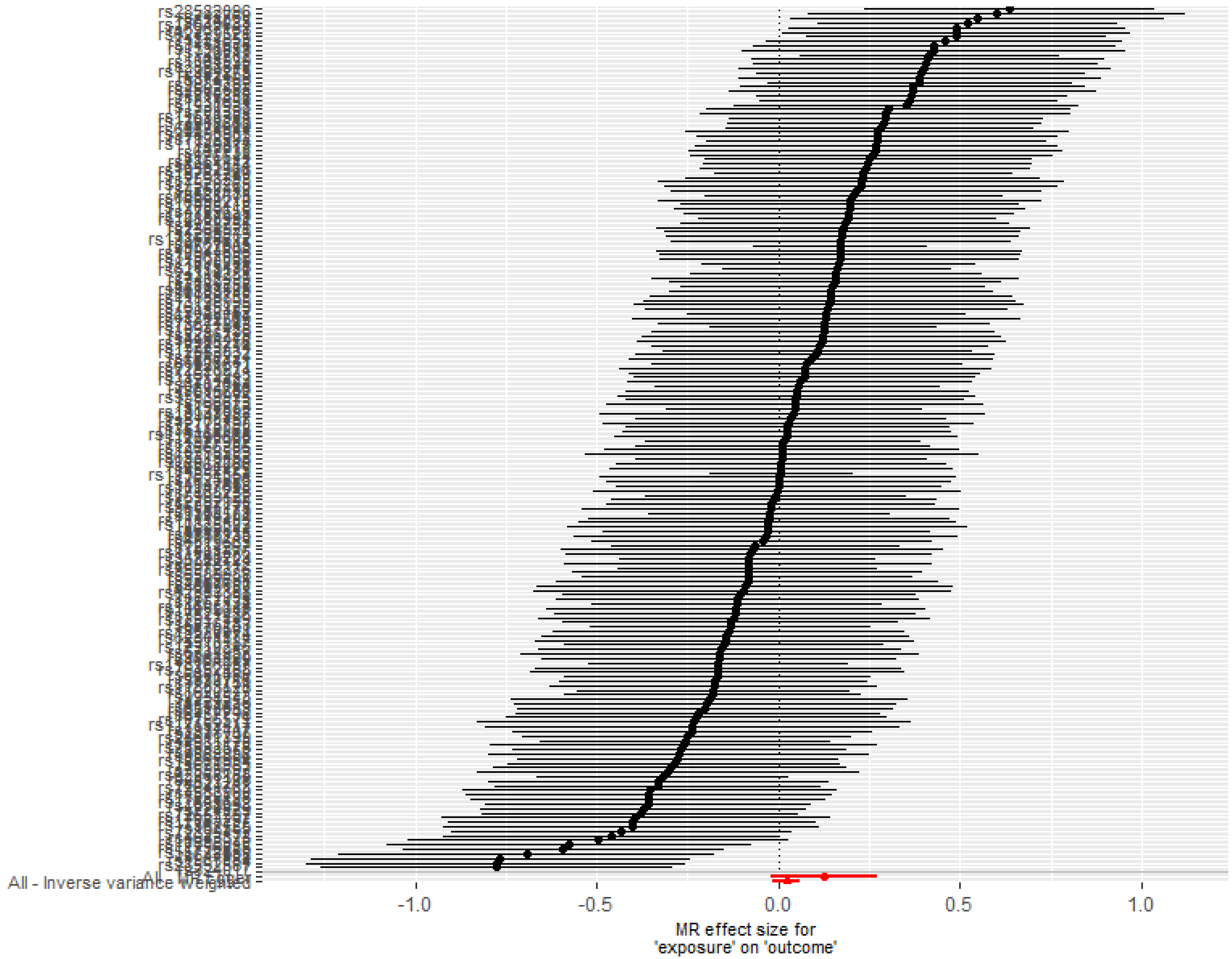

Abbreviations: MR: Mendelian randomization

Supplementary Figure S97. Forest plot of frequent insomnia symptoms [Jansen *et al.* (2019)] and colon cancer association in males

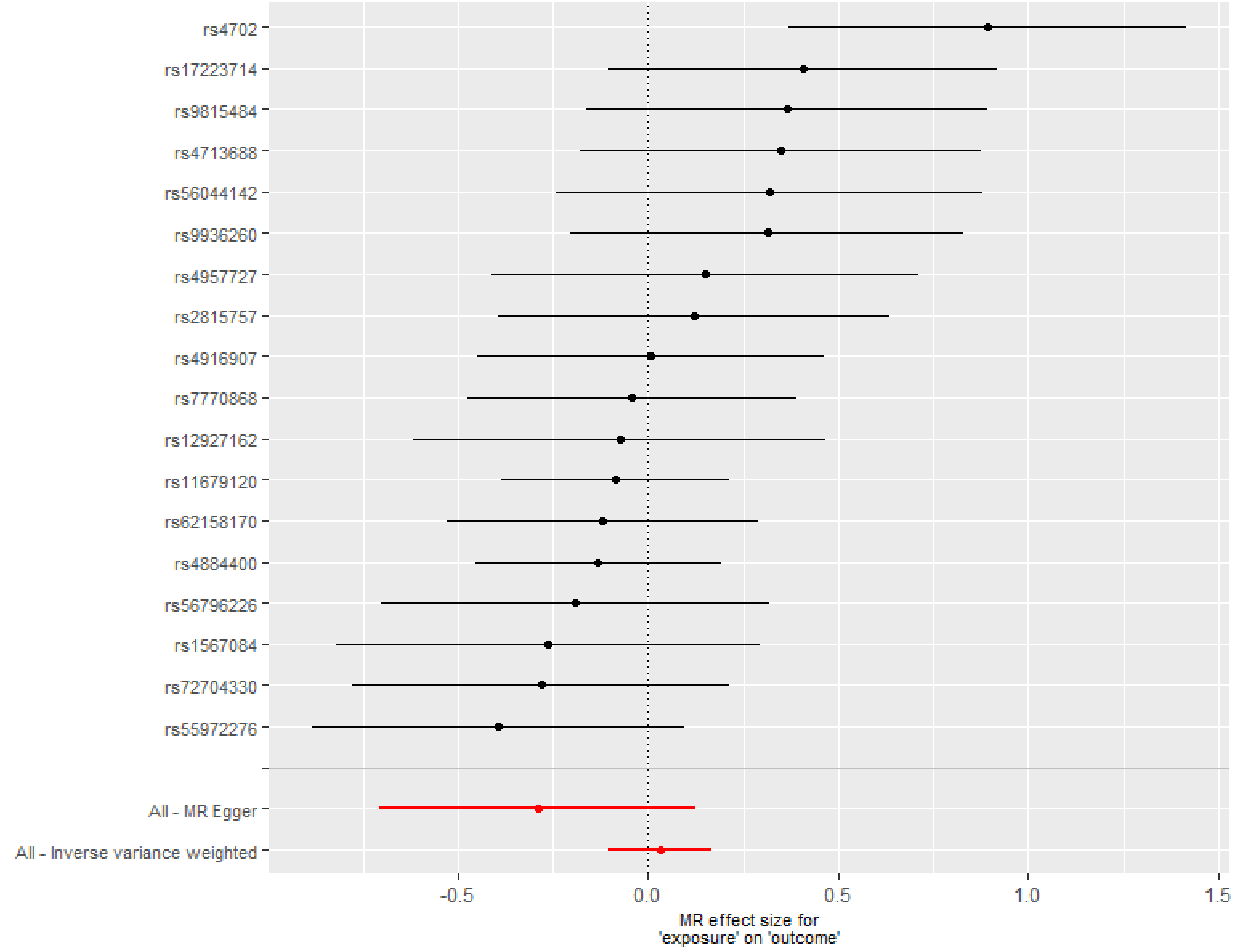

Abbreviations: MR: Mendelian randomization

Supplementary Figure S98. Forest plot of frequent insomnia symptoms [Jansen *et al.* (2019)] and colon cancer association in females

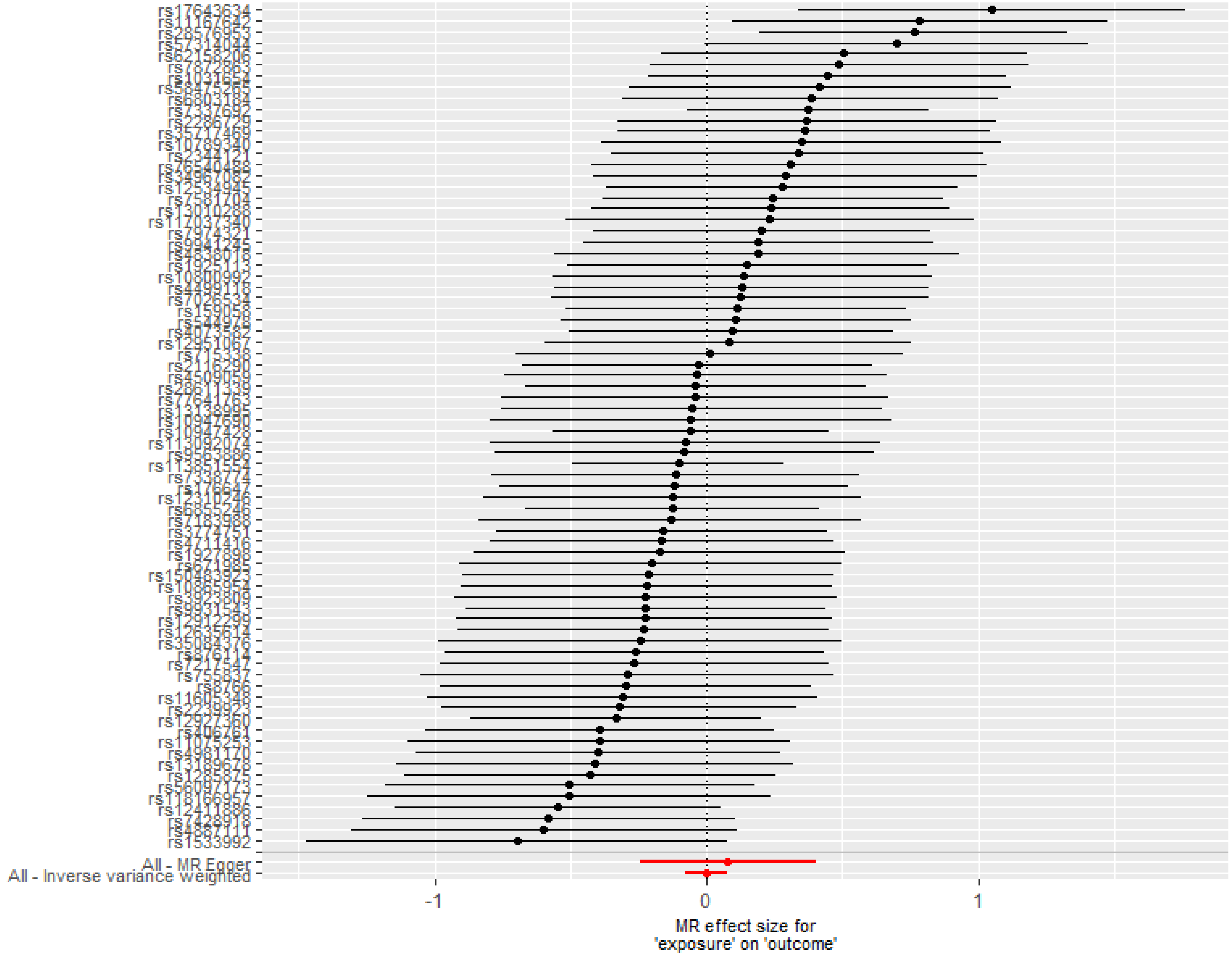

Abbreviations: MR: Mendelian randomization

Supplementary Figure S99. Forest plot of frequent insomnia symptoms [Jansen *et al.* (2019)] and colon cancer association

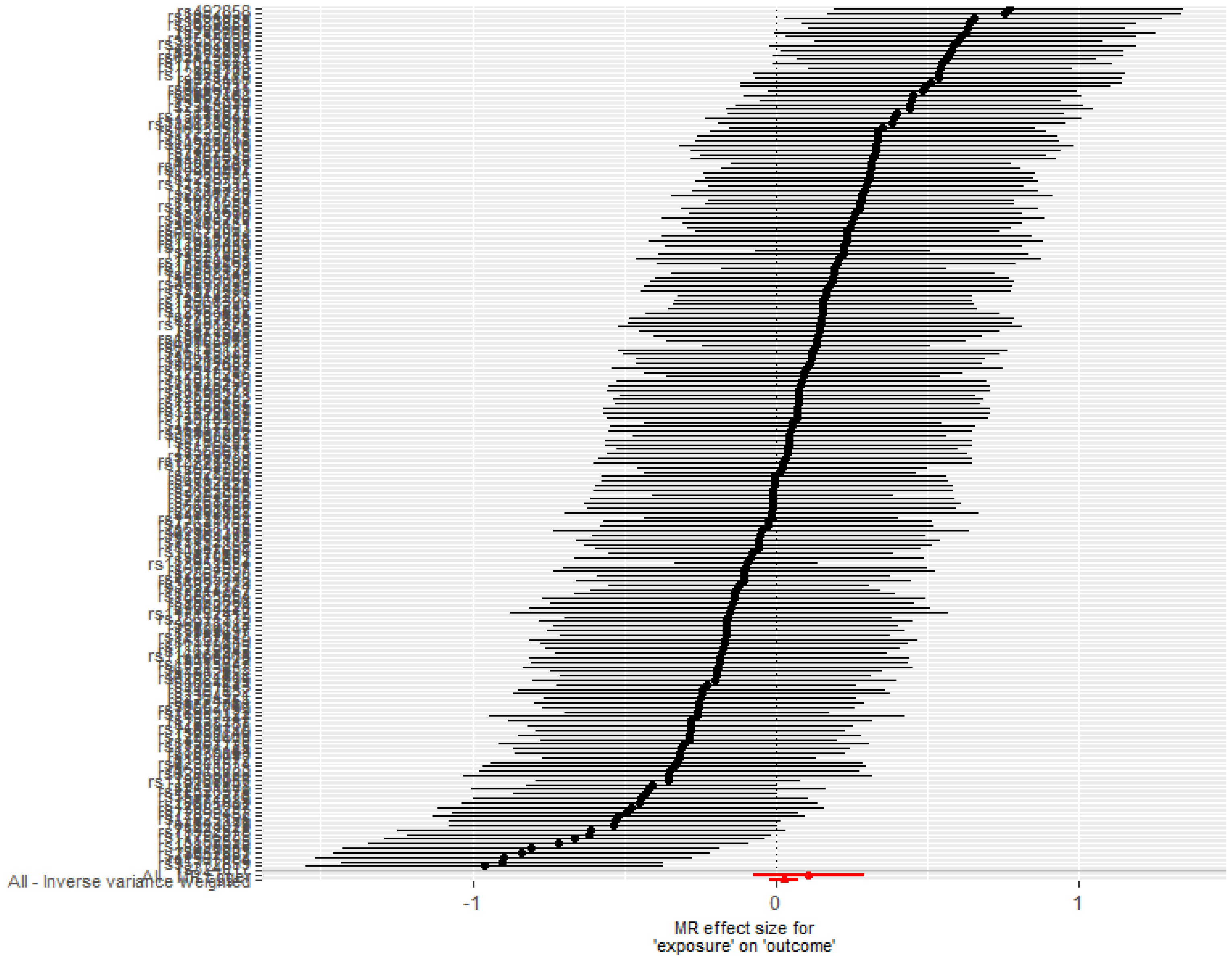

Abbreviations: MR: Mendelian randomization

Supplementary Figure S100. Forest plot of frequent insomnia symptoms [Jansen *et al.* (2019)] and proximal colon cancer association

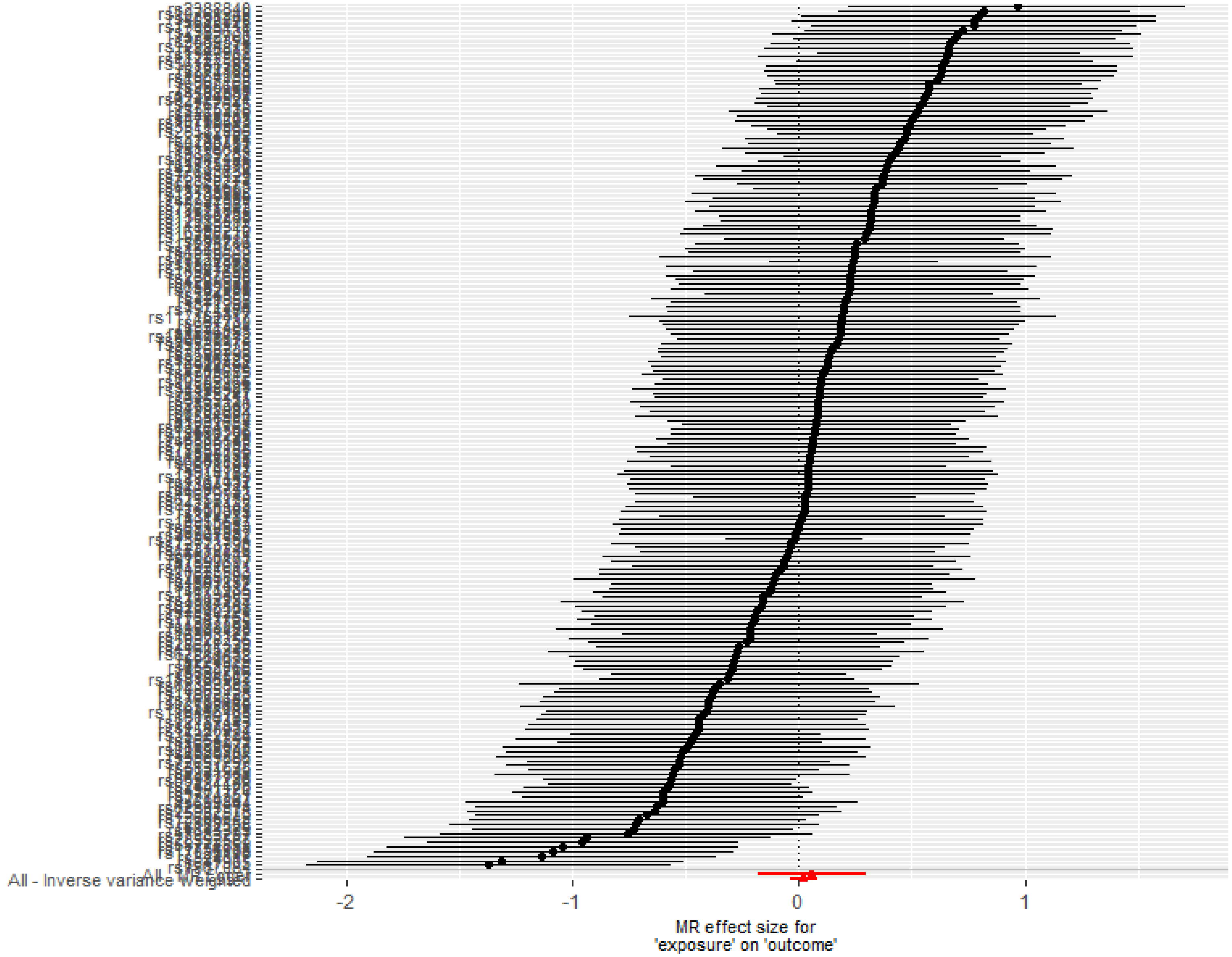

Abbreviations: MR: Mendelian randomization

Supplementary Figure S101. Forest plot of frequent insomnia symptoms [Jansen *et al.* (2019)] and distal colon cancer association

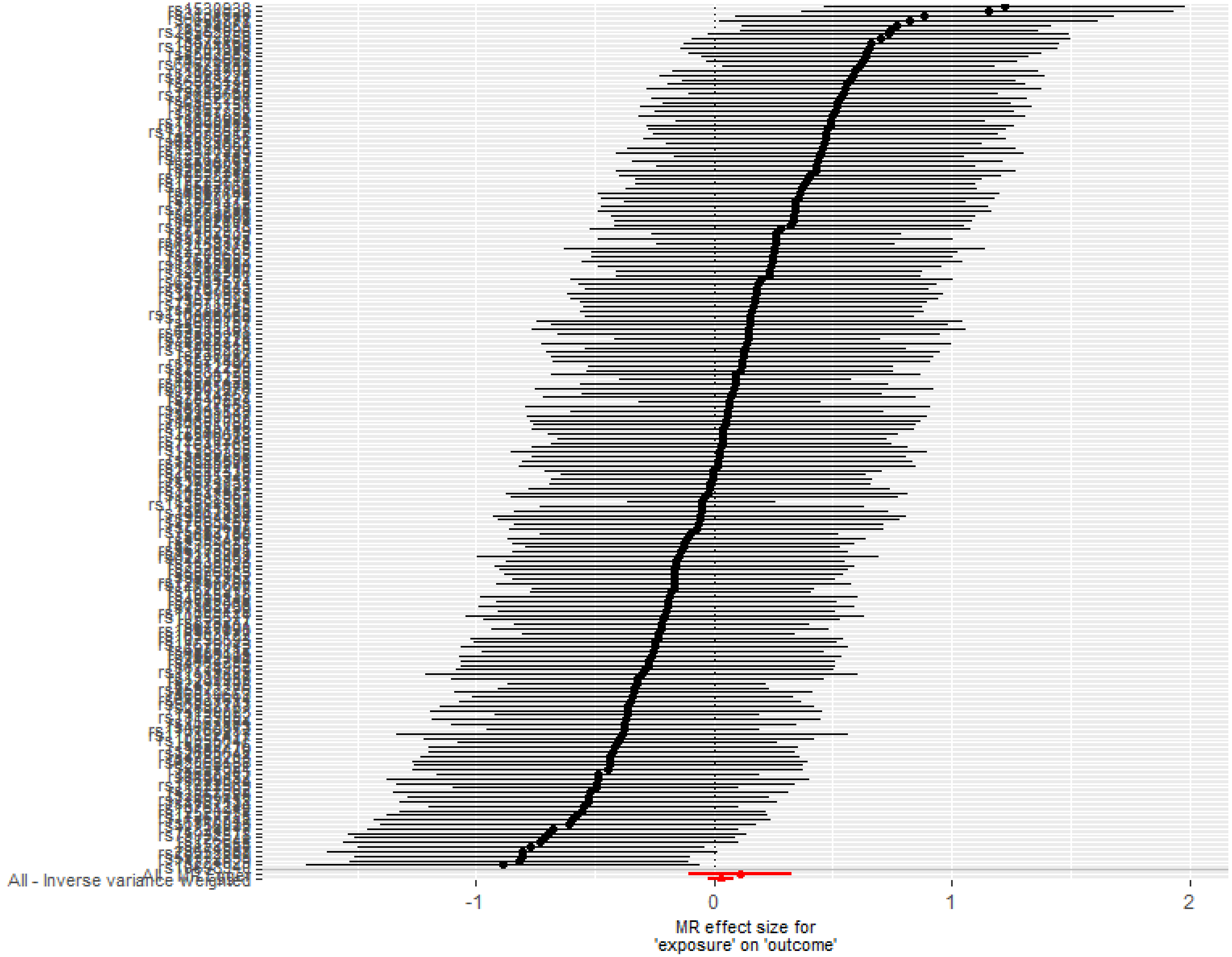

Abbreviations: MR: Mendelian randomization

Supplementary Figure S102. Forest plot of frequent insomnia symptoms [Jansen *et al.* (2019)] and rectal cancer association in males

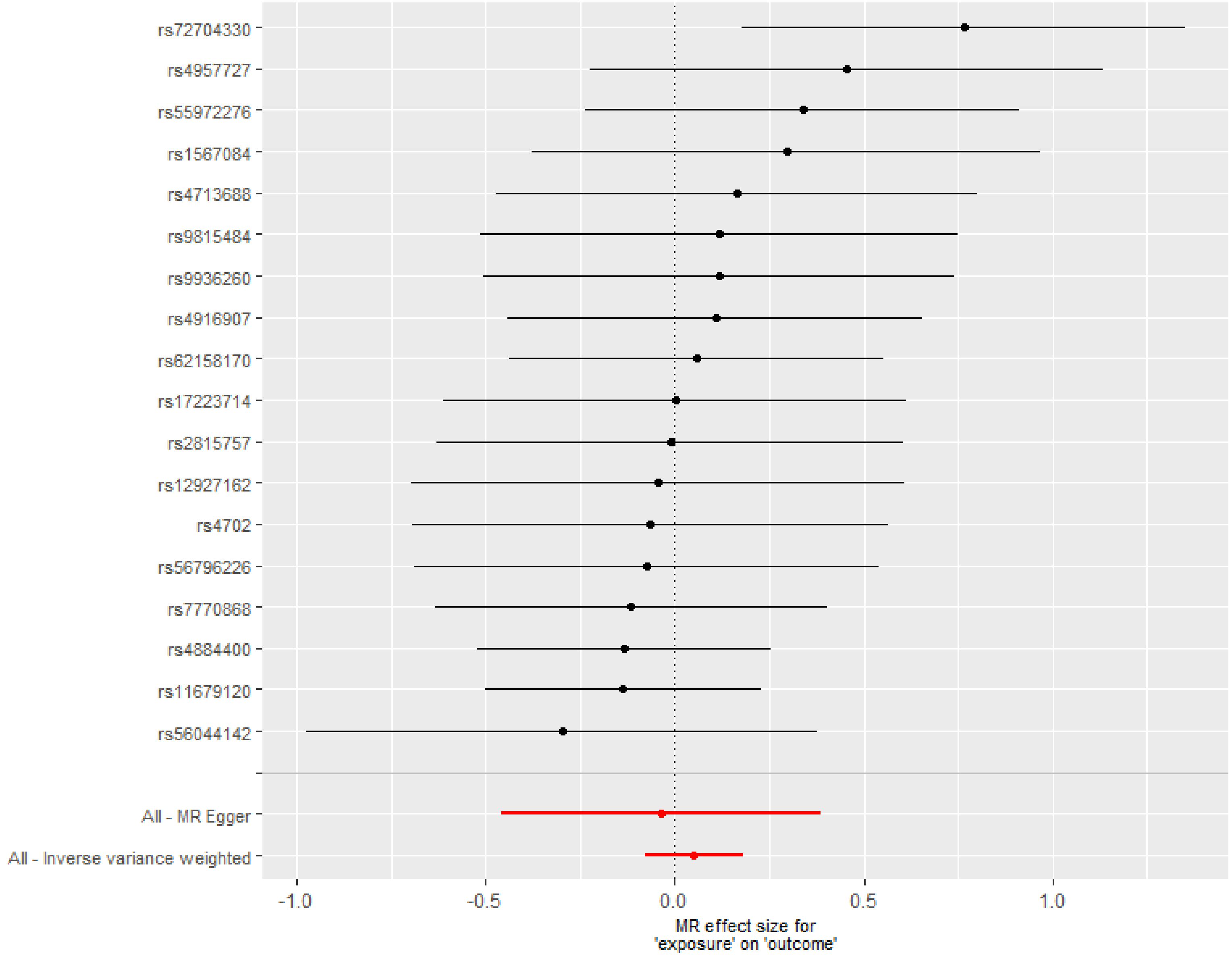

Abbreviations: MR: Mendelian randomization

Supplementary Figure S103. Forest plot of frequent insomnia symptoms [Jansen *et al.* (2019)] and rectal cancer association in females

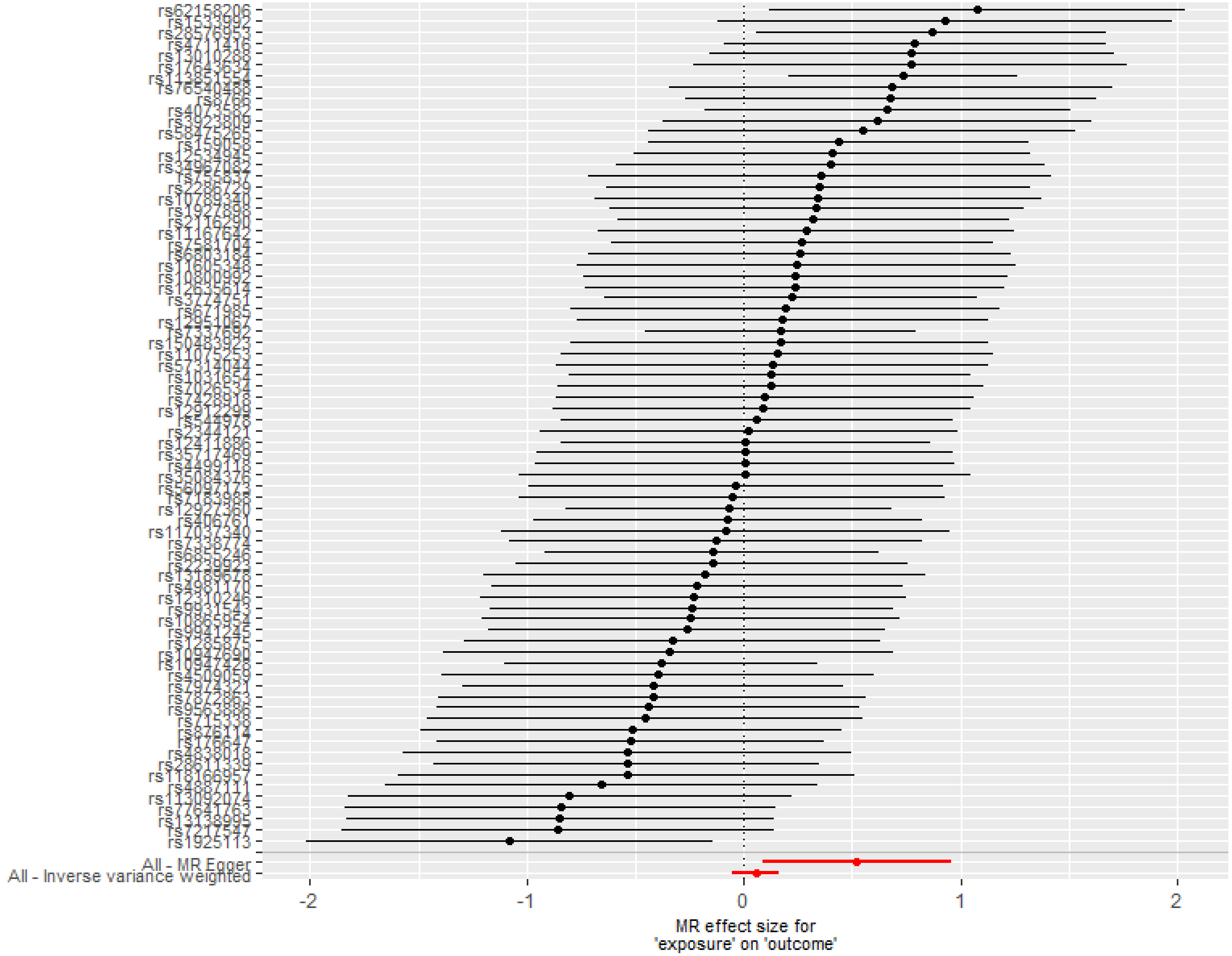

Abbreviations: MR: Mendelian randomization

Supplementary Figure S104. Forest plot of frequent insomnia symptoms [Jansen *et al.* (2019)] and rectal cancer association

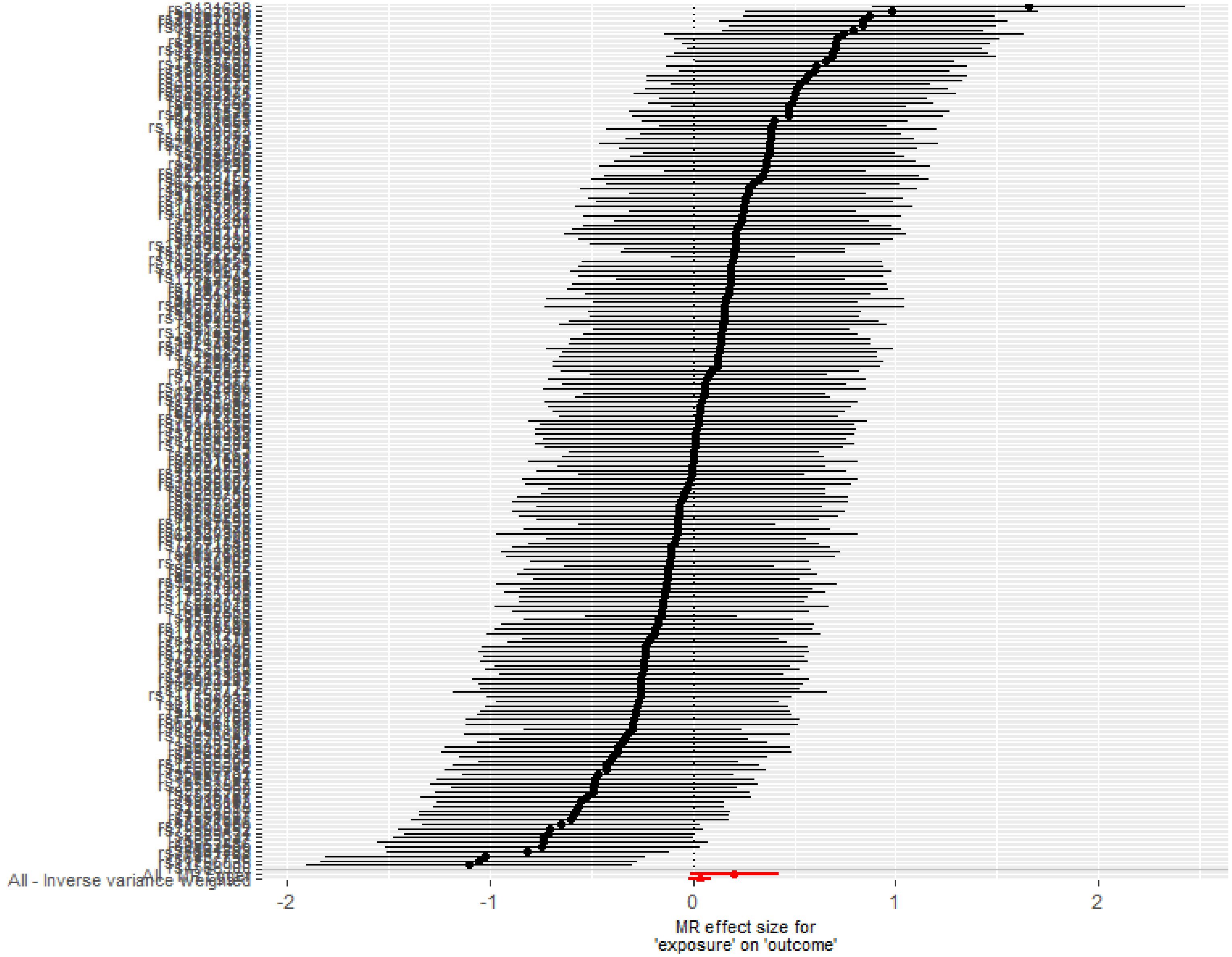

Abbreviations: MR: Mendelian randomization

Supplementary Figure S105. Funnel plot of frequent insomnia symptoms [Jansen *et al.* (2019)] and colorectal cancer association in males

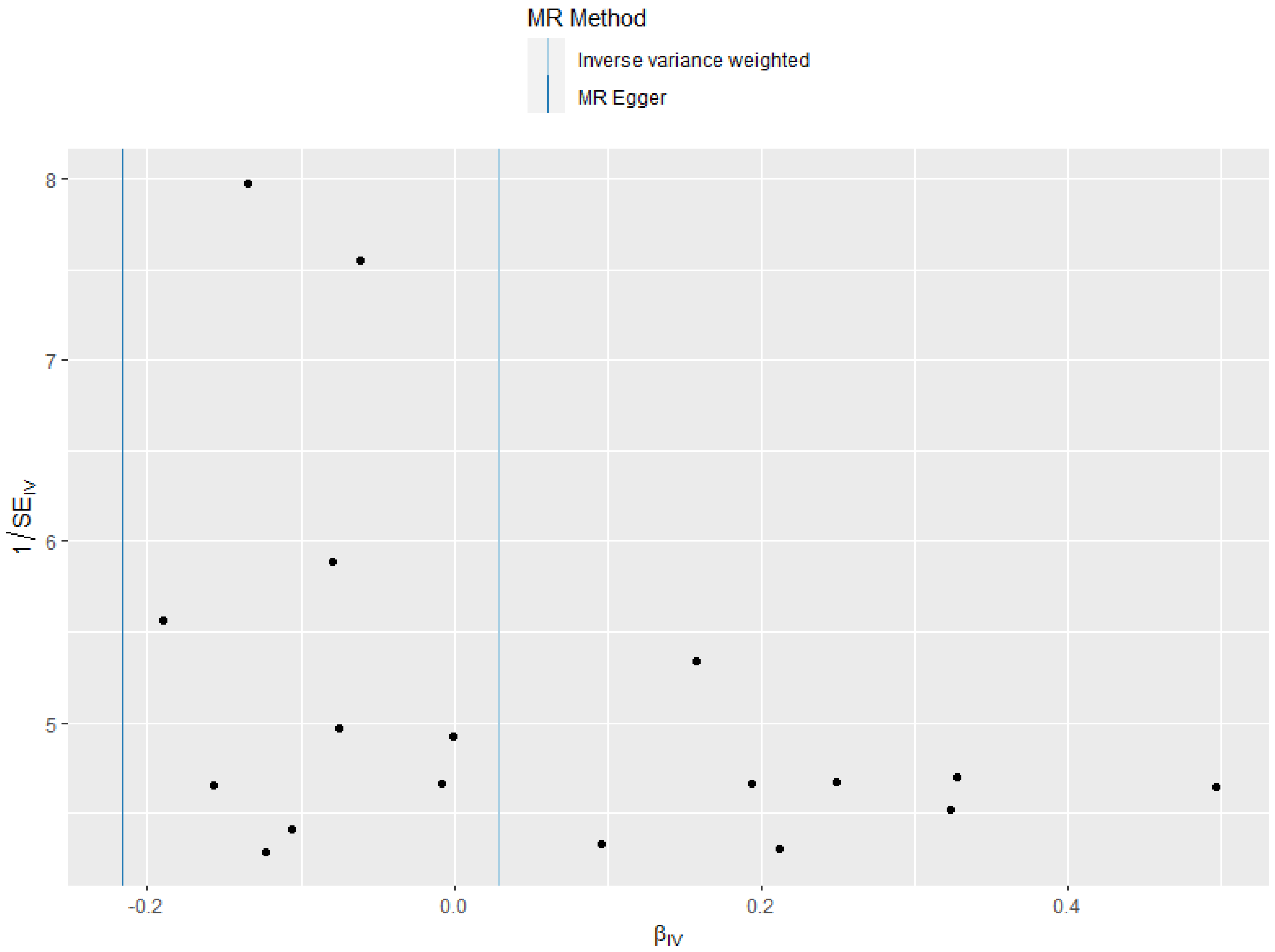

Abbreviations: MR: Mendelian randomization; SE_{IV}: Inverse-variance Standard Error; β_{IV}: Inverse-variance beta coefficient

Supplementary Figure S106. Funnel plot of frequent insomnia symptoms [Jansen *et al.* (2019)] and colorectal cancer association in females

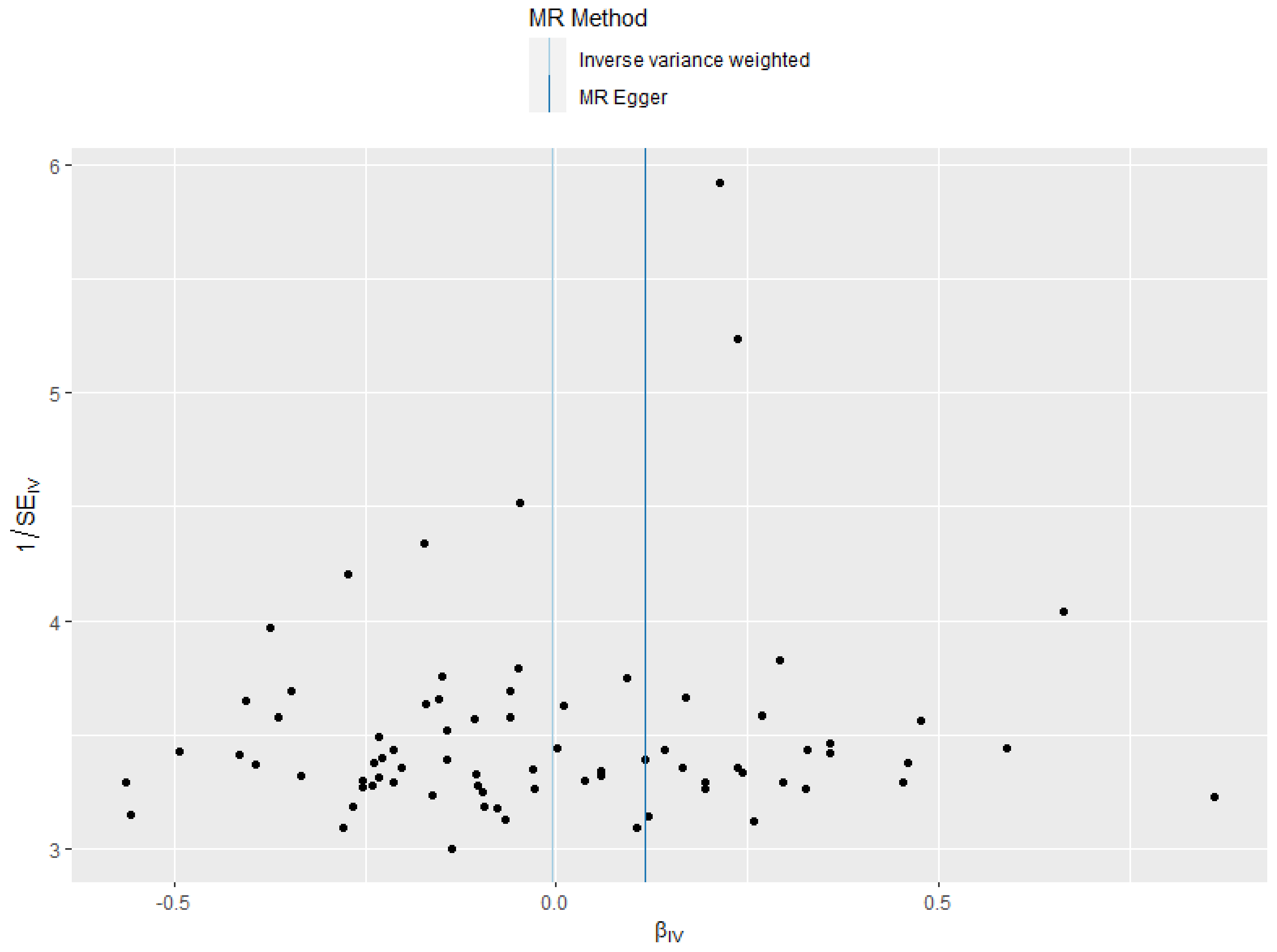

Abbreviations: MR: Mendelian randomization; SE_{IV}: Inverse-variance Standard Error; β_{IV}: Inverse-variance beta coefficient

Supplementary Figure S107. Funnel plot of frequent insomnia symptoms [Jansen *et al.* (2019)] and colorectal cancer association

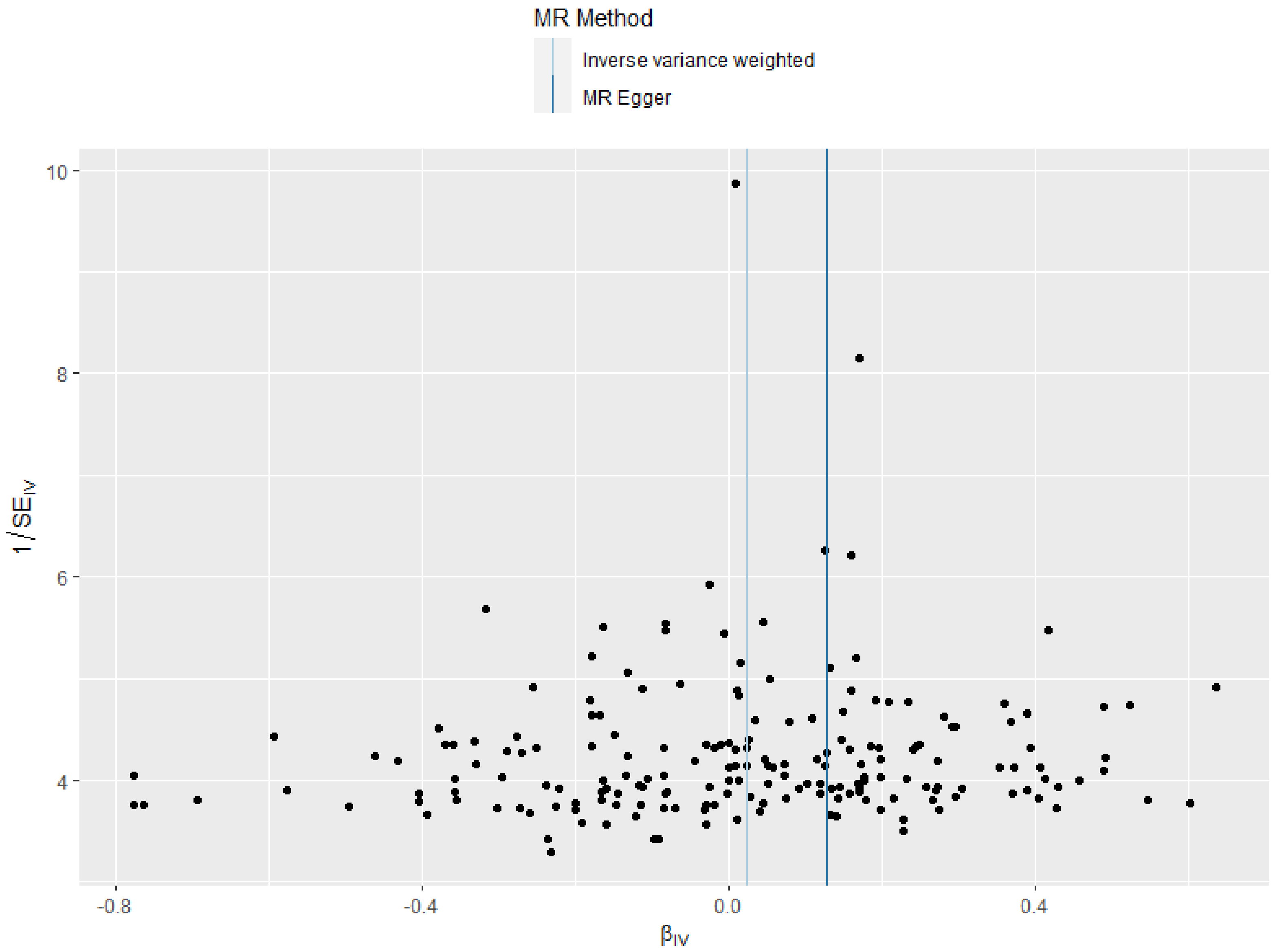

Abbreviations: MR: Mendelian randomization; SE_{IV}: Inverse-variance Standard Error; β_{IV}: Inverse-variance beta coefficient

Supplementary Figure S108. Funnel plot of frequent insomnia symptoms [Jansen *et al.* (2019)] and colon cancer association in males

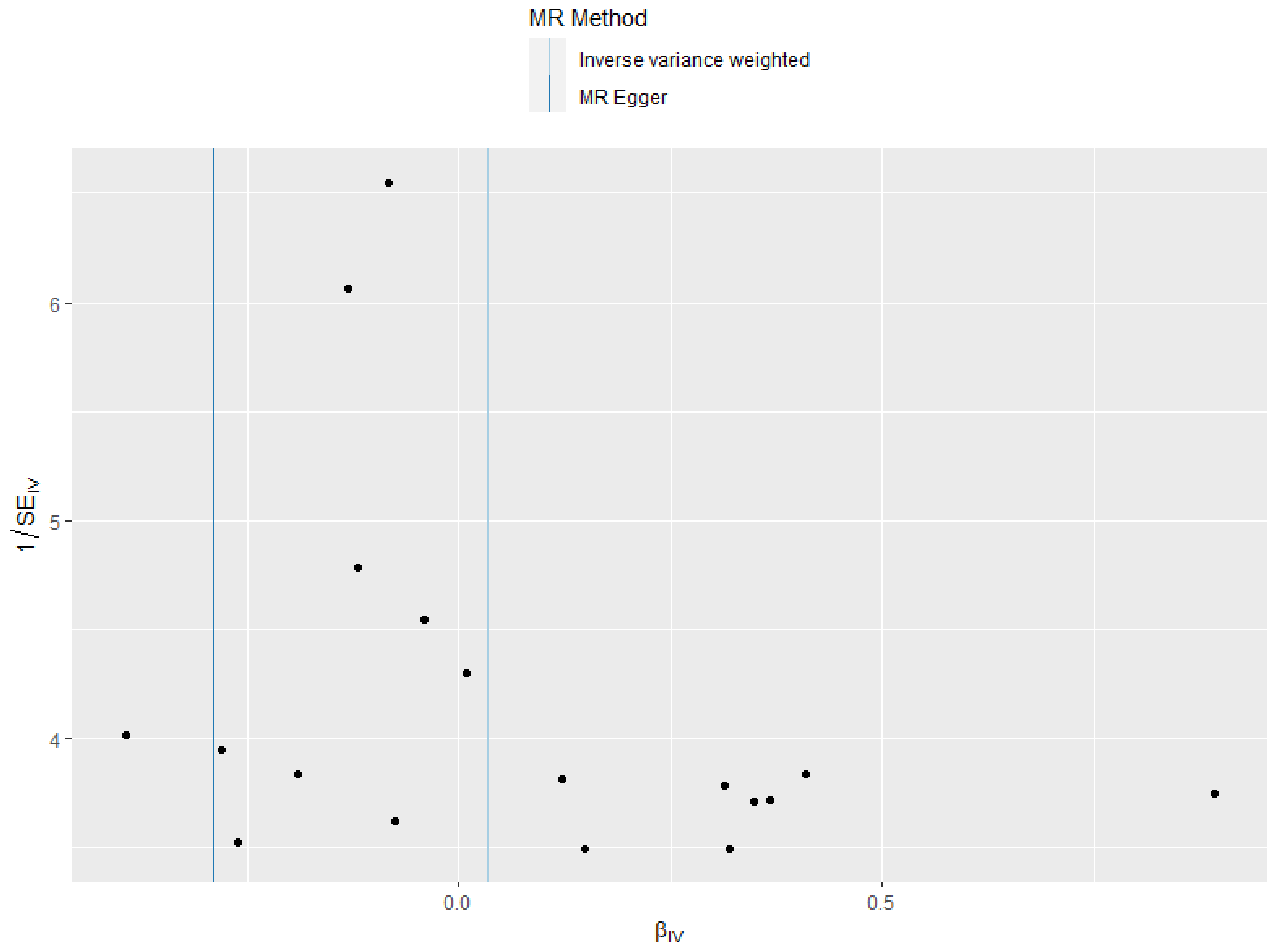

Abbreviations: MR: Mendelian randomization; SE_{IV}: Inverse-variance Standard Error; β_{IV}: Inverse-variance beta coefficient

Supplementary Figure S109. Funnel plot of frequent insomnia symptoms [Jansen *et al.* (2019)] and colon cancer association in females

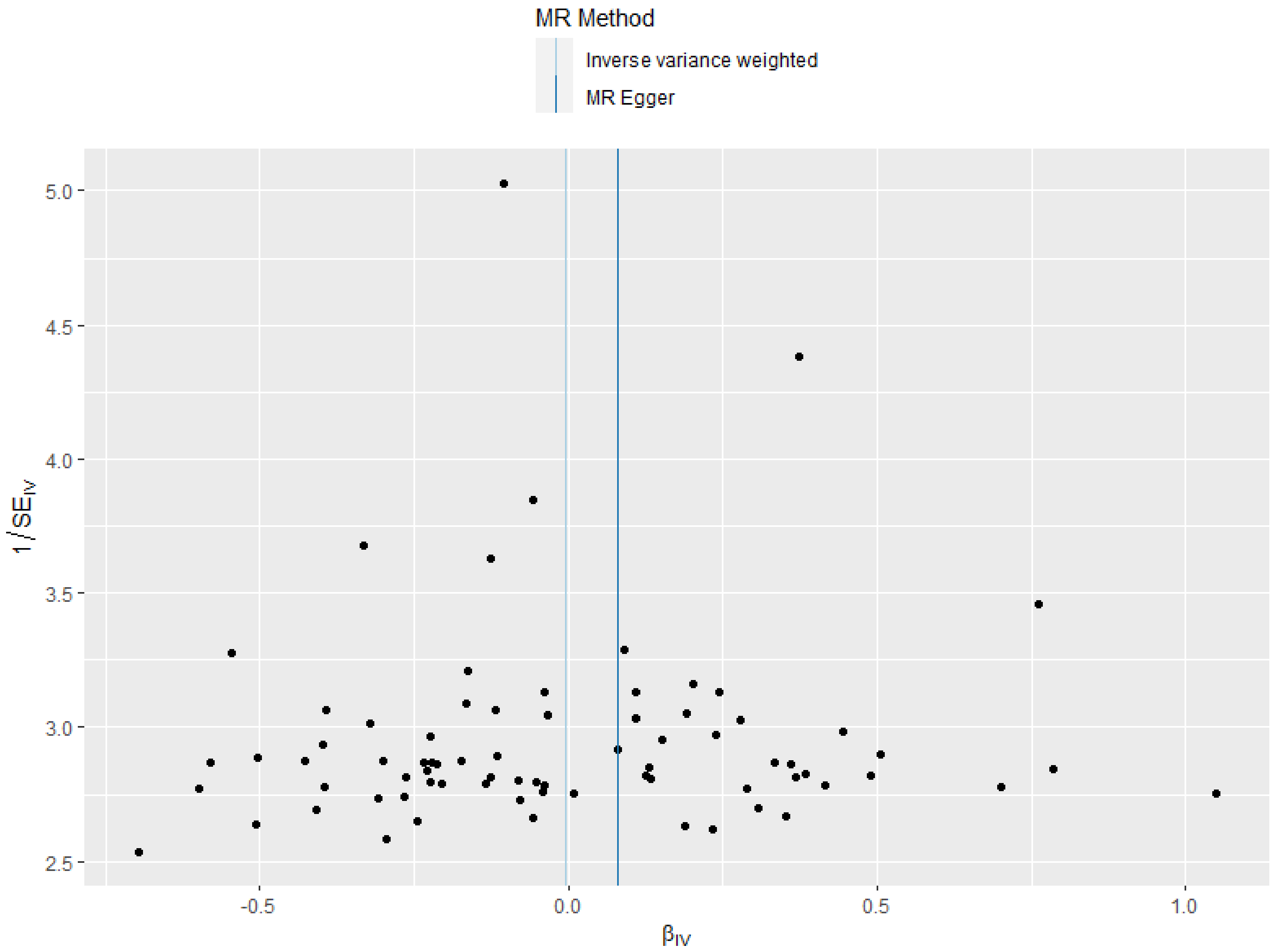

Abbreviations: MR: Mendelian randomization; SE_{IV}: Inverse-variance Standard Error; β_{IV}: Inverse-variance beta coefficient

Supplementary Figure S110. Funnel plot of frequent insomnia symptoms [Jansen *et al.* (2019)] and colon cancer association

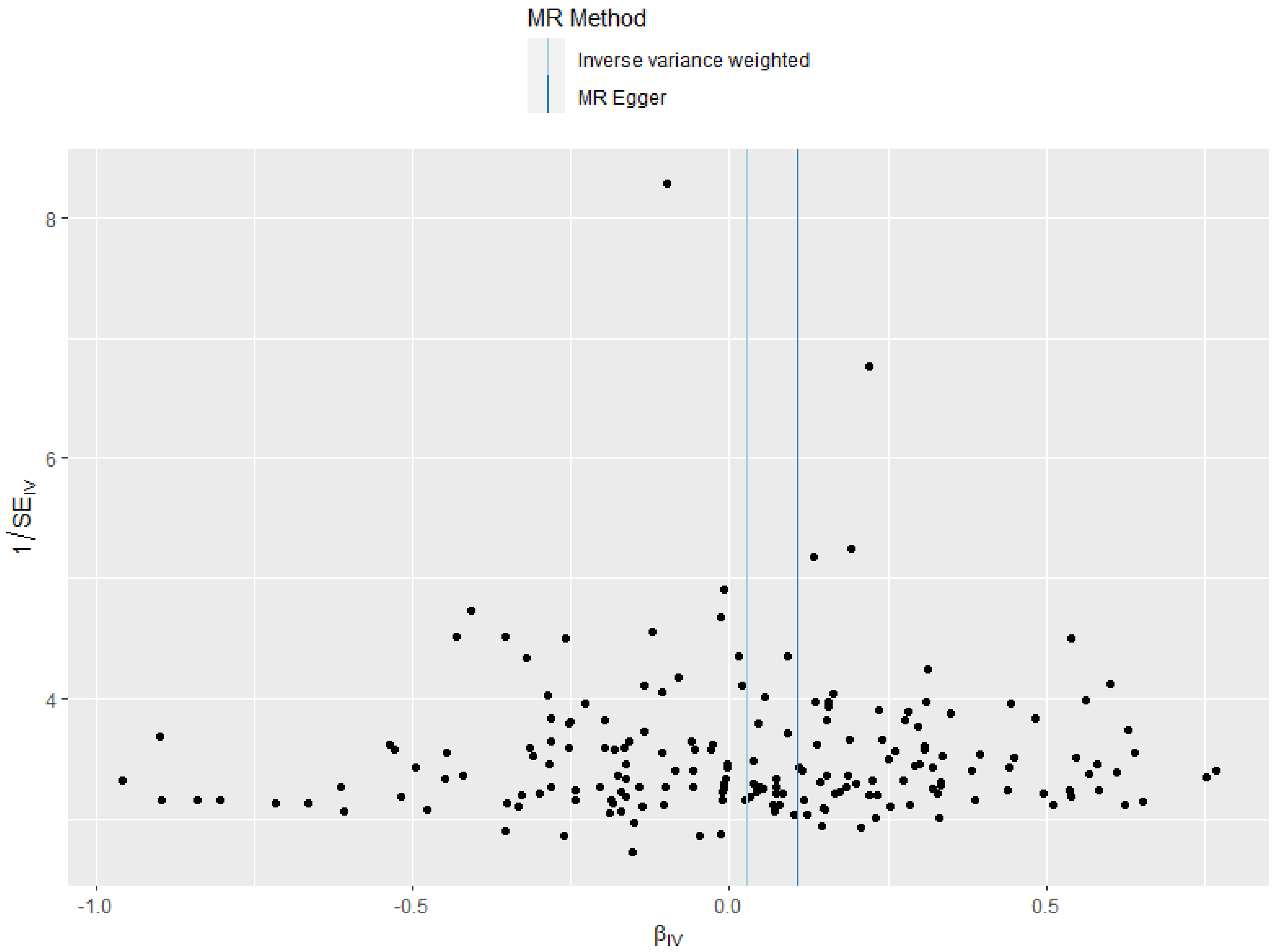

Abbreviations: MR: Mendelian randomization; SE_{IV}: Inverse-variance Standard Error; β_{IV}: Inverse-variance beta coefficient

Supplementary Figure S111. Funnel plot of frequent insomnia symptoms [Jansen *et al.* (2019)] and proximal colon cancer association

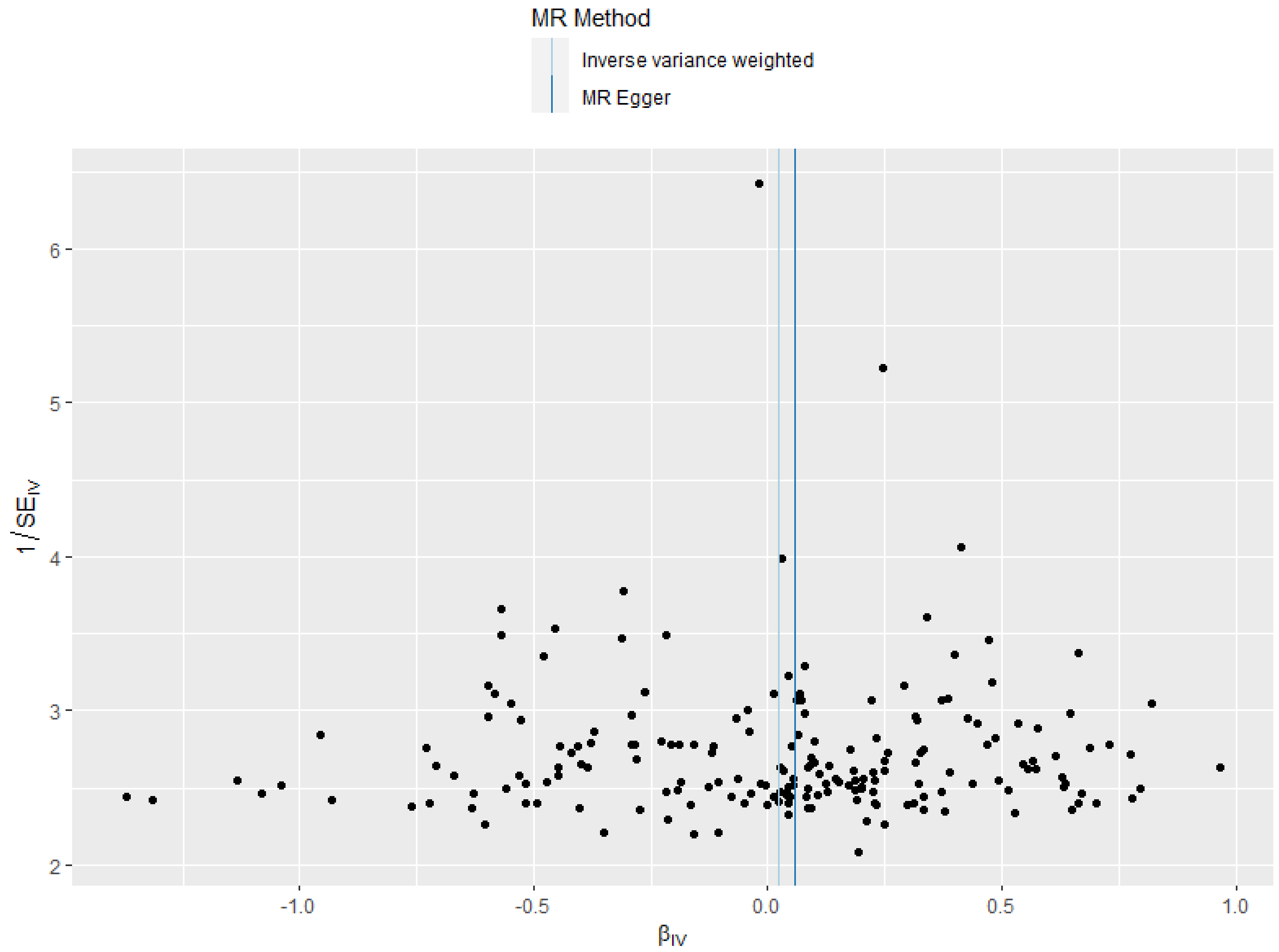

Abbreviations: MR: Mendelian randomization; SE_{IV}: Inverse-variance Standard Error; β_{IV}: Inverse-variance beta coefficient

Supplementary Figure S112. Funnel plot of frequent insomnia symptoms [Jansen *et al.* (2019)] and distal colon cancer association

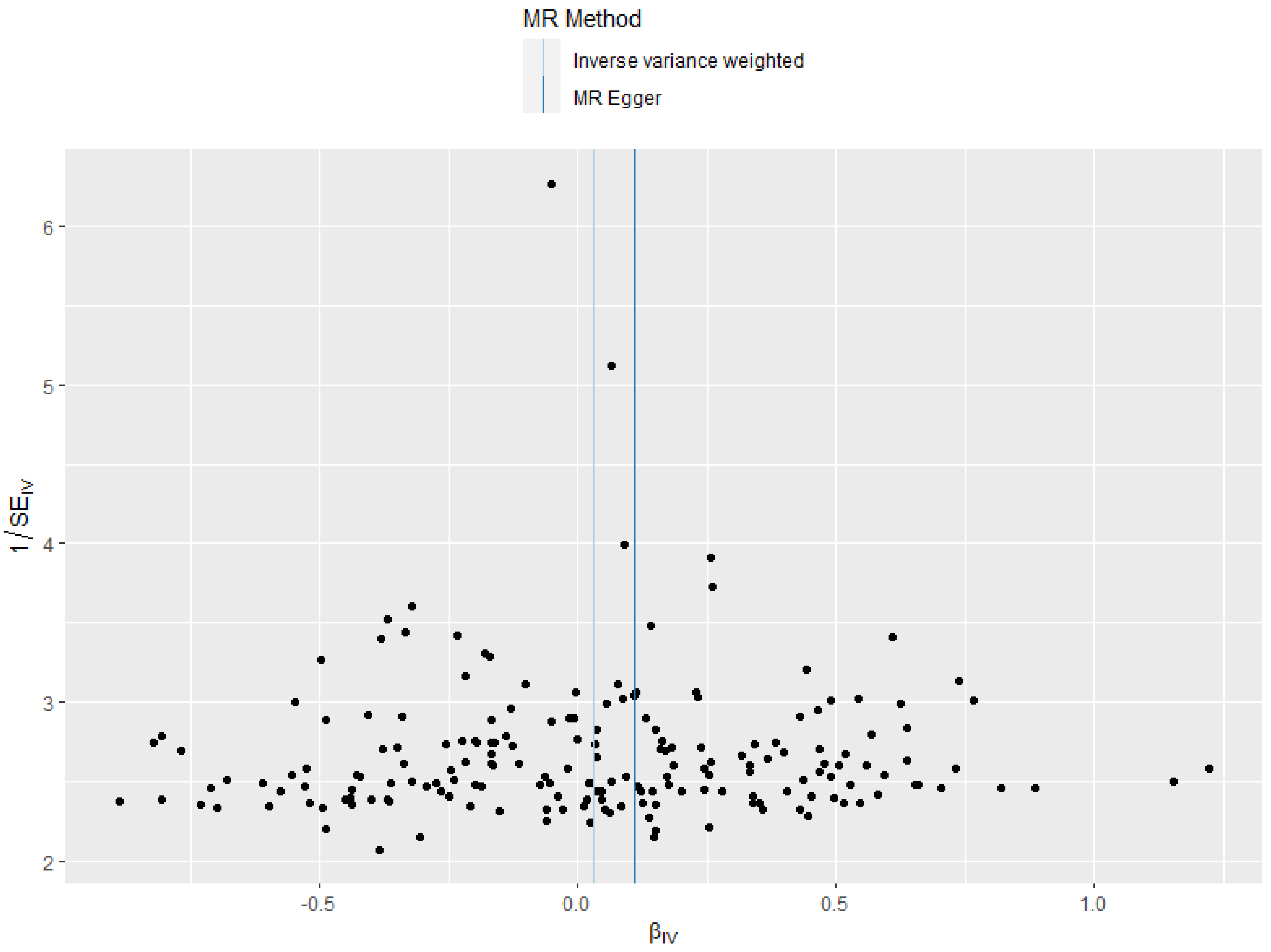

Abbreviations: MR: Mendelian randomization; SE_{IV}: Inverse-variance Standard Error; $β_{IV}$: Inverse-variance beta coefficient

Supplementary Figure S113. Funnel plot of frequent insomnia symptoms [Jansen *et al.* (2019)] and rectal cancer association in males

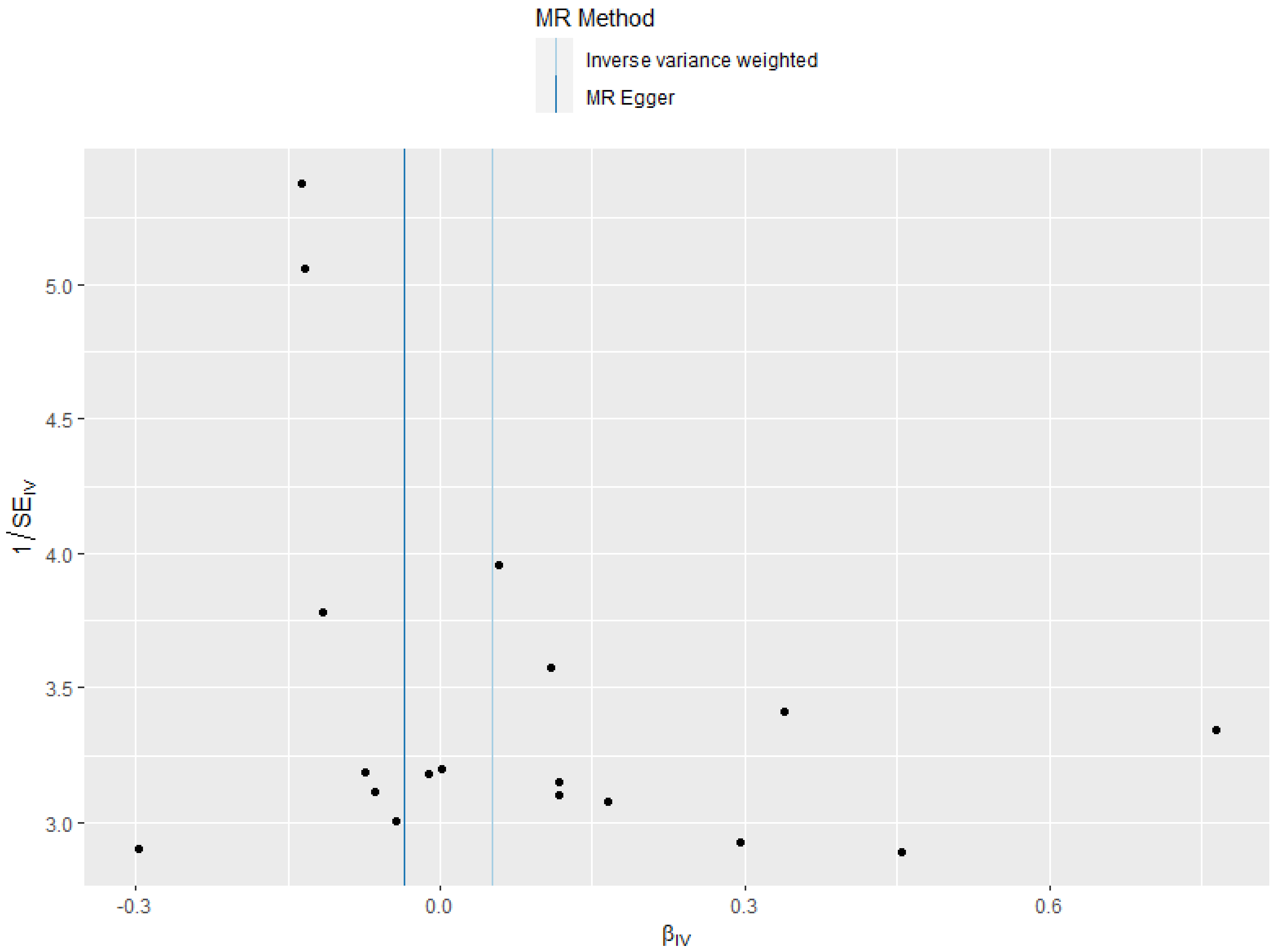

Abbreviations: MR: Mendelian randomization; SE_{IV}: Inverse-variance Standard Error; β_{IV}: Inverse-variance beta coefficient

Supplementary Figure S114. Funnel plot of frequent insomnia symptoms [Jansen *et al.* (2019)] and rectal cancer association in females

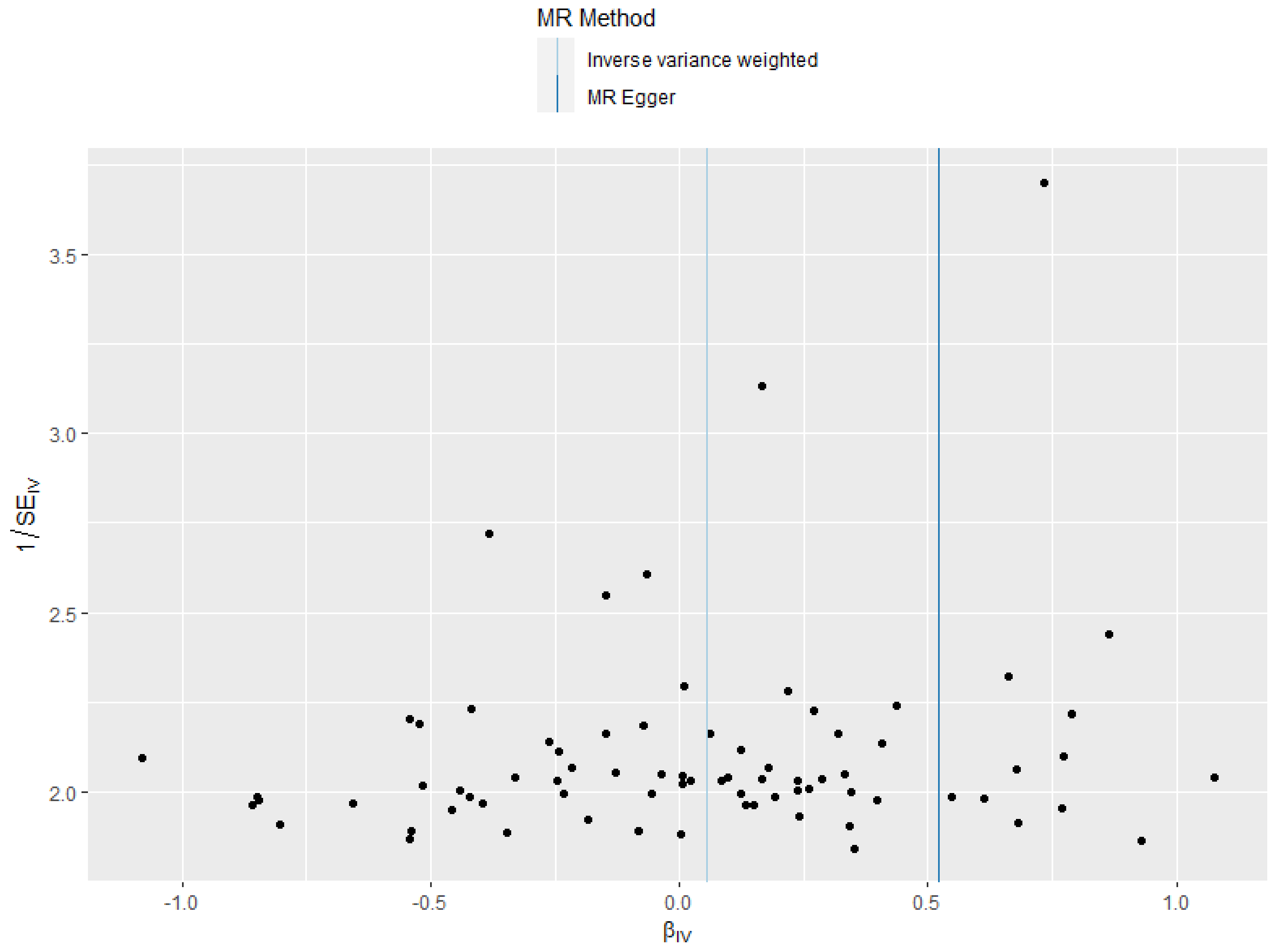

Abbreviations: MR: Mendelian randomization; SE_{IV}: Inverse-variance Standard Error; β_{IV}: Inverse-variance beta coefficient

Supplementary Figure S115. Funnel plot of frequent insomnia symptoms [Jansen *et al.* (2019)] and rectal cancer association

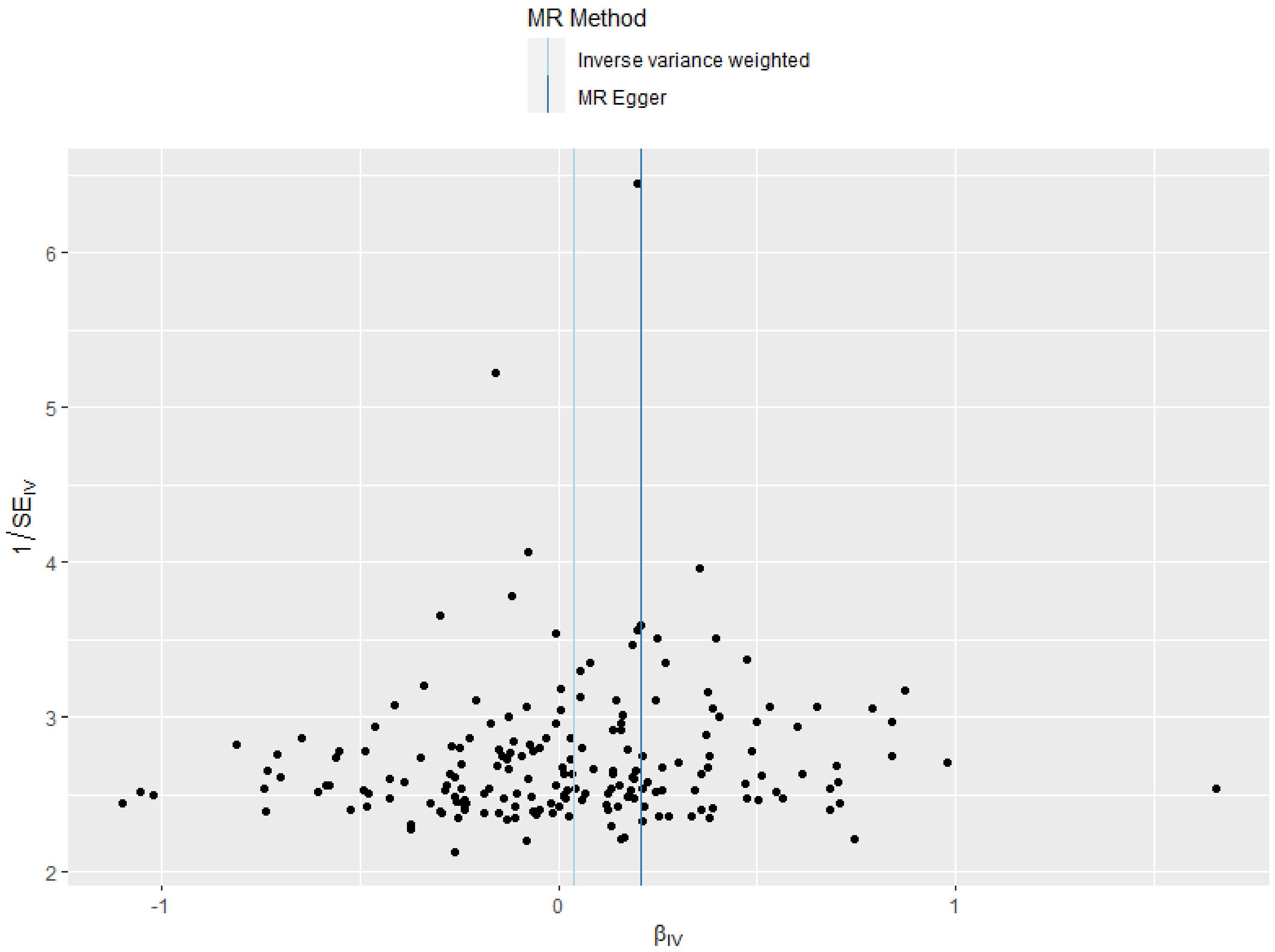

Abbreviations: MR: Mendelian randomization; SE_{IV}: Inverse-variance Standard Error; β_{IV}: Inverse-variance beta coefficient

Primary MR analyses: Sleep duration

Supplementary Figure S116. Scatter plot of sleep duration and colorectal cancer association in males

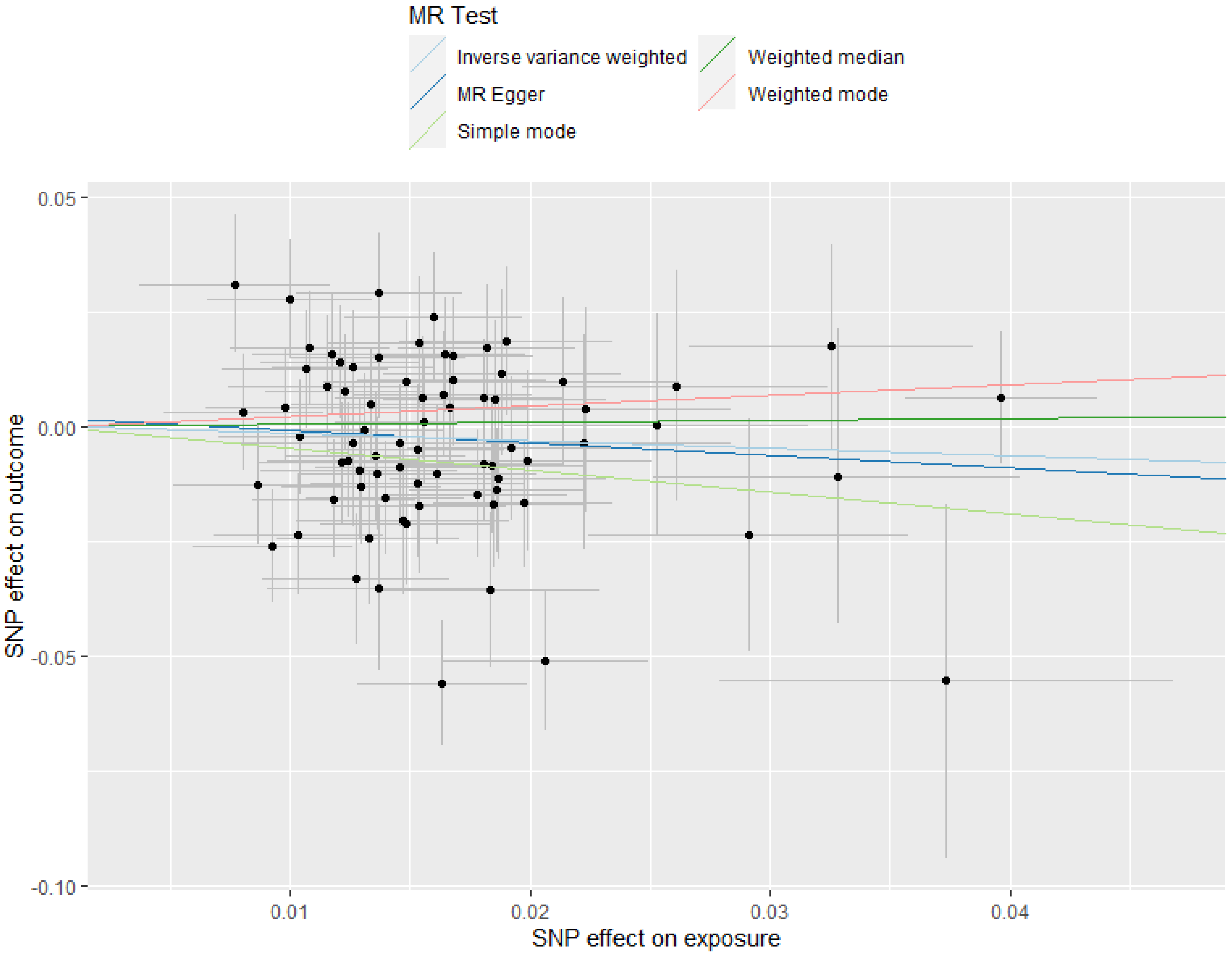

Abbreviations: MR: Mendelian randomization; SNP: Single Nucleotide Polymorphism

Supplementary Figure S117. Scatter plot of sleep duration and colorectal cancer association in females

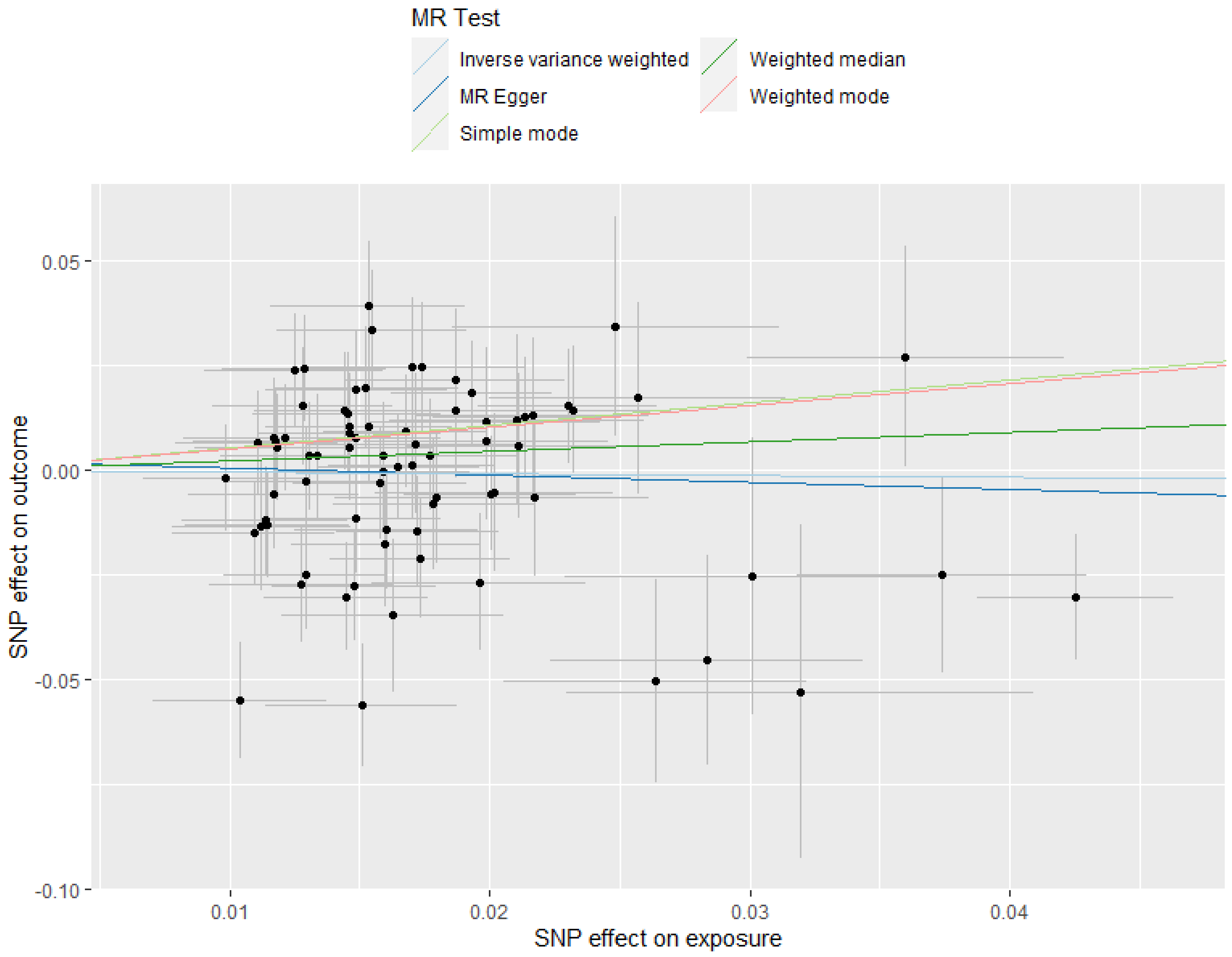

Abbreviations: MR: Mendelian randomization; SNP: Single Nucleotide Polymorphism

Supplementary Figure S118. Scatter plot of sleep duration and colorectal cancer association

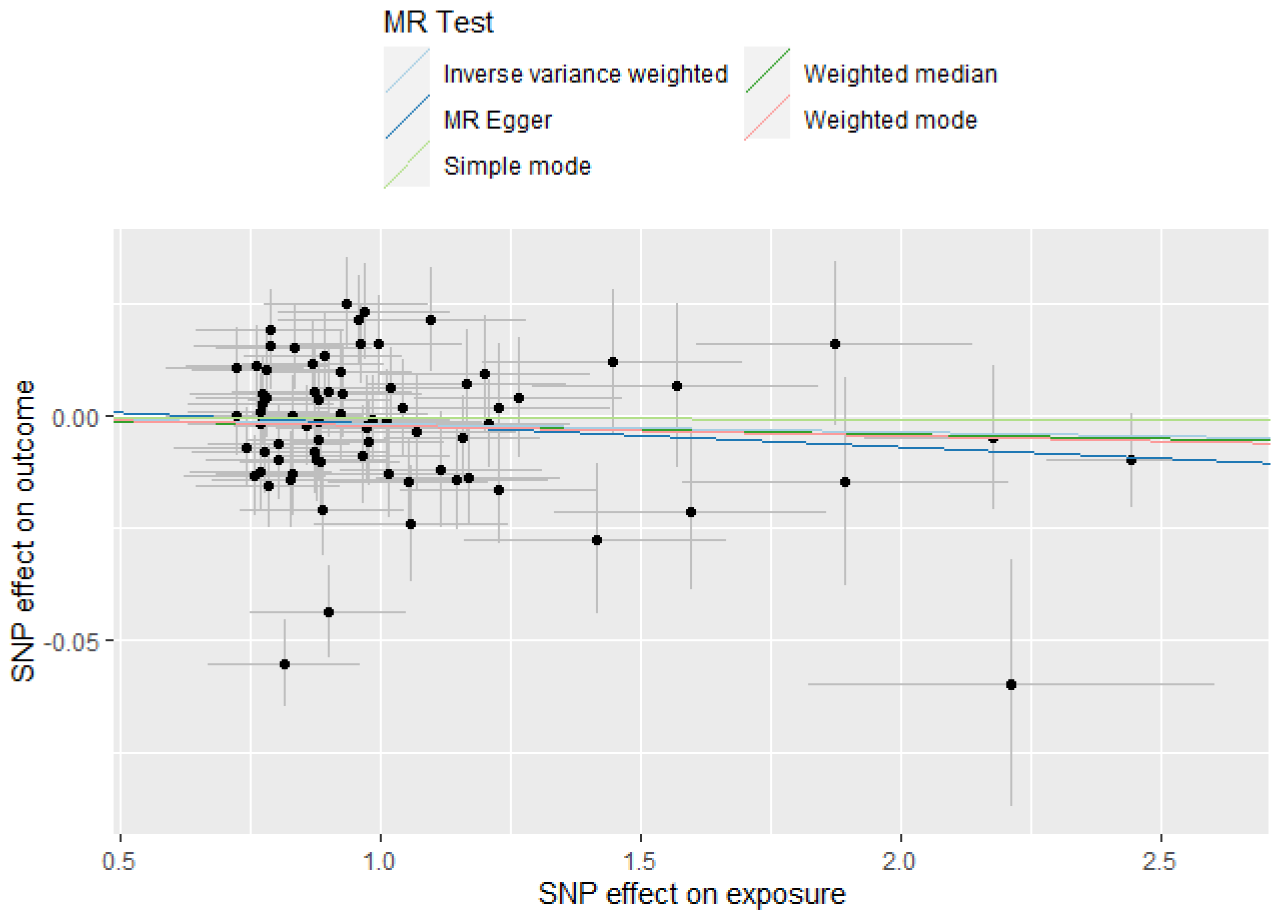

Abbreviations: MR: Mendelian randomization; SNP: Single Nucleotide Polymorphism

Supplementary Figure S119. Scatter plot of sleep duration and colon cancer association in males

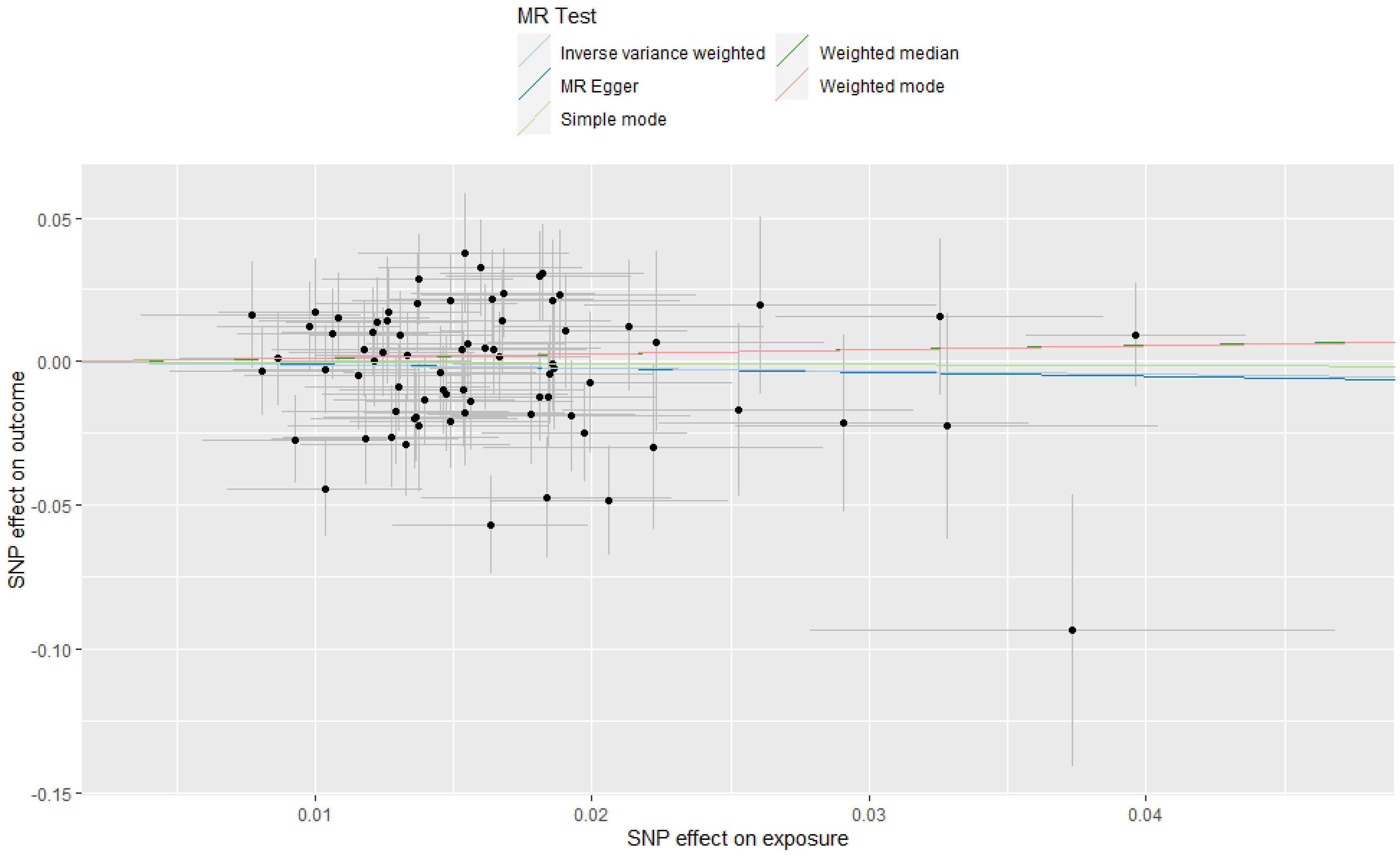

Abbreviations: MR: Mendelian randomization; SNP: Single Nucleotide Polymorphism

Supplementary Figure S120. Scatter plot of sleep duration and colon cancer association in females

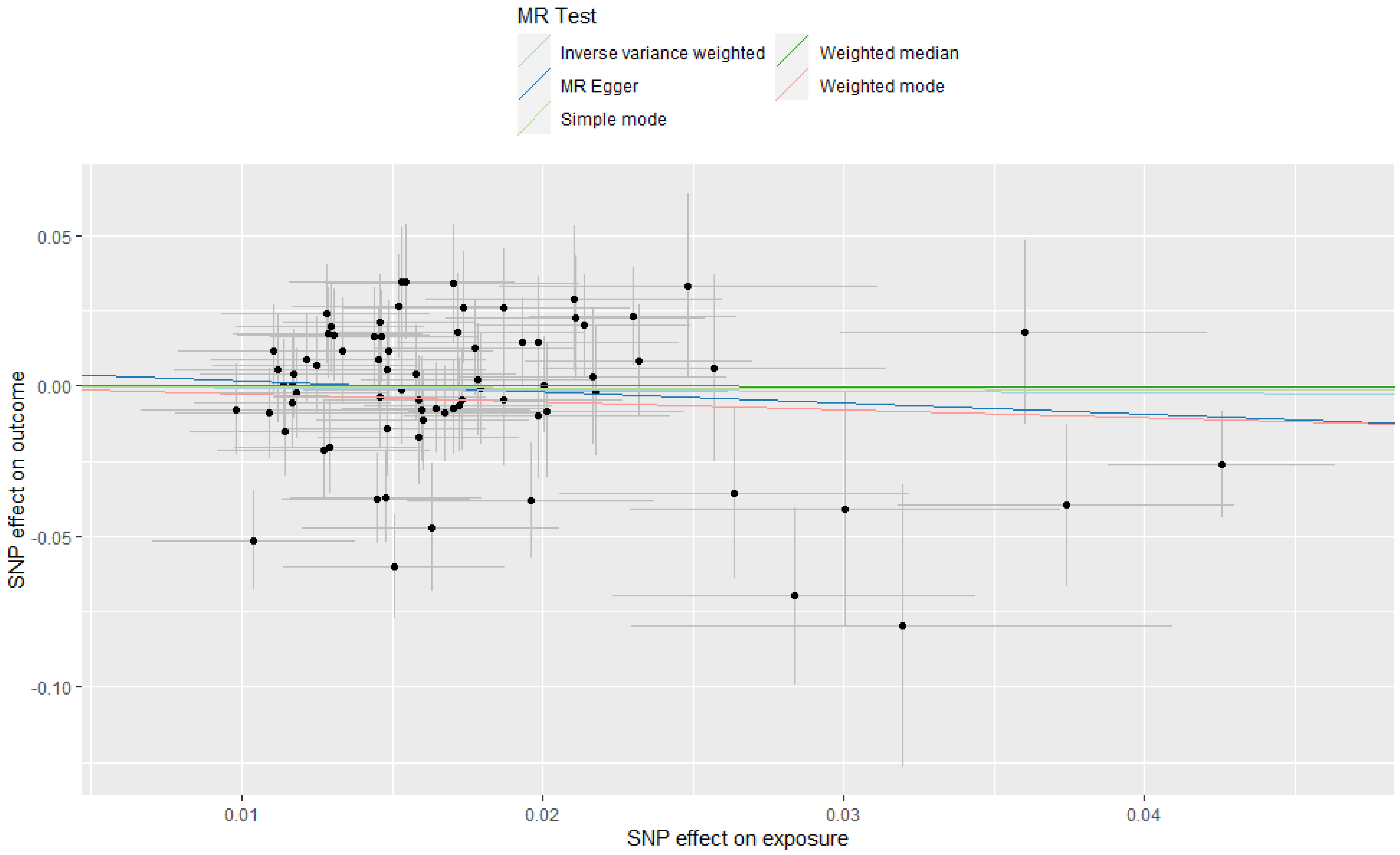

Abbreviations: MR: Mendelian randomization; SNP: Single Nucleotide Polymorphism

Supplementary Figure S121. Scatter plot of sleep duration and colon cancer association

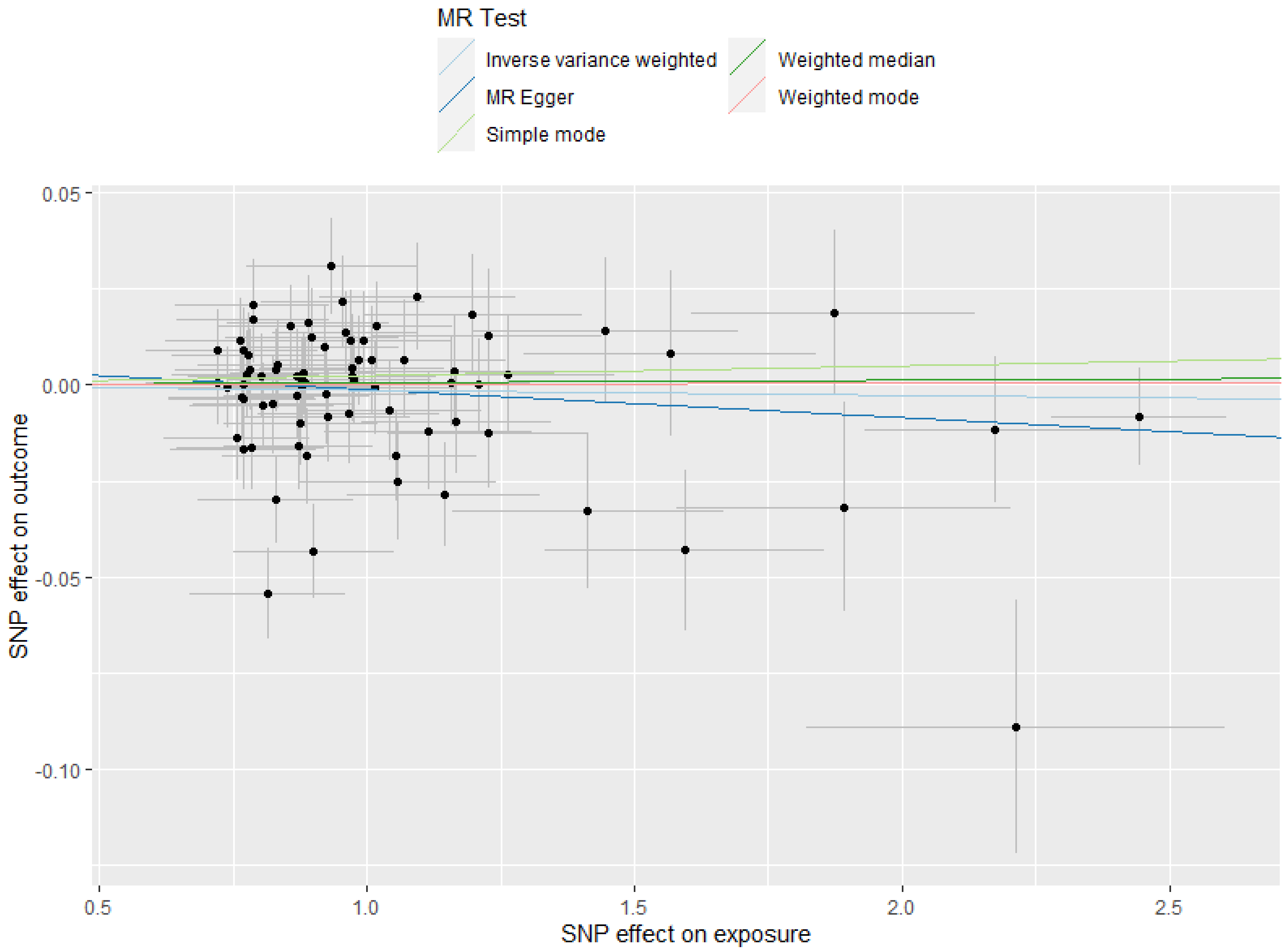

Abbreviations: MR: Mendelian randomization; SNP: Single Nucleotide Polymorphism

Supplementary Figure S122. Scatter plot of sleep duration and proximal colon cancer association

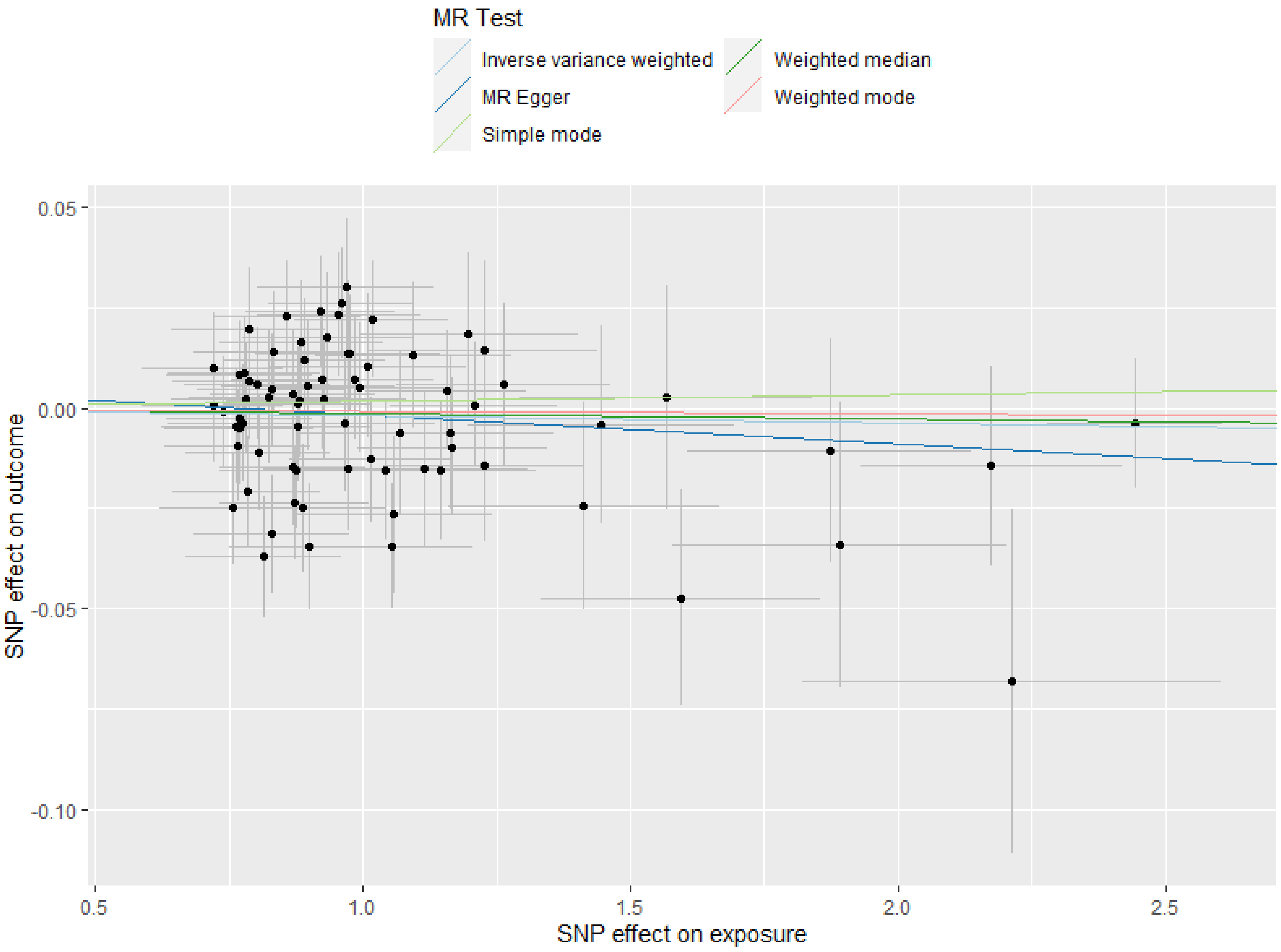

Abbreviations: MR: Mendelian randomization; SNP: Single Nucleotide Polymorphism

Supplementary Figure S123. Scatter plot of sleep duration and distal colon cancer association

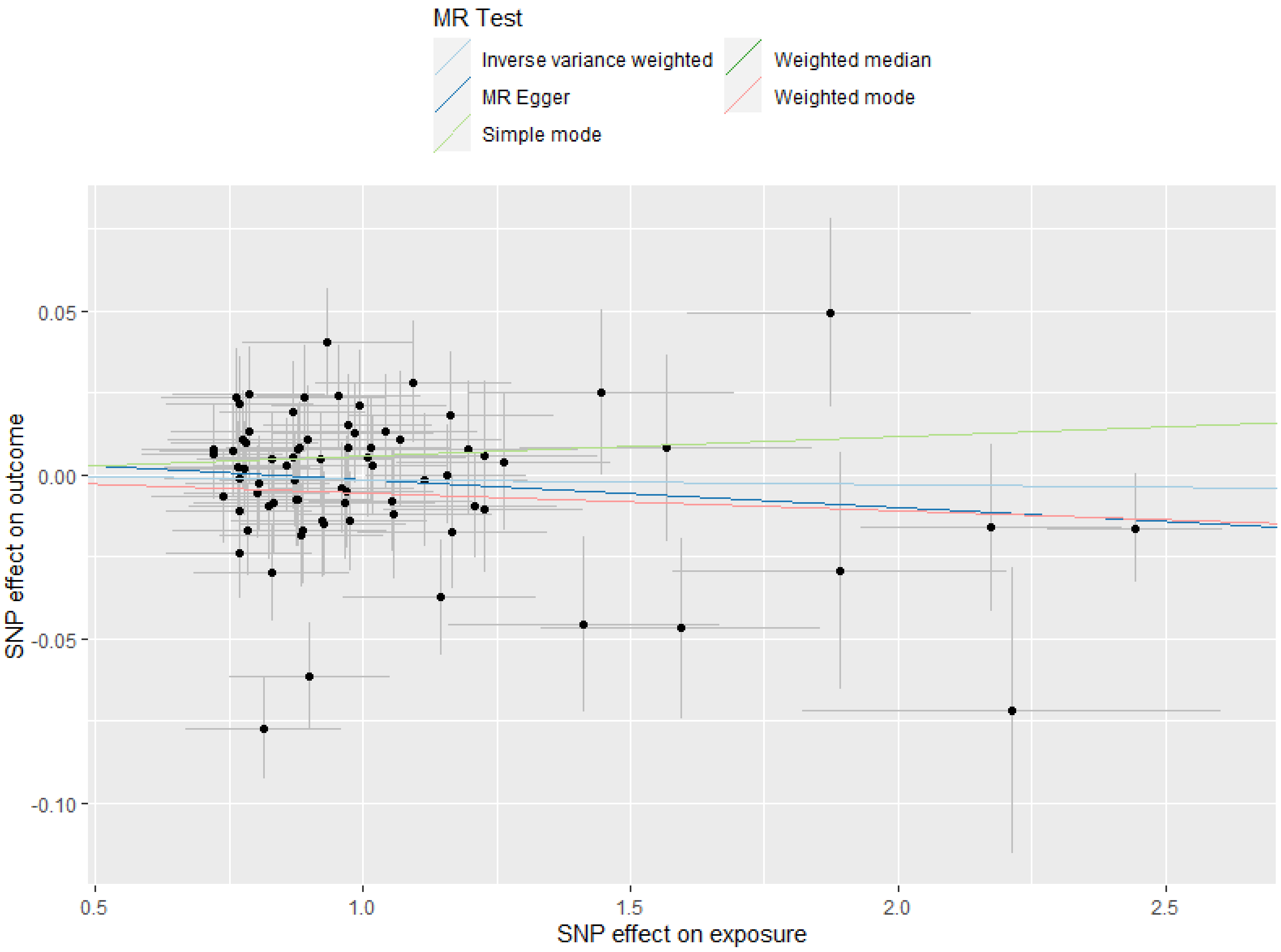

Abbreviations: MR: Mendelian randomization; SNP: Single Nucleotide Polymorphism

Supplementary Figure S124. Scatter plot of sleep duration and rectal cancer association in males

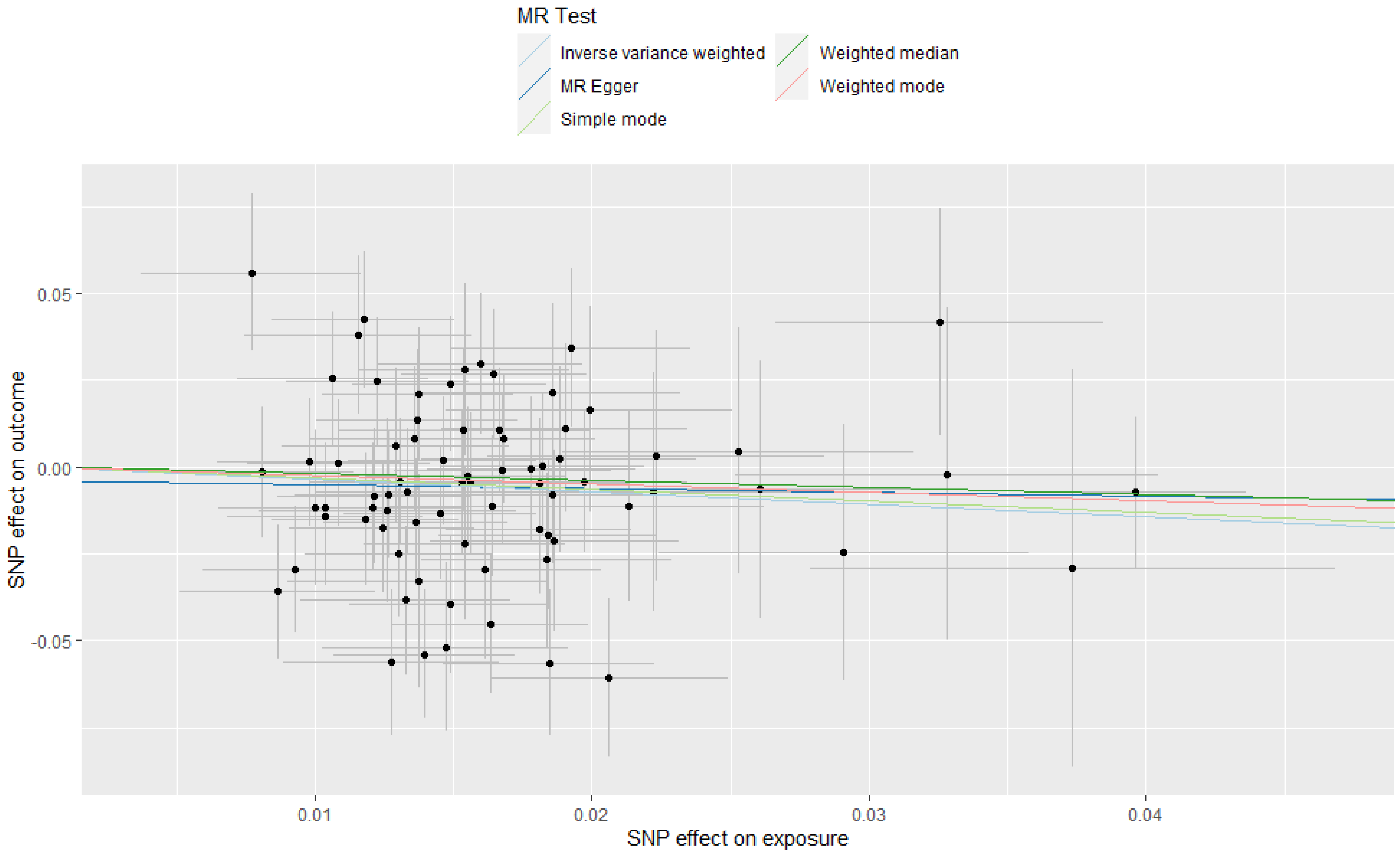

Abbreviations: MR: Mendelian randomization; SNP: Single Nucleotide Polymorphism

Supplementary Figure S125. Scatter plot of sleep duration and rectal cancer association in females

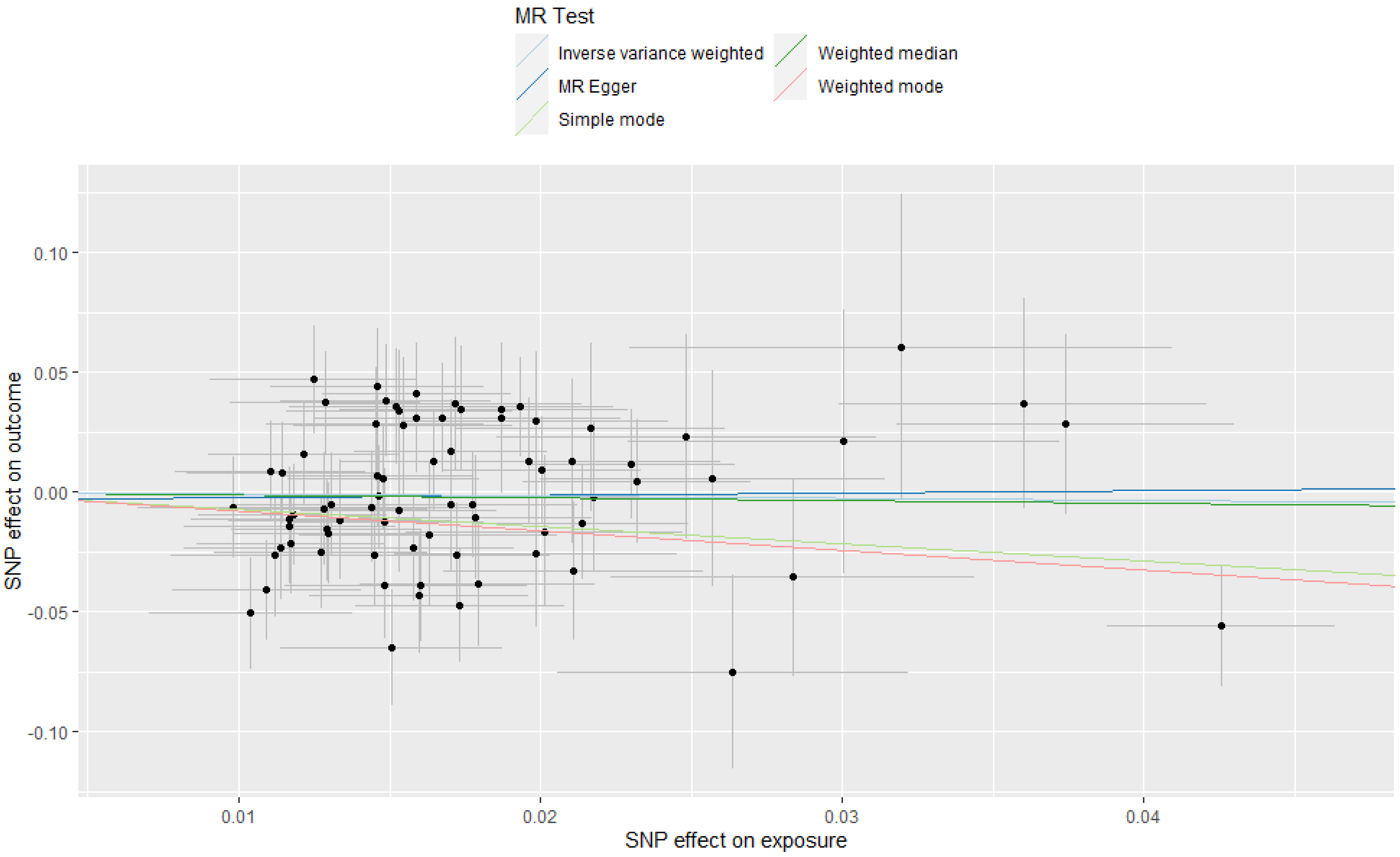

Abbreviations: MR: Mendelian randomization; SNP: Single Nucleotide Polymorphism

Supplementary Figure S126. Scatter plot of sleep duration and rectal cancer association

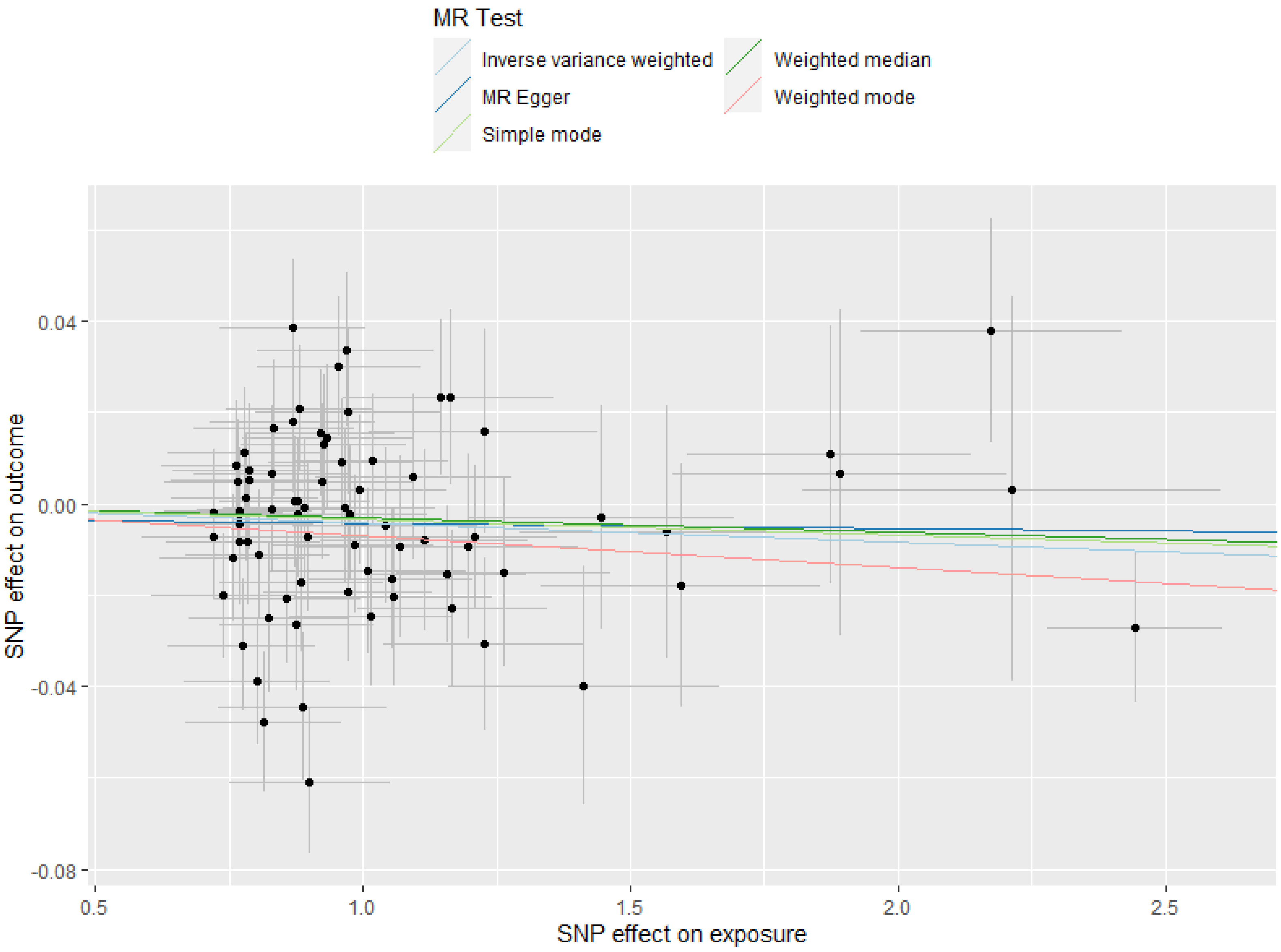

Abbreviations: MR: Mendelian randomization; SNP: Single Nucleotide Polymorphism

Supplementary Figure S127. Forest plot of sleep duration and colorectal cancer association in males

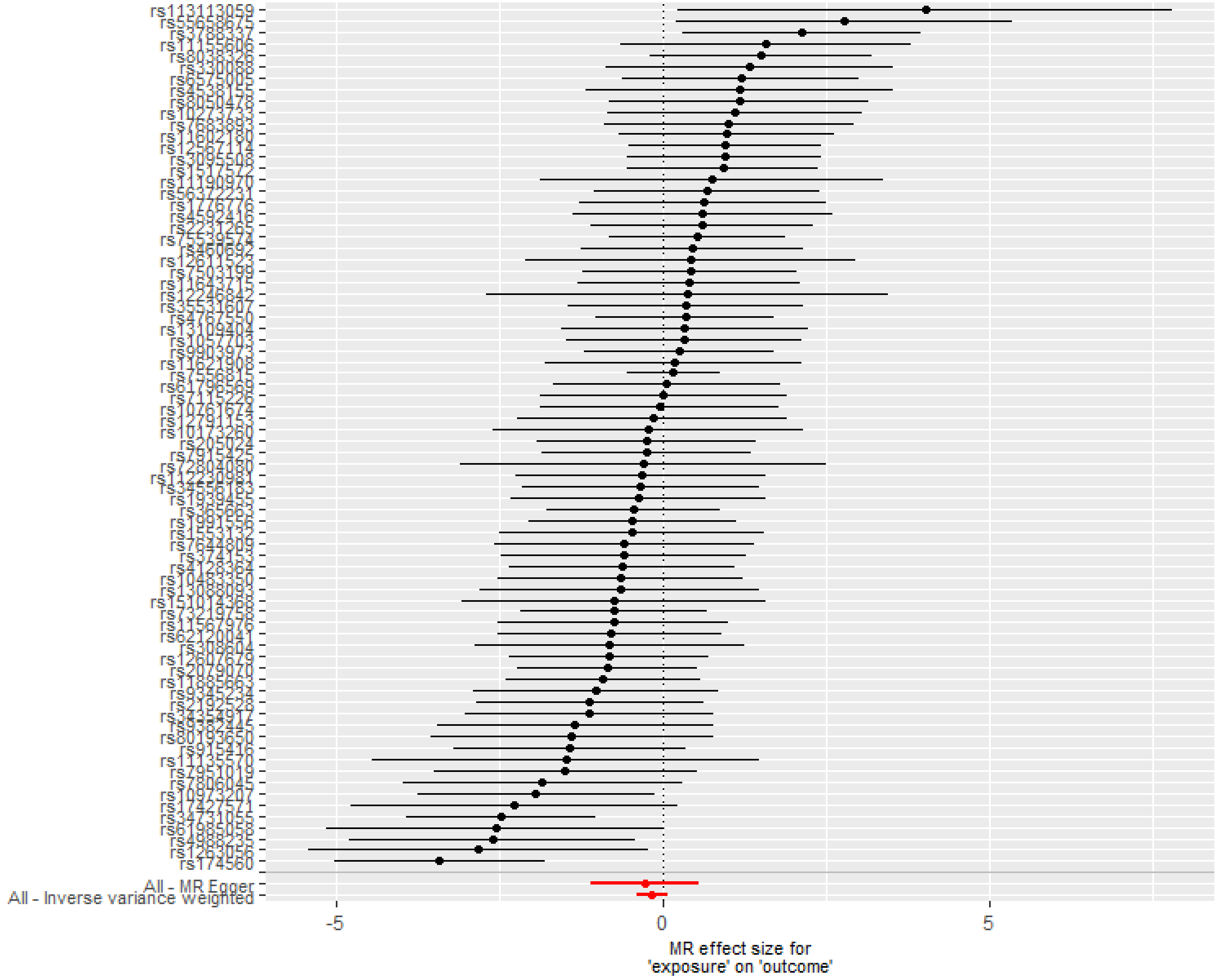

Abbreviations: MR: Mendelian randomization

Supplementary Figure S128. Forest plot of sleep duration and colorectal cancer association in females

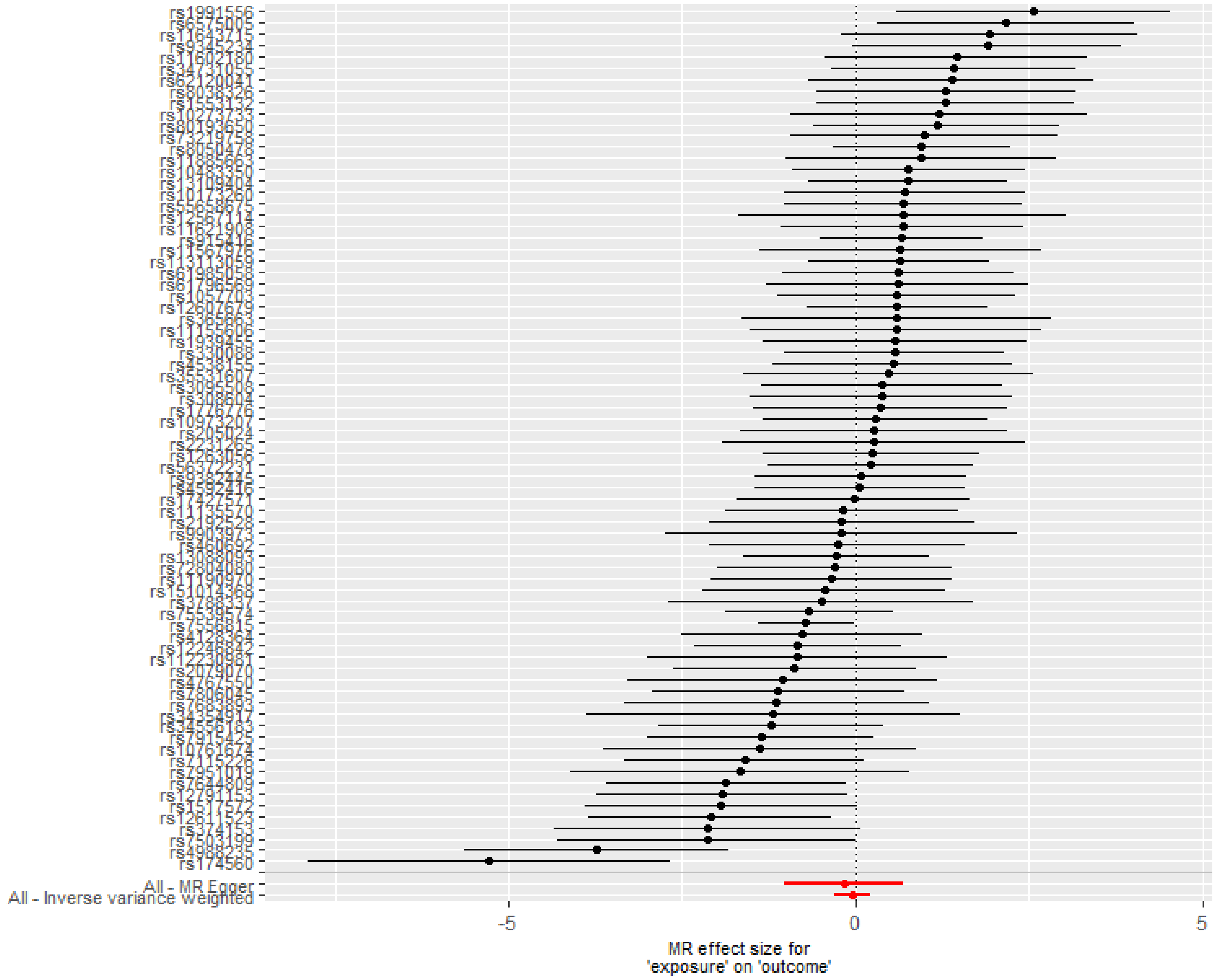

Abbreviations: MR: Mendelian randomization

Supplementary Figure S129. Forest plot of sleep duration and colorectal cancer association

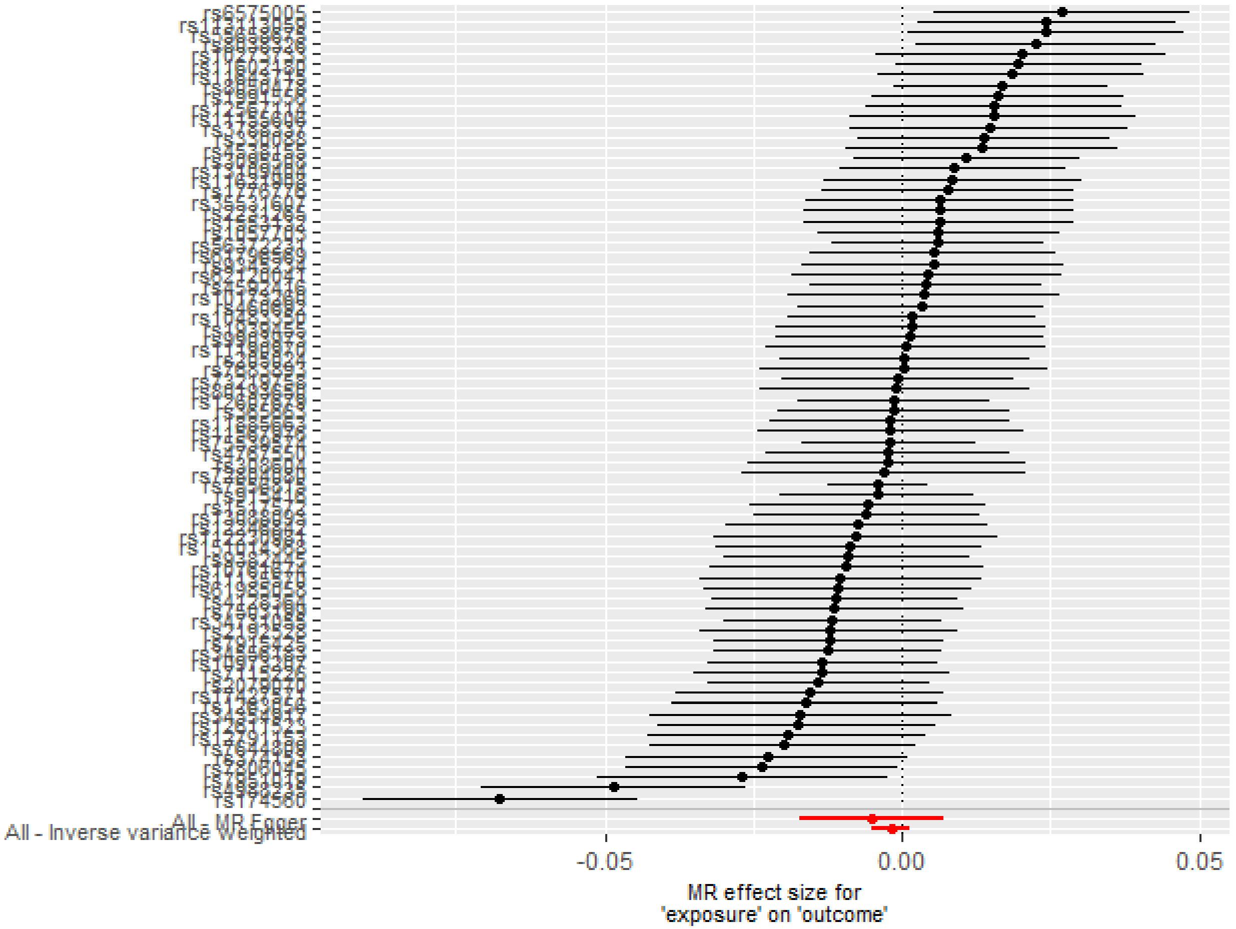

Abbreviations: MR: Mendelian randomization

Supplementary Figure S130. Forest plot of sleep duration and colon cancer association in males

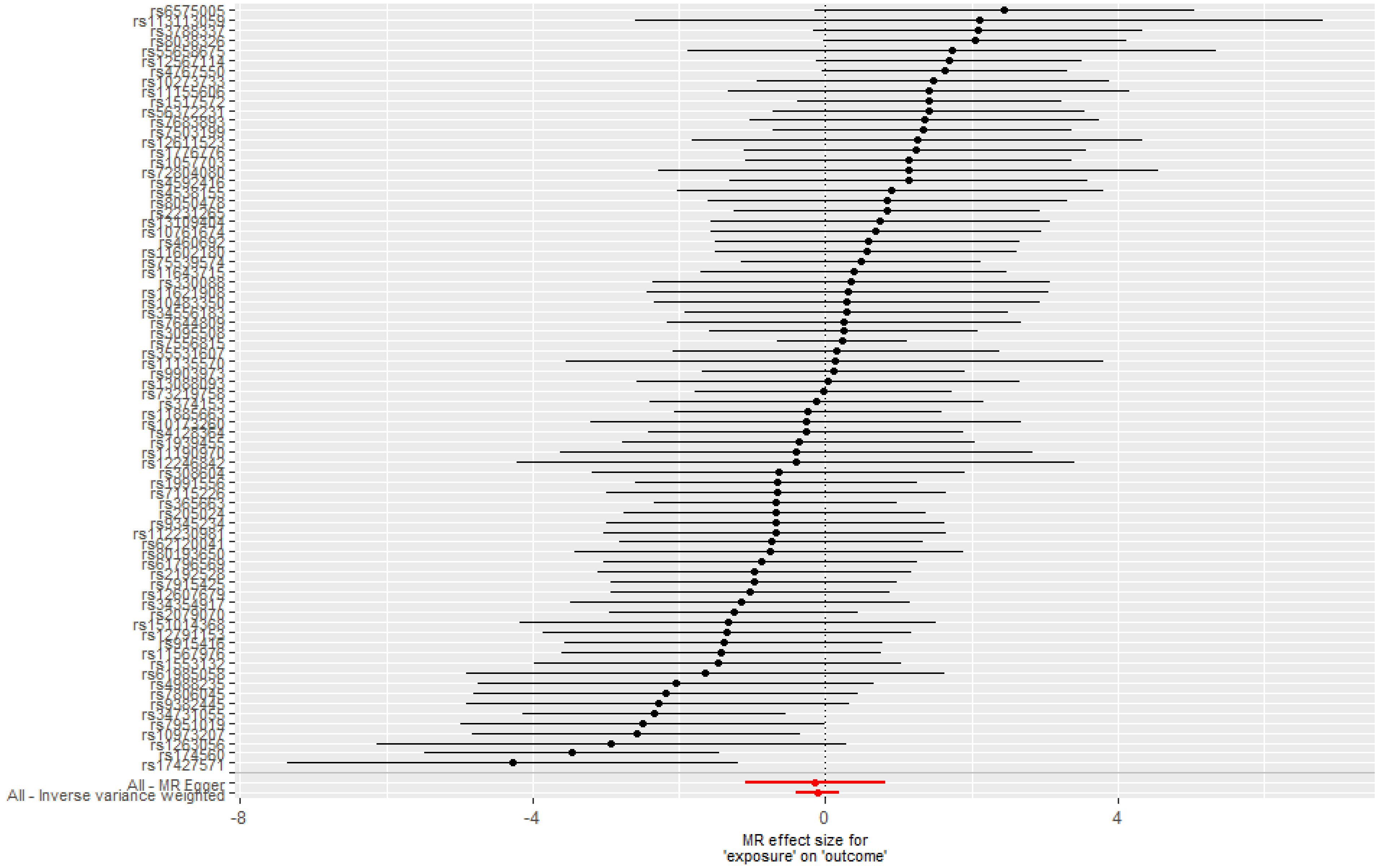

Abbreviations: MR: Mendelian randomization

Supplementary Figure S131. Forest plot of sleep duration and colon cancer association in females

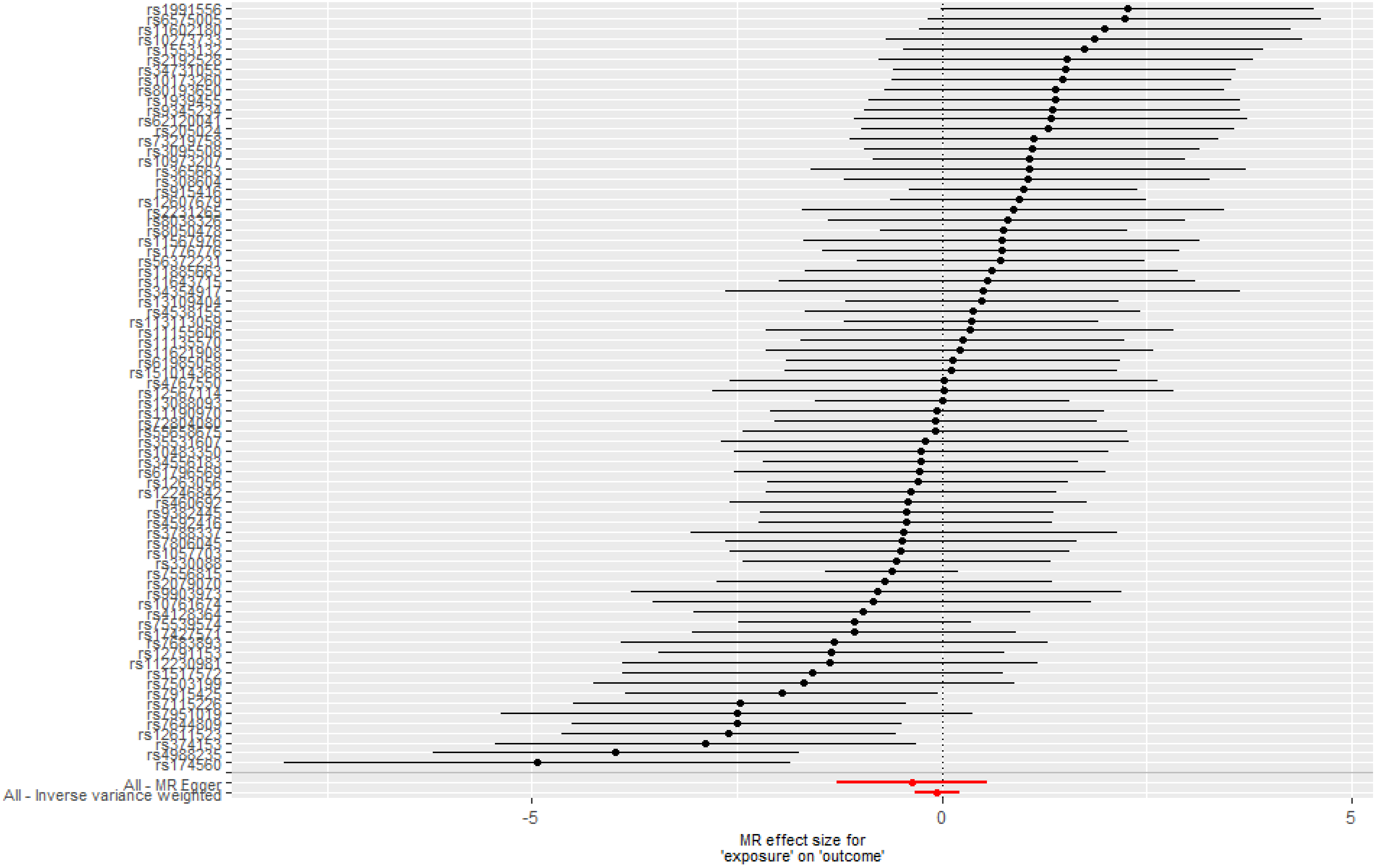

Abbreviations: MR: Mendelian randomization

Supplementary Figure S132. Forest plot of sleep duration and colon cancer association

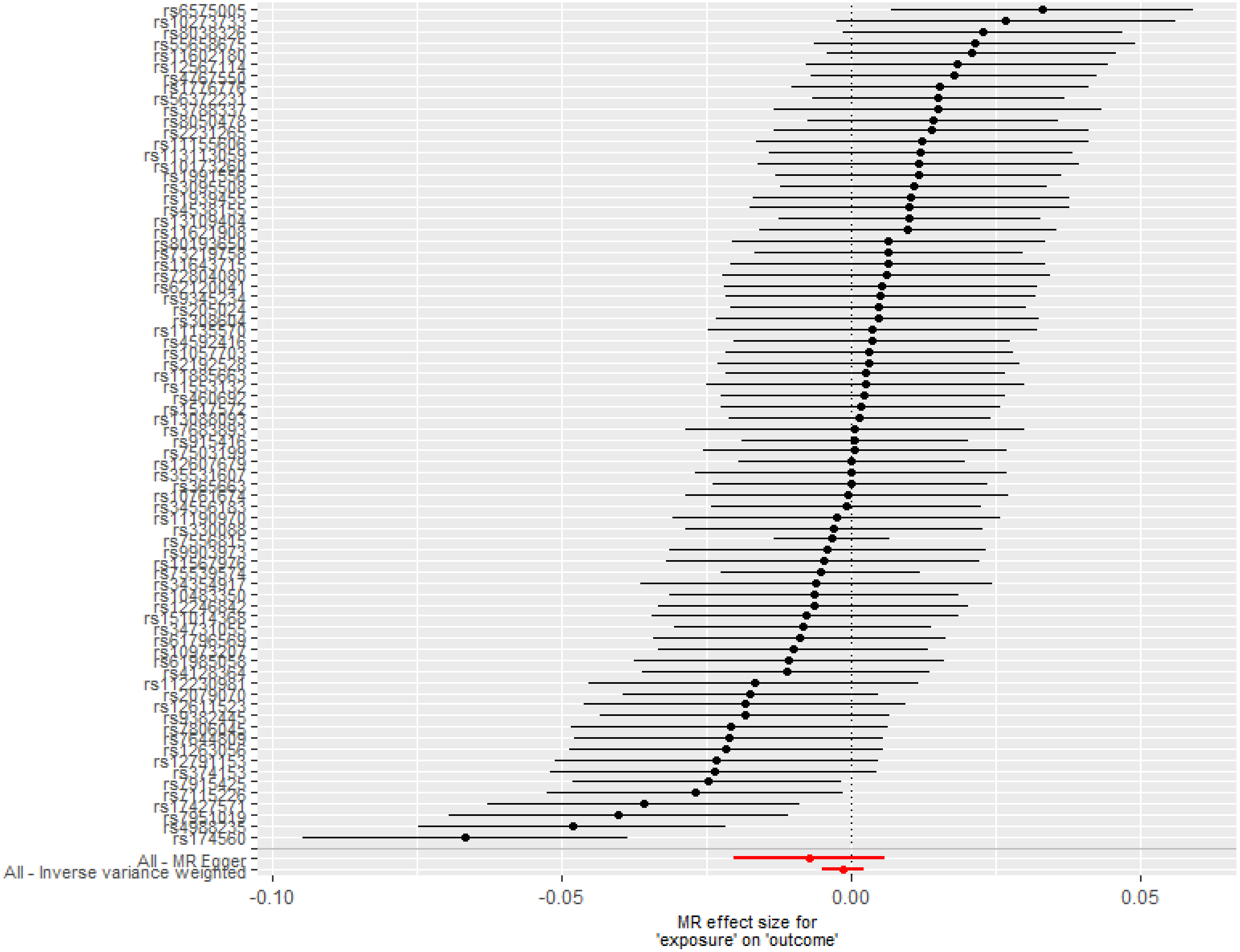

Abbreviations: MR: Mendelian randomization

Supplementary Figure S133. Forest plot of sleep duration and proximal colon cancer association

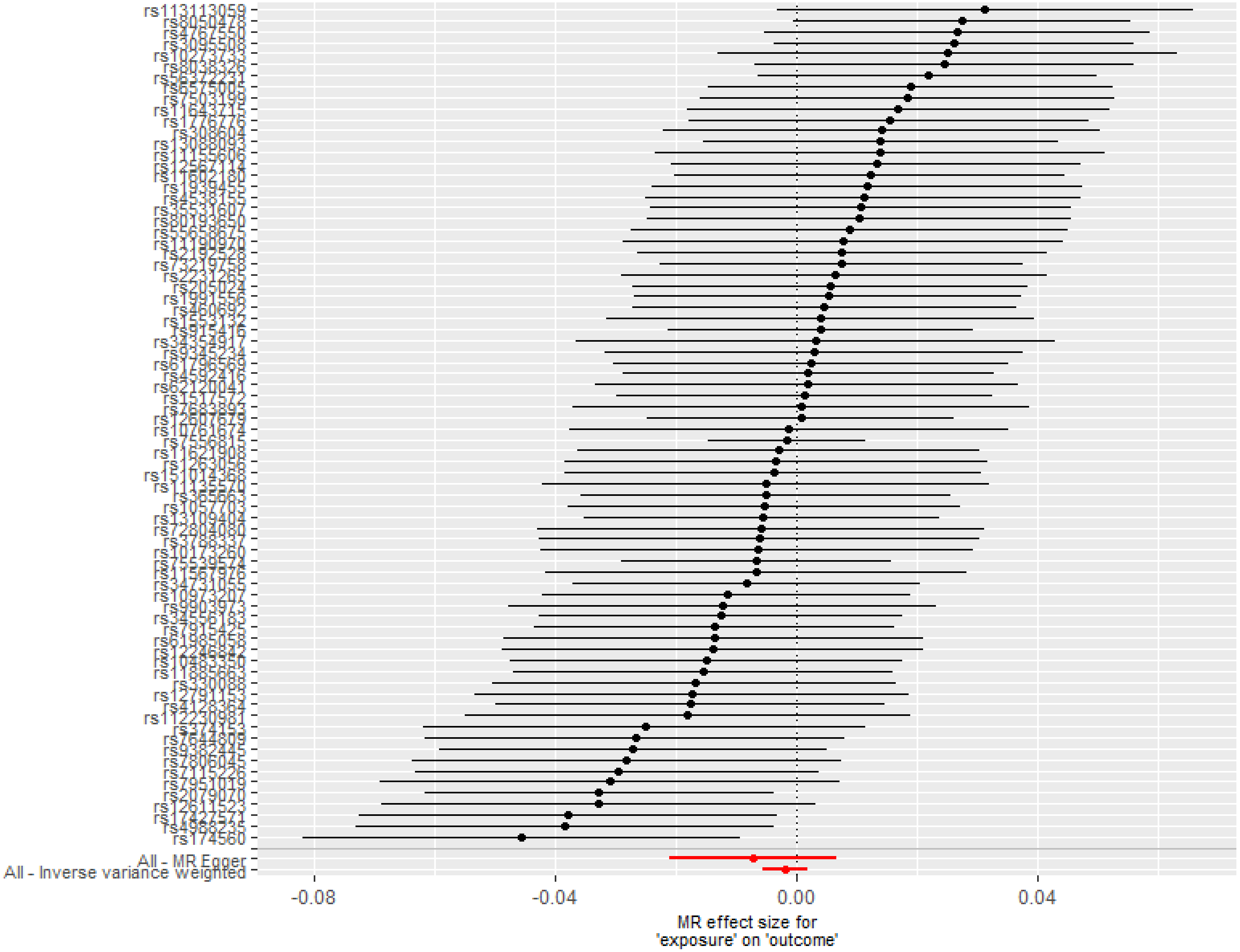

Abbreviations: MR: Mendelian randomization

Supplementary Figure S134. Forest plot of sleep duration and distal colon cancer association

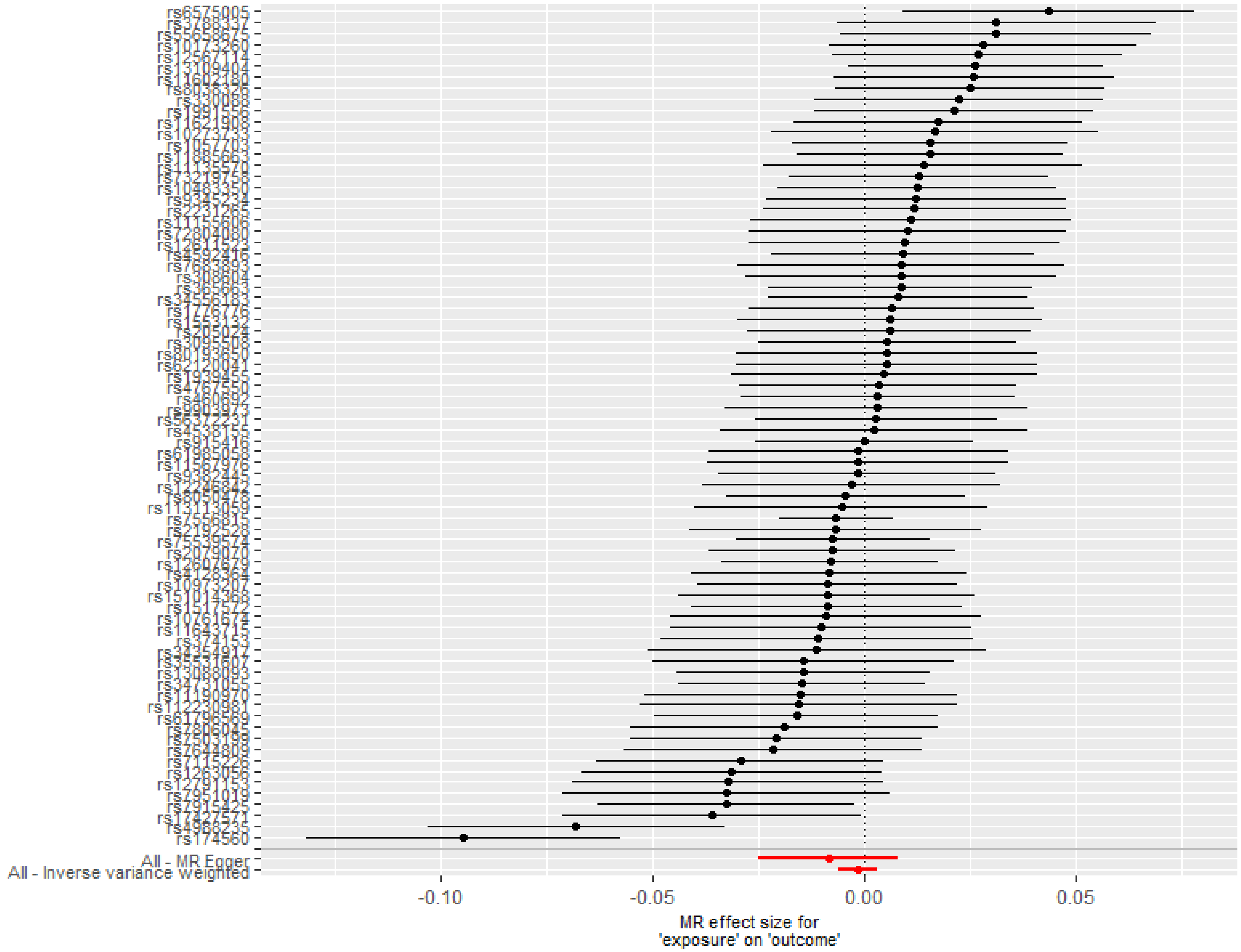

Abbreviations: MR: Mendelian randomization

Supplementary Figure S135. Forest plot of sleep duration and rectal cancer association in males

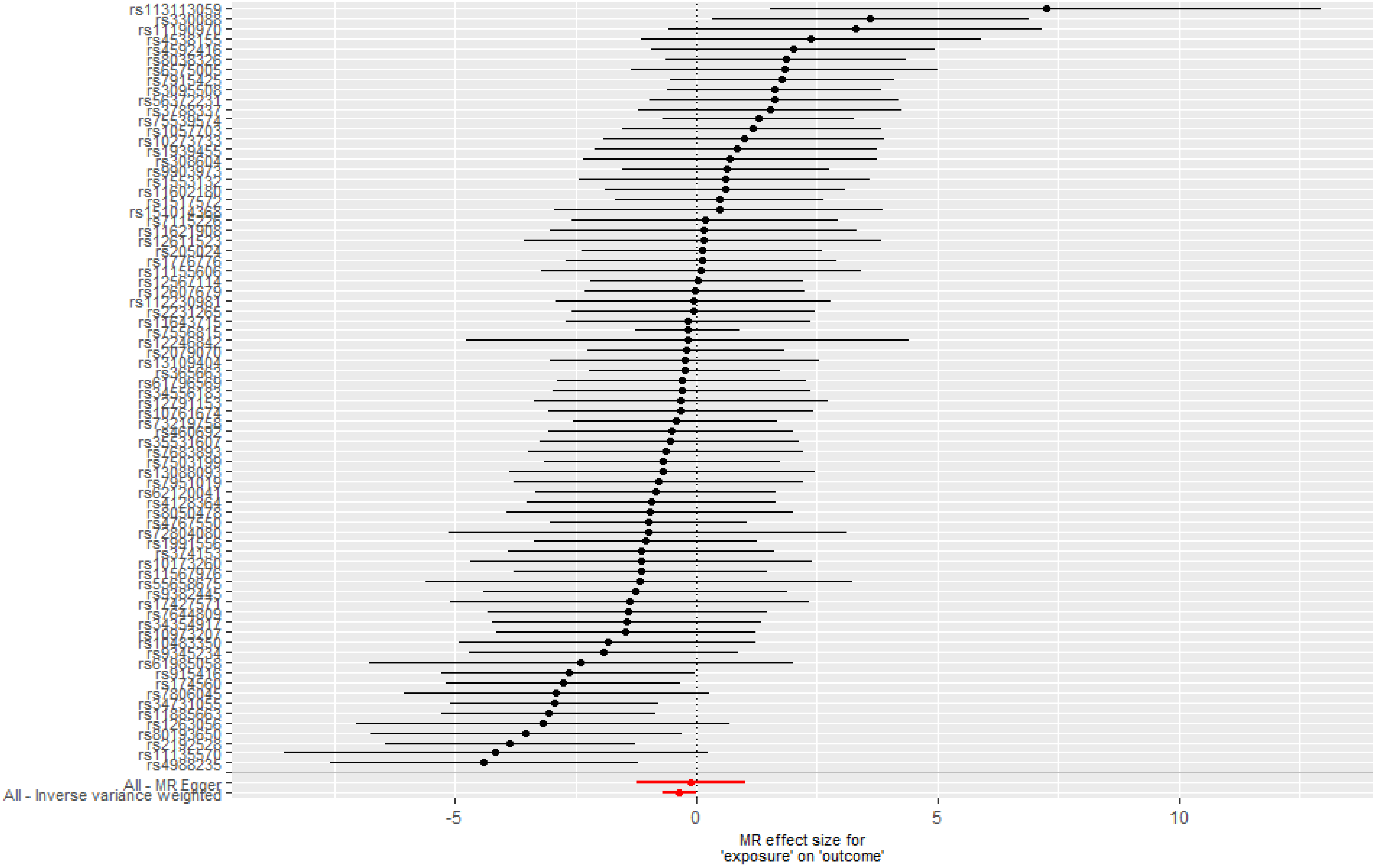

Abbreviations: MR: Mendelian randomization

Supplementary Figure S136. Forest plot of sleep duration and rectal cancer association in females

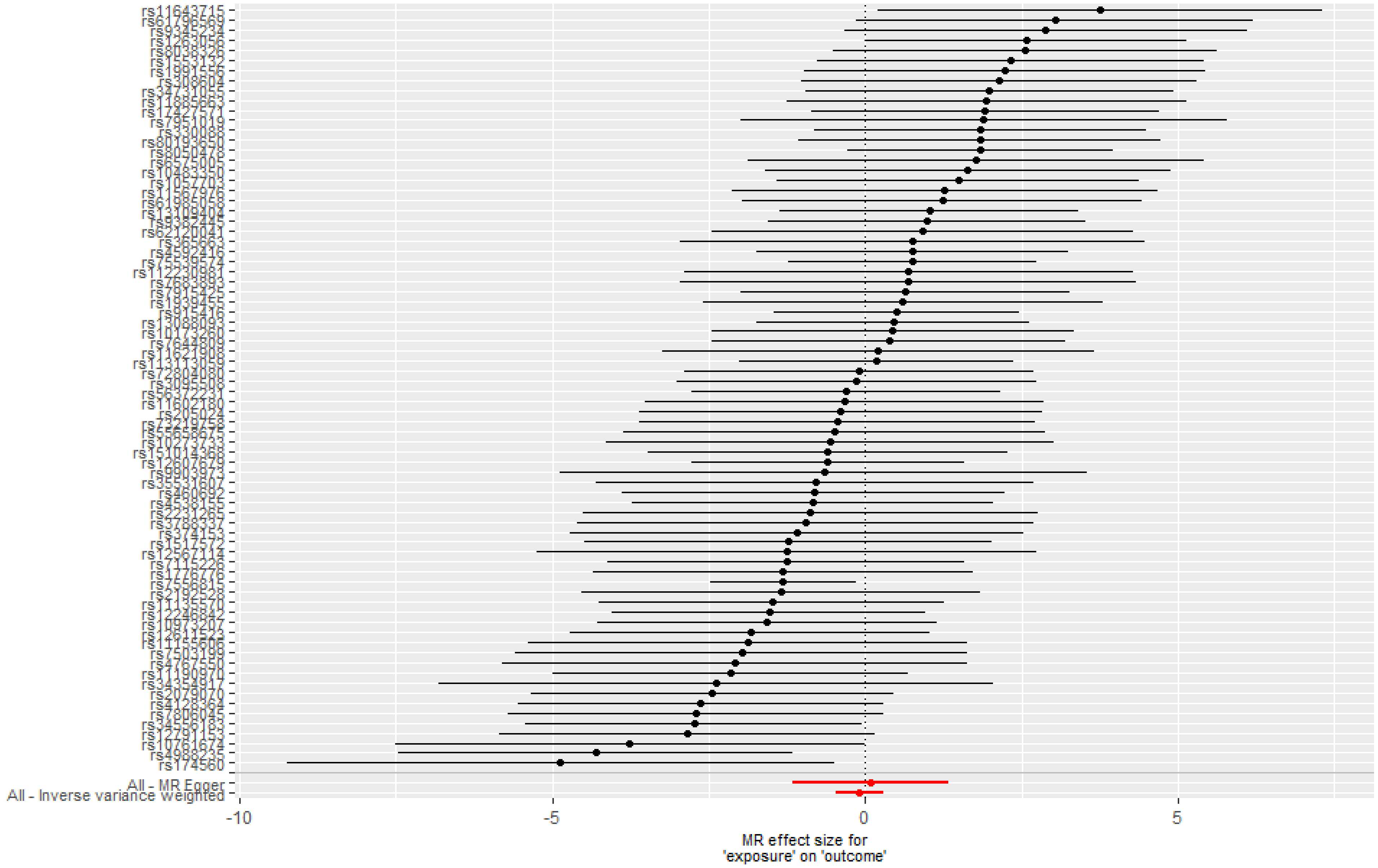

Abbreviations: MR: Mendelian randomization

Supplementary Figure S137. Forest plot of sleep duration and rectal cancer association

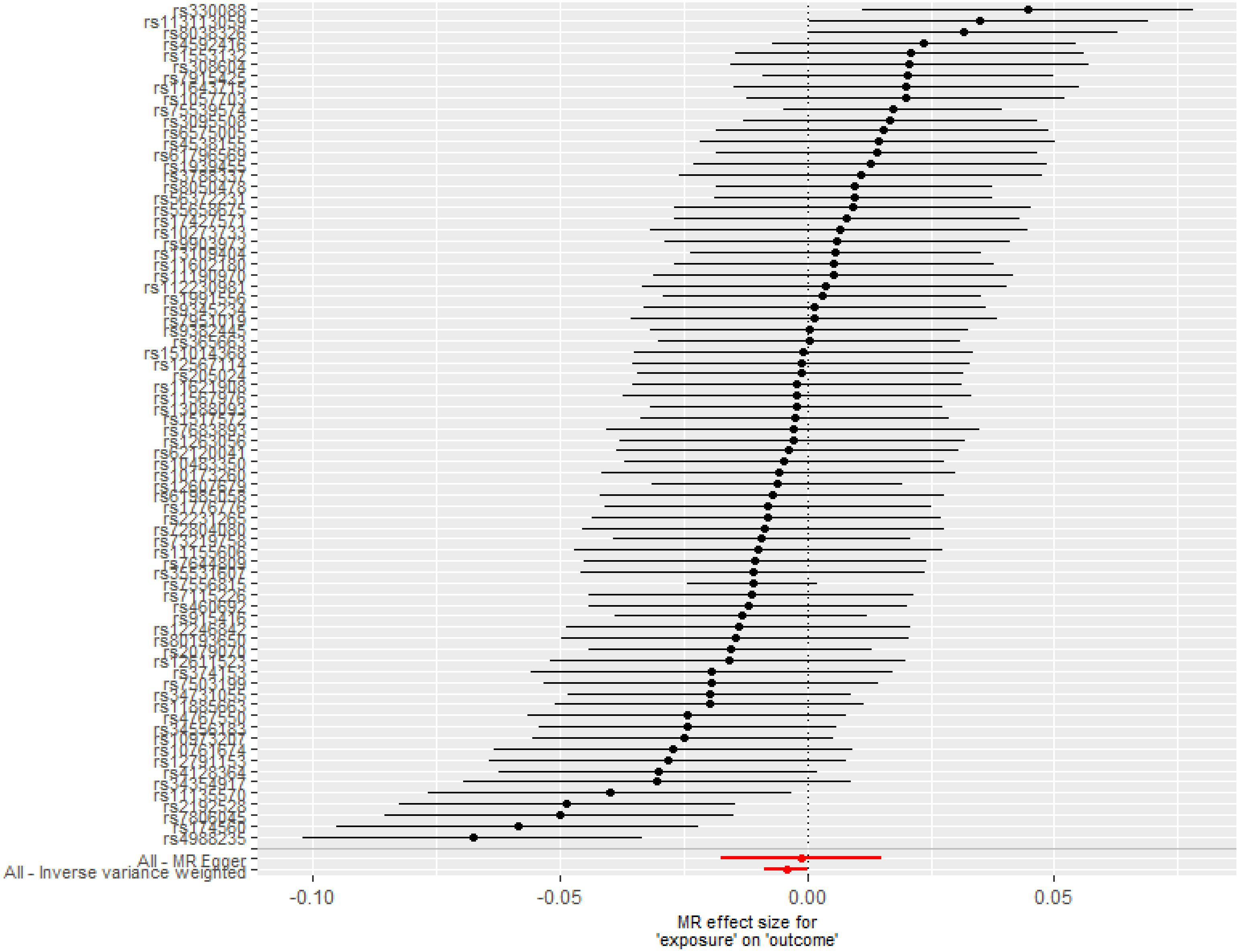

Abbreviations: MR: Mendelian randomization

Supplementary Figure S138. Funnel plot of sleep duration and colorectal cancer association in males

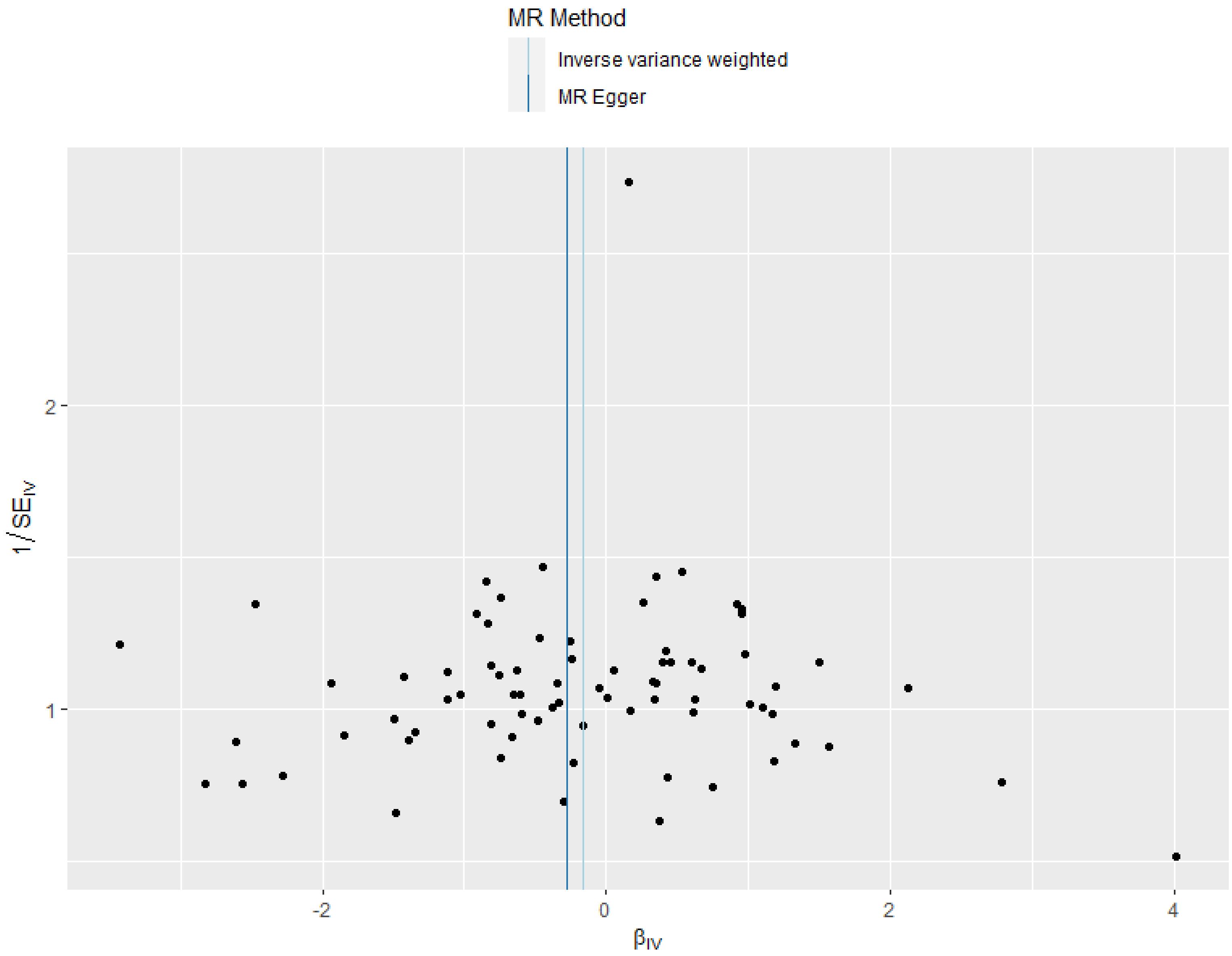

Abbreviations: MR: Mendelian randomization; SE_{IV}: Inverse-variance Standard Error; β_{IV}: Inverse-variance beta coefficient

Supplementary Figure S139. Funnel plot of sleep duration and colorectal cancer association in females

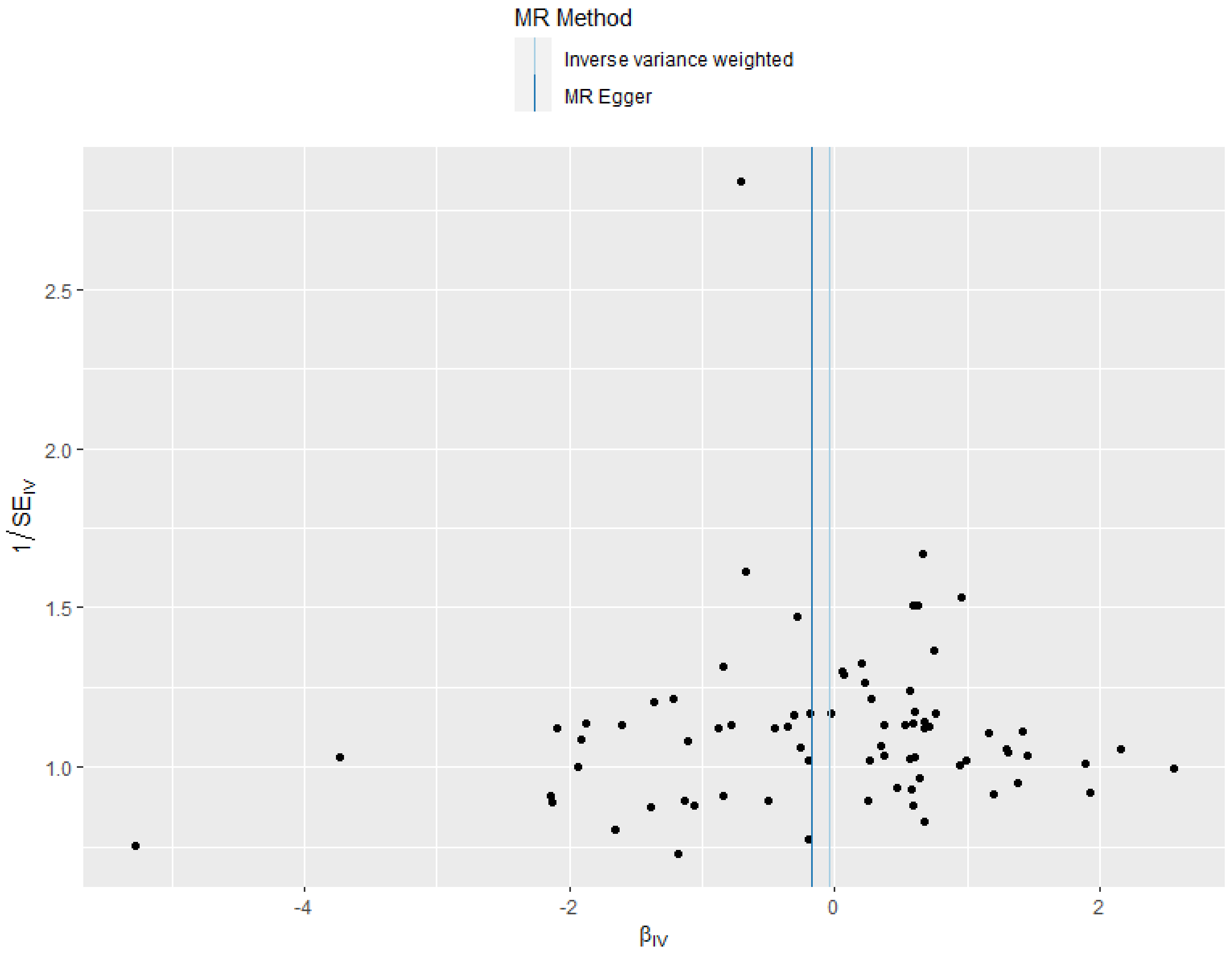

Abbreviations: MR: Mendelian randomization; SE_{IV}: Inverse-variance Standard Error; β_{IV}: Inverse-variance beta coefficient

Supplementary Figure S140. Funnel plot of sleep duration and colorectal cancer association

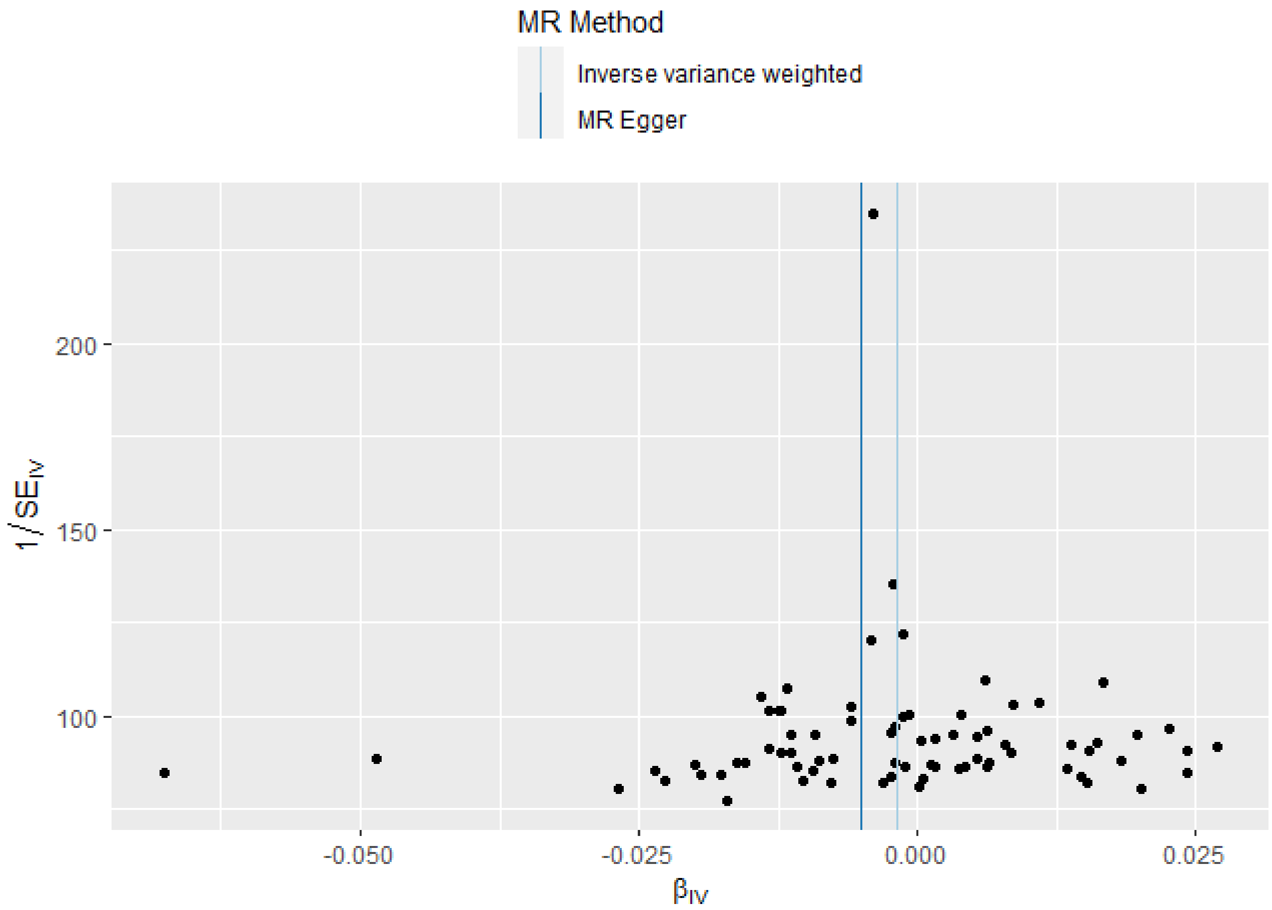

Abbreviations: MR: Mendelian randomization; SE_{IV}: Inverse-variance Standard Error; β_{IV}: Inverse-variance beta coefficient

Supplementary Figure S141. Funnel plot of sleep duration and colon cancer association in males

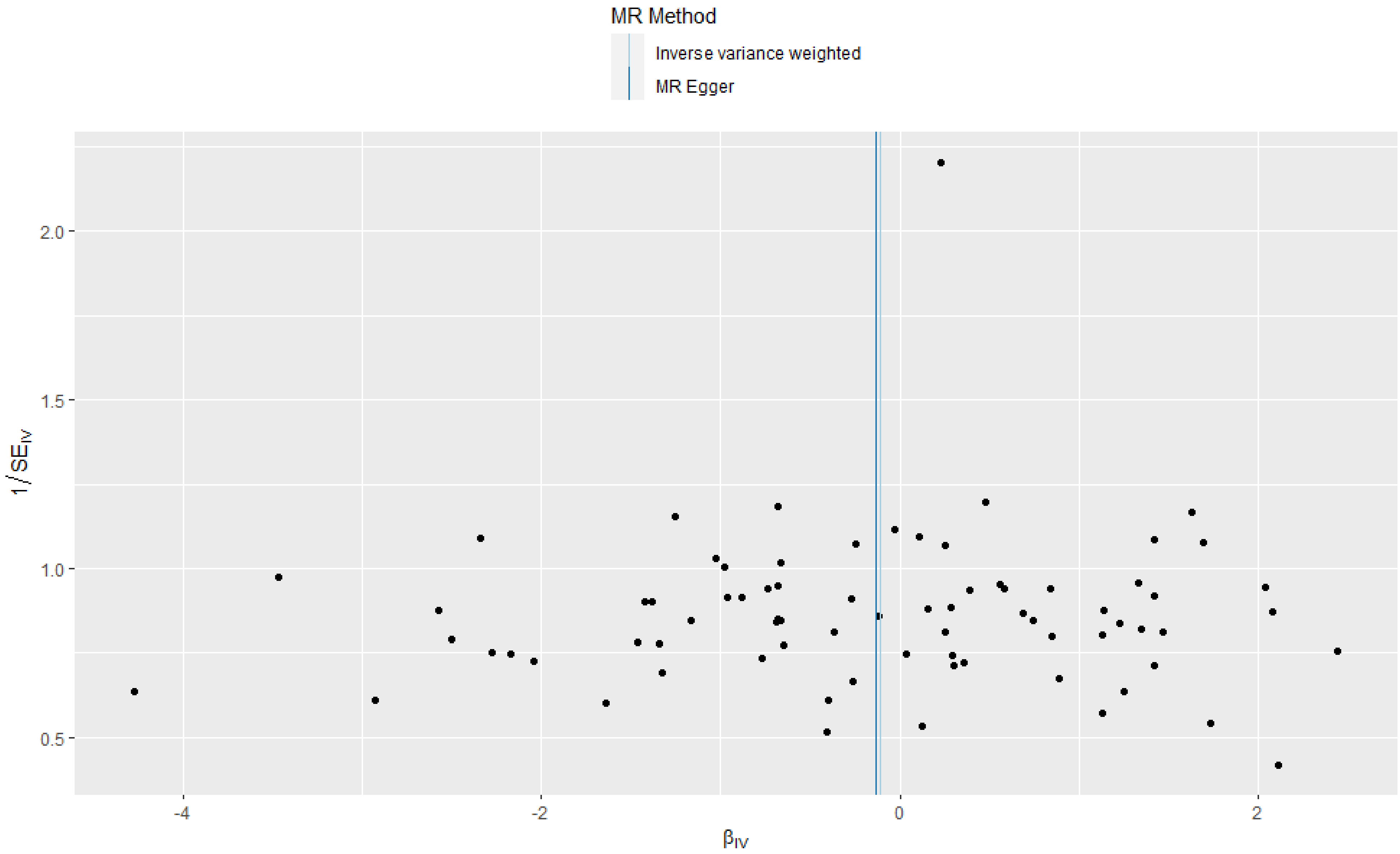

Abbreviations: MR: Mendelian randomization; SE_{IV}: Inverse-variance Standard Error; β_{IV}: Inverse-variance beta coefficient

Supplementary Figure S142. Funnel plot of sleep duration and colon cancer association in females

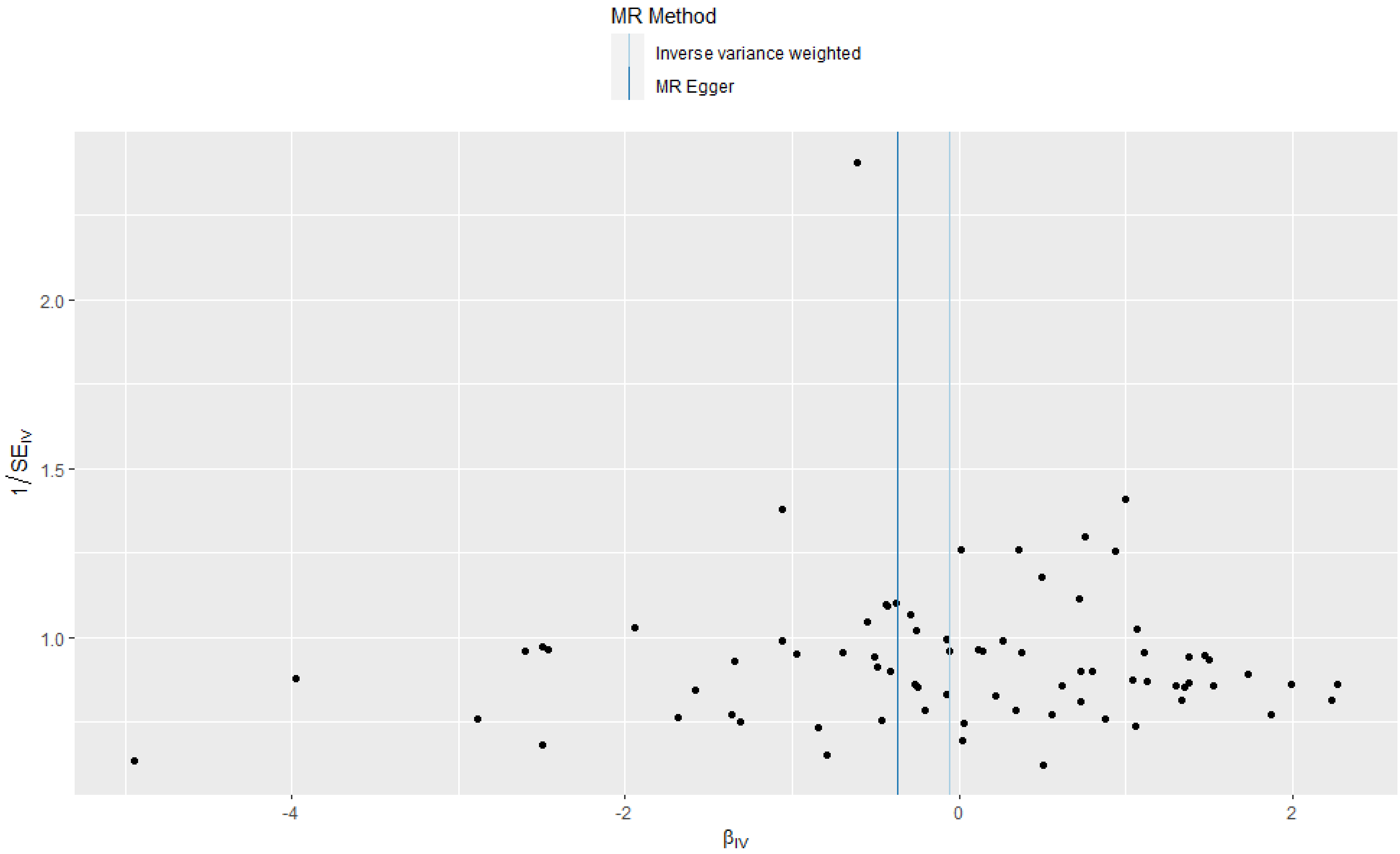

Abbreviations: MR: Mendelian randomization; SE$_{IV}$: Inverse-variance Standard Error; β$_{IV}$: Inverse-variance beta coefficient

Supplementary Figure S143. Funnel plot of sleep duration and colon cancer association

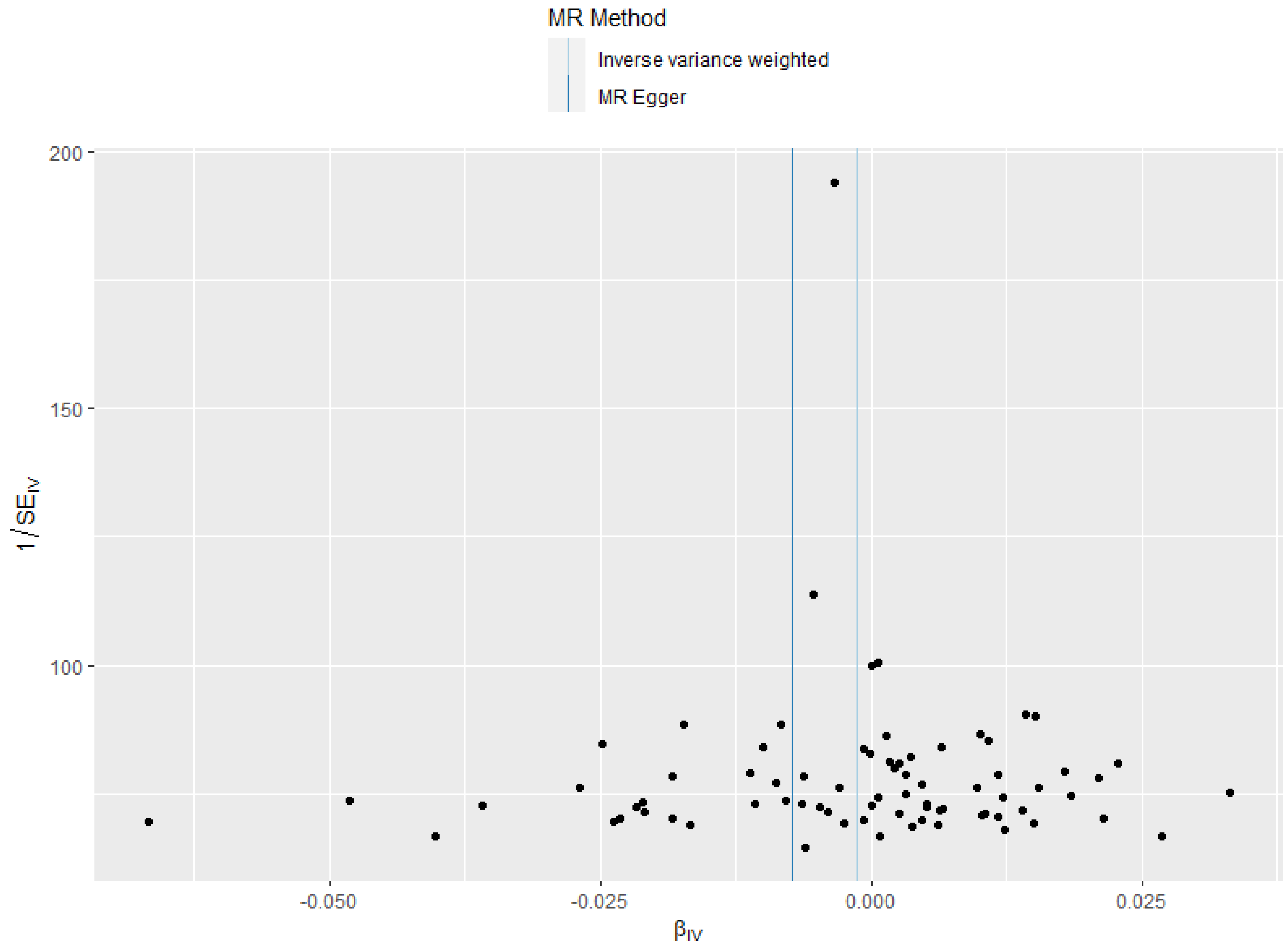

Abbreviations: MR: Mendelian randomization; SE_{IV}: Inverse-variance Standard Error; β_{IV}: Inverse-variance beta coefficient

Supplementary Figure S144. Funnel plot of sleep duration and proximal colon cancer association

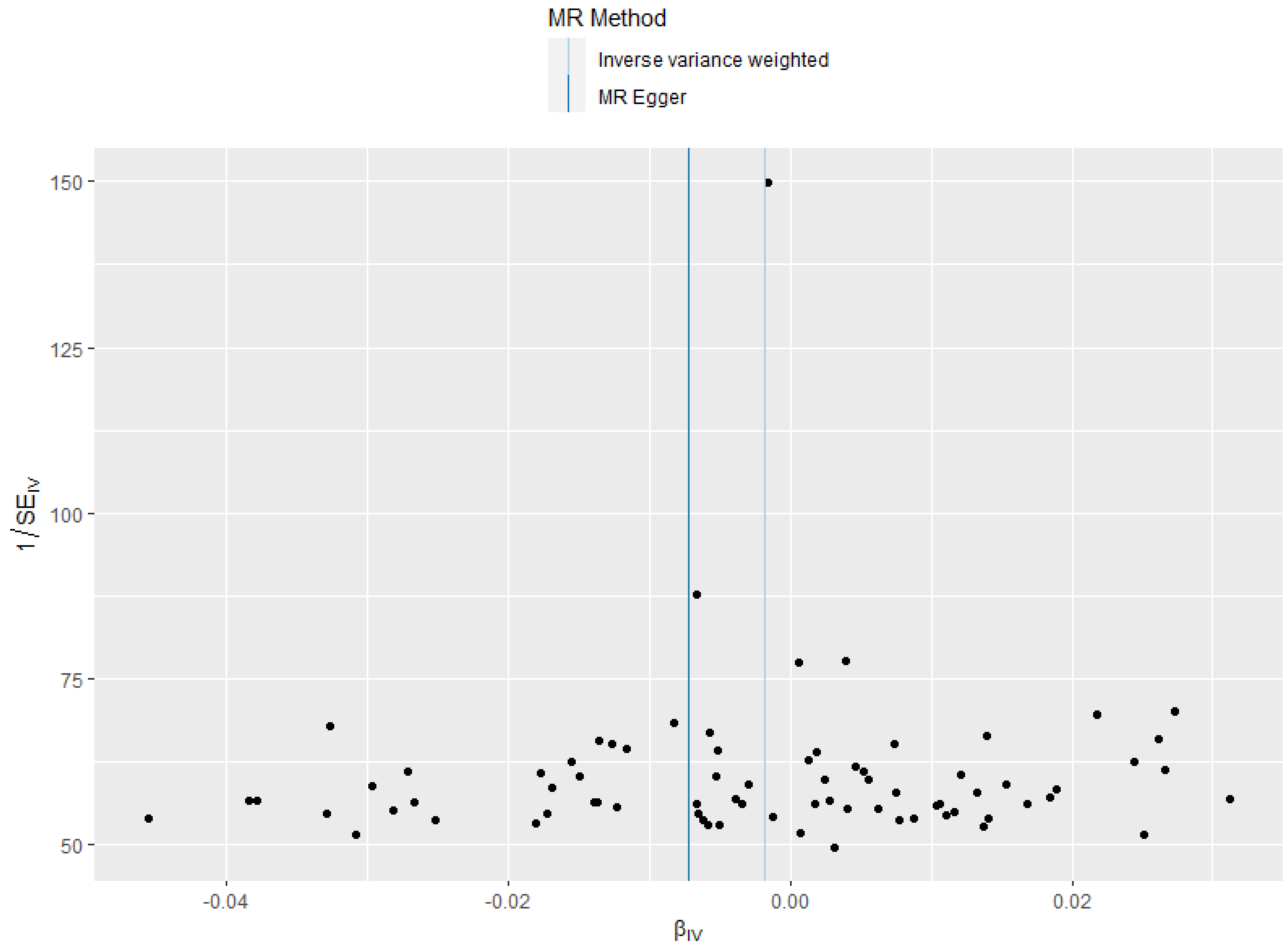

Abbreviations: MR: Mendelian randomization; SE_{IV}: Inverse-variance Standard Error; β_{IV}: Inverse-variance beta coefficient

Supplementary Figure S145. Funnel plot of sleep duration and distal colon cancer association

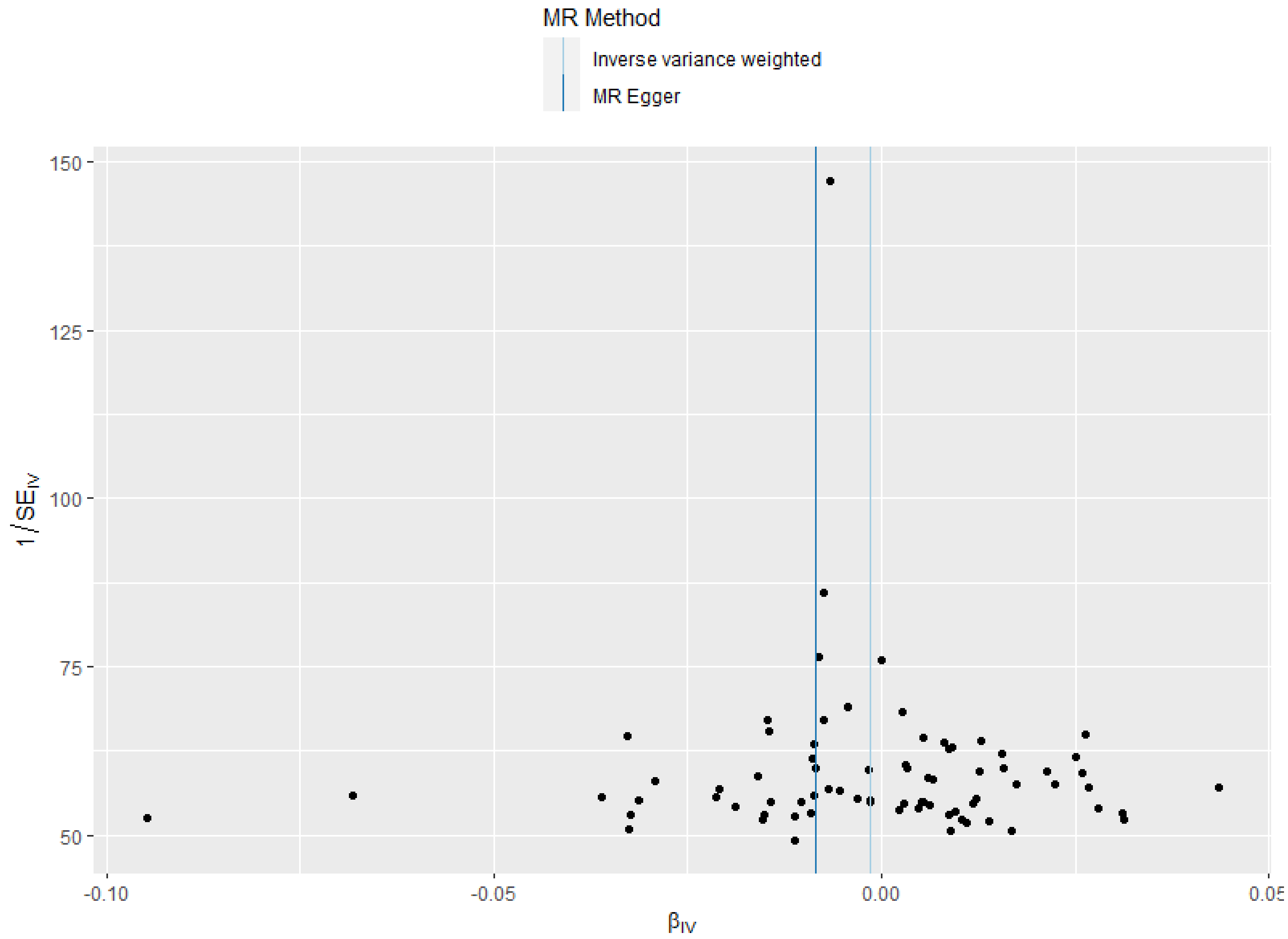

Abbreviations: MR: Mendelian randomization; SE_{IV}: Inverse-variance Standard Error; β_{IV}: Inverse-variance beta coefficient

Supplementary Figure S146. Funnel plot of sleep duration and rectal cancer association in males

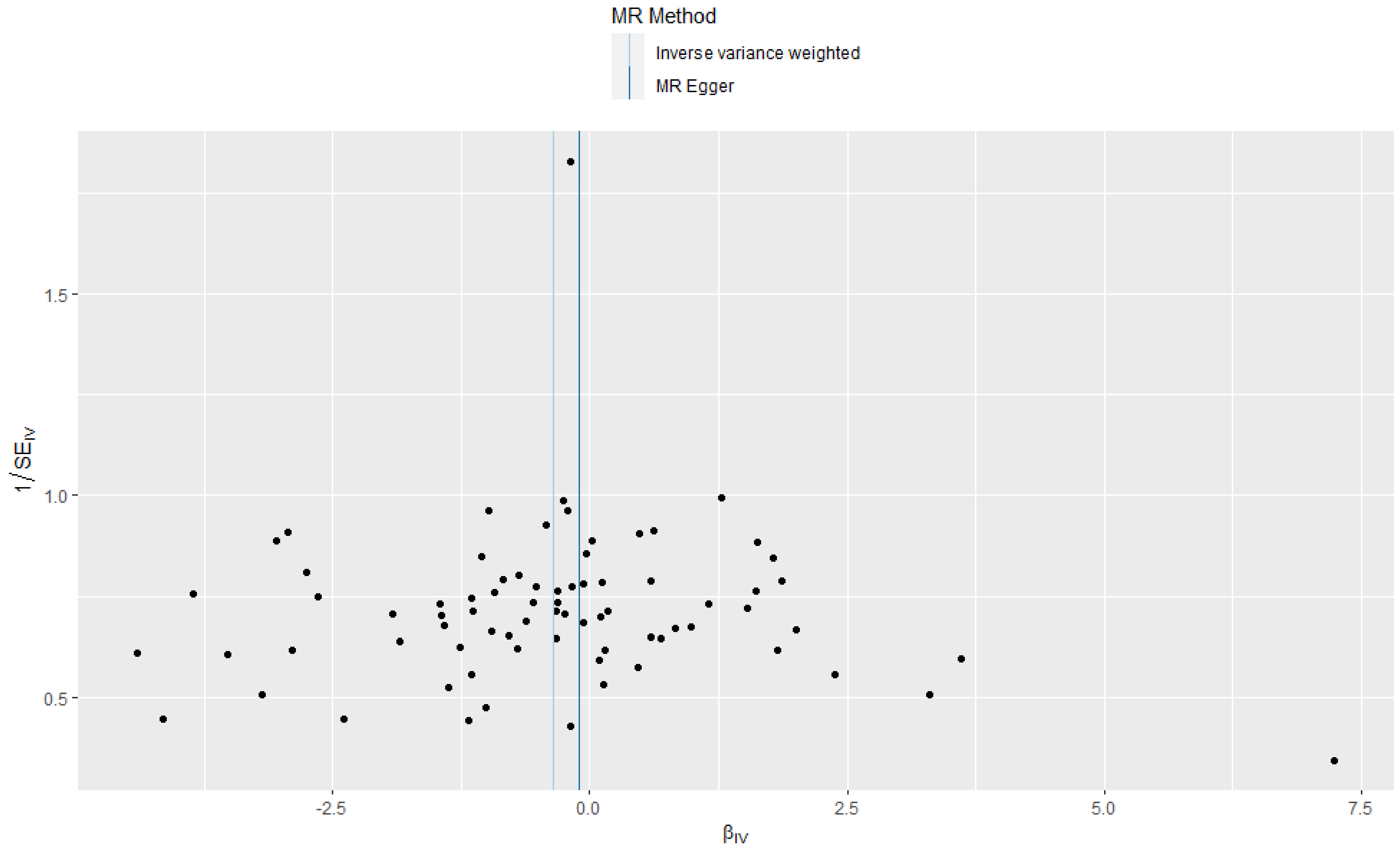

Abbreviations: MR: Mendelian randomization; SE_{IV}: Inverse-variance Standard Error; β_{IV}: Inverse-variance beta coefficient

Supplementary Figure S147. Funnel plot of sleep duration and rectal cancer association in females

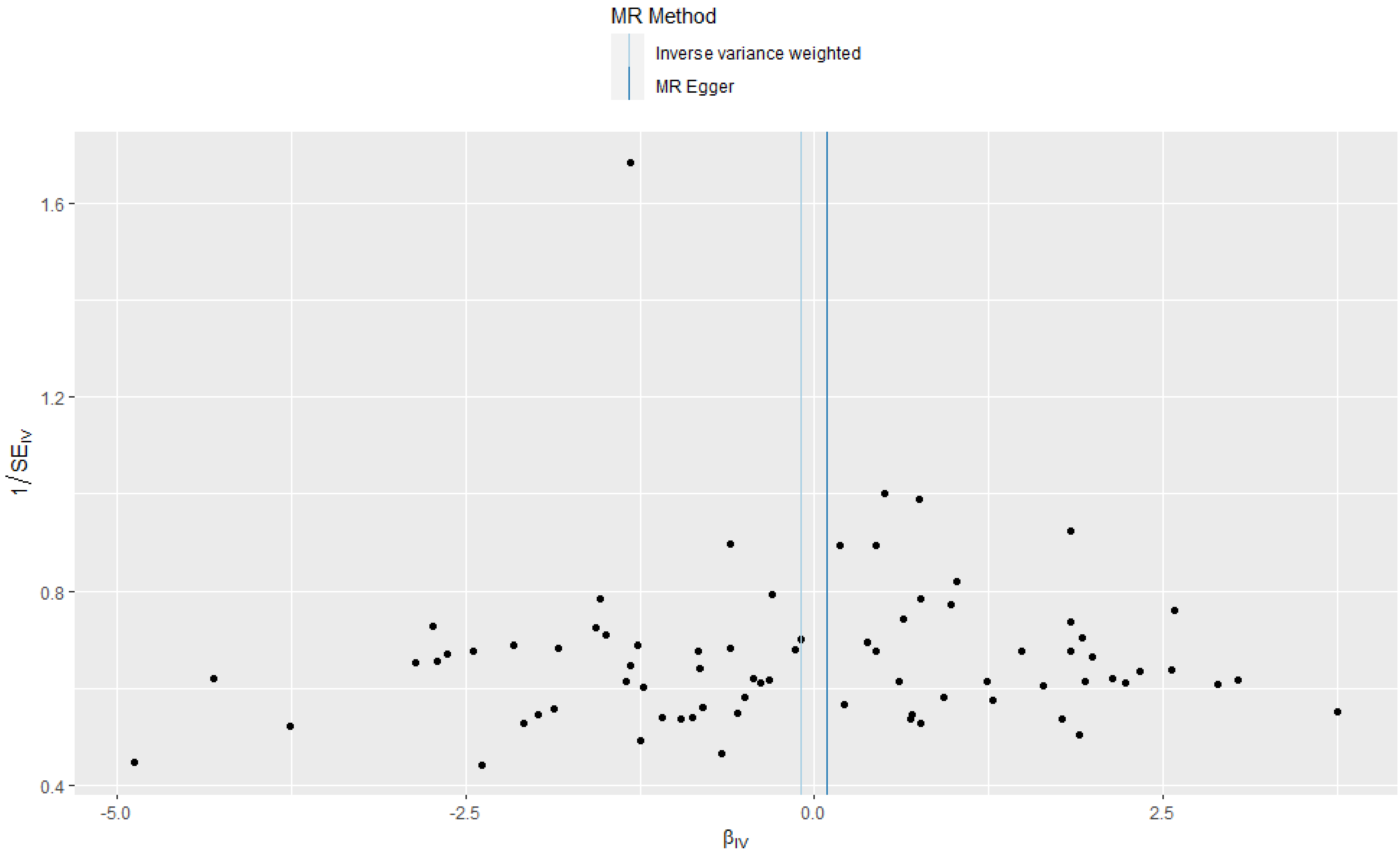

Abbreviations: MR: Mendelian randomization; SE_{IV}: Inverse-variance Standard Error; β_{IV}: Inverse-variance beta coefficient

Supplementary Figure S148. Funnel plot of sleep duration and rectal cancer association

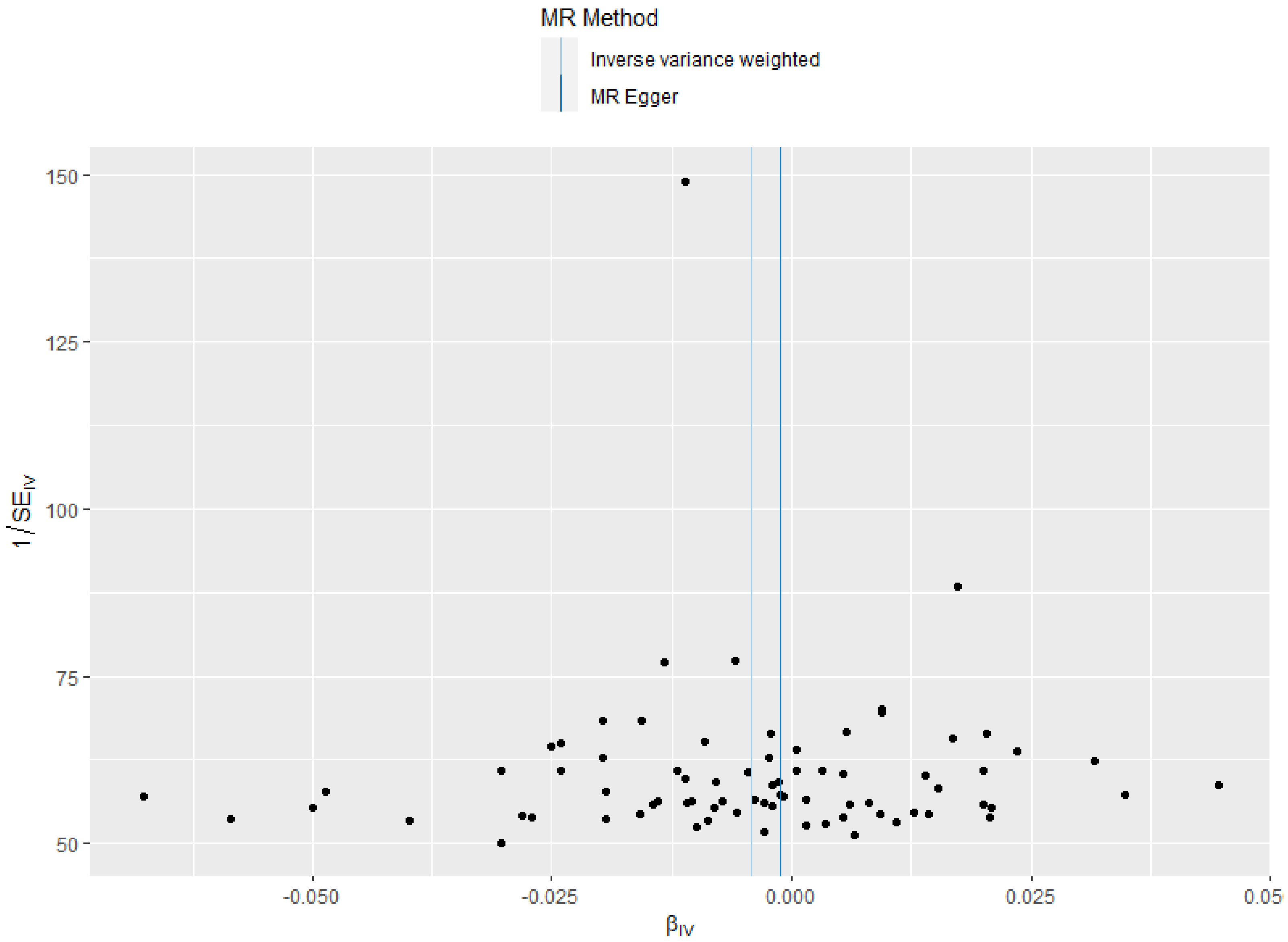

Abbreviations: MR: Mendelian randomization; SE_{IV}: Inverse-variance Standard Error; β_{IV}: Inverse-variance beta coefficient
